# Iridium‐Catalyzed α‐Selective Arylation of Styrenes by Dual C−H Functionalization

**DOI:** 10.1002/anie.201808299

**Published:** 2018-09-26

**Authors:** Phillippa Cooper, Giacomo E. M. Crisenza, Lyman J. Feron, John F. Bower

**Affiliations:** ^1^ School of Chemistry University of Bristol Bristol BS8 1TS UK; ^2^ Medicinal Chemistry Oncology, IMED Biotech Unit AstraZeneca Cambridge UK

**Keywords:** arylation, C−H activation, iridium, styrene

## Abstract

An Ir^I^‐system modified with a ferrocene derived bisphosphine ligand promotes α‐selective arylation of styrenes by dual C−H functionalization. These studies offer a regioisomeric alternative to the Pd‐catalyzed Fujiwara–Moritani reaction.

The intermolecular Heck reaction is the foremost method available for the C−H arylation of alkenes.^1^ For processes involving styrenes, arylation occurs predominantly at the β‐position.^1^, ^2^ In electronically predisposed cases, significant levels of α‐arylation are observed,^3^ but complete selectivity for C−C bond formation at this position can be achieved only under specialized conditions.^4^ The related intermolecular Fujiwara–Moritani reaction, which is most effective in the presence of directing groups, operates under oxidative conditions and is attractive because it achieves C−H arylation of alkenes by dual C−H functionalization, thereby circumventing the preparation of an aryl (pseudo)halide (Scheme 1 A).1c, 5 The regioselectivity trends of this process mirror the Heck reaction, such that the method also does not provide a general approach to the α‐selective arylation of styrenes. This type of selectivity has been observed in rare cases involving heteroarenes, but substrate scope is severely limited.^6^ The paucity of general methods for direct styrene α‐arylation often mandates multistep synthetic workarounds, thereby increasing cost, effort, and waste.^7^

**Scheme 1:**
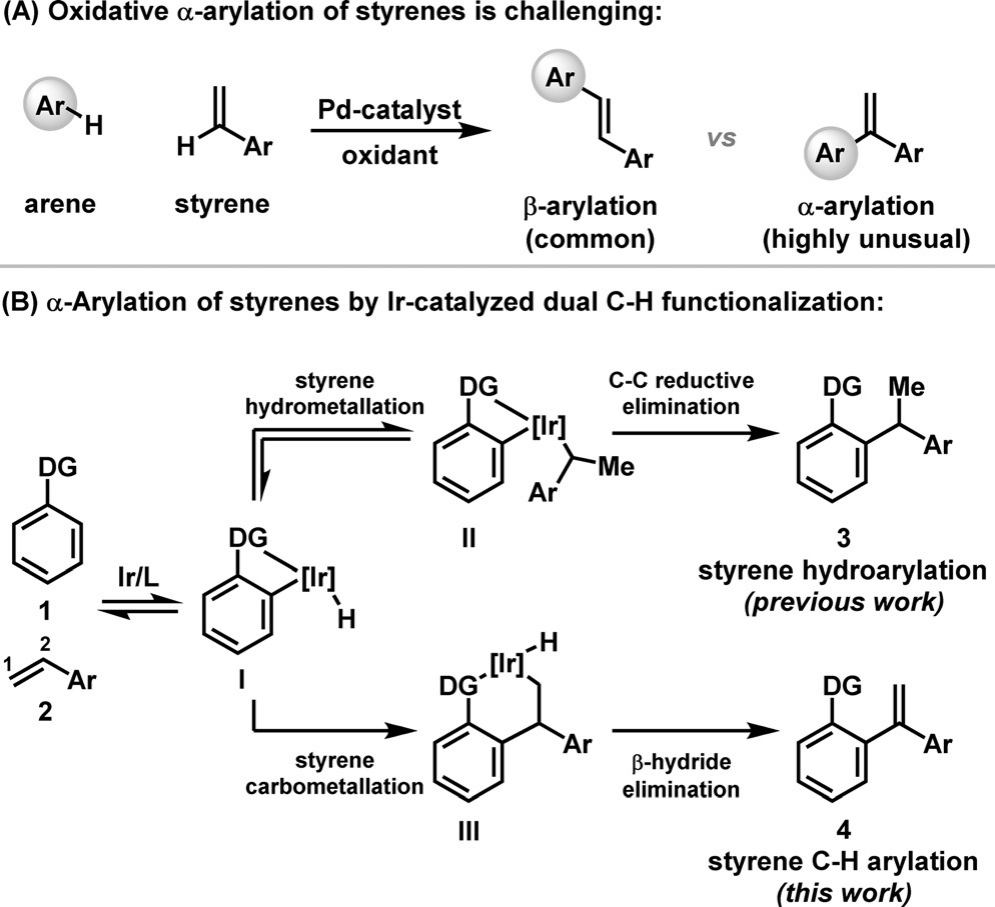
Introduction.

We have previously described Ir‐catalyzed branch selective hydroarylations of styrenes with acetanilides **1** (DG=NHAc, Scheme 1 B).8a These processes were posited to occur via a sequence of carbonyl directed C−H activation (to **I**), alkene hydrometallation (to **II**), and C−C reductive elimination (to **3**). In this report, we show that modification of the Ir‐center with specific bisphosphine ligands alters the reaction outcome to provide a method for the α‐selective arylation of styrenes (**1** to **4**). This new dual C−H functionalization method is regioisomeric with respect to the Fujiwara–Moritani and Heck reactions,^1^, ^5^ and previous Ir‐catalyzed C−H alkenylation processes,^9^, ^10^ thereby providing proof‐of‐concept for a unique approach to the α‐arylation of styrenes. Note that products mechanistically related to **4** have been observed as minor components in enamide C−H alkylation reactions.^11^

Natural abundance ^13^C kinetic isotope effect (KIE) experiments on our previously developed alkene hydroarylation reaction (**1** to **3**, DG=NHAc) are suggestive of a C−C reductive elimination pathway for the formation of **3** (see the Supporting Information).8a,8b, 12, 13 Accordingly, we reasoned that the proposed C−H alkenylation process outlined in Scheme 1 B (**1** to **4**) requires a catalyst system that can enforce access to an alkene carbometallation manifold at the expense of the prevailing C−C reductive elimination pathway. It has previously been shown by Shibata and co‐workers that styrene carbometallation occurs with complete branch selectivity using bisphosphine‐ligated iridacycles derived from C−C bond activation of biphenylene.^14^ Accordingly, if a carbometallative manifold could be accessed, then we expected high regioselectivity for the formation of **III**, which, in turn, would provide α‐arylated styrene **4**, rather than the corresponding β‐arylated isomer (not depicted).

An assay of potential ligand systems was undertaken on the coupling of acetanilide **1 a** and styrene **2 a** using [Ir(cod)_2_]OTf as precatalyst and dioxane as solvent. From these studies, we observed that the use of dppf (**L‐1**) afforded a 3:7 mixture of alkene **4 aa** and hydroarylation product **3 aa**, with the former generated in 23 % yield. This prompted the evaluation of a variety of related ligand systems **L2–L6**, which were prepared in three steps from ferrocene (see the SI).^15^ In general, the selectivity for alkenylation vs. hydroarylation, and the yield of **4 aa** increased as the aromatic unit of the ligand became more electron poor, with **L‐4** providing **4 aa** in 24 % yield and 8:2 selectivity over **3 aa**. However, **L‐5**, which possesses highly electron withdrawing pentafluorophenyl units, did not provide **4 aa**, and instead a mixture of branched and linear hydroarylation products **3 aa** and ***iso*** **‐3 aa** formed.^16^ Ligand systems with more electron rich aromatic units, such as **L‐6**, were not effective, and resulted in hydroarylation only. As **L‐4** provided the highest selectivity for **4 aa,** further optimization studies were undertaken using this ligand. Pleasingly, by increasing the reaction time to 72 hours we found that **4 aa** could be formed in 62 % yield. The conversion of **III** to **4** releases an Ir^III^‐dihydride, and, as indicated by GCMS analysis of crude reaction mixtures, turnover is achieved by reduction of a sacrificial equivalent of styrene to ethyl benzene. As such, we reasoned that oxidants other than styrene might offer additional improvements. In the event, by using 200 mol % *tert*‐butylethylene as an exogenous oxidant^9^ and increasing the catalyst loading to 7.5 mol %, **4 aa** was formed with 10:2 selectivity over **3 aa** and could be isolated in pure form in 74 % yield (further optimization studies with respect to the oxidant are detailed in the SI). For clarity, the product numbering system used in Table 1 is retained throughout subsequent discussion: **3**=hydroarylation product; **4**=C−H arylation product; **first letter** specifies anilide precursor; **second letter** specifies styrene precursor.

**Table 1:** Selected optimization results.

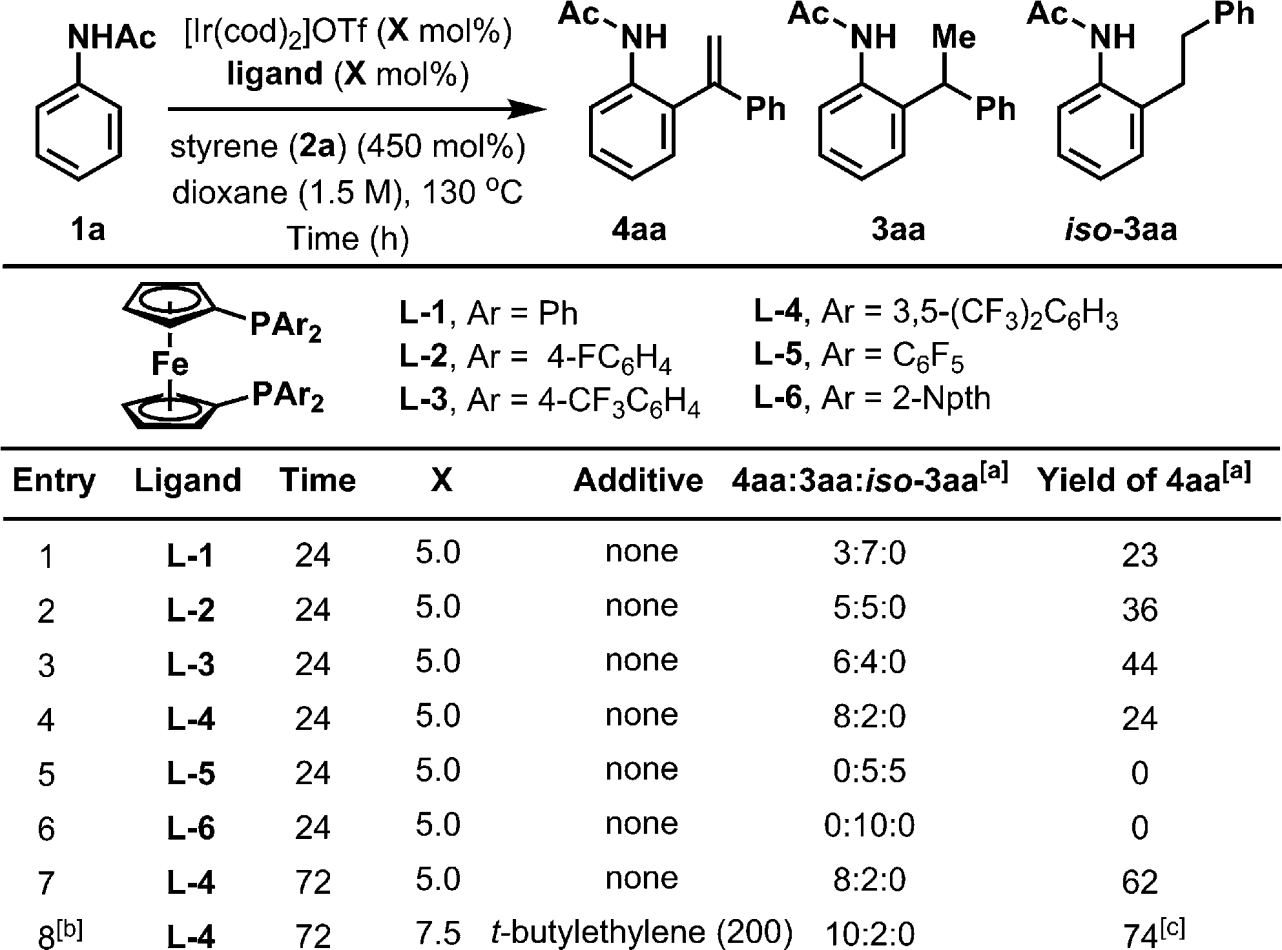

[a] Yields and selectivities were determined by ^1^H NMR analysis using 1,3,5‐trimethoxybenzene as a standard. [b] Reaction run at 0.5 M. [c] Isolated yield.

With optimized conditions in hand, we sought initially to explore scope with respect to the directing group (R^1^, Table 2 A). These studies revealed that a wide range of sterically distinct anilide‐based systems can be employed (**1 a**–**i**), such that **4 aa–ia** were all formed in good to excellent yield, with high selectivity over the corresponding hydroarylation product (**3**).^17^ Note that systems where R^1^=aryl are not suitable because competing *ortho*‐ selective hydroarylation of this unit predominates.8c The process tolerates diverse substitution on the anilide partner (Table 2 B). Indeed, *para*‐substituted systems engage efficiently (**4 oa–rb**), and arenes possessing *meta*‐substitution undergo highly regioselective C−H alkenylation at the less hindered *ortho*‐position; for example, C−H arylation of styrene **2 a** with acetanilide **1 m** provided **4 ma** (65 % yield) as a single regioisomer and with good selectivity over the corresponding hydroarylation product (5:1). The functional group compatibility of the process is good, with, for example, the potentially labile C−Br bond of **4 oa** remaining intact for further diversification. *Ortho*‐substitution can impact selectivity; **4 ua** was formed with only 2:1 selectivity over **3 ua**, but generation of **4 vb** was highly selective. In all cases, the target products were easily separated from the minor hydroarylation products by column chromatography.

**Table 2:** Scope of the anilide component.^[a]^

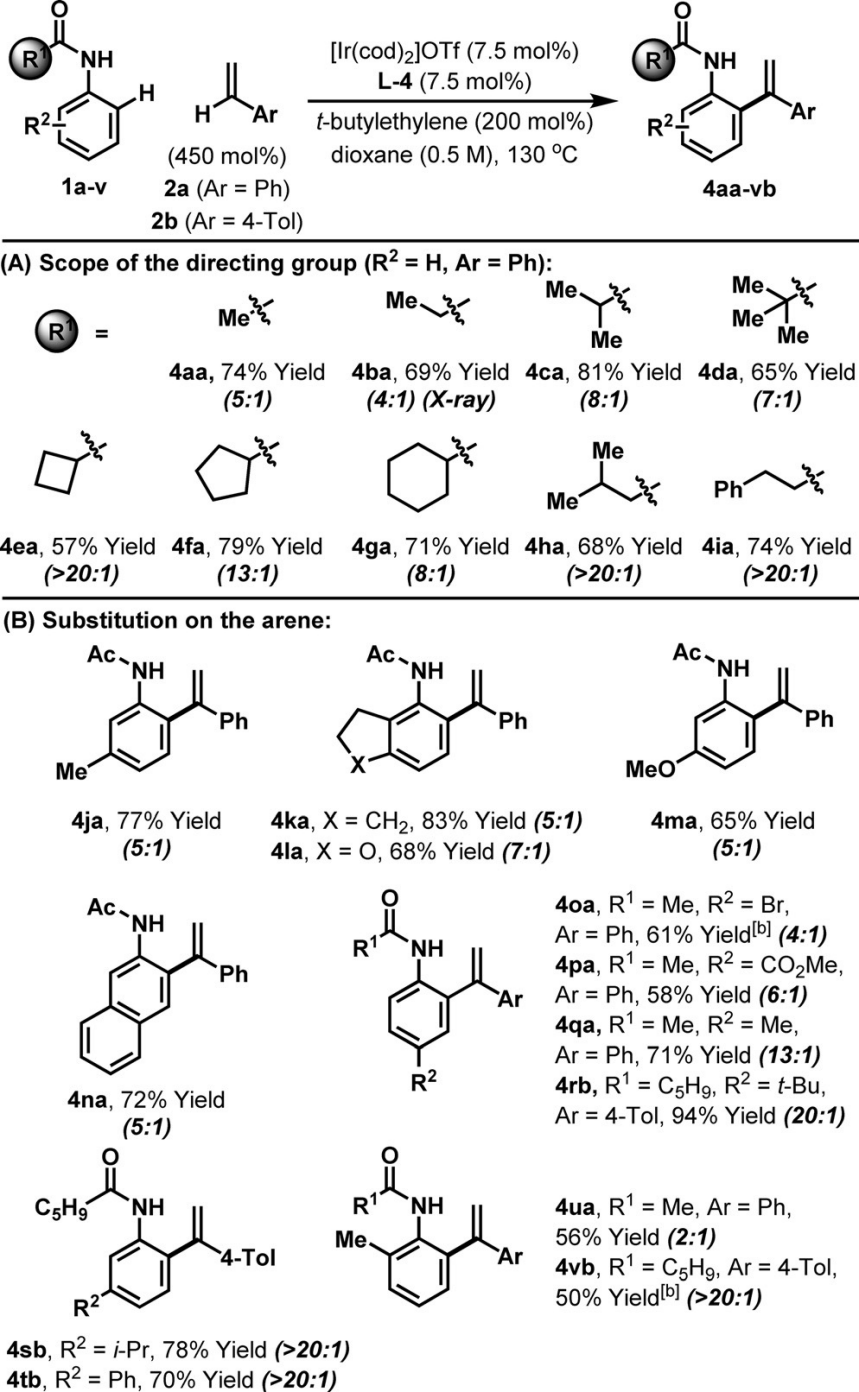

[a] Isolated yields of the C−H arylation product are quoted. Selectivites (C−H arylation product **4**: hydroarylation product **3**) were determined by ^1^H NMR analysis of crude material and are quoted in parentheses. [b] 10 mol % catalyst was used.

Using anilide **1 f**, we have assessed the scope of the alkenylation process with respect to the styrene partner (Table 3). Electronically diverse systems all participate with acceptable levels of efficiency; for example, *para*‐fluoro system **4 fd** and *para*‐methyl system **4 fb** were generated in 75 % and 77 % yield, respectively. An electronic trend is evident for alkenylation vs. hydroarylation selectivity (cf. **4 fb** vs. **4 fe**), however, steric effects are more pronounced. Indeed, *ortho*‐substitution on the styrene lowers product selectivity, such that fluoro system **4 fj** was formed with 2:1 selectivity over the corresponding hydroarylation product. Despite this modest selectivity, analytically pure **4 fj** could still be isolated in 50 % yield. At the present stage, the process is applicable to styrenes only; alkyl substituted alkenes participate with low levels of efficiency with respect to both yield and product selectivity.

**Table 3:** Scope of the styrene.^[a]^

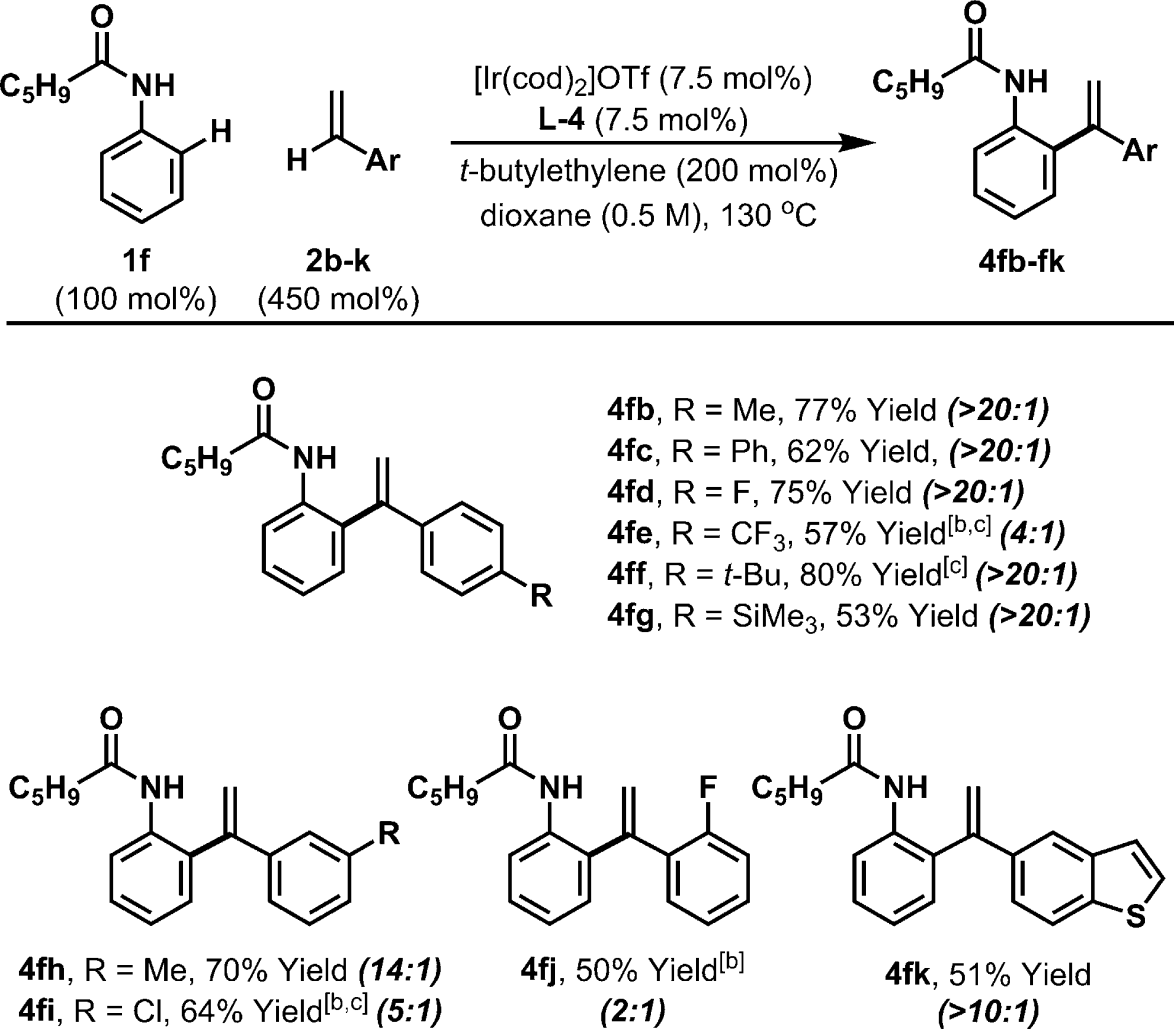

[a] See Table 2 footnotes. [b] 10 mol % catalyst was used. [c] Run at 1.0 M.

The anilide‐based C−H alkenylation products are useful intermediates for synthesis, especially in heterocyclization processes. Treatment of the alkenylation products with POCl_3_ effects smooth cycloaromatization to provide quinolines,^18^ as exemplified by the synthesis of **5 a**–**d** (Scheme 2 A). Note that this de novo heteroaromatization strategy offers high levels of modularity, and its suitability for the construction of challenging polycyclic systems, such as **5 c** and **5 d**, is significant. The protocol even extended to the two‐step conversion of estrone derived acetanilide **1 w** to the unusual quinoidal steroid **5 e**. Other classes of heterocyclization can also be achieved; treatment of **4 aa** with SelectFluor^19^ or iodine^20^ provided adducts **6** and **7**, respectively (Scheme 2 B). Free aniline **8** was accessed by acid hydrolysis of **4 ha** and could be converted in high yield to cinnoline **9** or dihydroquinoline **10**, which possesses a tetrasubstituted stereocenter.

**Scheme 2:**
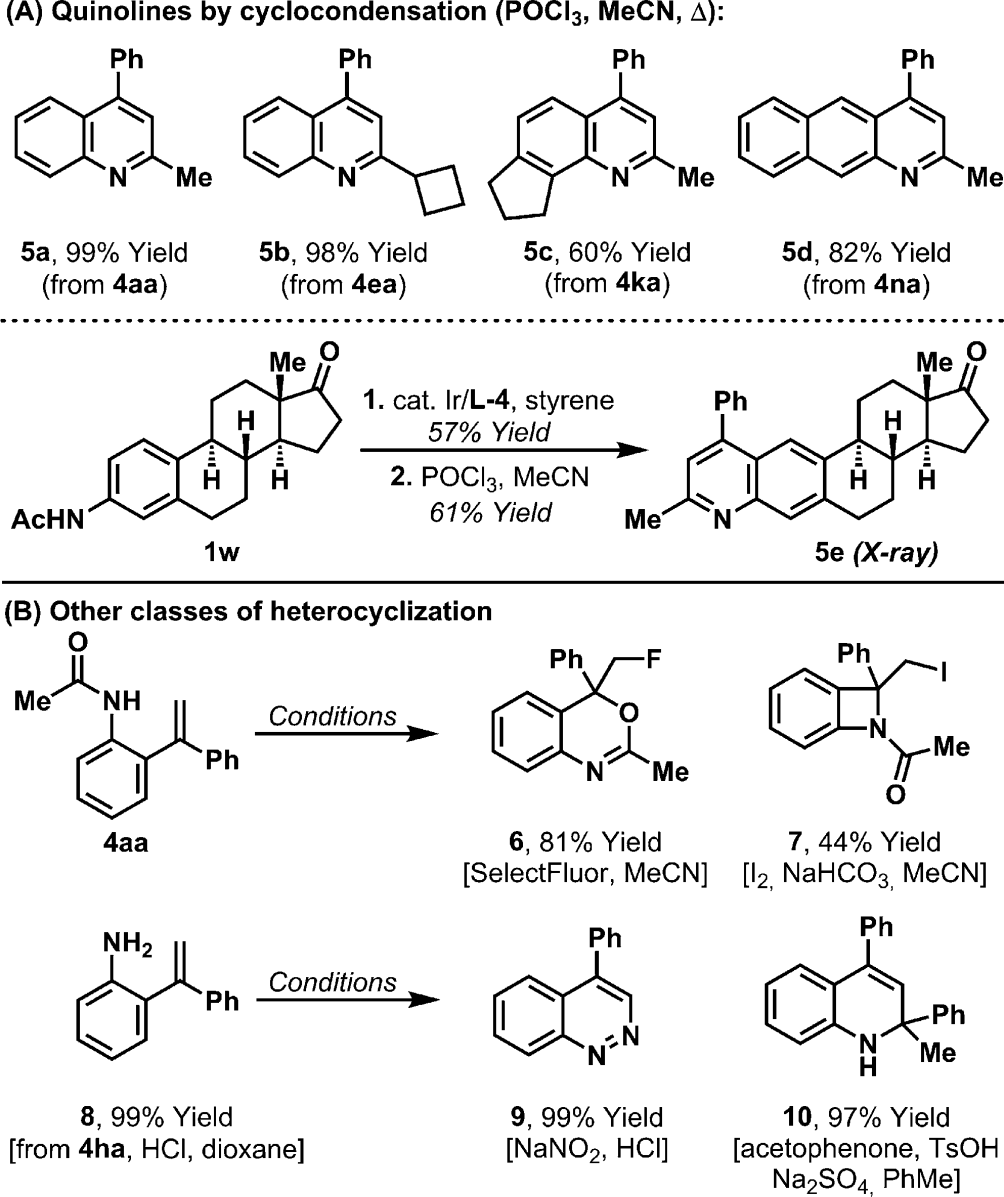
Product derivatizations.

The C−H alkenylation processes outlined here represent proof‐of‐concept for a broader family of styrene α‐arylation protocols. In preliminary efforts to extend the scope of our approach, we have assayed a selection of other aromatic partners leading to the observation that α‐selective arylation using pyrrole **11 a** is feasible (Scheme 3 A). Here, **L‐4** was not a suitable ligand, but, instead, we found that use of ferrocene‐based system (*S*,*S*)‐*f*‐Binaphane^21^ provided targets **12 a–c** in 62–74 % yield. The seemingly fickle nature of the ligand requirements highlights a future challenge in the development of new processes. For pyrrole **11 b**, which is arylated at C3, C−H alkenylation to provide **12 d** was highly regioselective. Using **L‐4** we have also found that dehydrogenative C−C bond formation can be combined with a further dehydrogenation event. When enamide **13** was exposed to optimized conditions dehydrogenative aromatization (to **1 n**) was followed by C−H alkenylation, which provided **4 na** in 60 % yield (Scheme 3 B).^22^

**Scheme 3:**
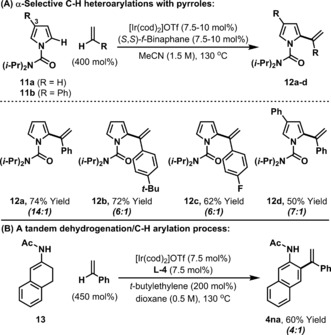
Extensions of the C−H alkenylation process.

It is pertinent to comment on mechanistic details of the processes described here. A control experiment involving resubjection of hydroarylation product **3 aa** to optimized C−H arylation conditions did not provide alkene **4 aa**. This result supports the idea that **4 aa** is generated via a carbometallative pathway (**I** to **III** to **4** in Scheme 1 B) rather than via dehydrogenation of **3 aa**. C−H arylation of *deuterio*‐**2 c** with acetanilide **1 q** resulted in scrambling of the deuterium labels in product *deuterio*‐**4 qc** and in recovered *deuterio*‐**2 c** and **1 q**. This suggests that reversible styrene hydrometallation (**I** to **II**) is also operative under optimized conditions (Scheme 4). Accordingly, the minor alkene hydroarylation products (e.g., **3 aa**) might arise via either C−C reductive elimination from **II** or C−H reductive elimination from **III**. At the present stage we have been unable to discriminate these pathways, such that meaningful rationalizations for product selectivity in each case cannot be made.

**Scheme 4:**
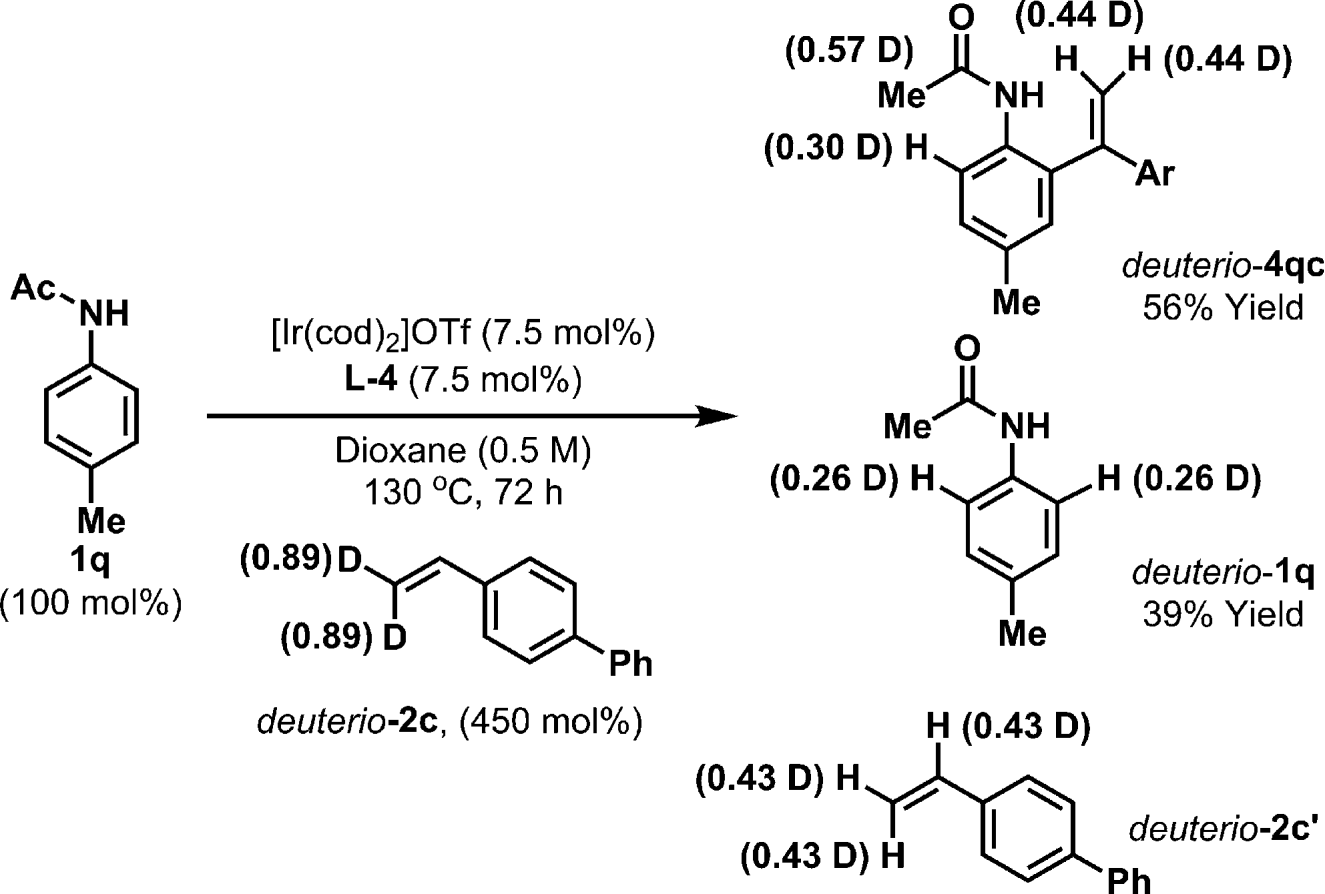
A deuterium labelling study.

In summary, we outline a unique Ir‐catalyzed method for the α‐selective C−H arylation of styrenes. This dual C−H functionalization protocol offers a regioisomeric alternative to the well‐established Pd‐catalyzed Fujiwara–Moritani reaction. Efforts to broaden the utility of the method are ongoing and the results of these studies will be reported in due course.

X‐ray data is available under CCDC 1838966–1838967.

## Conflict of interest

The authors declare no conflict of interest.

## Supporting information

As a service to our authors and readers, this journal provides supporting information supplied by the authors. Such materials are peer reviewed and may be re‐organized for online delivery, but are not copy‐edited or typeset. Technical support issues arising from supporting information (other than missing files) should be addressed to the authors.

Supplementary

## References

[1] Reviews:

[1a] R. F. Heck , Acc. Chem. Res. 1979, 12, 146–151;

[1b] W. Cabri , I. Candiani , Acc. Chem. Res. 1995, 28, 2–7;

[1c] The Mizoroki–Heck Reaction (Ed.: M. Oestreich) Wiley, Chichester, 2009.

[2] For example, see:

[2a] A. F. Littke , G. C. Fu , J. Am. Chem. Soc. 2001, 123, 6989–7000; 11459477 10.1021/ja010988c

[2b] P. M. Murray , J. F. Bower , D. K. Cox , E. K. Galbraith , J. S. Parker , J. B. Sweeney , Org. Process Res. Dev. 2013, 17, 397–405.

[3] Selected examples:

[3a] W. Cabri , I. Candiani , A. Bedeschi , R. Santi , J. Org. Chem. 1992, 57, 3558–3563;

[3b] J. Ruan , J. A. Iggo , N. G. Berry , J. Xiao , J. Am. Chem. Soc. 2010, 132, 16689–16699. 21028842 10.1021/ja1081926

[4a] L. Qin , X. Ren , Y. Lu , Y. Li , J. Zhou , Angew. Chem. Int. Ed. 2012, 51, 5915–5919; 10.1002/anie.201201806 22556044

[4b] Y. Zou , L. Qin , X. Ren , Y. Lu , Y. Li , J. Zhou , Chem. Eur. J. 2013, 19, 3504–3511; 23345260 10.1002/chem.201203646

[4c] L. Qin , H. Hirao , J. Zhou , Chem. Commun. 2013, 49, 10236–10238. 10.1039/c3cc45911j 24060852

[5] Seminal studies:

[5a] I. Moritanl , Y. Fujiwara , Tetrahedron Lett. 1967, 8, 1119–1122;

[5b] Y. Fujiwara , I. Moritani , M. Matsuda , S. Teranishi , Tetrahedron Lett. 1968, 9, 3863–3865;

[5c] Y. Fujiwara , I. Moritani , S. Danno , R. Asano , S. Teranishi , J. Am. Chem. Soc. 1969, 91, 7166–7169; Reviews:27462934 10.1021/ja01053a047

[5d] L. Zhou , W. Lu , Chem. Eur. J. 2014, 20, 634–642; 24356913 10.1002/chem.201303670

[5e] J. Le Bras , J. Muzart , Chem. Rev. 2011, 111, 1170–1214; 21391560 10.1021/cr100209d

[5f] E. M. Beccalli , G. Broggini , M. Martinelli , S. Sottocornola , Chem. Rev. 2007, 107, 5318–5365; Examples of high yielding styrene β-(hetero)arylation:17973536 10.1021/cr068006f

[5g] C. Aouf , E. Thiery , J. L. Bras , J. Muzart , Org. Lett. 2009, 11, 4096–4099; 19691352 10.1021/ol901570p

[5h] A. García-Rubia , M. Á. Fernández-Ibáñez , R. Gómez Arrayás , J. C. Carretero , Chem. Eur. J. 2011, 17, 3567–3570; 21360602 10.1002/chem.201003633

[5i] M. Yu , Z. Liang , Y. Wang , Y. Zhang , J. Org. Chem. 2011, 76, 4987–4994; 21545111 10.1021/jo200666z

[5j] C. Huang , B. Chattopadhyay , V. Gevorgyan , J. Am. Chem. Soc. 2011, 133, 12406–12409; 21766826 10.1021/ja204924j PMC3156791

[5k] P. Gandeepan , C.-H. Cheng , J. Am. Chem. Soc. 2012, 134, 5738–5741; 22432641 10.1021/ja300168m

[5l] X. Cong , J. You , G. Gao , J. Lan , Chem. Commun. 2013, 49, 662–664; 10.1039/c2cc37291f 23146996

[5m] P. Wang , P. Verma , G. Xia , J. Shi , J. X. Qiao , S. Tao , P. T. W. Cheng , M. A. Poss , M. E. Farmer , K.-S. Yeung , J.-Q. Yu , Nature 2017, 551, 489. Metals other than palladium can also be used (See reference [10]). 29168802 10.1038/nature24632 PMC5726549

[6a] M. Ghosh , A. Naskar , S. Mitra , A. Hajra , Eur. J. Org. Chem. 2015, 715–718;

[6b] Y. Yang , K. Cheng , Y. Zhang , Org. Lett. 2009, 11, 5606–5609. 19924879 10.1021/ol902315w

[7] For the processes described here, a classical alternative requires a) the preparation of an acid chloride, b) its use in *ortho*-selective Friedel–Crafts acylation of an anilide and c) olefination of the F/C product. For selected recent alternative approaches to α-arylated styrenes, see:

[7a] J. Tang , D. Hackenberger , L. J. Goossen , Angew. Chem. Int. Ed. 2016, 55, 11296–11299; 10.1002/anie.201605744 27485163

[7b] S. Agasti , A. Dey , D. Maiti , Chem. Commun. 2016, 52, 12191–12194. 10.1039/c6cc07032a 27711336

[8a] G. E. M. Crisenza , O. O. Sokolova , J. F. Bower , Angew. Chem. Int. Ed. 2015, 54, 14866–14870; 10.1002/anie.201506581 PMC4691333 26490739

[8b] S. Grélaud , P. Cooper , L. Feron , J. F. Bower , J. Am. Chem. Soc. 2018, 140, 9351–9356; For related ketone and benzamide directed processes, see:30024748 10.1021/jacs.8b04627

[8c] G. E. M. Crisenza , N. G. McCreanor , J. F. Bower , J. Am. Chem. Soc. 2014, 136, 10258–10261. 25019322 10.1021/ja505776m

[9] A mechanistically similar process allows the internal C−H heteroarylation of α-olefins, but styrenes afford β-heteroarylation products: C. S. Sevov , J. F. Hartwig , J. Am. Chem. Soc. 2014, 136, 10625–10631.25032781

[10] For a review on oxidative couplings of C−H bonds, see: C. Liu , J. Yuan , M. Gao , S. Tang , W. Li , R. Shi , A. Lei , Chem. Rev. 2015, 115, 12138–12204. 26558751 10.1021/cr500431s

[11] D. Xing , G. Dong , J. Am. Chem. Soc. 2017, 139, 13664–13667. 28918637 10.1021/jacs.7b08581

[12] D. A. Singleton , A. A. Thomas , J. Am. Chem. Soc. 1995, 117, 9357–9358.

[13] This interpretation must be treated with caution because we have been unable to devise an acceptable computational model; using a styrene (**2 c**) as the limiting component, a significant ^13^C KIE is observed for C2 but not C1 (see the SI).

[14] H. Takano , K. S. Kanyiva , T. Shibata , Org. Lett. 2016, 18, 1860–1863. 27031498 10.1021/acs.orglett.6b00619

[15] **L-3** and **L-4** have been reported previously:

[15a] O. R. Hughes , D. A. Young , J. Am. Chem. Soc. 1981, 103, 6636–6642;

[15b] B. C. Hamann , J. F. Hartwig , J. Am. Chem. Soc. 1998, 120, 3694–3703.

[16] We speculate that the distinct outcome with this ligand may reflect a switch to a C−C reductive elimination pathway enforced by the highly electron withdrawing pentafluorophenyl units.

[17] The higher selectivities observed with bulkier directing groups might indicate that DG dissociation precedes β-hydride elimination to **4** (cf. **4 ua** vs. **4 vb**); this interpretation must be treated with caution because the pathway to byproducts **3** has not been established.

[18] L. Ni , Z. Li , F. Wu , J. Xu , X. Wu , L. Kong , H. Yao , Tetrahedron Lett. 2012, 53, 1271–1274.

[19] J.-F. Zhao , X.-H. Duan , H. Yang , L.-N. Guo , J. Org. Chem. 2015, 80, 11149–11155. 26472067 10.1021/acs.joc.5b01909

[20] K. Kazuhiro , M. Kazuna , M. Osamu , K. Hisatoshi , Bull. Chem. Soc. Jpn. 2005, 78, 886–889.

[21] D. Xiao , X. Zhang , Angew. Chem. Int. Ed. 2001, 40, 3425–3428; 10.1002/1521-3773(20010917)40:18<3425::aid-anie3425>3.0.co;2-o 11592159

[22] For a recent report on Ir-catalyzed dehydrogenation processes, see: Z. Wang , Z. He , L. Zhang , Y. Huang , J. Am. Chem. Soc. 2018, 140, 735–740. 29251918 10.1021/jacs.7b11351

