# Supplementary material for: Iridium‐Catalyzed α‐Selective Arylation of Styrenes by Dual C−H Functionalization
Source: Angew Chem Int Ed Engl. 2018 Sep 26;57(43):14198–202. doi: 10.1002/anie.201808299 (PMC6391973; doi:10.1002/anie.201808299)
Supplement: Supplementary file 1 — Supplementary [file ANIE-57-14198-s001.pdf]

Angewandte Chemie
Eine Zeitschrift der Gesellschaft Deutscher Chemiker

Supporting Information

Iridium-Catalyzed α-Selective Arylation of Styrenes by Dual C−H Functionalization

*Phillippa Cooper, Giacomo E. M. Crisenza, Lyman J. Feron, and John F. Bower**

anie_201808299_sm_miscellaneous_information.pdf

Supporting Information

Table of Contents

General Experimental Details 2
Experimental Procedures and Data 3
^{13}C-KIE Determination Experiments 3
Ligand Synthesis 8
Substrate Synthesis 11
Selected Reaction Optimization Tables 17
Reaction Scope 18
Product Derivatizations 36
Deuterium Labelling Experiments 47
Copies of ^{1}H and ^{13}C NMR 50
References 107

General Experimental Details

All reagents requiring purification were purified using standard laboratory techniques according to methods published by Perrin, Armarego, and Perrin (Pergamon Press, 1966). Catalytic reactions were carried out in Young-type re-sealable tubes. Styrene was distilled before use. Iridium pre-catalysts were synthesized according to previously reported procedures.[1] Anhydrous solvents were obtained by distillation using standard procedures or by passage through drying columns supplied by Anhydrous Engineering Ltd. Anhydrous 1,4-dioxane was sparged with argon for 10 minutes prior to use. Flash column chromatography (FCC) was performed using silica gel (Aldrich 40-63 µm, 230-400 mesh). Thin layer chromatography was performed using aluminium backed 60 F_{254} silica plates. Visualization was achieved by UV fluorescence or a basic $KMnO_4$ solution and heat. Proton nuclear magnetic resonance spectra (NMR) were recorded at 400 MHz or 500 MHz as stated. ^{13}C NMR spectra were recorded at 100 MHz or 125 MHz as stated. Chemical shifts (δ) are given in parts per million (ppm). Peaks are described as singlets (s), doublets (d), triplets (t), quartets (q), septets (sept), multiplets (m) and broad (br.). Coupling constants (*J*) are quoted to the nearest 0.5 Hz. All assignments of NMR spectra were based on 2D NMR data (DEPT^{135}, COSY, HSQC and HMBC). Where compounds were isolated

as a mixture of isomers (*e.g.* rotamers), they are referred as *A* and *B*. *In situ* yields were determined by employing 1,3,5-trimethoxybenzene as an internal standard. Mass spectra were recorded using a Bruker MicroTof (ESI+ mode) and a Bruker Ultraflex II (MALDI). Infrared spectra were recorded on a Perkin Elmer Spectrum Two FTIR spectrometer as thin films or solids compressed on a diamond plate. Melting points were determined using Reichert melting point apparatus and are uncorrected. Optical rotations were measured using a ADP440$^+$ polarimeter at the concentration and temperature stated.

Experimental Procedures and Data

^{13}C-KIE Determination Experiments (Singleton Method):

Quantitative ^{13}C NMR analysis: All NMR samples were prepared employing ~100 mg of recovered **2c** in 0.7 mL of pre-treated $CDCl_3$ (filtered through a pad of dry K_2CO_3). The ^{13}C NMR spectra were recorded at 126 MHz using inverse gated decoupling and employing a 500 MHz instrument equipped with a CryoProbe™. The spectra were recorded according to the following parameters: 1024 scans, π/6 pulse, 15 s relaxation delay. A total of six spectra were recorded for each sample. The resulting six FIDs were processed at the same time applying the same phase correction, a fifteenth order polynomial fit baseline correction and 256K zero filling. Integrations were numerically determined using a constant region for each peak corresponding to five times of the peak widths at half height (± $5w_{1/2}$). The peak belonging to C**6** of **2c** was chosen as the internal standard and was set with an integration of 1000.

Formulae applied for the determination of ^{13}C KIEs: The formulas employed in the calculations for the determination of the KIE were reported by Saunders[2] and Singleton[3] and are summarized as follows:

F = conversion of starting material.

R/R$_0$ = proportion of the minor isotopic component in recovered material compared to the original starting material.

Δ(R/R$_0$) = $R/R_0((\Delta R/R)^2 + (\Delta R_0/R_0)^2)^{1/2}$

$$\textbf{KIE} = \frac{\ln(1-F)}{\ln[(1-F)R/R_0]}$$

$$\Delta\text{KIE}_F = \frac{\partial KIE}{\partial F}\,\Delta F = \frac{-\ln(R/R_0)}{(1-F)\ln^2[(1-F)R/R_0]}\,\Delta F$$

$$\Delta\text{KIE}_R = \frac{\partial KIE}{\partial(R/R_0)}\,\Delta(R/R_0) = \frac{-\ln(1-F)}{(R/R_0)\ln^2[(1-F)R/R_0]}\,\Delta R/R_0$$

ΔKIE = $KIE*((\Delta KIE_R/KIE)^2 + (\Delta KIE_F/KIE)^2)^{1/2}$

^{13}C-KIE determination in the branch-selective hydroarylation of 1l with 2c:

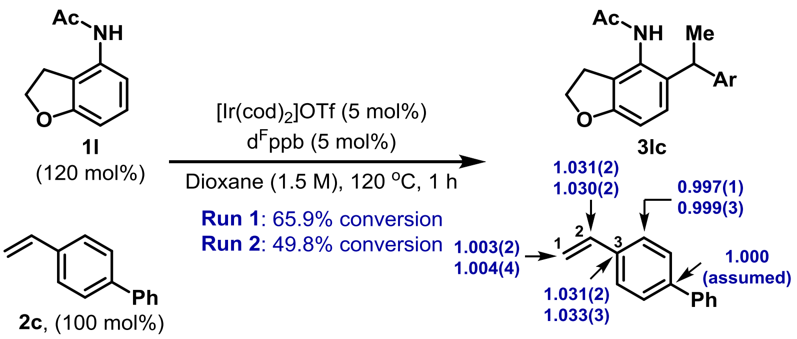

Interpretation of data: Previously reported deuterium labelling studies support reversible C-H oxidative addition and alkene hydrometallation in advance of the C-C bond forming event (see Scheme 1B in the main paper).[8] This is presumably the first irreversible step of the cycle and might proceed via either C-C reductive elimination (only C2 involved in the first irreversible step; **I** to **II** to **3**) or alkene carbometallation (C1 and C2 involved in the first irreversible step; **I** to **III** to **3**). In parallel experiments,

the hydroarylation reaction between equimolar quantities of styrene **2c** and acetanilide **1l** was run to 49.8% and 65.9% conversion, and analysis of **2c** recovered from each run revealed KIEs at C2 of 1.030 and 1.031, respectively, with no significant ^{13}C enrichment at C1; interestingly, a significant KIE was also observed at C3. At the present stage, we have been unable to devise an acceptable computational model to account for these values; nevertheless, the absence of a significant KIE at C1 is suggestive of a C-C reductive elimination pathway for the formation of **3lc**.

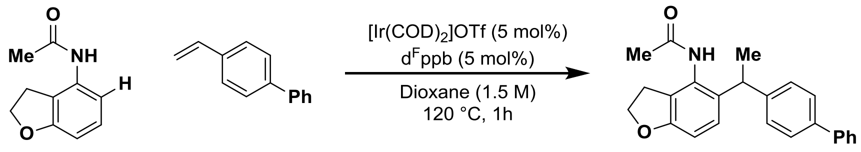

1l (120 mol%) **2c** (100 mol%) **3lc**

An oven-dried re-sealable tube, fitted with a magnetic stirrer, was charged with **1l** (2.40 mmol, 120 mol%), **2c** (2.00 mmol, 100 mol%), $[Ir(cod)_2]OTf$ (0.10 mmol, 5 mol%) and d^Fppb (0.10 mmol, 5 mol%). The tube was fitted with a rubber septum and purged with nitrogen. Anhydrous dioxane (1.3 mL) was added *via* syringe and the tube was sealed with a Young's tap. The reaction vessel was placed into a pre-heated heating block at 120 ^oC and stirred for 1 h. The reaction mixture was cooled to room temperature and concentrated *in vacuo*. The crude mixture was transferred into a 20 mL volumetric flask, which was previously charged with a known amount of internal standard (1,3,5-trimethoxybenzene, ~80 mg), and the flask was filled with $CDCl_3$, pre-treated over anhydrous K_2CO_3. Six aliquots of 0.4 mL each were taken from the solution and transferred into six NMR tubes, which were subsequently diluted with additional 0.3 mL of pre-treated $CDCl_3$ each. A ^1H NMR spectrum was recorded for each sample employing a 500 MHz instrument, using the following parameters: 16 scans, $\pi/2$ pulse, 6.5 s acquisition time and 40 s relaxation delay. The conversion of the alkene starting material (**F**) was determined by integration of the C**1**-$\underline{H}_{trans}$ signal of **2c** against the aromatic C-$\underline{H}$ signal of the internal standard. The remaining crude material was purified by FCC using toluene as the eluent to recover unreacted **2c**, and continued elution (toluene/EtOAc 10-30%) provided **3lc** as a colorless solid.

^1H NMR analysis provided a conversion of 65.9 ± 0.6% for the first experiment and of 49.8 ± 0.6% for the second experiment. Purification of the crude mixture by FCC (toluene) afforded 122 mg of unreacted **2c** (34% recovery) for the first sample and 162 mg (45% recovery) for the second sample.

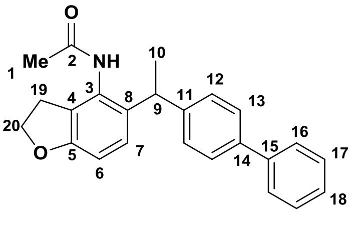

Data for *N*-(5-(1-([1,1'-biphenyl]-4-yl)ethyl)-2,3-dihydrobenzofuran-4-yl)acetamide (**3lc**) 0.9:0.1 mixture of rotamers *A*:*B*, >25:1 branched:linear): ν_{max} / cm^{-1}: 3218 (s), 3179 (m), 3028 (s), 2969 (m), 2895 (m), 1641 (s), 1521 (s), 1478 (s), 1289 (s); ^{1}H NMR ($CDCl_3$, 400 MHz): δ 7.62 – 7.47 (4H, m, C**13**-H, C**16**-H, *A*+*B*), 7.47 – 7.39 (2H, m, C**17**-H, *A*+*B*), 7.38 – 7.30 (1H, m, C**18**-H, *A*+*B*), 7.28 – 7.15 (3H, m, C**7**-H and C**12**-H, *A*+*B*), 6.85 (0.1H, d, *J* = 8.0 Hz, C**6**-H, *B*), 6.76 (0.9H, d, *J* = 8.0 Hz, C**6**-H, *A*), 6.60 (1H, br. s, N-H, *A*+*B*), 4.65 – 4.48 (2H, m, C**20**-H_2, *A*+*B*), 4.30 – 4.12 (1H, m, C**9**-H, *A*+*B*), 3.25 – 2.91 (2H, m, C**19**-H_2, *A*+*B*), 2.02 (3H, s, C**1**-H_3, *A*+*B*), 1.61 (3H, d, *J* = 7.0 Hz, C**10**-H_3, *A*+*B*); *Signals for rotamer A:* ^{13}C NMR ($CDCl_3$, 100 MHz): δ 167.5 (C**2**), 159.7 (C**5**), 145.3 (C**11**), 140.6 (C**15**), 139.3 (C**14**), 131.7 (C**3**), 131.6 (C**8**), 128.8 (C**17**), 127.6 (*2 signals*, C**12** and C**13**), 127.3 (C**18**), 126.9 (*2 signals*, C**16** and C**7**), 125.8 (C**4**), 107.5 (C**6**), 71.5 (C**20**), 40.2 (C**9**), 29.4 (C**19**), 23.4 (C**1**), 22.0 (C**10**); HRMS: (ESI^+) Calculated for $C_{24}H_{24}NO_2$: 358.1802. Found $[M+H]^+$: 358.1798; m.p. = 173 - 174 ˚C (hexane/CH_2Cl_2).

Tables used for the determination of the ^{13}C-KIE values are reported on the next pages.

First experiment:

Conversion (F)							
**fid1**	**fid2**	**fid3**	**fid4**	**fid5**	**fid6**	**F**	**ΔF**
66.0	65.2	65.5	65.8	67.0	65.8	**65.9**	**0.6**

^{13}C NMR integration of alkene starting material (R_0)								
**ppm peaks**	**fid1**	**fid2**	**fid3**	**fid4**	**fid5**	**fid6**	**R_0**	**ΔR_0**
**113.9 (C1)**	888.1	889.4	888.8	889.8	891.5	889.8	**889.6**	**1.2**
**136.5 (C2)**	1088.4	1087.5	1086.9	1086.7	1088.9	1089.3	**1088.0**	**1.1**
**136.7 (C3)**	998.4	997.2	997.3	997.5	1000.0	998.9	**998.2**	**1.1**
**126.8 (C4)**	1967.3	1966.3	1965.6	1967.5	1969.3	1967.4	**1967.2**	**1.3**
**140.6 (C6)**	1000.0	1000.0	1000.0	1000.0	1000.0	1000.0	**1000.0**	**0.0**

^{13}C NMR integration of alkene from 65.9 ± 0.6% conversion reaction (R)								
ppm peaks	fid1	fid2	fid3	fid4	fid5	fid6	R	ΔR
113.9 (C1)	890.0	894.1	893.9	891.5	891.5	893.1	**892.3**	**1.6**
136.5 (C2)	1126.8	1124.3	1123.2	1122.5	1123.1	1124.9	**1124.1**	**1.6**
136.7 (C3)	1034.7	1032.8	1030.8	1029.6	1029.1	1028.9	**1031.0**	**2.3**
126.8 (C4)	1955.6	1959.2	1962.0	1960.9	1961.1	1962.2	**1960.1**	**2.4**
140.6 (C6)	1000.0	1000.0	1000.0	1000.0	1000.0	1000.0	**1000.0**	**0.0**

Determination of ^{13}C KIEs						
ppm peaks	R/R_0	$\Delta(R/R_0)$	ΔKIE_F	ΔKIE_R	KIE	ΔKIE
113.9 (C1)	1.003136	0.002243	-0.000049	0.002092	1.002920	0.002092
136.5 (C2)	1.033263	0.001766	-0.000542	0.001691	1.031383	0.001776
136.7 (C3)	1.032812	0.002598	-0.000534	0.002486	1.030951	0.002543
126.8 (C4)	0.996395	0.001399	0.000056	0.001297	0.996653	0.001298
140.6 (C6)	1	0	0	0	**1**	**0**

Output		
C	KIE	
C1	**1.003 ± 0.002**	
C2	**1.031 ± 0.002**	
C3	**1.031 ± 0.003**	
C4	**0.997 ± 0.001**	

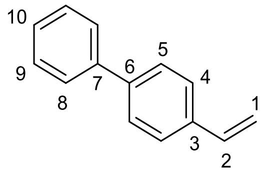

Second experiment:

Conversion (F)							
fid1	fid2	fid3	fid4	fid5	fid6	F	ΔF
50.6	48.8	49.6	49.9	50.1	49.9	**49.8**	**0.6**

^{13}C NMR integration of alkene starting material (R_0)								
ppm peaks	fid1	fid2	fid3	fid4	fid5	fid6	R_0	ΔR_0
113.9 (C1)	909.1	906.6	906.0	905.4	905.9	907.3	**906.7**	**1.3**
136.4 (C2)	1066.5	1066.3	1065.7	1065.3	1065.4	1068.1	**1066.2**	**1.0**
136.6 (C3)	1002.8	1000.9	999.7	998.7	1000.0	1001.9	**1000.7**	**1.5**
126.7 (C4)	1995.1	1993.2	1992.1	1990.5	1990.8	1996.1	**1993.0**	**2.2**

**140.6 (C6)**	1000.0	1000.0	1000.0	1000.0	1000.0	1000.0	**1000.0**	**0.0**

^{13}C NMR integration of alkene from 49.8 ± 0.6% conversion reaction (R)								
**ppm peaks**	**fid1**	**fid2**	**fid3**	**fid4**	**fid5**	**fid6**	**R**	**ΔR**
**113.9 (C1)**	907.0	906.0	910.9	910.4	910.4	910.8	**909.3**	**2.2**
**136.4 (C2)**	1088.1	1087.9	1089.8	1088.8	1086.8	1088.0	**1088.2**	**1.0**
**136.6 (C3)**	1021.4	1022.0	1024.5	1024.0	1022.8	1023.9	**1023.1**	**1.3**
**126.7 (C4)**	1986.2	1987.9	1996.7	1994.2	1989.6	1991.8	**1991.1**	**4.0**
**140.6 (C6)**	1000.0	1000.0	1000.0	1000.0	1000.0	1000.0	**1000.0**	**0.0**

Determination of ^{13}C KIEs						
**ppm peaks**	**R/R_0**	**$\Delta(R/R_0)$**	**ΔKIE_F**	**ΔKIE_R**	**KIE**	**ΔKIE**
**113.9 (C1)**	1.002811	0.002793	-0.000071	0.004073	**1.004087**	**0.004074**
**136.4 (C2)**	1.020632	0.001367	-0.000544	0.002062	**1.030523**	**0.002133**
**136.6 (C3)**	1.022408	0.001985	-0.000593	0.003007	**1.033208**	**0.003065**
**126.7 (C4)**	0.999047	0.002290	0.000024	0.003315	**0.998620**	**0.003315**
**140.6 (C6)**	1	0	0	0	**1**	**0**

Output		
**C**	**KIE**	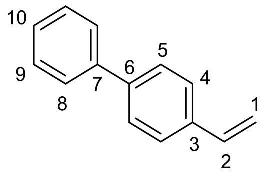
C1	**1.004 ± 0.004**	
C2	**1.030 ± 0.002**	
C3	**1.033 ± 0.003**	
C4	**0.999 ± 0.003**	

Ligand Synthesis

1,1'-Bis(dichlorophosphino)ferrocene

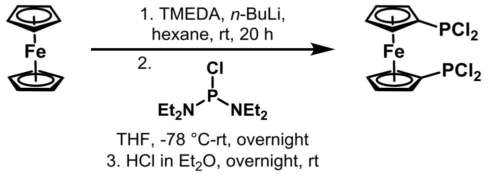

The title compound was prepared following a literature procedure.[4] Ferrocene (1.86 g, 10.0 mmol) was added to a round-bottomed Schlenk flask, purged with nitrogen and dissolved in dry and deoxygenated hexane (47 mL). Distilled tetramethylethylenediamine (3.15 mL, 21.0 mmol) was added dropwise to

the stirred solution over 10 minutes at ambient temperature, followed by *n*-BuLi (1.60 M in hexanes, 13.8 mL, 22.0 mmol). The solution was stirred for 22 h, before the resulting suspension was cooled to -78 °C in an acetone/dry ice bath. *N*,*N*-Bis(diethylamino)chlorophosphine (4.40 mL, 21.0 mmol) dissolved in dry and deoxygenated THF (14 mL) was added dropwise to the stirred suspension. Once the addition had finished the mixture was warmed to ambient temperature and stirred for 4 days. The reaction mixture was then cooled to -78 °C in an acetone/dry ice bath and treated with a solution of HCl in diethyl ether (2.00 M, 80.0 mL, 160 mmol). The solution was warmed to ambient temperature and stirred overnight. The resulting salts were filtered off through an oven-dried sinter funnel under a flow of nitrogen. The salts were washed with dry hexane (7 × 10 mL) and the filtrate was collected and concentrated in *vacuo,* affording the product (3.28 g, 85%) as an orange powder. *Note: an inert atmosphere was maintained at all times and the compound was stored in a glovebox.* ^{1}H NMR (400 MHz, $CDCl_3$): δ 4.75-4.62 (8H, m); ^{31}P NMR (162 MHz, $CDCl_3$): δ 163.3. *The title compound was used without further purification. The spectroscopic properties for this compound were consistent with the data available in the literature.*[4]

<u>General Procedure A</u> for the preparation of ligands; L-2 – L-5:

An oven-dried multi-neck flask fitted with a condenser was charged with Mg turnings (500 mol%) and purged with nitrogen. One iodine bead was added and the solids were suspended in dry Et_2O (4.50 mL/mmol of Mg). To activate the magnesium, the solution was heated to reflux with a heatgun. Once cooled to ambient temperature, the fluorinated aryl bromide (600 mol%) was added *via* syringe and the solution was heated at reflux for 1 h. The solution was cooled to ambient temperature, before 1,1'-bis(dichlorophosphino)ferrocene (100 mol%) dissolved in dry Et_2O (6.00 mL/mmol of 1,1'-bis(dichlorophosphino)ferrocene) was added dropwise. The solution was stirred overnight. Unreacted Grignard reagent was quenched by the addition of water (15 mL/mmol). The resulting solution was extracted with CH_2Cl_2 (3 × 25 mL/ mmol). The organic extracts were combined, dried over Na_2SO_4 and concentrated in *vacuo*. The resulting oil was dissolved in CH_2Cl_2 and filtered through Celite® to afford the crude product as an orange oil. Purification by FCC (hexane/EtOAc 0-5%) followed by recrystallization with cyclohexane afforded the pure products.

*Ligands **L-1** and **L-6** were purchased from commercial sources (Aldrich, Strem) and used without any further purification.*

L-2

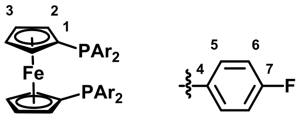

General Procedure A: The reaction was carried out with 1-bromo-4-fluorobenzene (1.17 mL, 10.6 mmol) to afford the title compound (157 mg, 14%) as an orange powder. ν_{max} / cm^{-1}: 3075 (m), 1587 (s), 1493 (m), 1224 (s), 1158 (s); ^{1}H NMR (400 MHz, CDCl$_3$): δ 7.34 – 7.23 (8H, m, C**6**-H), 7.09 – 6.99 (8H, m, C**5**-H), 4.35 (4H, app. t, J = 2.0 Hz, C**3**-H), 3.97 (4H, app. q, J = 2.0 Hz, C**2**-H); ^{13}C NMR (126 MHz, CDCl$_3$): δ 163.4 (d, $^{1}J_{C-F}$ = 249.0 Hz, C**7**), 135.3 (dd, $^{2}J_{C-F}$ = 21.0, $^{3}J_{C-P}$ = 8.0 Hz, C**6**), 134.2 (dd, $^{1}J_{C-P}$ = 10.0, $^{4}J_{C-F}$ = 3.5 Hz, C**4**), 115.5 (dd, $^{3}J_{C-F}$ = 21.0, $^{2}J_{C-P}$ = 8.0 Hz, C**5**), 76.8 (C**1**), 73.7 (d, $^{2}J_{C-P}$ = 15.0 Hz, C**2**), 72.7 (dd, $^{3}J_{C-P}$ = 3.5, $^{4}J_{C-P}$ = 1.5 Hz, C**3**); ^{19}F NMR (377 MHz, CDCl$_3$): δ -112.3 (tq, J = 9.5, 5.5 Hz); ^{31}P NMR (162 MHz, CDCl$_3$): δ -19.3; HRMS: (MALDI) calculated for $C_{34}H_{24}F_4FeP_2$: 626.0634. Found [M]: 626.0628; m.p. 140 - 141 °C (cyclohexane).

L-3

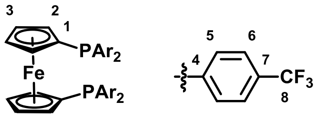

General Procedure A: The reaction was carried out with 4-bromobenzotrifluoride (1.48 mL, 10.6 mmol) to afford the title compound (179 mg, 12%) as an orange powder. ν_{max} / cm^{-1}: 2926 (m), 1606 (s), 1319 (s), 1121 (s), 1059 (s); ^{1}H NMR (500 MHz, CDCl$_3$): δ 7.55 (8H, d, J = 7.5 Hz, C**6**-H), 7.38 (8H, app. t, J = 7.5 Hz, C**5**-H), 4.33 (4H, app. t, J = 2.0 Hz, C**3**-H), 3.99 (4H, app. q, J = 2.0 Hz, C**2**-H); ^{13}C NMR (126 MHz, CDCl$_3$): δ 143.0 (d, $^{1}J_{C-P}$ = 13.0 Hz, C**4**), 133.8 (d, $^{2}J_{C-P}$ = 20.0 Hz, C**5**), 131.1 (q, $^{2}J_{C-F}$ = 32.5 Hz, C**7**), 125.2 (dd, $^{3}J_{C-F}$ = 7.0, $^{4}J_{C-F}$ = 3.5 Hz, C**6**), 125.1 (q, $^{1}J_{C-F}$ = 273 Hz, C**8**), 75.0 (d, $^{1}J_{C-P}$ = 7.5 Hz, C**1**), 74.0 (d, $^{2}J_{C-P}$ = 15.0 Hz, C**2**), 73.0 – 72.9 (m, C**3**); ^{19}F NMR (377 MHz, CDCl$_3$): δ -62.7, ^{31}P NMR (162 MHz, CDCl$_3$): δ -16.6; HRMS: (MALDI) calculated for $C_{38}H_{24}F_{12}FeP_2$: 826.0506. Found [M]: 826.0499; m.p. 151 - 153 °C (cyclohexane).

L-4

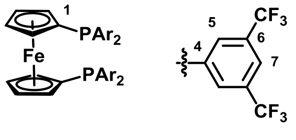

General Procedure A: The reaction was carried out with (1,3)-bis(trifluoromethyl)-5-bromobenzene (1.42 mL, 8.22 mmol) to afford the title compound (632 mg, 42%) as an orange powder. ν_{max} / cm^{-1}: 2923 (m), 2858 (m), 1352 (s), 1273 (s), 1095 (s); ^{1}H NMR (500 MHz, CDCl$_3$): δ 7.89 (4H, s, C**7**-H), 7.69 (8H, app. d, J = 6.5 Hz, C**5**-H), 4.44 (4H, app. t, J = 2.0 Hz, C**3**-H), 4.00 (4H, app. q, J = 2.0 Hz,

C**2**-H); ^{13}C NMR (126 MHz, $CDCl_3$): δ 140.3 (d, $^{1}J_{C-P}$ = 17.5 Hz, C**4**), 132.9 (dd, $^{2}J_{C-P}$ = 21.0, $^{3}J_{C-F}$ = 3.5 Hz, C**5**), 132.1 (qd, $^{2}J_{C-F}$ = 33.5, $^{3}J_{C-P}$ = 6.5 Hz, C**6**), 123.4 (q, $^{3}J_{C-F}$ = 3.5 Hz, C**7**), 122.9 (q, $^{1}J_{C-F}$ = 278.0 Hz, C**8**) 73.8 (d, $^{1}J_{C-P}$ = 7.0 Hz, C**1**), 73.6 (d, $^{2}J_{C-P}$ = 15.5 Hz, C**2**), 73.3 – 73.2 (m, C**3**); ^{19}F NMR (377 MHz, $CDCl_3$): δ -62.9; ^{31}P NMR (162 MHz, $CDCl_3$): δ -14.9; HRMS: (MALDI) calculated for $C_{42}H_{20}F_{24}FeP_2$: 1098.0002. Found [M]: 1098.0014; m.p. 145 - 147 °C (cyclohexane).

L-5

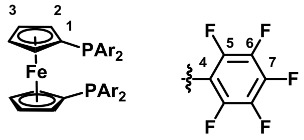

General Procedure A: The reaction was carried out with bromopentafluorobenzene (1.32 mL, 10.6 mmol) to afford the title compound (40.8 mg, 3%) as an orange powder. ν_{max} / cm^{-1}: 2929 (m), 1638 (m), 1514 (s), 1469 (s), 1086 (s); ^{1}H NMR (500 MHz, $CDCl_3$): δ 4.42 (4H, app. t, J = 2.0 Hz, C**3**-H), 4.29 (4H, app. q, J = 2.0 Hz, C**2**-H); ^{13}C NMR (126 MHz, $CDCl_3$): δ 147.5 (d, $^{1}J_{C-F}$ = 242.0 Hz, C**6**), 142.7 (d, $^{1}J_{C-F}$ = 257.5 Hz, C**7**), 137.8 (d, $^{1}J_{C-F}$ = 256.0 Hz, C**5**), 109.0 (dt, $^{1}J_{C-P}$ = 34.5, $^{2}J_{C-F}$ = 20.5 Hz, C**4**), 74.8 (d, $^{2}J_{C-P}$ = 20.5 Hz, C**2**), 73.2 (C**3**), 68.9 (C**1**); ^{19}F NMR (377 MHz, $CDCl_3$): δ -129.2 (–) -129.5 (8F, m, F_{meta}), -149.0 – -149.2 (4F, m, F_{para}), -159.5 (–) -159.8 (8F, m, F_{ortho}); ^{31}P NMR (162 MHz, $CDCl_3$): δ -58.4; HRMS: (MALDI) calculated for $C_{34}H_8F_{20}FeP_2$: 913.9126. Found [M]: 913.9120; m.p. 194 - 196 °C (cyclohexane).

Substrate Synthesis

*Substrates **1a, 1b, 1j, 1o, 1q** and **1u** were purchased from commercial sources (Aldrich, Alfa-Aesar) and used without any further purification.*

*Substrates **1c**[5] (quantitative) and **1d**[5] (quantitative), **1f**[6] (89% yield), **1g**[6] (85% yield), **1i**[7] (78% yield), **1l**[8] (58% yield) and **1n**[8] (83% yield) were synthesized following a literature procedure. The spectroscopic properties of these compounds are consistent with the data available in the literature: **1c**[5], **1d**[9], **1f**[10], **1g**[11], **1i**[12], **1**[8], **1n**[8].*

*Substrates **1k** (quantitative), **1m** (quantitative) and **1p** (quantitative) were synthesized following **General Procedure B**. The spectroscopic properties of these compounds were consistent with the data available in the literature: **1k**[13], **1m**[14], **1p**[15].*

*Substrate **1r** was synthesised following **General Procedure D.** The spectroscopic properties of this compound were consistent with the literature.*[16]

*Styrenes **2a-2i** were purchased from commercial sources (Aldrich, Strem).*

General Procedure B for the preparation of acetanilide substrates 1k, 1m, 1p:

To an ice cooled (0 °C) solution of aniline (100 mol%) and pyridine (60 mol%) in CH_2Cl_2 (0.7 M) was added dropwise acetic anhydride (110 mol%). The reaction was warmed to ambient temperature and stirred until consumption of the starting material (monitored by TLC). Water (2 mL/mmol) was added and the mixture was extracted with CH_2Cl_2 (3 × 5 mL/mmol). The organic extracts were combined, washed with saturated aq. $NaHCO_3$ (3 × 2 mL/mmol) and brine (2 mL/mmol), dried over Na_2SO_4 and concentrated *in vacuo* to provide the crude product. Purification by FCC afforded pure acetanilide.

General Procedure C for the preperation of acetanilide substrates 1e and 1h:

To an oven-dried flask was added EDCI (110 mol%) and DMAP (1 mol%) under nitrogen. CH_2Cl_2 (0.2 M), aniline (100 mol%) and the relative carboxylic acid (105 mol%) were added. The resulting solution was stirred at ambient temperature until full consumption of aniline was observed by TLC. The reaction mixture was washed with aqueous saturated sodium bicarbonate solution before being dried over Na_2SO_4 and concentrated *in vacuo*. Purification by FCC afforded pure acetanilide.

General Procedure D for the preparation of acetanilides 1r, 1s, 1t and 1v:

To a stirred solution of the corresponding aniline (5.00 mmol) and triethylamine (0.732 mL, 5.25 mmol) in dry EtOAc (8 mL) at 0 °C was added cyclopentanecarbonyl chloride (0.638 mL, 5.25 mmol) dropwise over 5 minutes. The solution was then allowed to warm to ambient temperature and stirred until completion (as monitored by TLC). The resulting slurry was taken up in EtOAc (20 mL) and washed with water (20 mL) and brine (20 mL), before being dried over Na_2SO_4 and concentrated in *vacuo*. Purification of the residues by recrystallisation (hexane/EtOAc) afforded the title compounds.

***N*-Phenylcyclobutanecarboxamide (1e)**

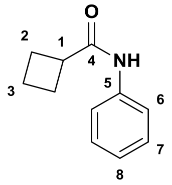

General Procedure C*:* Purification of the residue by FCC (hexane/EtOAc 60%) afforded the title compound (602 mg, 68%) as a colorless solid. ν_{max} / cm^{-1} : 3249 (m), 2943 (m), 1655 (s), 1443 (s); ^{1}H NMR (400 MHz, $CDCl_3$): δ 7.53 (2H, d, *J* = 8.0 Hz, C**6**-H), 7.31 (2H, dd, *J* = 8.0, 8.0 Hz, C**7**-H), 7.13 – 7.04 (2H, m, C**8**-H, N-H), 3.16 (1H, app. p, *J* = 8.5 Hz, C**1**-H), 2.48 – 2.32 (2H, m, C**2**-H_2), 2.29 – 2.18 (2H, m, C**2**-H_2), 2.08 – 1.82 (2H, m, C**3**-H_2); ^{13}C NMR (101 MHz, $CDCl_3$): δ 173.4 (C**4**), 138.2 (C**5**), 129.1 (C**7**), 124.1 (C**8**), 119.8 (C**6**), 41.0 (C**1**), 25.4 (C**2**), 18.2 (C**3**); HRMS: (ESI^{+}) calculated for $C_{11}H_{13}NONa$ 198.0889. Found $[M+Na]^{+}$ 198.0892; m.p. 103 - 106 °C ($CDCl_3$).

3-Methyl-*N*-phenylbutanamide (1h)

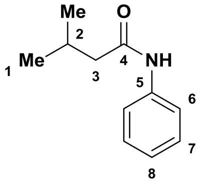

General Procedure C*:* Purification of the residue by FCC (hexane/EtOAc 60%) afforded the title compound (731 mg, 78%) as a colorless solid. ν_{max} / cm^{-1} : 3244 (m), 2964 (m), 1654 (s), 1597 (s); ^{1}H NMR (400 MHz, $CDCl_3$): δ 7.52 (2H, d, *J* = 8.0 Hz, C**6**-H), 7.31 (2H, dd, *J* = 8.0, 8.0 Hz, C**7**-H), 7.24 (1H, s, N-H), 7.10 (1H, t, *J* = 8.0 Hz, C**8**-H), 2.25 – 2.18 (3H, m, C**3**-H_2, C**2**-H), 1.02 (6H, d, *J* = 6.0 Hz, C**1**-H_3); ^{13}C NMR (101 MHz, $CDCl_3$): δ 171.0 (C**4**), 138.1 (C**5**), 129.1 (C**7**), 124.3 (C**8**), 120.0 (C**6**), 47.3 (C**2**), 26.4 (C**3**), 22.6 (C**1**); HRMS: (ESI^{+}) calculated for $C_{11}H_{15}NONa$ 200.1046. Found $[M+Na]^{+}$ 200.1049; m.p. 106 - 109 °C ($CDCl_3$).

N-(3-Isopropylphenyl)cyclopentanecarboxamide (1s)

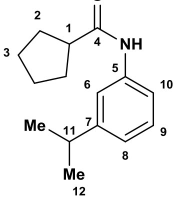

General Procedure D: Purification of the residue by recrystallization (hexane/EtOAc) afforded the title compound (1.16 g, quantitative) as colourless needles. V_{max}/ Cm^{-1} : 3295 (m), 2958 (m), 2858 (m), 1656 (s), 1510 (s); ^{1}H NMR (400 MHz, $CDCl_3$): δ 7.48 – 7.42 (1H, m, C**8**-H), 7.41 (1H, s, N-H), 7.36 – 7.29 (1H, m, C**10**-H), 7.28 – 7.17 (1H, m, C**9**-H), 6.95 (1H, d, *J* = 7.5 Hz, C**6**-H), 2.86 (1H, hept, *J* = 6.5 Hz, C**11**-H), 2.67 (1H, p, *J* = 8.0 Hz, C**1**-H), 1.98 – 1.49 (8H, m, C**2**-H_2, C**3**-H_2), 1.23 (6H, d, *J* = 6.5 Hz, C**12**-H_3); ^{13}C NMR (101 MHz, $CDCl_3$): δ 174.8 (C**4**), 150.0 (C**7**), 138.3 (C**5**), 128.9 (C**9**), 122.3 (C**6**), 118.0 (C**8**), 117.3 (C**10**), 47.0 (C**1**), 34.3 (C**11**), 30.7 (Cyclopentyl), 26.2 (Cyclopentyl), 24.0 (C**12**); HRMS: (ESI^+) calculated for $C_{15}H_{21}NONa$ 254.1515. Found $[M+Na]^+$ 254.1514; m.p. = 52 - 54 °C (hexane/EtOAc).

***N*-([1,1'-Biphenyl]-3-yl)cyclopentanecarboxamide (1t)**

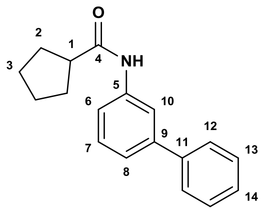

General Procedure D: Purification of the residue by recrystallization (hexane/EtOAc) afforded the title compound (1.14 g, 88%) as a colourless powder. V_{max}/ Cm^{-1} : 3296 (m), 2951 (m), 1658 (s), 1550 (s); ^{1}H NMR (400 MHz, $CDCl_3$): δ 7.83 (1H, s, C**10**-H), 7.61 – 7.56 (2H, m, C**12**-H), 7.48 (1H, dd, *J* = 8.0, 2.0 Hz, ArCH), 7.45 – 7.39 (2H, m, C**13**-H), 7.38 – 7.31 (2H, m, C**7**-H, ArCH), 7.27 (1H, s, N-H), 2.71 (1H, p, *J* = 8.0 Hz, C**1**-H), 2.02 – 1.87 (4H, m, Cyclopentyl), 1.86 – 1.71 (2H, m, Cyclopentyl), 1.69 – 1.58 (2H, m, Cyclopentyl); ^{13}C NMR (101 MHz, $CDCl_3$): δ 174.8 (C**4**), 142.3 (C**9**), 140.8 (C**11**), 138.7 (C**5**), 129.5 (ArCH), 128.9 (C**13**), 127.6 (ArCH), 127.3 (C**12**), 123.0 (ArCH), 118.6 (ArCH), 47.1 (C**1**), 30.7 (Cyclopentyl), 26.2 (Cyclopentyl); HRMS: (ESI^+) calculated for $C_{18}H_{20}NO$ 266.1539. Found $[M+H]^+$ 266.1541; m.p. = 110 - 112 °C (hexane/EtOAc).

***N*-(*o*-Tolyl)cyclopentanecarboxamide (1v)**

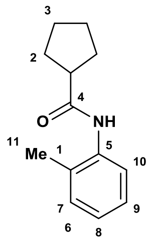

General Procedure D: Purification of the residue by recrystallization (hexane/EtOAc) afforded the title compound (742 mg, 74%) as a colourless powder. V_{max}/ Cm^{-1} : 3372 (m), 2956 (m), 2864 (m), 1650 (s), 1532 (s); ^{1}H NMR (400 MHz, $CDCl_3$): δ 7.86 – 7.80 (1H, m, C**10**-H), 7.21 – 7.13 (2H, m, C**7**-H, C**9**-H), 7.09 – 7.00 (1H, m, C**8**-H), 6.98 (1H, s, N-H), 2.72 (1H, p, *J* = 8.0 Hz, C**1**-H), 2.24 (3H, s, C**11**-H_3), 1.99 – 1.54 (8H, m, C**2**-H_2, C**3**-H_2); ^{13}C NMR (101 MHz, $CDCl_3$): δ 174.6 (C**4**), 136.0 (C**5**), 130.5 (C**7**), 128.8 (C**6**), 126.9 (C**9**), 125.0 (C**8**), 123.1 (C**10**), 46.9 (C**1**), 30.7 (Cyclopentyl), 26.1 (Cyclopentyl), 17.9 (C**11**); HRMS: (ESI^+) calculated for $C_{13}H_{18}NO$ 204.1383. Found $[M+H]^+$ 204.1381; m.p. = 130 - 132 °C (hexane/EtOAc).

(13*S*)-13-Methyl-17-oxo-7,8,9,11,12,13,14,15,16,17-decahydro-6*H*-cyclopenta[*a*]phenanthren-3-yl trifluoromethanesulfonate

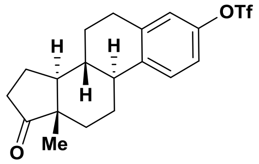

The title compound was prepared following a literature procedure.[17] To a flame-dried round-bottomed flask was added estrone (2.00 g, 7.40 mmol) in dry CH_2Cl_2 (37 mL) under N_2. Pyridine (1.20 mL, 14.8 mmol) was added and the solution was cooled to 0 °C. Triflic anhydride (1.50 mL, 8.88 mmol) was added dropwise over 5 minutes. The solution was warmed to ambient temperature and was stirred for 1.5 h. The reaction was quenched by the addition of water (20 mL). The layers were separated, and the aqueous phase was extracted with CH_2Cl_2 (3 × 30 mL). The organic extracts were combined, washed with brine (30 mL), dried over Na_2SO_4 and concentrated in *vacuo*. The residue was purified by FCC (hexane/EtOAc 30%) to afford the title compound (3.04 g, quantitative) as an orange solid. ^{1}H NMR (400 MHz, $CDCl_3$): δ 7.33 (1H, d, *J* = 8.5 Hz), 7.06 – 6.95 (2H, m), 2.98 – 2.93 (2H, m), 2.61 – 2.46 (1H, m), 2.44 – 2.35 (1H, m), 2.35 – 2.25 (1H, m), 2.22 – 2.00 (3H, m), 2.00 – 1.92 (1H, m), 1.69 – 1.38 (6H, m), 0.91 (3H, s); ^{13}C NMR (101 MHz, $CDCl_3$): δ 220.4, 147.7, 140.4, 139.4, 127.3, 121.3, 118.8 (q, *J* = 321.0 Hz), 118.4, 50.5, 47.9, 44.2, 37.8, 35.9, 31.6, 29.5, 26.2, 25.8, 21.7, 13.9; m.p. 85 - 87 °C (CH_2Cl_2) (Lit.[16] 87-89 °C. *no recrystallization solvent specified*); $[\alpha]^{22}_D$ = + 114.6 (c = 0.20, CH_2Cl_2). *The spectroscopic properties for this compound were consistent with the data available in the literature.*[18]

***N*-((8*R*,9*S*,13*S*,14*S*)-13-Methyl-17-oxo-7,8,9,11,12,13,14,15,16,17-decahydro-6*H*-cyclopenta[*a*]phenanthren-3-yl)acetamide (1w)**

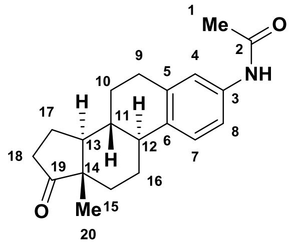

The title compound was prepared following a literature procedure.[19] An oven dried resealable tube was charged with (13*S*)-13-methyl-17-oxo-7,8,9,11,12,13,14,15,16,17-decahydro-6*H*-cyclopenta[*a*] phenanthrene-3-yl trifluoromethanesulfonate (805 mg, 2.00 mmol), the preceding acetanilide (177 mg, 3.00 mmol), $Pd_2(dba)_3$ (18.0 mg, 0.020 mmol), Me_4*t*-BuXPhos (48.0 mg, 0.100 mmol) and K_3PO_4 (1.06 g, 5.00 mmol). The tube was fitted with a rubber septum and purged with nitrogen before *t*-BuOH (deoxygenated with Ar for 10 minutes) was added and the tube was sealed with a Young's tap. The reaction mixture was heated at 110 °C for 18 h, cooled to ambient temperature and filtered over a pad of Celite®, washing with EtOAc and DCM/MeOH (1:1). The resulting solution was concentrated in *vacuo* and the crude product was purified by FCC (toluene/EtOAC 40-50%) to afford the desired product (526 mg, 84%) as a colorless solid. ν_{max} / cm^{-1}: 3291 (m), 2936 (m), 2920 (m), 1734 (s), 1658 (s); ^1H NMR (400 MHz, DMSO-d_6): δ 9.74 (1H, s, N-H), 7.33-7.25 (2H, m, C**4**-H, C**8**-H), 7.17 (1H, d, *J* = 8.5 Hz, C**7**-H), 2.85-2.76 (2H, m, C**9**-H_2), 2.44 (1H, dd, *J* = 19.0, 8.5 Hz, C**18**-H_2), 2.38-2.28 (1H, m, C**16**-H_2), 2.24-2.14 (1H, m, C**12**-H), 2.05 (1H, dd, *J* = 19.0, 8.5 Hz, C**18**-H_2), 2.00 (3H, s, C**1**-H_3), 1.98-1.88 (2H, m, C**10**-H_2, C**17**-H_2), 1.78-1.70 (1H, m, C**15**-H_2), 1.61-1.28 (6H, m, C**10**-H_2, C**11**-H, C**13**-H, C**15**-H_2, C**16**-H_2, C**17**-H_2), 0.83 (3H, s, C**20**-H_3); ^{13}C NMR (101 MHz, DMSO-d_6): δ 219.6 (C**19**), 167.9 (C**2**), 136.9 (C**5**), 136.3 (C**3**), 134.3 (C**6**), 125.4 (C**7**), 119.1 (C**4**), 116.6 (C**8**), 49.6 (C**13**), 47.3 (C**14**), 43.6 (C**12**), 37.7 (C**11**), 35.3 (C**18**), 31.3 (C**15**), 29.1 (C**9**), 26.0 (C**10**), 25.3 (C**16**), 23.9 (C**1**), 21.1 (C**17**), 13.5 (C**20**); HRMS (ESI$^+$) calculated for $C_{20}H_{26}NO_2$ 312.1958. Found $[M+H]^+$ 312.1979; m.p. 230 °C (degradation) ($CDCl_3$); $[\alpha]^{20}_D$ = + 134.7 (c = 0.20, CH_2Cl_2).

Trimethyl(4-vinylphenyl)silane (2g)

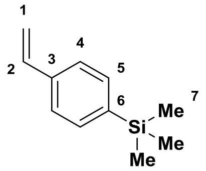

The title compound was prepared following a literature procedure.[20] To an oven-dried Schlenk tube was added magnesium turnings (265 mg, 10.9 mmol), THF (11 mL), chlorotrimethylsilane (0.693 mL, 5.46 mmol) and 4-bromostyrene (0.714 mL, 5.46 mmol) under nitrogen. The Schlenk tube was placed in a commercial ultrasonic cleaning bath (Ultrawave Ltd. SFE 510/1, 220-240 KHz, 275 W) and sonicated for 3 h. The mixture was washed with aqueous saturated sodium chloride solution (10 mL) and extracted

with Et_2O (3 × 20 mL). The organic extracts were combined, dried over Na_2SO_4 and concentrated in *vacuo*. Purification of the residue by FCC (hexane/Et_2O 0-5%) afforded the title compound (673 mg, 70%) as a colorless oil. ν_{max} / cm^{-1}: 3063 (m), 2956 (m), 1629 (m), 824 (s); ^1H NMR (400 MHz, $CDCl_3$): δ 7.51 (2H, d, *J* = 8.0 Hz, C**4**-H), 7.42 (2H, d, *J* = 8.0 Hz, C**5**-H), 6.74 (1H, dd, *J* = 17.5, 11.0 Hz, C**2**-H), 5.80 (1H, dd, *J* = 17.5, 1.0 Hz, C**1**-H), 5.27 (1H, dd, *J* = 11.0, 1.0 Hz, C**1**-H), 0.29 (9H, s, C**7**-H_3); ^{13}C NMR (101 MHz, $CDCl_3$): δ 140.3 (C**6**), 138.1 (C**3**), 137.0 (C**2**), 133.7 (C**4**), 125.7 (C**5**), 114.2 (C**1**), -0.9 (C**7**). *A mass could not be observed by ESI or MALDI.*

5-Vinylbenzo[*b*]thiophene (2k)

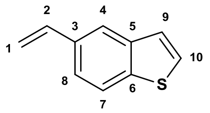

The title compound was prepared following a literature procedure.[21] A resealable tube was charged with 5-bromo-1-benzothiophene (512 mg, 2.40 mmol), vinyl boronic acid pinacol ester (0.448 mL, 2.64 mmol), $Pd(OAc)_2$ (21.6 mg, 0.096 mmol), SPhos (78.8 mg, 0.192 mmol) and K_3PO_4 (1.53 g, 7.21 mmol). The tube was purged with nitrogen before the addition of 1,4-dioxane (9.6 mL) and water (0.216 mL). The reaction tube was sealed and heated to 80 °C for 2 h. The resulting solution was filtered through celite® with EtOAc before being washed with H_2O (20 mL) and brine (20 mL). The organic extracts were combined, dried over Na_2SO_4, filtered and concentrated in *vacuo*. The resulting residue was purified by FCC (hexane) to afford the title compound (350 mg, 90%) as a colourless solid. ν_{max} / Cm^{-1} : 2959 (m), 2921 (m), 1627 (s), 1426 (s); ^1H NMR (500 MHz, $CDCl_3$): δ 7.82 (1H, dd, *J* = 8.5, 1.0 Hz, C**8**-H), 7.81 (1H, d, *J* = 1.0 Hz, C**4**-H), 7.46 (1H, dd, *J* = 8.5, 1.0 Hz, C7-H), 7.44 (1H, d, *J* = 5.5 Hz, C**9**-H), 7.32 (1H, dd, *J* = 5.5, 1.0 Hz, C**10**-H), 6.84 (1H, dd, *J* = 17.5, 11.0 Hz, C**2**-H), 5.81 (1H, dd, *J* = 17.5, 1.0 Hz, C**1**-H_2), 5.28 (1H, dd, *J* = 11.0, 1.0 Hz, C**1**-H_2); ^{13}C NMR (126 MHz, $CDCl_3$): δ 140.1 (C**5**), 139.3 (C**6**), 137.1 (C**2**), 134.1 (C**3**), 127.0 (C**9**), 124.1 (C**10**), 122.6 (ArCH), 122.4 (C**7**), 121.8 (ArCH), 113.6 (C**1**); m.p. 34 - 36 °C ($CDCl_3$); *A mass could not be observed by ESI or MALDI.*

Selected Reaction Optimization Tables:

Optimization of reaction with regard to the oxidant:

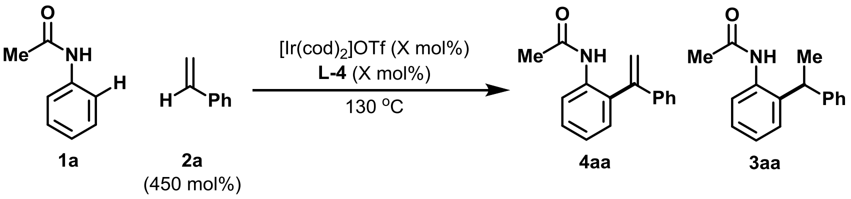

[a] Yields and selectivities were determined by 1H NMR analysis using 1,3,5-trimethoxybenzene as a standard.

Entry	Time	Solvent	X	Additive	Yield[a]	4aa:3aa
1	24 h	dioxane (1.5 M)	5.0 mol%	none	23%	10:2
2	24 h	dioxane (1.5 M)	5.0 mol%	pinacolone (1 equiv.)	49%	8:2
3	24 h	dioxane (1.5 M)	5.0 mol%	benzoquinone (1 equiv.)	0	0:0
4	24 h	dioxane (1.5 M)	5.0 mol%	benzophenone (1 equiv.)	37%	7.5:2.5
5	24 h	dioxane (1.5 M)	5.0 mol%	norbornene (1 equiv.)	28%	7.5:2.5
6	72 h	dioxane (0.5 M)	7.5 mol%	pinacolone (2 equiv.)	68%	7.5:2.5
7	72 h	dioxane (0.5 M)	7.5 mol%	*t*-butylethylene (2 equiv.)	74%	10:2

Reaction Scope

<u>General Procedure E</u> for branch-selective Heck-like reaction on acetanilide substrates:

An oven-dried re-sealable tube, fitted with a magnetic stirrer, was charged with substrate (0.143 mmol, 100 mol%), [Ir(cod)$_2$]OTf (7.5 - 10 mol%) and **L-4** (7.5 - 10 mol%). The tube was fitted with a rubber septum and purged with nitrogen. Styrene derivative (450 mol%) and *t*-butylethylene (200 mol%) in anhydrous 1,4-dioxane (0.5-1.0 M concentration with respect to substrate) were added and the tube was fitted with a Young's tap. The reaction mixture was then heated at 130 °C for 72-96 h, before being cooled to ambient temperature and concentrated in *vacuo.* Purification of the residues by FCC afforded the pure products. *Note that the alkenylation and hydroarylation products were easily separated by FCC. In some cases, a second column was performed to remove an impurity associated with degradation of the ligand.*

***N*-(2-(1-Phenylvinyl)phenyl)acetamide (4aa)**

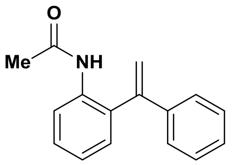

General Procedure E: The reaction was carried out with Ir(cod)$_2$OTf (7.5 mol%), **L-4** (7.5 mol%) and 1,4-dioxane (0.5 M with respect to substrate) and was run for 72 h. Purification of the residue by FCC (hexane/EtOAc 10-40%) afforded the title compound (25.0 mg, 74%) as a colorless solid. ^{1}H NMR (400 MHz, DMSO-d_6): δ 8.83 (1H, s), 7.49 (1H, d, *J* = 8.0 Hz), 7.39 – 7.26 (4H, m), 7.24 – 7.13 (4H, m), 5.76 (1H, d, *J* = 1.0 Hz), 5.31 (1H, d, *J* = 1.0 Hz), 1.60 (3H, s); ^{13}C NMR (101 MHz, DMSO-d_6): δ 167.9, 146.3, 140.0, 135.5, 130.2, 128.1, 127.9, 127.6, 126.5, 125.9, 125.0, 116.7, 22.7; m.p. 120 - 122 °C (hexane/EtOAc). *The spectroscopic properties for this compound were consistent with the data available in the literature.*[22]

***N*-(2-(1-Phenylvinyl)phenyl)propionamide (4ba)**

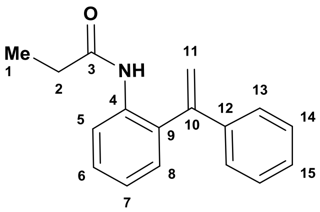

General Procedure E: The reaction was carried out with Ir(cod)$_2$OTf (7.5 mol%), **L-4** (7.5 mol%) and 1,4-dioxane (0.5 M with respect to substrate) and was run for 72 h. The crude material was purified by FCC (1st column: hexane/EtOAc 0-30%; 2nd column: hexane/EtOAc 30%) to afford the title compound (24.7 mg, 69%, 0.96:0.04 mixture of rotamers *A:B*) as golden plates. ν_{max} / cm^{-1}: 3415 (m), 3288 (m), 1668 (s), 1516 (s), 1448 (s); *Signals for rotamer A*: ^{1}H NMR (400 MHz, CDCl$_3$): δ 8.22 (1H, d, *J* = 8.0

Hz, C**5**-H), 7.38 (1H, ddd, *J* = 8.0, 8.0, 2.0 Hz, C**6**-H), 7.35 – 7.31 (5H, m, C**13**-H, C**14**-H, C**15**-H), 7.29 – 7.25 (1H, m, C**8**-H), 7.19 – 7.12 (1H, m, C**7**-H), 6.99 (1H, br. s, N-H), 5.88 (1H, d, *J* = 1.5 Hz, C**11**-H_2), 5.37 (1H, d, *J* = 1.5 Hz, C**11**-H_2), 2.01 (2H, q, *J* = 7.5 Hz, C**2**-H_2), 0.92 (3H, t, *J* = 7.5 Hz, C**1**-H_3); ^{13}C NMR (101 MHz, $CDCl_3$): δ 171.7 (C**3**), 146.5 (C**10**), 139.3 (C**12**), 135.4 (C**4**), 131.8 (C**9**), 130.4 (C**8**), 129.0 (ArCH), 128.9 (ArCH), 128.7 (C**6**), 126.6 (ArCH), 124.2 (C**7**), 121.7 (C**5**), 117.4 (C**11**), 30.8 (C**2**), 9.4 (C**1**); HRMS: (ESI^+) calculated for $C_{17}H_{18}NO$ 252.1383. Found $[M+H]^+$ 252.1386; m.p. 113 - 115 °C ($CDCl_3$).

Characteristic signals for rotamer B: ^{1}H NMR (400 MHz, $CDCl_3$): δ 8.08 (1H, d, *J* = 8.5 Hz, C**5**-H), 5.84 (1H, s, C**11**-H_2), 5.33 (1H, s, C**11**-H_2).

The structure of compound ***4ba*** *was confirmed by single crystal X-ray diffraction of crystals obtained from $CDCl_3$ (Figure 1).*

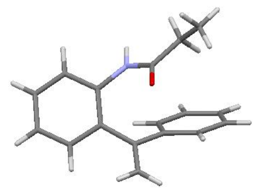

Figure 1

_N_-(2-(1-Phenylvinyl)phenyl)isobutyramide (4ca)

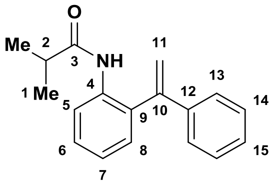

General Procedure E: The reaction was carried out with $Ir(cod)_2OTf$ (7.5 mol%), **L-4** (7.5 mol%) and 1,4-dioxane (0.5 M with respect to substrate) and was run for 72 h. The crude material was purified by FCC (2 columns; hexane/EtOAc 20%) to afford the title compound (30.7 mg, 81%, 0.98:0.02 mixture of rotamers *A:B*) as an orange oil. ν_{max} / cm^{-1}: 3418 (m), 2966 (m), 1676 (s), 1515 (s), 1444 (s); *Signals for rotamer A*: ^{1}H NMR (400 MHz, $CDCl_3$): δ 8.26 (1H, d, *J* = 8.0 Hz, C**5**-H), 7.41 – 7.35 (1H, m, C**6**-H), 7.35 – 7.31 (5H, m, C**13**-H, C**14**-H, C**15**-H), 7.30 – 7.25 (1H, m, C**8**-H), 7.15 (1H, ddd, *J* = 8.0, 8.0, 1.0 Hz, C**7**-H), 7.03 (1H, s, N-H), 5.90 (1H, d, *J* = 1.5 Hz, C**11**-H_2), 5.38 (1H, d, *J* = 1.5 Hz, C**11**-H_2), 2.13 (1H, sept, *J* = 7.0 Hz, C**2**-H), 0.91 (6H, d, *J* = 7.0 Hz, C**1**-H_3); ^{13}C NMR (101 MHz, $CDCl_3$): δ 174.8 (C**3**), 146.5 (C**10**), 139.2 (C**12**), 135.4 (C**4**), 131.7 (C**9**), 130.5 (C**8**), 129.0 (ArCH), 128.9 (ArCH), 128.7 (ArCH), 126.6 (ArCH), 124.1 (C**7**), 121.4 (C**5**), 117.5 (C**11**), 36.8 (C**2**), 19.2 (C**1**); HRMS: (ESI^+) calculated for $C_{18}H_{19}NONa$ 288.1359. Found $[M+Na]^+$ 288.1362.

Characteristic signals for rotamer B: ^{1}H NMR (400 MHz, CDCl$_3$): δ 8.13 (1H, d, *J* = 8.5 Hz, C**5**-H), 5.86 (1H, d, *J* = 1.0 Hz, C**11**-H_2), 5.33 (1H, d, *J* = 1.0 Hz, C**11**-H_2).

N-(2-(1-Phenylvinyl)phenyl)pivalamide (4da)

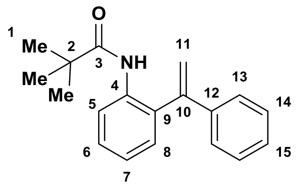

General Procedure E: The reaction was carried out with Ir(cod)$_2$OTf (7.5 mol%), **L-4** (7.5 mol%) and 1,4-dioxane (0.5 M with respect to substrate) and was run for 96 h. The crude material was purified by FCC (2 columns; hexane/EtOAc 0-10%) to afford the title compound (26.0 mg, 65%, 0.97:0.03 mixture of rotamers *A:B*) as a brown oil. ν_{max} / cm^{-1}: 3431 (m), 2959 (m), 1686 (s), 1516 (s), 1300 (s); *Signals for rotamer A*: ^{1}H NMR (400 MHz, CDCl$_3$): δ 8.29 (1H, dd, *J* = 8.0, 1.0 Hz, C**5**-H), 7.40 – 7.36 (1H, m, C**6**-H), 7.37 – 7.30 (6H, m, C**13**-H, C**14**-H, C**15**-H, N-H), 7.29 – 7.24 (1H, m, C**8**-H), 7.14 (1H, ddd, *J* = 8.0, 8.0, 1.0 Hz, C**7**-H), 5.92 (1H, d, *J* = 1.5 Hz, C**11**-H_2), 5.38 (1H, d, *J* = 1.5 Hz, C**11**-H_2), 0.94 (9H, s, C**1**-H_3); ^{13}C NMR (101 MHz, CDCl$_3$): δ 176.4 (C**3**), 146.5 (C**10**), 138.8 (C**12**), 135.6 (C**4**), 131.7 (C**9**), 130.6 (C**8**), 129.1 (ArCH), 129.0 (ArCH), 128.8 (ArCH), 126.61 (ArCH), 124.1 (C**7**), 121.3 (C**5**), 117.4 (C**11**), 39.7 (C**2**), 27.3 (C**1**); HRMS: (ESI$^+$) calculated for $C_{19}H_{21}NONa$ 302.1515. Found [M+Na]$^+$ 302.1518.

Characteristic signals for rotamer B: ^{1}H NMR (400 MHz, CDCl$_3$): δ 8.17 (1H, d, *J* = 8.0 Hz, C**5**-H), 5.88 (1H, s, C**11**-H_2), 5.33 (1H, s, C**11**-H_2).

N-(2-(1-Phenylvinyl)phenyl)cyclobutanecarboxamide (4ea)

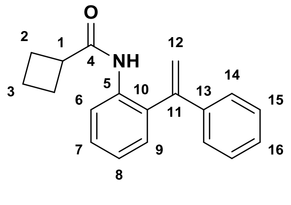

General Procedure E: The reaction was carried out with Ir(cod)$_2$OTf (7.5 mol%), **L-4** (7.5 mol%) and 1,4-dioxane (0.5 M with respect to substrate) and was run for 72 h. The crude material was purified by FCC (toluene/Et$_2$O 0-2%) to afford the title compound (22.9 mg, 57%) as a yellow oil. ν_{max} / cm^{-1} : 3411 (m), 2942 (m), 1680 (s), 1515 (s); ^{1}H NMR (400 MHz, CDCl$_3$): δ 8.26 (1H, d, *J* = 8.5 Hz, C**6**-H), 7.41 – 7.35 (1H, m, C**7**-H), 7.34 – 7.31 (5H, m, C**13**-H, C**14**-H, C**16**-H), 7.30 – 7.24 (1H, m, C**9**-H), 7.14 (1H, ddd, *J* = 7.5, 7.5, 1.0 Hz, C**8**-H), 6.90 (1H, s, N-H), 5.87 (1H, d, *J* = 1.5 Hz, C**12**-H_2), 5.37 (1H, d,

J = 1.5 Hz, C**12**-H_2), 2.79 (1H, app. p, J = 8.5 Hz, C**1**-H), 2.04 – 1.59 (6H, m, C**2**-H_2, C**3**-H_2); ^{13}C NMR (101 MHz, $CDCl_3$): δ 173.0 (C**4**), 146.4 (C**11**), 139.3 (C**13**), 135.4 (C**5**), 131.6 (C**10**), 130.5 (C**9**), 129.0 (C**15**), 129.0 (C**7**), 128.7 (C**16**), 126.6 (C**14**), 124.1 (C**8**), 121.3 (C**6**), 117.5 (C**12**), 40.8 (C**1**), 25.1 (C**2**), 17.9 (C**3**); HRMS: (ESI$^+$) calculated for $C_{19}H_{20}NO$ 278.1539. Found $[M+H]^+$ 278.1545.

***N*-(2-(1-Phenylvinyl)phenyl)cyclopentanecarboxamide (4fa)**

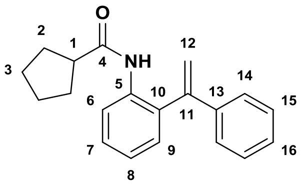

General Procedure E: The reaction was carried out with $Ir(cod)_2OTf$ (7.5 mol%), **L-4** (7.5 mol%) and 1,4-dioxane (0.5 M with respect to substrate) and was run for 72 h. The crude material was purified by FCC (hexane/EtOAc 0-5%) to afford the title compound (33.0 mg, 79%) as a yellow oil. ν_{max} / cm^{-1}: 3415 (m), 2952 (m), 1681 (s), 1514 (s), 1443 (s); ^{1}H NMR (400 MHz, $CDCl_3$): δ 8.26 (1H, d, J = 8.0 Hz, C**6**-H), 7.42 – 7.28 (6H, m, C**7**-H, C**14**-H, C**15**-H, C**16**-H), 7.26 (1H, d, J = 7.5 Hz, C**9**-H), 7.13 (1H, dd, J = 7.5, 7.5 Hz, C**8**-H), 7.02 (1H, br s, N-H), 5.89 (1H, s, C**12**-H_2), 5.37 (1H, s, C**12**-H_2), 2.37 – 2.19 (1H, m, C**1**-H), 1.69 – 1.34 (8H, m, C**2**-H_2, C**3**-H_2); ^{13}C NMR (101 MHz, $CDCl_3$): δ 174.1 (C**4**), 146.5 (C**11**), 139.3 (C**13**), 135.6 (C**5**), 131.5 (C**10**), 130.4 (C**9**), 129.0 (ArCH), 128.9 (ArCH), 128.7 (ArCH), 126.6 (ArCH), 124.0 (C**8**), 121.4 (C**6**), 117.4 (C**12**), 47.0 (C**1**), 30.0 (Cyclopentyl), 25.9 (Cyclopentyl); HRMS: (ESI$^+$) calculated for $C_{20}H_{22}NO$ 292.1696. Found $[M+H]^+$ 292.1685.

***N*-(2-(1-Phenylvinyl)phenyl)cyclohexanecarboxamide (4ga)**

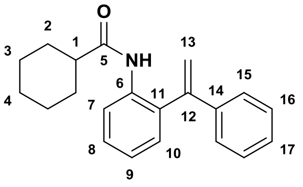

General Procedure E: The reaction was carried out with $Ir(cod)_2OTf$ (7.5 mol%), **L-4** (7.5 mol%) and 1,4-dioxane (0.5 M with respect to substrate) and was run for 72 h. The crude material was purified by FCC (2 columns; hexane/EtOAc 0-10%) to afford the title compound (31.2 mg, 71%) as a yellow powder. ν_{max} / cm^{-1}: 3418 (m), 2926 (m), 1679 (s), 1515 (s), 1279 (s); ^{1}H NMR (400 MHz, $CDCl_3$): δ 8.27 (1H, d, J = 8.0 Hz, C**7**-H), 7.41 – 7.36 (1H, m, C**8**-H), 7.34 – 7.30 (5H, m, C**15**-H, C**16**-H, C**17**-H), 7.29 – 7.25 (1H, m, C**10**-H), 7.14 (1H, ddd, J = 8.0, 8.0, 1.0 Hz, C**9**-H), 7.03 (1H, br. s, N-H), 5.89 (1H, d, J = 1.0 Hz, C**13**-H_2), 5.38 (1H, d, J = 1.0 Hz, C**13**-H_2), 1.94 – 1.78 (1H, m, C**1**-H), 1.74 – 1.62 (2H, m, cy), 1.63 – 1.49 (3H, m, cy), 1.22 – 1.01 (5H, m, cy); ^{13}C NMR (101 MHz, $CDCl_3$): δ 174.0 (C**5**), 146.6 (C**12**), 139.3 (C**14**), 135.5 (C**6**), 131.6 (C**11**), 130.5 (ArCH), 129.0 (ArCH), 128.9 (ArCH),

128.7 (ArCH), 126.7 (C**9**), 124.1 (C**7**), 117.6 (C**13**), 46.6 (C**1**), 29.3 (Cy), 25.7 (Cy), 25.7 (Cy); HRMS: (ESI$^+$) calculated for $C_{21}H_{23}NONa$ 328.1672. Found $[M+Na]^+$ 328.1671; m.p. 57 - 59 °C ($CDCl_3$).

3-Methyl-*N*-(2-(1-phenylvinyl)phenyl)butanamide (4ha)

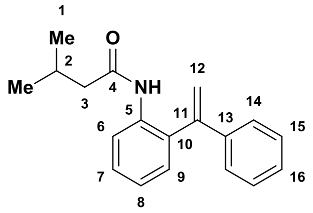

General Procedure E: The reaction was carried out with $Ir(cod)_2OTf$ (7.5 mol%), **L-4** (7.5 mol%) and 1,4-dioxane (0.5 M with respect to substrate) and was run for 72 h. The crude material was purified by FCC (toluene/Et_2O 0-2%) to afford the title compound (27.2 mg, 68%) as a yellow oil. ν_{max} / cm^{-1} : 3417 (m), 2957 (m), 1671 (s), 1516 (s); ^{1}H NMR (400 MHz, $CDCl_3$): δ 8.26 (1H, d, *J* = 8.0 Hz, C**6**-H), 7.45 – 7.35 (1H, m, C**7**-H), 7.36 – 7.29 (5H, m, C**14**-H, C**15**-H, C**16**-H), 7.27 (1H, dd, *J* = 6.0, 1.5 Hz, C**9**-H), 7.15 (1H, dd, *J* = 7.5, 7.5 Hz, C**8**-H), 6.98 (1H, s, N-H), 5.88 (1H, d, *J* = 1.5 Hz, C**12**-H_2), 5.37 (1H, d, *J* = 1.5 Hz, C**12**-H_2), 1.89 – 1.77 (3H, m, C**2**-H, C**3**-H_2), 0.81 – 0.76 (6H, m, C**1**-H_3); ^{13}C NMR (101 MHz, $CDCl_3$): δ 170.6 (C**4**), 146.5 (C**11**), 139.3 (C**13**), 135.4 (C**5**), 131.6 (C**10**), 130.4 (C**9**), 129.0 (C**14**), 128.9 (C**7**), 128.7 (C**16**), 126.6 (C**15**), 124.2 (C**8**), 121.4 (C**6**), 117.4 (C**12**), 47.3 (C**2**), 26.2 (C**3**), 22.4 (C**1**); HRMS: (ESI$^+$) calculated for $C_{19}H_{22}NO$ 280.1696. Found $[M+H]^+$ 280.1707.

3-Phenyl-*N*-(2-(1-phenylvinyl)phenyl)propanamide (4ia)

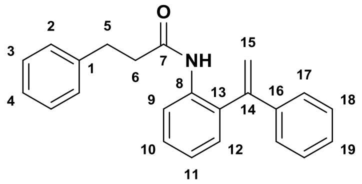

General Procedure E: The reaction was carried out with $Ir(cod)_2OTf$ (7.5 mol%), **L-4** (7.5 mol%) and 1,4-dioxane (0.5 M with respect to substrate) and was run for 72 h. The crude material was purified by FCC (hexane/EtOAc 0-10%) to afford the title compound (33.9 mg, 74%) as a yellow oil. ν_{max} / cm^{-1}: 3416 (m), 3026 (m), 2923 (s) 1669 (s), 1516 (s); ^{1}H NMR (400 MHz, $CDCl_3$): δ 8.21 (1H, d, *J* = 8.0 Hz, C**9**-H), 7.41 – 7.36 (1H, m, C**10**-H), 7.35 – 7.23 (8H, m, C**3**-H, C**12**-H, C**17**-H, C**18**-H, C**19**-H), 7.22 – 7.14 (2H, m, C**11**-H, C**4**-H), 7.12 – 7.08 (2H, m, C**2**-H), 6.95 (1H, s, N-H), 5.83 (1H, s, C**15**-H_2), 5.30 (1H, s, C**15**-H_2), 2.78 – 2.71 (2H, m, C**5**-H_2), 2.32 – 2.23 (2H, m, C**6**-H_2); ^{13}C NMR (101 MHz, $CDCl_3$): δ 170.1 (C**7**), 146.3 (C**14**), 140.6 (C**1**), 139.4 (C**16**), 135.2 (C**8**), 131.9 (C**13**), 130.4 (C**12**), 129.0 (ArCH), 128.9 (ArCH), 128.7 (ArCH), 128.6 (ArCH), 128.3 (C**2**), 126.6 (ArCH), 126.3 (C**4**), 124.4 (C**11**), 121.8 (C**9**), 117.5 (C**15**), 39.4 (C**6**), 31.4 (C**5**); HRMS: (ESI$^+$) calculated for $C_{23}H_{22}NO$ 328.1696. Found $[M+H]^+$ 328.1696.

***N*-(5-Methyl-2-(1-phenylvinyl)phenyl)acetamide (4ja)**

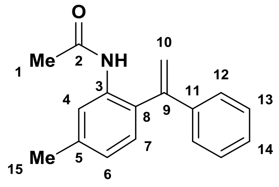

General Procedure E: The reaction was carried out with Ir(cod)$_2$OTf (7.5 mol%), **L-4** (7.5 mol%) and 1,4-dioxane (0.5 M with respect to substrate) and was run for 72 h. Purification of the residue by FCC (toluene/EtOAc 10-30%) afforded the title compound (27.5 mg, 77%) as a colorless solid. ν_{max} / cm^{-1}: 2253 (m), 1686 (m), 1381 (m), 904 (m), 726 (s); ^{1}H NMR (CDCl$_3$, 400 MHz): δ 7.97 (1H, s, C**4**-H), 7.40 – 7.25 (5H, m, C**12**-H, C**13**-H, **14**-H), 7.15 (1H, d, *J* = 8.0 Hz, C**7**-H), 6.97 (1H, d, *J* = 8.0 Hz, C**6**-H), 6.90 (1H, s, N-H), 5.83 (1H, s, C**10**-H$_2$), 5.35 (1H, s, C**10**-H$_2$), 2.39 (3H, s, C**15**-H$_3$), 1.77 (3H, s, C**1**-H$_3$); ^{13}C NMR (CDCl$_3$, 100 MHz): 168.4 (C**2**), 146.8 (C**11**), 140.0 (C**9**), 139.2 (C**8**), 135.3 (C**5**), 130.5 (C**7**), 129.5 (C**3**), 129.2 (ArCH), 128.8 (ArCH), 126.9 (ArCH), 125.5 (C**6**), 122.8 (C**4**), 117.5 (C**10**); 24.7 (C**1**), 21.9 (C**15**); HRMS: (ESI$^+$) calculated for $C_{17}H_{18}NO$: 252.1383. Found [M+H]$^+$; 252.1383; m.p. 98 - 100 °C (CDCl$_3$).

***N*-(5-(1-Phenylvinyl)-2,3-dihydro-1*H*-inden-4-yl)acetamide (4ka)**

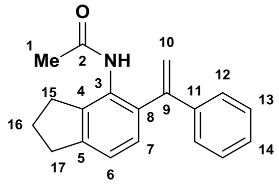

General Procedure E: The reaction was carried out with Ir(cod)$_2$OTf (7.5 mol%), **L-4** (7.5 mol%) and 1,4-dioxane (0.5 M with respect to substrate) and was run for 72 h. The crude material was purified by FCC (1st column: hexane/EtOAc 10-40%; 2nd column: hexane/EtOAc 40%) to afford the title compound (33.1 mg, 83%, 0.92:0.08 mixture of rotamers *A:B*) as an off-white solid. ν_{max} / cm^{-1}: 3255 (m), 2952 (m), 1660 (s), 1524 (s), 1289 (s); *Signals for rotamer A*: ^{1}H NMR (400 MHz, CDCl$_3$): δ 7.35 – 7.27 (5H, m, C**12**-H, C**13**-H, C**14**-H), 7.20 – 7.14 (2H, m, C**6**-H, C**7**-H), 6.51 (1H, br. s, N-H), 5.70 (1H, d, *J* = 1.5 Hz, C**10**-H$_2$), 5.31 (1H, d, *J* = 1.5 Hz, C**10**-H$_2$), 2.98 (2H, t, *J* = 7.5 Hz, C**17**-H$_2$), 2.81 (2H, t, *J* = 7.5 Hz, C**15**-H$_2$), 2.14 – 2.01 (2H, m, C**16**-H$_2$), 1.63 (3H, s, C**1**-H$_3$); ^{13}C NMR (101 MHz, CDCl$_3$): δ 167.9 (C**2**), 147.7 (C**9**), 146.1 (C**5**), 142.2 (C**4**), 140.7 (C**11**), 135.0 (C**8**), 130.6 (C**3**), 128.8 (C**6**), 128.8 (ArCH), 128.1 (ArCH), 126.5 (ArCH), 122.9 (C**7**), 116.5 (C**10**), 33.4 (C**17**), 32.0 (C**15**), 25.4 (C**16**), 22.9 (C**1**); HRMS: (ESI$^+$) calculated for $C_{19}H_{19}NONa$ 300.1359. Found [M+Na]$^+$ 300.1356; m.p. 106 - 108 °C (CDCl$_3$).

Characteristic signals for rotamer B: ^{1}H NMR (400 MHz, CDCl$_3$): δ 6.35 (1H, s, N-H), 5.66 (1H, s, C**10**-H$_2$), 5.26 (1H, s, C**10**-H$_2$).

***N*-(5-(1-Phenylvinyl)-2,3-dihydrobenzofuran-4-yl)acetamide (4la)**

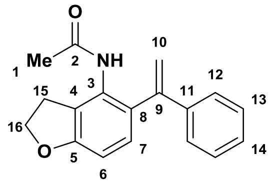

General Procedure E: The reaction was carried out with Ir(cod)$_2$OTf (7.5 mol%), **L-4** (7.5 mol%) and 1,4-dioxane (0.5 M with respect to substrate) and was run for 96 h. The crude material was purified by FCC (hexane/EtOAc 30-50%) to afford the title compound (27.2 mg, 68%, 0.98:0.02 mixture of rotamers *A:B*) as an off-white solid. ν_{max} / cm^{-1}: 3259 (m), 2927 (m), 1746 (s), 1666 (s), 1462 (s); *Signals for rotamer A*: ^{1}H NMR (400 MHz, CDCl$_3$): δ 7.42 – 7.28 (5H, m, C**12**-H, C**13**-H, C**14**-H), 7.13 (1H, d, *J* = 8.0 Hz, C**7**-H), 6.72 (1H, d, *J* = 8.0 Hz, C**6**-H), 6.52 (1H, s, N-H), 5.67 (1H, d, *J* = 1.5 Hz, C**10**-H$_2$), 5.29 (1H, d, *J* = 1.5 Hz, C**10**-H$_2$), 4.61 (2H, t, *J* = 8.5 Hz, C**16**-H$_2$), 3.15 (2H, t, *J* = 8.5 Hz, C**15**-H$_2$), 1.60 (3H, s, C**1**-H$_3$); ^{13}C NMR (101 MHz, CDCl$_3$): δ 167.6 (C**2**), 161.3 (C**5**), 147.2 (C**9**), 140.7 (C**11**), 131.4 (C**3**), 130.4 (C**7**), 128.9 (ArCH), 128.6 (C**8**), 128.2 (C**14**), 126.5 (ArCH), 124.8 (C**4**), 116.6 (C**10**), 107.3 (C**6**), 71.9 (C**16**), 29.8 (C**15**), 23.0 (C**1**); HRMS: (ESI$^+$) calculated for $C_{18}H_{17}NO_2Na$ 302.1151. Found [M+Na]$^+$ 302.1148; m.p. 143 - 145 °C (CDCl$_3$).

Characteristic signals for rotamer B: ^{1}H NMR (400 MHz, CDCl$_3$): 6.66 (1H, d, *J* = 8.0 Hz, C**6**-H), 5.63 (1H, s, C**10**-H$_2$), 5.24 (1H, s, C**10**-H$_2$).

N-(5-Methoxy-2-(1-phenylvinyl)phenyl)acetamide (4ma)

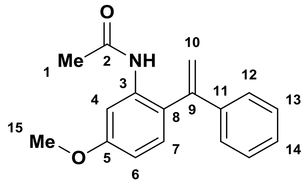

General Procedure E: The reaction was carried out with Ir(cod)$_2$OTf (7.5 mol%), **L-4** (7.5 mol%) and 1,4-dioxane (0.5 M with respect to substrate) and was run for 72 h. The crude material was purified by FCC (hexane/EtOAc 10-30%) to afford the title compound (24.9 mg, 65%, 0.97:0.03 mixture of rotamers *A:B*) as a brown oil. ν_{max} / cm^{-1}: 3280 (m), 2936 (m), 1676 (s), 1524 (s), 1239 (s); *Signals for rotamer A*: ^{1}H NMR (400 MHz, CDCl$_3$): δ 7.88 (1H, d, *J* = 2.5 Hz, C**4**-H), 7.38 – 7.28 (5H, m, C**12**-H, C**13**-H, C**14**-H), 7.16 (1H, d, *J* = 8.5 Hz, C**7**-H), 7.01 (1H, br. s, N-H), 6.70 (1H, dd, *J* = 8.5, 2.5 Hz, C**6**-H), 5.81 (1H, d, *J* = 1.5 Hz, C**10**-H$_2$), 5.34 (1H, d, *J* = 1.5 Hz, C**10**-H$_2$), 3.84 (3H, s, C**15**-H$_3$), 1.79 (3H, s, C**1**-H$_3$); ^{13}C NMR (101 MHz, CDCl$_3$): δ 168.2 (C**2**), 160.0 (C**5**), 146.2 (C**9**), 139.9 (C**11**), 136.4 (C**3**), 131.1 (C**7**), 128.9 (ArCH), 128.6 (C**14**), 126.8 (ArCH), 123.9 (C**8**), 117.2 (C**10**), 110.5 (C**6**), 106.6 (C**4**), 55.6 (C**15**), 24.5 (C**1**); HRMS: (ESI$^+$) calculated for $C_{17}H_{17}NO_2Na$: 290.1151. Found [M+Na]$^+$ 290.1146.

Characteristic signals for rotamer B: ^{1}H NMR (400 MHz, $CDCl_3$): δ 7.79 (1H, s, C**4**-H), 5.79 (1H, s, C**10**-H_2), 5.30 (1H, s, C**10**-H_2).

***N*-(3-(1-Phenylvinyl)naphthalen-2-yl)acetamide (4na)**

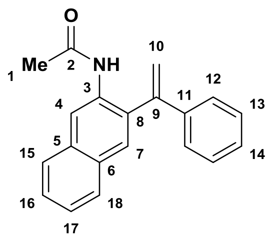

General Procedure E: The reaction was carried out with $Ir(cod)_2OTf$ (7.5 mol%), **L-4** (7.5 mol%) and 1,4-dioxane (0.5 M with respect to substrate) and was run for 72 h. The crude material was purified by FCC (1st column: hexane/EtOAc 30-50%; 2nd column: hexane/EtOAc 10-30%) to afford the title compound (29.6 mg, 72%) as a brown solid. ν_{max} / cm^{-1}: 3416 (m), 3054 (m), 1673 (s), 1525 (s), 1483 (s); ^{1}H NMR (400 MHz, $CDCl_3$): δ 8.70 (1H, s, C**4**-H), 7.87 (1H, d, *J* = 8.0 Hz, C**15**-H), 7.82 – 7.75 (2H, m, C**7**-H, C**18**-H), 7.52 – 7.40 (2H, m, C**16**-H, C**17**-H), 7.39 – 7.31(5H, m, C**12**-H, C**13**-H, C**14**-H), 7.10 (1H, br. s, N-H), 5.96 (1H, s, C**10**-H_2), 5.50 (1H, s, C**10**-H_2), 1.84 (3H, s, C**1**-H_3); ^{13}C NMR (101 MHz, $CDCl_3$): δ 168.1 (C**2**), 146.3 (C**9**), 139.3 (C**11**), 133.7 (C**5**), 132.9 (C**3**), 131.9 (C**8**), 130.3 (C**6**), 129.4 (ArCH), 129.0 (ArCH), 128.7 (ArCH), 127.8 (C**15**), 127.5 (ArCH), 126.6 (ArCH), 126.5 (C**16**), 125.4 (C**17**), 118.5 (C**4**), 117.8 (C**10**), 24.4 (C**1**); HRMS: (ESI^+) calculated for $C_{20}H_{17}NONa$ 310.1202. Found $[M+Na]^+$ 310.1213; m.p. 101 - 103 °C ($CDCl_3$).

***N*-(4-Bromo-2-(1-phenylvinyl)phenyl)acetamide (4oa)**

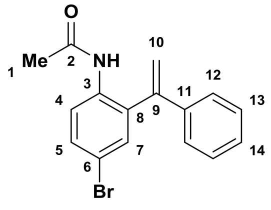

General Procedure E: The reaction was carried out with $Ir(cod)_2OTf$ (10 mol%), **L-4** (10 mol%). 1,4-dioxane (0.5 M with respect to substrate) and was run for 72 h. The crude material was purified by FCC (hexane/EtOAc 10-30%) to afford the title compound (27.7 mg, 61%) as off-white needles. ν_{max} / cm^{-1}: 3286 (m), 3027 (m), 1670 (s), 1506 (s), 1293 (s); ^{1}H NMR (400 MHz, $CDCl_3$): δ 8.09 (1H, d, *J* = 9.0 Hz, C**4**-H), 7.48 (1H, dd, *J* = 9.0, 2.5 Hz, C**5**-H), 7.41 (1H, d, *J* = 2.5 Hz, C**7**-H), 7.39 – 7.28 (5H, m, C**12**-H, C**13**-H, C**14**-H), 6.90 (1H, br. s, N-H), 5.89 (1H, s, C**10**-H_2), 5.38 (1H, s, C**10**-H_2), 1.78 (3H, s, C**1**-H_3); ^{13}C NMR (101 MHz, $CDCl_3$): δ 168.1 (C**2**), 145.3 (C**9**), 138.7 (C**11**), 134.5 (C**3**), 133.7 (C**8**), 132.9 (C**7**), 131.8 (C**5**), 129.2 (ArCH), 129.0 (C**14**), 126.6 (ArCH), 123.3 (C**4**), 118.2 (C**10**), 117.1 (C**6**), 24.4 (C**1**); HRMS: (ESI^+) calculated for $C_{16}H_{14}BrNONa$ 338.0151. Found $[M+Na]^+$ 338.0156; m.p. 118 - 120 °C ($CDCl_3$).

Methyl 4-acetamido-3-(1-phenylvinyl)benzoate (4pa)

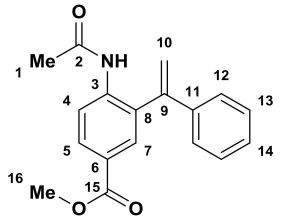

General Procedure E: The reaction was carried out with Ir(cod)$_2$OTf (7.5 mol%), **L-4** (7.5 mol%) and 1,4-dioxane (0.5 M with respect to substrate) and was run for 96 h. The crude material was purified by FCC (1st column: hexane/EtOAc 30-50%; 2nd column: hexane/EtOAc 40-50%) to afford the title compound (22.2 mg, 58%) as a yellow oil. ν_{max} / cm^{-1}: 3312 (m), 2951 (m), 1710 (s), 1512 (s), 1256 (s); ^{1}H NMR (400 MHz, CDCl$_3$): δ 8.38 (1H, d, *J* = 8.5 Hz, C**4**-H), 8.04 (1H, dd, *J* = 8.5, 2.0 Hz, C**5**-H), 7.95 (1H, d, *J* = 2.0 Hz, C**7**-H), 7.38 – 7.28 (5H, m, C**12**-H, C**13**-H, C**14**-H), 7.17 (1H, s, N-H), 5.94 (1H, d, *J* = 1.0 Hz, C**10**-H$_2$), 5.42 (1H, d, *J* = 1.0 Hz, C**10**-H$_2$), 3.90 (3H, s, C**16**-H$_3$), 1.83 (3H, s, C**1**-H$_3$); ^{13}C NMR (101 MHz, CDCl$_3$): δ 168.3 (C**2**), 166.7 (C**15**), 145.5 (C**9**), 139.6 (C**3**), 138.8 (C**11**), 131.8 (C**7**), 130.9 (C**8**), 130.6 (C**5**), 129.2 (ArCH), 129.0 (C**14**), 126.6 (ArCH), 125.6 (C**6**), 120.4 (C**4**), 118.4 (C**10**), 52.2 (C**16**), 24.6 (C**1**); HRMS: (ESI$^+$) calculated for $C_{18}H_{17}NO_3Na$ 318.1101. Found [M+Na]$^+$ 318.1098.

***N*-(4-Methyl-2-(1-phenylvinyl)phenyl)acetamide (4qa)**

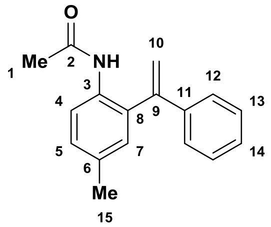

General Procedure E: The reaction was carried out with Ir(cod)$_2$OTf (7.5 mol%), **L-4** (7.5 mol%) and 1,4-dioxane (0.5 M with respect to substrate) and was run for 72 h. The crude material was purified by FCC (hexane/EtOAc 10-30%) to afford the title compound (25.4 mg, 71%) as a brown oil. ν_{max} / cm^{-1}: 3414 (m), 3024 (m), 1664 (s), 1514 (s), 1498 (s); ^{1}H NMR (400 MHz, CDCl$_3$): δ 7.98 (1H, d, *J* = 8.5 Hz, C**4**-H), 7.41 – 7.22 (5H, m, C**12**-H, C**13**-H, C**14**-H), 7.18 (1H, dd, *J* = 8.5, 2.0 Hz, C**5**-H), 7.12 – 7.03 (1H, m, C**7**-H), 6.88 (1H, br. s, N-H), 5.84 (1H, s, C**10**-H$_2$), 5.35 (1H, s, C**10**-H$_2$), 2.34 (3H, s, C**1**-H$_3$), 1.77 (3H, s, C**15**-H$_3$); ^{13}C NMR (101 MHz, CDCl$_3$): δ 168.1 (C**2**), 146.6 (C**9**), 139.6 (C**11**), 134.1 (C**6**), 132.7 (C**3**), 132.2 (C**8**), 130.8 (C**7**), 129.4 (C**5**), 128.9 (ArCH), 128.6 (C**14**), (ArCH), 122.2 (C**4**), 117.1 (C**10**), 24.3 (C**15**), 21.0 (C**1**); HRMS: (ESI$^+$) calculated for $C_{17}H_{17}NONa$ 274.1202. Found [M+Na]$^+$ 274.1199.

***N*-(4-(*Tert*-butyl)-2-(1-(*p*-tolyl)vinyl)phenyl)cyclopentanecarboxamide (4rb)**

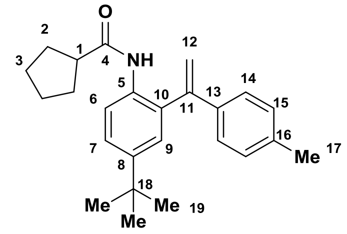

General Procedure E: The reaction was carried out with Ir(cod)$_2$OTf (7.5 mol%), **L-4** (7.5 mol%) and 1,4-dioxane (0.5 M with respect to substrate) and was run for 72 h. The crude material was purified by FCC (hexane/Et$_2$O 0-10%) to afford the title compound (48.8 mg, 94%) as a yellow oil. ν_{max}/ Cm^{-1} : 3311 (m), 2957 (m), 1683 (s), 1511 (s); ^{1}H NMR (400 MHz, CDCl$_3$): δ 8.13 (1H, d, *J* = 8.5 Hz, C**6**-H), 7.38 (1H, dd, *J* = 8.5, 2.5 Hz, C**7**-H), 7.26 – 7.23 (2H, m, C**14**-H), 7.22 (1H, s, C**9**-H), 7.14 (2H, d, *J* = 8.0 Hz, C**15**-H), 6.98 (1H, s, N-H), 5.84 (1H, d, *J* = 1.5 Hz, C**12**-H$_2$), 5.30 (1H, d, *J* = 1.5 Hz, C**12**-H$_2$), 2.35 (3H, s, C**17**-H$_3$), 2.33 – 2.24 (1H, m, C**1**-H), 1.73 – 1.40 (8H, m, C**3**-H$_2$, C**2**-H$_2$), 1.32 (9H, s, C**19**-H$_3$); ^{13}C NMR (101 MHz, CDCl$_3$): δ 174.1 (C**4**), 146.9 (C**8**), 146.8 (C**11**), 138.6 (C**16**), 136.5 (C**13**), 133.0 (C**5**), 131.4 (C**10**), 129.6 (C**15**), 127.2 (C**9**), 127.2 (C**14**), 125.7 (C**7**), 121.1 (C**6**), 116.2 (C**12**), 46.9 (C**1**), 34.5 (C**18**), 31.5 (C**19**), 30.1 (Cyclopentyl), 25.9 (Cyclopentyl), 21.3 (C**17**); HRMS: (ESI$^+$) calculated for $C_{25}H_{32}NO$ 362.2478. Found [M+H]$^+$ 362.2482.

***N*-(5-Isopropyl-2-(1-(*p*-tolyl)vinyl)phenyl)cyclopentanecarboxamide (4sb)**

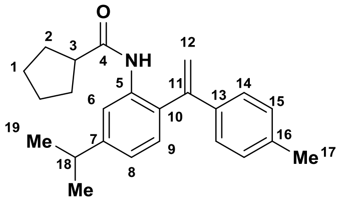

General Procedure E: The reaction was carried out with Ir(cod)$_2$OTf (7.5 mol%), **L-4** (7.5 mol%) and 1,4-dioxane (0.5 M with respect to substrate) and was run for 72 h. The crude material was purified by FCC (hexane/Et$_2$O 0-10%) to afford the title compound (38.6 mg, 78%) as a yellow oil. ν_{max}/ Cm^{-1} : 3414 (m), 2958 (m), 1683 (s), 1523 (s); ^{1}H NMR (400 MHz, CDCl$_3$): δ 8.20 (1H, s, C**6**-H), 7.23 (2H, d, *J* = 8.0 Hz, C**14**-H), 7.19 – 7.11 (3H, m, C**9**-H, C**15**-H), 7.07 (1H, s, N-H), 6.99 (1H, dd, *J* = 7.5, 2.0 Hz, C**8**-H), 5.81 (1H, d, *J* = 1.5 Hz, C**12**-H$_2$), 5.30 (1H, d, *J* = 1.5 Hz, C**12**-H$_2$), 2.95 (1H, hept, *J* = 7.0 Hz, C**18**-H), 2.35 (3H, s, C**17**-H$_3$), 2.34 – 2.23 (1H, m, C**1**-H), 1.67 – 1.42 (8H, m, C**3**-H$_2$, C**2**-H$_2$), 1.30 (6H, d, *J* = 7.0 Hz, C**19**-H$_3$); ^{13}C NMR (101 MHz, CDCl$_3$): δ 174.2 (C**4**), 149.8 (C**7**), 146.4 (C**11**), 138.6 (C**16**), 136.7 (C**13**), 135.5 (C**5**), 130.2 (C**9**), 129.6 (C**15**), 129.1 (C**10**), 126.7 (C**14**), 121.9 (C**8**), 119.4 (C**6**), 116.3 (C**12**), 47.0 (C**1**), 34.3 (C**18**), 30.0 (Cyclopentyl), 25.9 (Cyclopentyl), 24.0 (C**19**), 21.3 (C**17**); HRMS: (ESI$^+$) calculated for $C_{24}H_{30}NO$ 348.2322. Found [M+H]$^+$ 362.2332.

***N*-(4-(1-(*p*-Tolyl)vinyl)-[1,1'-biphenyl]-3-yl)cyclopentanecarboxamide (4tb)**

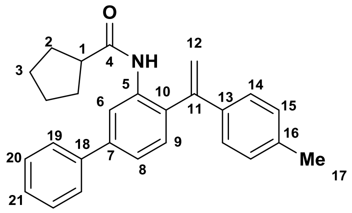

General Procedure E: The reaction was carried out with $Ir(cod)_2OTf$ (7.5 mol%), **L-4** (7.5 mol%) and 1,4-dioxane (0.5 M with respect to substrate) and was run for 72 h. The crude material was purified by FCC (hexane/Et_2O 0-10%) to afford the title compound (38.3 mg, 70%) as a yellow oil. V_{max}/ Cm^{-1} : 3412 (m), 2952 (m), 1683 (s), 1525 (s); ^1H NMR (400 MHz, $CDCl_3$): δ 8.62 (1H, d, *J* = 2.0 Hz, C**6**-H), 7.73 – 7.66 (2H, m, C**19**-H), 7.47 – 7.41 (2H, m, C**20**-H), 7.41 – 7.32 (3H, m, C**8**-H, C**9**-H, C**21**-H), 7.28 (2H, d, *J* = 8.0 Hz, C**14**-H), 7.17 (2H, d, *J* = 8.0 Hz, C**15**-H), 7.13 (1H, s, N-H), 5.87 (1H, d, *J* = 1.5 Hz, C**12**-H_2), 5.37 (1H, d, *J* = 1.5 Hz, C**12**-H_2), 2.37 (3H, s, C**17**-H_3), 2.38 – 2.25 (1H, m, C**1**-H), 1.69 – 1.46 (8H, m, C**3**-H_2, C**2**-H_2); ^{13}C NMR (101 MHz, $CDCl_3$): δ 174.3 (C**4**), 146.1 (C**11**), 141.7 (C**7**), 140.6 (C**18**), 138.8 (C**16**), 136.5 (C**13**), 136.0 (C**5**), 130.8 (C**10**), 130.5 (C**8**), 129.7 (C**15**), 128.8 (C**20**), 127.6 (C**21**), 127.3 (C**19**), 126.7 (C**14**), 122.5 (C**9**), 119.9 (C**6**), 116.6 (C**12**), 47.0 (C**1**), 30.1 (Cyclopentyl), 25.9 (Cyclopentyl), 21.3 (C**17**); HRMS: (ESI^+) calculated for $C_{27}H_{28}NO$ 382.2165. Found $[M+H]^+$ 382.2183.

***N*-(5-Methyl-2-(1-phenylvinyl)phenyl)acetamide (4ua)**

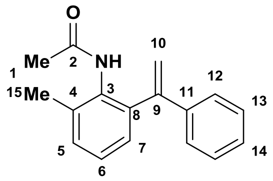

General Procedure E: The reaction was carried out with $Ir(cod)_2OTf$ (7.5 mol%), **L-4** (7.5 mol%) and 1,4-dioxane (0.5 M with respect to substrate) and was run for 72 h. Purification of the residue by FCC (1st column: hexane/EtOAc 10-40%; 2nd column: hexane/Et_2O 60-70%) afforded the title compound (20.0 mg, 56%, 0.86:0.14 mixture of rotamers *A:B*) as an off-white solid. ν_{max} / cm^{-1}: 3250 (m), 3023 (m), 1659 (s), 1521 (s), 1444 (s); *Signals for rotamer A*: ^1H NMR (400 MHz, $CDCl_3$): δ 7.36 – 7.27 (6H, m, ArCH), 7.25 – 7.20 (2H, m, ArCH), 6.41 (1H br. s, N-H), 5.73 (1H, d, *J* = 1.5 Hz, C**10**-H_2), 5.32 (d, *J* = 1.5 Hz, C**10**-H_2), 2.25 (3H, s, C**1**-H_3), 1.67 (3H, s, C**15**-H_3); ^{13}C NMR (101 MHz, $CDCl_3$): δ 168.4 (C**2**), 147.9 (C**9**), 140.5 (C**8**), 138.9 (C**11**), 136.7 (C**4**), 133.2 (C**3**), 130.8 (C**5**), 128.8 (ArCH), 128.3 (ArCH), 128.1 (ArCH), 127.4 (ArCH), 126.5 (ArCH), 116.6 (C**10**), 22.9 (C**1**), 18.7 (C**15**); HRMS: (ESI^+) calculated for $C_{17}H_{17}NONa$ 274.1202. Found $[M+Na]^+$ 274.1207; m.p. 108 - 110 °C ($CDCl_3$).

Characteristic signals for rotamer B: ^{1}H NMR (400 MHz, $CDCl_3$): δ 6.26 (1H, s, N-H), 5.69 (1H, d, *J* = 1.0 Hz, C**10**-H_2), 5.28 (1H, d, *J* = 1.0 Hz, C**10**-H_2).

***N*-(2-Methyl-6-(1-(*p*-tolyl)vinyl)phenyl)cyclopentanecarboxamide (4vb)**

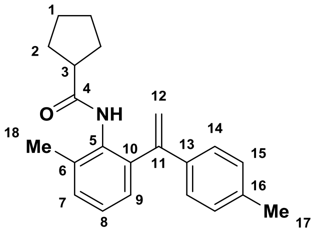

General Procedure D: The reaction was carried out with $Ir(cod)_2OTf$ (10 mol%), **L-4** (10 mol%) and 1,4-dioxane (0.5 M with respect to substrate) and was run for 96 h. The crude material was purified by FCC (hexane/EtOAC 0-10%) to afford the title compound (22.9 mg, 50%) as a yellow oil. V_{max}/ Cm^{-1} : 3263 (m), 2953 (m), 1652 (s), 1511 (s); ^{1}H NMR (400 MHz, $CDCl_3$): δ 7.23 – 7.20 (1H, m, C**7**-H), 7.20 – 7.12 (4H, m, C**8**-H, C**9**-H, C**14**-H), 7.13 – 7.03 (2H, m, C**15**-H), 6.46 (1H, s, N-H), 5.69 (1H, d, *J* = 1.5 Hz, C**12**-H_2), 5.19 (1H, d, *J* = 1.5 Hz, C**12**-H_2), 2.31 (3H, s, C**17**-H_3), 2.27 – 2.22 (1H, m, C**1**-H), 2.20 (3H, s, C**18**-H_3), 1.68 – 1.35 (8H, m, C**2**-H_2, C**3**-H_2); ^{13}C NMR (101 MHz, $CDCl_3$): δ 174.0 (C4), 147.5 (C11), 138.8 (C10), 138.0 (C16), 137.2 (C13), 136.4 (C6), 133.3 (C5), 130.6 (C7), 129.4 (C15), 128.2 (C9), 127.0 (C8), 126.3 (C14), 115.3 (C12), 45.9 (C1), 30.2 (Cyclopentyl), 25.9 (Cyclopentyl), 21.3 (C17), 18.8 (C18); HRMS: (ESI^+) calculated for $C_{22}H_{26}NO$ 320.2009. Found $[M+H]^+$ 320.2010;

***N*-((13*S*)-13-methyl-17-oxo-2-(1-phenylvinyl)-7,8,9,11,12,13,14,15,16,17-decahydro-6*H*-cyclopenta[*a*]phenanthren-3-yl)acetamide (4wa)**

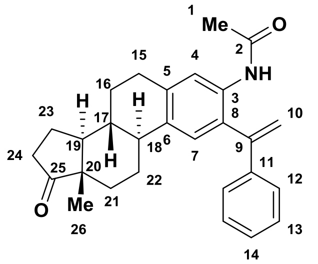

General Procedure E: The reaction was carried out with $Ir(cod)_2OTf$ (10 mol%), **L-4** (10 mol%) and 1,4-dioxane (0.5 M with respect to substrate) and was run for 72 h. Purification of the residue by FCC (hexane/EtOAc 50%) afforded the title compound (23.1 mg, 57%) as an orange oil. ν_{max} / cm^{-1} : 3313 (m), 2929 (m), 1735 (s), 1679 (s), 1514 (s); ^{1}H NMR (500 MHz, $CDCl_3$): δ 7.84 (1H, s, C**4**-H), 7.38 – 7.28 (5H, m, C**12**-H, C**13**-H, C**14**-H), 7.18 (1H, s, C**7**-H), 6.84 (1H, s, N-H), 5.81 (1H, s, C**10**-H_2), 5.33 (1H, s, C**10**-H_2), 3.02 – 2.89 (2H, m, C**16**-H_2), 2.51 (1H, dd, *J* = 19.0, 8.5 Hz, C**24**-H_2), 2.42 – 2.25 (2H, m, C**22**-H_2, C**18**-H_2), 2.20 – 2.01 (3H, m, C**15**-H_2, C**23**-H_2, C**24**-H_2), 1.94 (1H, dd, *J* = 12.0, 4.0 Hz, C**21**-H_2), 1.76 (3H, s, C**1**-H_3), 1.70 – 1.58 (3H, m, C**17**-H, C**23**-H_2), 1.56 – 1.41 (6H, m, C**15**-H_2, C**17**-H, C**19**-H, C**21**-H_2, C**22**-H_2, C**23**-H_2), 0.92 (3H, s, C**26**-H_3); ^{13}C NMR (126 MHz, $CDCl_3$): δ 221.0

(C**25**), 168.2 (C**2**), 146.8 (C**9**), 139.8 (C**11**), 137.3 (C**5**), 136.1 (C**6**), 132.9 (C**3**), 129.9 (C**8**), 128.9 (Ar<u>C</u>H), 128.6 (C**14**), 127.3 (C**7**), 126.7 (Ar<u>C</u>H), 122.4 (C**4**), 117.1 (C**10**), 50.6 (C**19**), 48.1 (C**20**), 44.3 (C**18**), 38.3 (C**17**), 36.0 (C**24**), 31.7 (C**21**), 29.6 (C**16**), 26.6 (C**15**), 25.9 (C**22**), 24.3 (C**1**), 21.7 (C**23**), 14.0 (C**26**); HRMS: (ESI^+) calculated for $C_{28}H_{32}NO_2$ 414.2428. Found $[M+H]^+$ 414.2427; $[\alpha]^{24}_D$ = + 97.5 (c = 1.0, CH_2Cl_2).

***N*-(2-(1-(*p*-Tolyl)vinyl)phenyl)cyclopentanecarboxamide (4fb)**

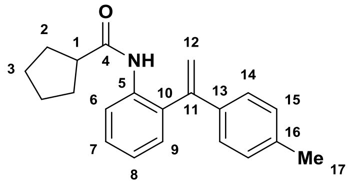

General Procedure E: The reaction was carried out with Ir(cod)$_2$OTf (7.5 mol%), **L-4** (7.5 mol%) and 1,4-dioxane (0.5 M with respect to substrate) and was run for 72 h. The crude material was purified by FCC (hexane/EtOAc 0-5%) to afford the title compound (33.5 mg, 77%, 0.93:0.07 mixture of rotamers *A:B*) as an orange oil. ν_{max} / cm^{-1}: 3418 (m), 2953 (m), 1682 (s) 1512 (s), 1445 (s); *Signals for rotamer A*: ^{1}H NMR (400 MHz, $CDCl_3$): δ 8.42 (1H, d, *J* = 8.0 Hz, C**6**-<u>H</u>), 7.52 (1H, ddd, *J* = 8.0, 8.0, 1.5 Hz, C**7**-<u>H</u>), 7.45 – 7.34 (3H, m, C**9**-<u>H</u>, C**15**-<u>H</u>), 7.32 – 7.24 (3H, m, C**8**-<u>H</u>, C**15**-<u>H</u>), 7.22 (1H, s, N-<u>H</u>), 6.01 (1H, d, *J* = 1.5 Hz, C**12**-<u>H$_2$</u>), 5.46 (1H, d, *J* = 1.5 Hz, C**12**-<u>H$_2$</u>), 2.50 (3H, s, C**17**-<u>H$_3$</u>), 2.49 – 2.41 (1H, m, C**1**-<u>H</u>), 1.84 – 1.56 (8H, m, C**2**-<u>H$_2$</u>, C**3**-<u>H$_2$</u>); ^{13}C NMR (101 MHz, $CDCl_3$): δ 174.2 (C**4**), 146.3 (C**11**), 138.7 (C**16**), 136.4 (C**13**), 135.6 (C**5**), 131.7 (C**10**), 130.4 (C**9**), 129.6 (C**15**), 128.8 (C**7**), 126.6(C**14**), 123.9 (C**8**), 121.2 (C**6**), 116.4 (C**12**), 47.0 (C**1**), 30.1 (C**2**), 25.9 (C**3**), 21.3 (C**17**); HRMS: (ESI^+) calculated for $C_{21}H_{24}NO$ 306.1852. Found $[M+H]^+$ 306.1861.

Characteristic signals for rotamer B: ^{1}H NMR (400 MHz, $CDCl_3$): δ 8.28 (1H, d, *J* = 8.5 Hz, C**6**-<u>H</u>), 5.97 (1H, s, C**12**-<u>H$_2$</u>), 5.42 (1H, s, C**12**-<u>H$_2$</u>).

***N*-(2-(1-([1,1'-Biphenyl]-4-yl)vinyl)phenyl)cyclopentanecarboxamide (4fc)**

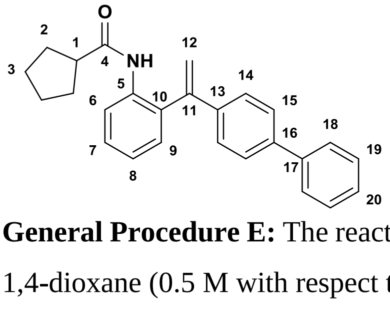

General Procedure E: The reaction was carried out with Ir(cod)$_2$OTf (7.5 mol%), **L-4** (7.5 mol%) and 1,4-dioxane (0.5 M with respect to substrate) and was run for 72 h. The crude material was purified by FCC (1st column: hexane/EtOAc 0-5%; 2nd column: hexane/EtOAc 5%) to afford the title compound (32.5 mg, 62%) as an off-white powder. V_{max} / cm^{-1}: 3415 (m), 3029 (m), 2953 (m), 1684 (s), 1515 (s); ^{1}H NMR (400 MHz, $CDCl_3$): δ 8.28 (1H, d, *J* = 8.5 Hz, C**6**-<u>H</u>), 7.65 – 7.54 (4H, m, ArC<u>H</u>), 7.52 – 7.34

(6H, m, ArCH), 7.30 (1H, dd, *J* = 7.5, 1.5 Hz, C**9**-H), 7.16 (1H, dd, *J* = 7.5, 7.5 Hz, C**8**-H), 7.07 (1H, br. s, N-H), 5.96 (1H, s, C**12**-H_2), 5.40 (1H, s, C**12**-H_2), 2.34 (1H, app. p, *J* = 7.5 Hz, C**1**-H), 1.68 – 1.36 (8H, m, C**2**-H_2, C**3**-H_2); ^{13}C NMR (101 MHz, $CDCl_3$): δ 174.2 (C**4**), 146.1 (C**11**), 141.6 (ArCH), 140.5 (ArCH), 138.1 (C**13**), 135.6 (C**5**), 131.5 (C**10**), 130.5 (C**9**), 129.0 (ArCH), 129.0 (ArCH), 127.7 (ArCH), 127.7 (ArCH), 127.1 (ArCH), 127.1 (ArCH), 124.1 (C**8**), 121.5 (C**6**), 117.3 (C**12**), 47.0 (C**1**), 30.1 (C**2**), 25.9 (C**3**); HRMS: (ESI^+) calculated for $C_{26}H_{25}NONa$ 390.1828. Found $[M+Na]^+$ 390.1829; m.p. 100 - 102 °C ($CDCl_3$).

***N*-(2-(1-(4-Fluorophenyl)vinyl)phenyl)cyclopentanecarboxamide (4fd)**

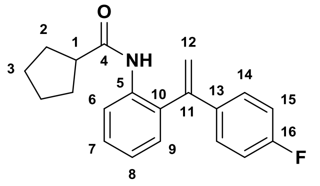

General Procedure E: The reaction was carried out with $Ir(cod)_2OTf$ (7.5 mol%), **L-4** (7.5 mol%) and 1,4-dioxane (0.5 M with respect to substrate) and was run for 72 h. The crude material was purified by FCC (2 columns; hexane/EtOAc 0-10%) to afford the title compound (33.2 mg, 75%, 0.96:0.04 mixture of rotamers *A:B*) as a yellow oil. ν_{max}/ cm^{-1}: 3062 (m), 2953 (m), 1666 (s), 1505 (s), 1506 (s); *Signals for rotamer A*: ^{1}H NMR (400 MHz, $CDCl_3$): δ 8.24 (1H, d, *J* = 8.0 Hz, C**6**-H), 7.37 (1H, ddd, *J* = 8.0, 8.0, 2.0 Hz, C**7**-H), 7.33 – 7.27 (2H, m, C**14**-H), 7.25 – 7.21 (1H, m, C**9**-H), 7.18 – 7.09 (1H, m, C**8**-H), 7.06 – 6.97 (3H, m, C**15**-H, N-H), 5.85 (1H, s, C**12**-H_2), 5.34 (1H, s, C**12**-H_2), 2.42 – 2.26 (1H, m, C**1**-H), 1.70 – 1.37 (8H, m, C**2**-H_2, C**3**-H_2); ^{13}C NMR (101 MHz, $CDCl_3$): δ 174.2 (C**4**), 163.0 (d, $^1J_{C-F}$ = 248.5 Hz, C**16**), 145.4 (C**11**), 135.5 (C**5**),135.4 (d, $^4J_{C-F}$ = 3.5 Hz, C**13**), 131.4 (C**10**), 130.3 (C**9**), 129.0 (C**7**), 128.4 (d, $^3J_{C-F}$ = 8.0 Hz, C**14**), 124.1 (C**8**), 121.6 (C**6**), 117.0 (C**12**), 115.8 (d, $^2J_{C-F}$ = 21.5 Hz, C**15**), 46.97 (C**1**), 30.10 (C**2**), 25.90 (C**3**); HRMS: (ESI^+) calculated for $C_{20}H_{21}FNO$ 310.1602. Found $[M+H]^+$ 310.1606.

Characteristic signals for rotamer B: ^{1}H NMR (400 MHz, $CDCl_3$): 8.12 (1H, d, *J* = 8.5 Hz, C**6**-H), 5.81 (1H, s, C**12**-H_2), 5.29 (1H, s, C**12**-H_2), 1.90 (1H, app. p, *J* = 7.5 Hz, C**1**-H).

***N*-(2-(1-(4-(Trifluoromethyl)phenyl)vinyl)phenyl)cyclopentanecarboxamide (4fe)**

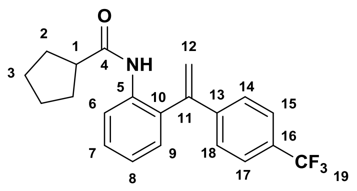

General Procedure E: The reaction was carried out with Ir(cod)$_2$OTf (10 mol%), **L-4** (10 mol%) and 1,4-dioxane (1.0 M with respect to substrate) and was run for 72 h. The crude material was purified by FCC (2 columns; hexane/EtOAc 0-10%) to afford the title compound (29.5 mg, 57%, 0.85:0.15 mixture of rotamers *A:B*) as a yellow oil. ν_{max} / cm^{-1}: 3282 (m), 2956 (m), 1658 (s), 1518 (s), 1322 (s); ^{1}H NMR (400 MHz, CDCl$_3$): δ 8.19 (1H, d, *J* = 8.0 Hz, C**6**-H, *A*+*B*), 7.58 (1.7H, d, *J* = 8.0 Hz, C**15**-H, *A*), 7.52 (0.3H, d, *J* = 8.0 Hz, C**15**-H, *B*), 7.43 (1.7H, d, *J* = 8.0 Hz, C**14**-H, *A*+*B*), 7.40 – 7.37 (0.85H, m, C**7**-H, *A*), 7.31 (0.15H, dd, *J* = 7.5, 7.5 Hz, C**7**-H, *B*), 7.24 (1H, m, C**9**-H, *A*+*B*), 7.16 (0.85H, dd, *J* = 7.5, 7.5 Hz, C**8**-H, *A*), 7.08 (0.15H, dd, *J* = 7.5, 7.5 Hz, C**8**-H, *B*), 6.91 (1H, s, N-H, *A*+*B*), 5.99 (1H, s, C**12**-H$_2$, *A*+*B*), 5.50 (1H, s, C**12**-H$_2$, *A*+*B*), 2.68 (0.15H, app. p, *J* = 8.0 Hz, C**1**-H, *B*), 2.31 (0.85H, app. p, *J* = 8.0 Hz, C**1**-H, *A*), 1.98 – 1.85 (0.85H, m, cyclopentyl, *A*), 1.84 – 1.75 (0.15H m, cypcloentyl, *B*), 1.68 – 1.42 (5.95H, m, cyclopentyl, *A*), 1.29 – 1.23 (1.05H, m, cyclopentyl, *B*); ^{13}C NMR (101 MHz, CDCl$_3$): δ 174.0 (C**4**, *A*), 173.9 (C**4**, *B*), 145.3 (C**11**, *A*), 145.27 (C**11**, *B*), 144.11 (C**13**, *B*), 142.68 (C**13**, *A*), 135.22 (C**5**, *A*+*B*), 130.95 (C**10**, *A*+*B*), 130.93 (C**16**, *A*+*B*), 130.58 (C**9**, *B*), 130.30 (C**9**, *A*), 129.14 (C**7**, *A*), 128.93 (C**7**, *B*), 126.75 (C**14**, *A*+*B*), 125.7 (q, $^3J_{C\text{-}F}$ = 3.5 Hz, C**15**, *A*+*B*), 124.3 (C**8**, *A*+*B*), 122.6 (C**17**, *A*+*B*), 122.0 (C**6**, *A*+*B*), 119.6 (C**12**, *B*), 119.2 (C**12**, *A*), 46.7 (C**1**, *A*+*B*), 30.5 (C**2**, *B*), 29.9 (C**2**, *A*), 26.0 (C**3**, *B*), 25.7 (C**3**, *A*); ^{19}F NMR (377 MHz, CDCl$_3$): δ -62.61; HRMS: (ESI$^+$) calculated for $C_{21}H_{21}F_3NO$ 360.1570. Found [M+H]$^+$ 360.1576.

***N*-(2-(1-(4-(*Tert*-butyl)phenyl)vinyl)phenyl)cyclopentanecarboxamide (4ff)**

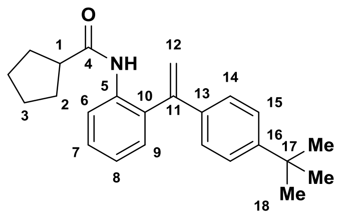

General Procedure E: The reaction was carried out with Ir(cod)$_2$OTf (7.5 mol%), **L-4** (7.5 mol%) and 1,4-dioxane (1.0 M with respect to substrate) and was run for 96 h. The crude material was purified by FCC (toluene/Et$_2$O 0-2%) to afford the title compound (39.6 mg, 80%) as a yellow oil. ν_{max} / cm^{-1}: 3415 (m), 2958 (m), 1686 (s), 1513 (s); ^{1}H NMR (400 MHz, CDCl$_3$): δ 8.26 (1H, d, *J* = 8.0 Hz, C**6**-H), 7.39 – 7.32 (3H, m, C**15**-H, C**7**-H), 7.29 – 7.24 (3H, m, C**14**-H, C**9**-H), 7.19 – 7.09 (1H, m, C**8**-H), 7.00 (1H, s, N-H), 5.85 (1H, d, *J* = 1.5 Hz, C**12**-H$_2$), 5.33 (1H, d, *J* = 1.5 Hz, C**12**-H$_2$), 2.32 – 2.23 (1H, m, C**1**-H), 1.63 – 1.51 (4H, m, C**3**-H$_2$), 1.47 – 1.40 (4H, m, C**2**-H$_2$), 1.31 (9H, s, C**18**-H$_3$); ^{13}C NMR (101 MHz, CDCl$_3$): δ 174.1 (C**4**), 152.0 (C**16**), 146.3 (C**11**), 136.3 (C**13**), 135.6 (C**5**), 131.6 (C**10**), 130.5

(C**9**), 128.9 (C**7**), 126.4 (C**14**), 125.9 (C**15**), 123.9 (C**8**), 121.2 (C**6**), 116.7 (C**12**), 47.0 (C**1**), 34.8 (C**17**), 31.4 (C**18**), 30.0 (C**3**), 25.8 (C**2**); HRMS: (ESI^+) calculated for $C_{24}H_{30}NO$ 348.2322. Found $[M+H]^+$ 348.2334.

N-(2-(1-(4-(Trimethylsilyl)phenyl)vinyl)phenyl)cyclopentanecarboxamide (4fg)

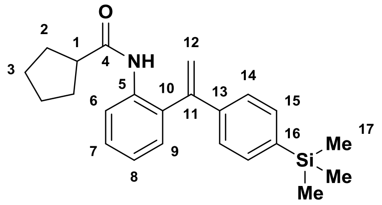

General Procedure E: The reaction was carried out with $Ir(cod)_2OTf$ (7.5 mol%), **L-4** (7.5 mol%) and 1,4-dioxane (0.5 M with respect to substrate) and was run for 96 h. The crude material was purified by FCC (toluene/Et_2O 0-2%) to afford the title compound (27.4 mg, 53%) as a yellow oil. ν_{max} / cm^{-1} : 3418 (m), 2954 (m), 1682 (s), 1516 (s); ^1H NMR (400 MHz, $CDCl_3$): δ 8.25 (1H, d, *J* = 8.0 Hz, C**6**-H), 7.52 – 7.46 (2H, m, C**15**-H), 7.41 – 7.34 (1H, m, C**7**-H), 7.34 – 7.23 (3H, m, C**9**-H, C**14**-H), 7.14 (1H, ddd, *J* = 7.5, 7.5, 1.0 Hz, C**8**-H), 6.98 (1H, s, N-H), 5.90 (1H, d, *J* = 1.5 Hz, C**12**-H_2), 5.38 (1H, d, *J* = 1.5 Hz, C**12**-H_2), 2.33 – 2.20 (1H, m, C**1**-H), 1.60 – 1.42 (8H, m, C**2**-H_2, C**3**-H_2), 0.26 (9H, s, C**17**-H_3); ^{13}C NMR (101 M Hz, $CDCl_3$): δ 174.0 (C**4**), 146.5 (C**11**), 141.2 (C**16**), 139.5 (C**13**), 135.4 (C**5**), 133.9 (C**15**), 131.3 (C**7**), 130.3 (C**9**), 128.8 (C**10**), 125.7 (C**14**), 123.9 (C**8**), 121.3 (C**6**), 117.4 (C**12**), 46.9 (C**1**), 29.8 (C**3**), 25.7 (C**2**), -1.2 (C**17**); HRMS: (ESI^+) calculated for $C_{23}H_{30}NOSi$ 364.2091. Found $[M+H]^+$ 364.2091.

N-(2-(1-(*m*-Tolyl)vinyl)phenyl)cyclopentanecarboxamide (4fh)

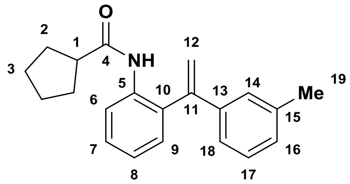

General Procedure E: The reaction was carried out with $Ir(cod)_2OTf$ (7.5 mol%), **L-4** (7.5 mol%) and 1,4-dioxane (0.5 M with respect to substrate) and was run for 72 h. The crude material was purified by FCC (2 columns; hexane/EtOAc 0-10%) to afford the title compound (30.5 mg, 70%, 0.93:0.07 mixture of rotamers *A:B*) as a yellow oil. ν_{max} / cm^{-1}: 3414 (m), 2953 (m), 1682 (s), 1515 (s), 1446 (s); *Signals for rotamer A*: ^1H NMR (400 MHz, $CDCl_3$): δ 8.26 (1H, d, *J* = 8.0 Hz, C**6**-H), 7.37 (1H, ddd, *J* = 8.0, 8.0, 1.5 Hz, C**7**-H), 7.29 – 7.18 (2H, m, C**9**-H, ArCH), 7.17 – 7.10 (4H, m, ArCH), 7.05 (1H, br. s, N-H), 5.86 (1H, s, C**12**-H_2), 5.35 (1H, s, C**12**-H_2), 2.38 – 2.25 (4H, m, C**1**-H, C**19**-H_3), 1.69 – 1.42 (8H, m, C**2**-H_2, C**3**-H_2); ^{13}C NMR (101 MHz, $CDCl_3$): δ 174.2 (C**4**), 146.7 (C**11**), 139.3 (C**13**), 138.7 (C**15**), 135.6 (C**5**), 131.7 (C**10**), 130.4 (C**9**), 129.5 (ArCH), 128.9 (C**7**), 128.9 (ArCH), 127.3 (ArCH), 124.0

(C**8**), 123.8 (Ar<u>C</u>H), 121.3 (C**6**), 117.3 (C**12**), 47.0 (C**1**), 30.0 (C**2**), 25.9 (C**3**), 21.5 (C**19**); HRMS: (ESI^+) calculated for $C_{21}H_{24}NO$ 306.1852. Found $[M+H]^+$ 306.1864.

Characteristic signals for rotamer B: ^{1}H NMR (400 MHz, $CDCl_3$): 8.14 (1H, d, *J* = 8.5 Hz, C**6**-<u>H</u>), 5.83 (1H, s, C**12**-<u>H</u>$_2$), 5.31 (1H, s, C**12**-<u>H</u>$_2$).

N-(2-(1-(3-Chlorophenyl)vinyl)phenyl)cyclopentanecarboxamide (4fi)

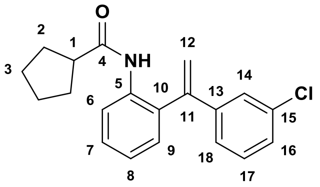

General Procedure E: The reaction was carried out with $Ir(cod)_2OTf$ (10 mol%), **L-4** (10 mol%) and 1,4-dioxane (1.0 M with respect to substrate) and was run for 72 h. The crude material was purified by FCC (hexane/EtOAc 0-5%) to afford the title compound (29.8 mg, 64%, 0.95:0.05 mixture of rotamers *A:B*) as a yellow oil. ν_{max} / cm^{-1}: 3290 (m), 3062 (m), 2954 (s), 1663 (s), 1515 (s); *Signals for rotamer A*: ^{1}H NMR (400 MHz, $CDCl_3$): δ 8.23 (1H, d, *J* = 8.0 Hz, C**6**-<u>H</u>), 7.43 – 7.34 (2H, m, C**7**-<u>H</u>, C**14**-<u>H</u>), 7.32 – 7.26 (1H, m, C**17**-<u>H</u>), 7.26 – 7.20 (2H, m, C**9**-<u>H</u>, C**16**-<u>H</u>), 7.18 – 7.10 (2H, m, C**8**-<u>H</u>, C**18**-<u>H</u>), 6.98 (1H, s, N-<u>H</u>), 5.90 (1H, s, C**12**-<u>H</u>$_2$), 5.42 (1H, s, C**12**-<u>H</u>$_2$), 2.41 – 2.28 (1H, m, C**1**-<u>H</u>), 1.72 – 1.35 (8H, m, C**2**-<u>H</u>$_2$, C**3**-<u>H</u>$_2$); ^{13}C NMR (101 MHz, $CDCl_3$): δ 174.2 (C**4**), 145.4 (C**11**), 141.3 (C**13**), 135.5 (C**5**), 135.0 (C**15**), 131.0 (C**10**), 130.4 (Ar<u>C</u>H), 130.2 (Ar<u>C</u>H), 129.2 (Ar<u>C</u>H), 128.7 (C**17**), 126.6 (Ar<u>C</u>H), 125.0 (Ar<u>C</u>H), 124.2 (C**8**), 121.8 (C**6**), 118.5 (C**12**), 47.0 (C**1**), 30.1 (C**2**), 25.9 (C**3**); HRMS: (ESI^+) calculated for $C_{20}H_{21}ClNO$ 326.1306. Found $[M+H]^+$ 326.1303.

Characteristic signals for rotamer B: ^{1}H NMR (400 MHz, $CDCl_3$): 8.37 (1H, d, *J* = 8.0 Hz, C**6**-<u>H</u>), 5.43 (1H, s, C**12**-<u>H</u>$_2$), 1.91 (1H, app. p, *J* = 7.5 Hz, C**1**-<u>H</u>).

N-(2-(1-(2-Fluorophenyl)vinyl)phenyl)cyclopentanecarboxamide (4fj)

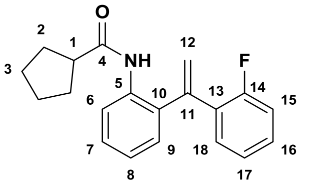

General Procedure E: The reaction was carried out with $Ir(cod)_2OTf$ (10 mol%) and **L-4** (10 mol%) and 1,4-dioxane (1.0 M with respect to substrate) and was run for 72 h. The crude material was purified by FCC (2 columns; hexane/EtOAc 0-10%) to afford the title compound (22.2 mg, 50%, 0.97:0.03 mixture of rotamers *A:B*) as an orange oil. ν_{max} / cm^{-1}: 3288 (m), 2952 (m) 1665 (s), 1514 (s), 1445 (s); *Signals for rotamer A*: ^{1}H NMR (400 MHz, $CDCl_3$): δ 8.23 (1H, d, *J* = 8.0 Hz, C**6**-<u>H</u>), 7.34 (1H, ddd, *J*

= 8.0, 8.0, 1.5 Hz, C**7**-H), 7.31 – 7.26 (1H, m, C**17**-H), 7.25 – 7.17 (2H, m, C**9**-H, N-H), 7.15 – 7.05 (4H, m, C**8**-H, C**15**-H, C**16**-H, C**18**-H), 5.95 (1H, s, C**12**-H_2), 5.61 (1H, s, C**12**-H_2), 2.42 (1H, app. p, *J* = 7.5 Hz, C**1**-H), 1.80 – 1.45 (8H, m, C**2**-H_2, C**3**-H_2); ^{13}C NMR (101 MHz, $CDCl_3$): δ 174.2 (C**4**), 160.3 (d, $^{1}J_{C-F}$ = 249.5 Hz, C**14**), 140.8 (C**11**), 135.2 (C**5**), 132.0 (C**10**), 130.4 (d, $^{4}J_{C-F}$ = 3.0 Hz, C**17**), 129.9 (d, $^{3}J_{C-F}$ = 3.0 Hz, ArCH), 129.84 (C**9**), 128.3 (C**7**), 127.7 (d, $^{2}J_{C-F}$ = 12.0 Hz, C**13**), 124.6 (d, $^{3}J_{C-F}$ = 3.5 Hz, ArCH), 124.0 (C**8**), 122.4 (d, ^{4}J = 6.5 Hz, C**12**), 121.6 (C**6**), 116.3 (d, $^{2}J_{C-F}$ = 22.5 Hz, C**15**), 47.0 (C**1**), 30.2 (C**3**), 26.0 (C**2**); ^{19}F NMR (377 MHz, $CDCl_3$): δ -114.17 (–) -114.33 (m); HRMS: (ESI$^+$) calculated for $C_{20}H_{21}FNO$ 310.1612. Found $[M+H]^+$ 310.1608.

Characteristic signals for rotamer B: ^{1}H NMR (400 MHz, $CDCl_3$): 8.36 (1H, d, *J* = 8.5 Hz, C**6**-H), 5.98 (1H, s, C**12**-H_2), 5.98 (1H, s, C**12**-H_2).

N-(2-(1-(Benzo[*b*]thiophen-5-yl)vinyl)phenyl)cyclopentanecarboxamide (4fk)

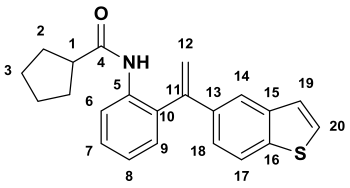

General Procedure E: The reaction was carried out with Ir(cod)$_2$OTf (7.5 mol%), **L-4** (7.5 mol%) and 1,4-dioxane (0.5 M with respect to substrate) and was run for 76 h. The crude material was purified by FCC (toluene/EtOAc 0-10%) to afford the title compound (25.3 mg, 51%) as a yellow oil. ν_{max} / cm^{-1} : 3311 (m), 2952 (m), 1678 (s), 1513 (s); ^{1}H NMR (400 MHz, $CDCl_3$): δ 8.28 (1H, d, *J* = 8.0 Hz, C**6**-H), 7.85 (1H, d, *J* = 8.5 Hz, C**17**-H), 7.68 (1H, d, *J* = 1.5 Hz, C**14**-H), 7.48 – 7.43 (1H, m, C**18**-H), 7.44 – 7.36 (2H, m, C**7**-H, C**19**-H), 7.33 – 7.27 (2H, m, C**9**-H, C**20**-H), 7.16 (1H, dd, *J* = 8.0, 1.5 Hz, C**8**-H), 7.09 (1H, s, N-H), 5.96 (1H, d, *J* = 1.0 Hz, C**12**-H_2), 5.41 (1H, d, *J* = 1.0 Hz, C**12**-H_2), 2.25 (1H, p, *J* = 7.5 Hz, C**1**-H), 1.57 – 1.31 (8H, m, C**2**-H_2, C**3**-H_2); ^{13}C NMR (101 MHz, $CDCl_3$): δ 174.2 (C**4**), 146.5 (C**11**), 140.2 (C**15**), 140.0 (C**16**), 135.7 (C**13**), 135.7 (C**5**), 131.7 (C**10**), 130.5 (C**9**), 129.0 (C**7**), 127.6 (C**18**), 124.2 (C**20**), 124.1 (C**8**), 122.9 (C**17**), 122.7 (C**19**), 122.0 (C**14**), 121.5 (C**6**), 117.2 (C**12**), 47.0 (C**1**), 30.0 (Cyclopentyl), 25.8 (Cyclopentyl); HRMS: (ESI$^+$) calculated for $C_{22}H_{22}NOS$ 348.1417. Found $[M+H]^+$ 348.1432.

Product Derivatizations

General Procedure F for quinoline synthesis:

The title compounds were prepared by adaptation of a literature procedure.[23] A 3-necked, oven-dried flask, fitted with a condensor was charged with the corresponding alkene (100 mol%) and purged with nitrogen. MeCN (0.03 M with respect to alkene) and $POCl_3$ (1000 mol%) were added and the solution was heated at reflux and stirred overnight. The reaction was cooled to ambient temperature and diluted with water (2.0 mL). Aqueous 1 M NaOH solution was added until pH 8 was reached and the resulting solution was extracted with ethyl acetate (3 × 5 mL). The organic extracts were combined, dried over Na_2SO_4 and concentrated in *vacuo*. Purification of the residues by FCC afforded the title compounds.

2-Methyl-4-phenylquinoline (5a)

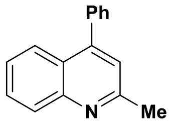

General Procedure F: The residue was purified by FCC (hexane/EtOAc 30%) to afford the title compound (91.6 mg, quantitative) as an off-white solid. ^{1}H NMR (400 MHz, $CDCl_3$): δ 8.09 (1H, d, *J* = 9.0 Hz), 7.86 (1H, dd, *J* = 9.0, 1.5 Hz,), 7.69 (1H, ddd, *J* = 8.5, 7.0, 1.5 Hz), 7.55 – 7.47 (5H, m), 7.44 (1H, ddd, *J* = 8.5, 7.0, 1.5 Hz), 7.24 (1H, s), 2.78 (3H, s); ^{13}C NMR (100 MHz, $CDCl_3$): δ 158.6, 148.7, 148.5, 138.3, 129.6, 129.5, 129.2, 128.7, 128.5, 125.9, 125.8, 125.2, 122.4, 25.5; m.p. 95 - 97 °C ($CDCl_3$) (Lit.[22] 92 - 94 °C, CH_2Cl_2/pentane). *The spectroscopic properties of this compound were consistent with the data available in the literature.* [24]

2-Cyclobutyl-4-phenylquinoline (5b)

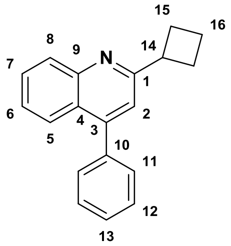

General Procedure F: The crude material was purified by FCC (EtOAc 100%) to afford the title compound (21.5 mg, 98%) as an orange oil. ν_{max} / cm^{-1}: 3059 (m), 2935 (m) 1591 (m), 1443 (m); ^{1}H NMR (400 MHz, $CDCl_3$): δ 8.14 (1H, d, *J* = 8.5 Hz, C**5**-H), 7.86 (1H, d, *J* = 8.5 Hz, C**8**-H), 7.75 – 7.65 (1H, m, C**6**-H), 7.56 – 7.48 (5H, m, C**11**-H, C**12**-H, C**13**-H), 7.46 – 7.40 (1H, m, C**7**-H), 7.35 – 7.26 (1H, m, C**2**-H), 3.91 (1H, app. p, *J* = 9.0 Hz, C**14**-H), 2.55 – 2.40 (4H, m, C**15**-H_2), 2.21 – 2.07 (1H, m, C**16**-H_2), 2.03 – 1.89 (1H, m, C**16**-H_2); ^{13}C NMR (101 MHz, $CDCl_3$): δ 164.7 (C**3**), 148.7 (C**9**), 148.4 (C**1**), 138.6 (C**10**), 129.7 (ArCH), 129.5 (C**5**), 129.3 (C**6**), 128.7 (ArCH), 128.4 (C**13**), 125.9 (C**7**), 125.7 (C**8**), 125.5 (C**4**), 120.0 (C**2**), 42.9 (C**14**), 28.5 (C**15**), 18.5 (C**16**); HRMS: (ESI$^+$) calculated for $C_{19}H_{18}N$ 260.1434. Found $[M+H]^+$ 260.1437.

2-Methyl-3-phenyl-8,9-dihydro-7*H*-cyclopenta[*h*]quinoline (5c)

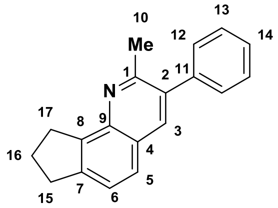

General Procedure F: The crude material was purified by FCC (hexane/EtOAc 30%) to afford the title compound (11.2 mg, 60%) as a colorless oil. ν_{max} / cm^{-1}: 3030 (m), 2573 (s) 1590 (s), 1407 (m); ^{1}H NMR (500 MHz, $CDCl_3$): δ 7.66 (1H, d, *J* = 8.5 Hz, C**5**-H), 7.54 – 7.43 (5H, m, C**12**-H, C**13**-H, C**14**-H), 7.33 (1H, d, *J* = 8.5 Hz, C**6**-H), 7.16 (1H, s, C**3**-H), 3.48 (2H, t, *J* = 7.5 Hz, C**17**-H_2), 3.13 (2H, t, *J* = 7.5 Hz, C**15**-H_2), 2.77 (3H, s, C**10**-H_3), 2.27 (2H, app. p, *J* = 7.5 Hz, C**16**-H_2); ^{13}C NMR (126 MHz, $CDCl_3$): δ 158.2 (C**1**), 148.8 (C**2**), 145.8 (C**4**), 145.3 (C**7**), 141.3 (C**3**), 139.0 (C**11**), 129.6 (ArCH), 128.5 (ArCH), 128.2 (C**14**), 124.5 (C**5**), 124.0 (C**9**), 123.0 (C**6**), 121.3 (C**3**), 34.3 (C**15**), 31.3 (C**17**), 25.8 (C**10**), 25.1 (C**16**); HRMS: (ESI$^+$) calculated for $C_{19}H_{18}N$ 260.1434. Found $[M+H]^+$ 260.1437.

2-Methyl-4-phenylbenzo[*g*]quinoline (5d)

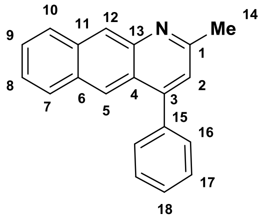

General Procedure F: The crude material was purified by FCC (hexane/EtOAc 20-30%) to afford the title compound (19.0 mg, 82%) as an orange oil. ν_{max} / cm^{-1}: 3057 (m), 2923 (m) 1596 (m), 1334 (m); ^{1}H NMR (400 MHz, $CDCl_3$): δ 8.67 (1H, s, C**12**-H), 8.40 (1H, s, C**5**-H), 8.07 (1H, d, *J* = 8.5 Hz, C**10**-H), 7.89 (1H, d, *J* = 8.5 Hz, C**7**-H), 7.64 – 7.54 (5H, m, C**16**-H, C**17**-H, C**18**-H), 7.54 – 7.38 (2H, m, C**9**-H, C**8**-H), 7.20 (1H, s, C**2**-H), 2.83 (3H, s, C**14**-H_3); ^{13}C NMR (101 MHz, $CDCl_3$): δ 159.7 (C**1**), 148.7 (C**13**), 145.0 (C**3**), 138.4 (C**15**), 133.9 (C**11**), 131.5 (C**6**), 129.7 (ArCH), 128.8 (ArCH), 128.6 (C**18**), 128.6 (C**7**), 128.3 (C**10**), 126.6 (C**12**), 126.5 (C**9**), 125.7 (C**8**), 125.3 (C**5**), 124.3 (C**4**), 122.0 (C**2**), 25.9 (C**14**); HRMS: (ESI$^+$) calculated for $C_{23}H_{16}N$ 270.1277. Found $[M+H]^+$ 270.1290.

(13a*S*)-8,13a-Dimethyl-10-phenyl-2,3,3a,3b,4,5,11b,12,13,13a-decahydro-1*H*-cyclopenta[5,6]naphtho[1,2-*g*]quinolin-1-one (5e)

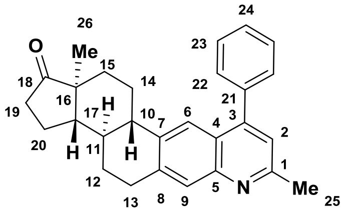

General Procedure F: The crude material was purified by FCC (EtOAc/MeOH 0-20%) to afford the title compound (13.6 mg, 61%) as a colorless solid. ν_{max} / cm^{-1} : 2928 (m), 2859 (m), 1738 (s), 1591 (s); ^{1}H NMR (400 MHz, $CDCl_3$): δ 7.82 (1H, s, C**9**-H), 7.76 (1H, s, C**6**-H), 7.60 – 7.44 (5H, m, C**22**-H, C**23**-H, C**24**-H), 7.14 (1H, s, C**2**-H), 3.23 – 3.06 (2H, m, C**13**-H_2), 2.74 (3H, s, C**25**-H_3), 2.51 (1H, dd, *J* = 18.0, 8.5 Hz, C**20**-H_2), 2.43 – 2.33 (1H, m, C**10**-H), 2.33 – 2.22 (1H, m, C**14**-H_2), 2.21 – 2.02 (3H, m, C**12**-H_2, C**19**-H_2, C**20**-H_2), 1.92 (1H, ddd, *J* = 13.0, 13.0, 3.0 Hz, C**15**-H_2), 1.73 – 1.38 (5H, m, C**11**-H, C**12**-H_2, C**14**-H_2, C**15**-H_2, C**17**-H), 0.90 (3H, s, C**26**-H_3); ^{13}C NMR (126 MHz, $CDCl_3$): δ 220.7 (C**18**), 158.0 (C**1**), 148.2 (C**3**), 146.9 (C**5**), 139.3 (C**8**), 138.9 (C**7**), 138.4 (C**21**), 129.4 (ArCH), 128.6 (ArCH), 128.3 (C**24**), 127.6 (C**9**), 123.3 (C**4**), 121.6 (C**2**), 121.3 (C**6**), 50.8 (C**17**), 47.9 (C**16**), 44.6 (C**10**), 38.1 (C**11**), 35.9 (C**20**), 31.4 (C**15**), 29.5 (C**13**), 26.5 (C**12**), 25.6 (C**14**), 25.2 (C**25**), 21.7 (C**19**), 13.8 (C**26**); HRMS: (ESI^+) calculated for $C_{28}H_{30}NO$ 397.2322. Found. $[M+H]^+$ 397.2317. m.p. 293 - 295 °C (hexane/EtOAc); $[\alpha]^{20}_D$ = + 134.7 (c = 0.20, CH_2Cl_2).

The structure of compound ***5e*** *was confirmed by single crystal X-ray diffraction of crystals obtained from* hexane/EtOAc *(Figure 2).*

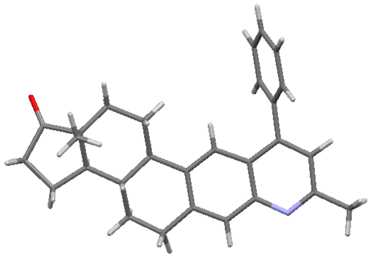

Figure 2

4-(Fluoromethyl)-2-methyl-4-phenyl-4*H*-benzo[*d*][1,3]oxazine (6)

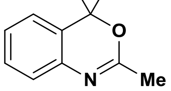

The title compound was prepared following a literature procedure.[25] An oven-dried Schlenk tube was charged with *N*-(2-(1-phenylvinyl)phenyl)acetamide **4aa** (50.0 mg, 0.21 mmol) and SelectFluor (82.0 mg, 0.23 mmol) and purged with nitrogen. Dry MeCN (2 mL) was added and the tube was sealed with a Young's tap and stirred at ambient temperature overnight. The solution was taken up in EtOAc before the solvent was removed in *vacuo*. Purification of the residue by FCC (hexane/EtOAc 10-20%) afforded the title compound (43.6 mg, 81% yield) as colorless cubes. ^{1}H NMR (400 MHz, $CDCl_3$): δ 7.42 – 7.31

(6H, m), 7.31 – 7.21 (2H, m), 7.22 – 7.10 (1H, m), 4.92 (2H, d, J = 47.0 Hz), 2.24 (3H, s); ^{13}C NMR (101 MHz, $CDCl_3$): δ 159.6, 139.5, 139.2, 139.2 (d, $^3J_{C-F}$ = 3.0 Hz), 129.7, 129.0, 128.7, 126.6, 126.4, 125.1, 124.8, 123.5, 84.4 (d, $^1J_{C-F}$ = 186.5 Hz), 82.2 (d, $^2J_{C-F}$ = 18.5 Hz), 21.9; ^{19}F NMR (377 MHz, $CDCl_3$): δ -219.4 (t, $^1J_{F-H}$ = 47.5 Hz); m.p. 86 - 88 °C ($CDCl_3$). *The spectroscopic properties for this compound were consistent with the data available in the literature.*[25]

1-(8-(Iodomethyl)-8-phenyl-7-azabicyclo[4.2.0]octa-1,3,5-trien-7-yl)ethan-1-one (7)

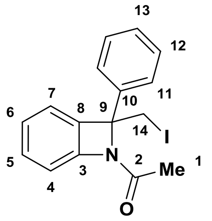

The title compound was prepared following a literature procedure.[26] To a solution of *N*-(2-(1-phenylvinyl)phenyl)acetamide **4aa** (55.0 mg, 0.23 mmol) and $NaHCO_3$ (58.0 mg, 0.69 mmol) in dry MeCN (3 mL) under nitrogen at 0 °C was added I_2 (176 mg, 0.70 mmol) portionwise over 15 minutes. The resulting solution was stirred overnight before being quenched with a 10% aqueous solution of $Na_2S_2O_3$ until the solution turned colorless. The solution was concentrated in *vacuo* before being extracted with diethyl ether (3 × 10 mL). The organic extracts were combined, dried and concentrated in *vacuo* to afford the title compound (36.8 mg, 44%) as an orange oil. ν_{max} / cm^{-1}: 3029 (m), 2925 (m) 1645 (m), 1257 (m); ^{1}H NMR (400 MHz, $CDCl_3$): δ 7.39 – 7.29 (6H, m, C**11**-H, C**12**-H, 2 × ArCH), 7.24 – 7.16 (2H, m, 2× ArCH), 7.05 – 6.99 (1H, m, C**7**-H), 3.90 (2H, s, C**14**-H_2), 2.25 (3H, s, C**1**-H_3); ^{13}C NMR (126 MHz, $CDCl_3$): δ 159.6 (C**2**), 140.6 (C**10**), 138.9 (C**3**), 129.7 (C**5**), 128.7 (ArCH), 128.6 (ArCH), 126.5 (C**6**), 126.2 (ArCH), 126.0 (C**8**), 124.9 (ArCH), 124.8 (C**7**), 81.6 (C**9**), 22.1 (C**14**), 13.5 (C1); HRMS: (ESI$^+$) calculated for $C_{16}H_{15}INO$ 364.0193. Found $[M+H]^+$ 364.0205.

2-(1-Phenylvinyl)aniline (8)

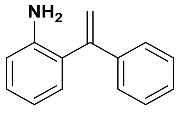

To a reaction tube was added **4ha** (50 mg, 0.188 mmol), aq. HCl (3 M, 14 mL) and dioxane (0.8 mL). The reaction tube was sealed and heated at reflux for 3 h. The reaction was cooled to ambient temperature before being quenched by the addition of saturated aq. sodium bicarbonate (10 mL) and extracted with Et_2O (3 × 10 mL). The organic extracts were combined, dried over Na_2SO_4 and concentrated in *vacuo*. The residue was purified by FCC (Hexane/Et_2O 50%) to afford the title compound (34.6 mg, 99%) as a colourless solid. ^{1}H NMR (400 MHz, $CDCl_3$): δ 7.40 – 7.36 (2H, m), 7.36 – 7.29 (3H, m), 7.17 (1H, ddd, J = 8.0, 7.5, 1.5 Hz), 7.12 (1H, dd, J = 8.0, 1.5 Hz), 6.80 (1H, td, J

= 7.5, 1.0 Hz), 6.70 (1H, dd, J = 8.0, 1.0 Hz), 5.81 (1H, d, J = 1.5 Hz), 5.37 (1H, d, J = 1.5 Hz), 3.56 (2H, s). ^{13}C NMR (101 MHz, $CDCl_3$) δ 147.3, 144.1, 139.8, 131.0, 128.9, 128.7, 128.2, 127.5, 126.8, 118.5, 116.3, 115.7, m.p. = 74-75 °C ($CDCl_3$) (Lit.[27] 80-82 °C. *no recrystallization solvent specified*); *The spectroscopic proprieties were consistent with the data available in literature.*[27]

Phenylcinnoline (9)

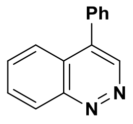

The title compound was prepared following a literature procedure.[28] To a Schlenk tube was added **8** **(**100 mg, 0.512 mmol) and aq. HCl (2 M, 1.4 mL) under nitrogen and the reaction was cooled to -5 °C. $NaNO_2$ (88.3 mg, 1.28 mmol) was added and the solution was stirred for 15 mins. Aq. H_3PO_2 (50%, 1 mL) was added and the resulting solution was stirred for 3 h at -5 °C before the reaction was warmed to ambient temperature and stirred for a further 3 h. The reaction was quenched by the addition of water (15 mL) and extracted with Et_2O (3 × 15 mL). The organic extracts were combined, dried over Na_2SO_4 and concentrated in *vacuo*. The residue was purified by FCC (hexane/Et_2O 50%) to afford the title compound (106 mg, quantitative) as a yellow oil. ^{1}H NMR (400 MHz, $CDCl_3$): δ 9.25 (1H, s), 8.59 (1H, dd, J = 8.5, 1.5 Hz), 7.98 (1H, d, J = 8.5 Hz,), 7.84 (1H, ddd, J = 8.5, 7.0, 1.5 Hz), 7.71 (1H, ddd, J = 8.5, 7.0, 1.5 Hz), 7.60 – 7.49 (5H, m); ^{13}C NMR (101 MHz, $CDCl_3$): δ 150.6, 144.6, 135.2, 134.3, 131.3, 130.5, 130.2, 129.9, 129.3, 129.1, 124.7, 124.5. *The spectroscopic properties for this compound were consistent with the data available in the literature.*[29]

2-Methyl-2,4-diphenyl-1,2-dihydroquinoline (10)

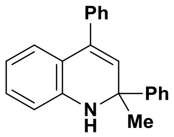

The title compound was prepared following a modified literature procedure.[30] To a resealable Schlenk tube was added **8** **(**100 mg, 0.512 mmol), TsOH (5.2 mg, 0.027 mmol), 2-acetophenone (78 μL, 0.666 mmol), Na_2SO_4 (145 mg, 1.02 mmol) and toluene (0.8 mL) under nitrogen. The tube was sealed and heated to 110 °C for 24 h. The solution was cooled to ambient temperature and quenched by the addition of water (15 mL) and extracted with Et_2O (3 × 15 mL). The organic extracts were combined, dried over Na_2SO_4 and concentrated in *vacuo*. Purification of the residue by FCC (toluene/hexane 10%) afforded the title compound (148 mg, 97%) as colourless plates. ^{1}H NMR (400 MHz, $CDCl_3$): δ 7.57 (2H, dd, J = 8.0, 1.5 Hz), 7.46 – 7.31 (7H, m), 7.30 – 7.18 (1H, m), 7.04 (1H, ddd, J = 8.0, 1.5, 1.5 Hz), 6.91 (1H, dd, J = 8.0, 1.5 Hz,), 6.61 – 6.52 (2H, m), 5.64 (1H, s), 4.21 (1H, br. s), 1.78 (3H, s); ^{13}C NMR (101

MHz, $CDCl_3$): δ 148.6, 142.9, 139.5, 135.9, 129.2, 129.1, 129.1, 128.5, 128.3, 127.5, 127.0, 126.3, 125.5, 120.6, 117.6, 113.6, 57.3, 30.2; m.p. = 114-116 °C ($CDCl_3$) Lit.[28] 113-115 °C (*no recrystallization solvent specified*). *The spectroscopic properties for this compound were consistent with the data available in the literature.*[30]

_N,N_-Diisopropyl-1*H*-pyrrole-1-carboxamide (11a)

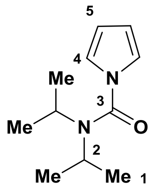

The title compound was prepared following a literature procedure.[31] A flame-dried round-bottom flask was charged with NaH (60% in mineral oil, 715 mg, 17.9 mmol), suspended in THF (15 mL) under nitrogen. The suspension was cooled to 0 °C and a solution of pyrrole (1.03 mL, 14.9 mmol) in THF (18 mL) was added dropwise over 10 minutes. The solution was stirred at 0 °C for 1 h, followed by dropwise addition of *N,N*-diisopropylcarbamoyl chloride (2.68 g, 16.4 mmol) in THF (20 mL) over 10 minutes. The solution was then warmed to ambient temperature and stirred overnight. The reaction was quenched by the addition of saturated aqueous ammonium chloride solution (50 mL) and extracted with CH_2Cl_2 (3 × 50 mL). The organic extracts were combined, washed with brine (50 mL), dried over Na_2SO_4, filtered and concentrated in *vacuo*. The crude material was purified by FCC (hexane/EtOAc 20%) to afford the title compound (2.46 g, quantitative) as colorless needles. ν_{max} / cm^{-1}: 2971 (m), 2934 (m), 1679 (s), 1430 (s), 1332 (s), 1318 (s); ^1H NMR (400 MHz, $CDCl_3$): δ 7.00 – 6.95 (2H, m, C**4**-H), 6.24 – 6.19 (2H, m, C**5**-H), 3.83 (2H, sept, *J* = 6.5 Hz, C**2**-H), 1.37 (12H, d, *J* = 6.5 Hz, C**1**-H_3); ^{13}C NMR (101 MHz, $CDCl_3$): δ 152.8 (C**3**), 120.2 (C**4**), 110.1 (C**5**), 48.7 (C**2**), 21.2 (C**1**); HRMS: (ESI^+) calculated for $C_{11}H_{19}N_2O$ 195.1492. Found $[M+H]^+$ 195.1492; m.p. 73 - 75 °C ($CDCl_3$).

3-Bromo-*N,N*-diisopropyl-1*H*-pyrrole-1-carboxamide

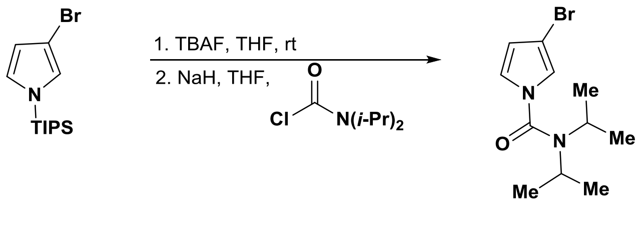

To a flame-dried flask was added 3-bromo-1-(triisopropylsilyl)-1*H*-pyrrole[32] (1.00 g, 3.31 mmol) in THF (10 mL) under nitrogen. TBAF (1M in THF, 3.31 mL) was added dropwise and the resulting solution was stirred at ambient temperature for 30 minutes. The reaction mixture was diluted with Et_2O

(20 mL) and washed with water (10 mL) and brine (10 mL). The organic extract was dried over Na_2SO_4 and concentrated in *vacuo*. The resulting residue was used without further purification.

To a suspension of NaH (60% in oil, 159 mg, 3.97 mmol) in dry THF (3 mL) at 0 °C was added the above residue in dry THF (3.7 mL) dropwise over 10 minutes. The resulting solution was stirred at 0 °C for 2 h before diisopropyl carbomoyl chloride (596 mg, 3.64 mmol) in THF (3.0 mL) was added dropwise over 10 minutes. After stirring for 15 minutes the solution was warmed to ambient temperature and stirred for a further 1 h. The reaction was quenched by the addition of water (10 mL) and extracted with Et_2O (3 × 10 mL). The organic extracts were combined, dried over Na_2SO_4 and concentrated in *vacuo*. The residue was purified by FCC (toluene/CH_2CL_2 15%) to afford the title product (550 mg, 61%) as a colourless oil. V_{max}/ Cm^{-1} : 2971 (m), 1685 (m), 1430 (s), 1321 (s); ^{1}H NMR (400 MHz, $CDCl_3$): δ 6.95 (1H, dd, *J* = 2.5, 1.5 Hz, C**7**-H), 6.90 (1H, dd, *J* = 3.0, 2.5 Hz, C**4**-H), 6.22 (1H, dd, *J* = 3.0, 1.5 Hz, C**5**-H), 3.81 (1H, p, *J* = 6.5 Hz, C**2**-H), 1.36 (12H, d, *J* = 6.5 Hz, C**1**-H_3); ^{13}C NMR (101 MHz, $CDCl_3$): δ 151.5 (C**3**), 120.9 (C**4**), 119.5 (C**7**), 112.9 (C**5**), 98.7 (C**6**), 48.8 (C**2**), 21.1 (C**1**); HRMS: (ESI$^+$) calculated for $C_{11}H_{18}N_2OBr$ 273.0597. Found $[M+H]^+$ 273.0602.

***N,N*-Diisopropyl-3-phenyl-1*H*-pyrrole-1-carboxamide (11b)**

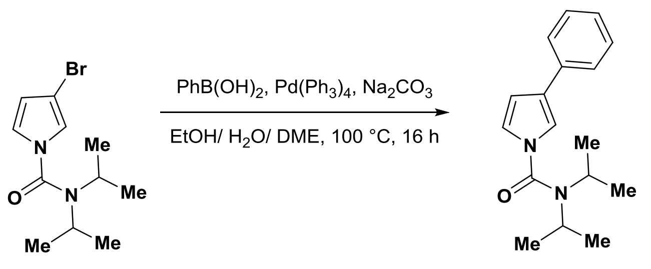

11b

An oven-dried re-sealable tube, fitted with a magnetic stirrer bar, was charged with 3-bromo-*N,N*-diisopropyl-1*H*-pyrrole-1-carboxamide (100 mg, 0.366 mmol), phenylboronic acid (78.0 mg, 0.640 mmol), Na_2CO_3 (77.6 mg, 0.732 mmol) and $Pd(PPh_3)_4$ (21.1 mg, 0.018 mmol). The tube was fitted with a rubber septum and purged with nitrogen. EtOH/H_2O/DME (0.73:0.73:2.20 mL) was added and the tube was fitted with a Young's tap. The reaction mixture was then heated to 100 °C for 16 h before being cooled to ambient temperature and quenched with water (5 mL). The mixture was extracted with Et_2O (3 × 5 mL) and the organic extracts were combined, dried over Na_2SO_4 and concentrated in *vacuo*. The residue was purified by FCC (hexane/EtOAc 10%) to afford the title compound (56 mg, 57%) as a colourless oil. V_{max}/ Cm^{-1} : 2970 (m), 2981 (m), 1683 (s), 1431 (s); ^{1}H NMR (400 MHz, $CDCl_3$): δ 7.58 – 7.48 (2H, m, C**9**-H), 7.35 (2H, dd, *J* = 8.5, 7.0 Hz, C**10**-H), 7.27 (1H, dd, *J* = 2.0, 2.0 Hz, C**4**-H), 7.24 – 7.16 (1H, m, C**11**-H), 7.00 (1H, dd, *J* = 3.0, 2.0 Hz, C**7**-H), 6.54 (1H, dd, *J* = 3.0, 2.0 Hz, C**5**-H), 3.88 (2H, hept, *J* = 6.5 Hz, C**2**-H), 1.39 (12H, d, *J* = 6.5 Hz, C**1**-H_3); ^{13}C NMR (101 MHz, $CDCl_3$): δ 152.4

(C**3**), 134.7 (C**8**), 128.7 (C**10**), 126.7 (C**6**), 126.7 (C**15**), 125.4 (C**11**), 121.0 (C**7**), 116.3 (C**4**), 108.5 (C**5**), 48.6 (C**2**), 21.1 (C**1**); HRMS: (ESI$^+$) calculated for $C_{17}H_{22}N_2ONa$ 293.1624. Found $[M+Na]^+$ 293.1634.

<u>General Procedure G</u> for branch-selective Heck-like reaction on pyrrole substrates:

An oven-dried re-sealable tube, fitted with a magnetic stirrer, was charged with pyrrole substrate (0.1 mmol, 100 mol%), $[Ir(cod)_2]OTf$ (7.5 - 10 mol%) and (*S,S*)-*f*-Binaphane (7.5 - 10 mol%). The tube was fitted with a rubber septum and purged with nitrogen. Styrene derivative (400 mol%) in anhydrous MeCN (1.5 M concentration with respect to substrate) were added and the tube was fitted with a Young's tap. The reaction mixture was then heated at 130 °C for 48-72 h, before being cooled to ambient temperature and concentrated in *vacuo*. Purification of the residues by FCC afforded the pure products.

***N,N*-Diisopropyl-2-(1-phenylvinyl)-1*H*-pyrrole-1-carboxamide (12a)**

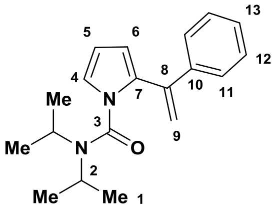

General Procedure G: The reaction was carried out with *N,N*-diisopropyl-1*H*-pyrrole-1-carboxamide **11a** (0.143 mmol), $[Ir(cod)_2]OTf$ (7.5 mol%) and (*S,S*)-f-Binaphane) (7.5 mol%) and was run for 48 h. The crude material was purified by FCC (hexane/EtOAc 0-4%) to afford the title compound (31.2 mg, 74%) as a colorless oil. ν_{max} / cm^{-1}: 2969 (m), 2933 (m), 1687 (s) 1432 (s), 1324 (s); ^{1}H NMR (400 MHz, $CDCl_3$): δ 7.43 – 7.35 (2H, m, C**11**-<u>H</u>), 7.34 – 7.27 (3H, m, C**12**-<u>H</u>, C**13**-<u>H</u>), 6.84 – 6.77 (1H, m, C**4**-<u>H</u>), 6.22 – 6.17 (2H, m, C**5**-<u>H</u>, C**6**-<u>H</u>), 5.40 (1H, d, *J* = 1.0 Hz, C**9**-<u>H</u>), 5.34 (d, *J* = 1.0 Hz, 1H, C**9**-<u>H</u>), 3.56 – 3.39 (2H, m, C**2**-<u>H</u>), 1.22 – 1.10 (12H, m, C**1**-<u>H$_3$</u>); ^{13}C NMR (101 MHz, $CDCl_3$): δ 152.0 (C**3**), 141.3 (C**10**), 141.0 (C**8**), 134.1 (C**7**), 128.2 (C**12**), 128.1 (C**11**), 127.9 (C**13**), 121.1 (C**4**), 113.9 (C**9**), 112.2 (Ar<u>C</u>H), 109.2 Ar<u>C</u>H), 48.4 (C**2**), 20.5 (C**1**); HRMS: (ESI+) calculated for $C_{19}H_{25}N_2O$ 297.1961. Found [M+H]+ 297.1967.

2-(1-(4-(*Tert*-butyl)phenyl)vinyl)-*N,N*-diisopropyl-1*H*-pyrrole-1-carboxamide (12b)

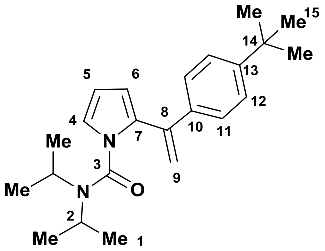

General Procedure G: The reaction was carried out with *N,N*-diisopropyl-1*H*-pyrrole-1-carboxamide **11a** (0.1 mmol), [Ir(cod)$_2$]OTf (10 mol%) and (*S,S*)-f-Binaphane) (10 mol%) and was run for 72 h. The crude material was purified by FCC (hexane/EtOAc 0-3%) to afford the title compound (25.4 mg, 72%) as an orange oil. V_{max}/ Cm^{-1} : 2964 (m), 2932 (m), 1689 (s), 1325 (s); ^{1}H NMR (400 MHz, $CDCl_3$): δ 7.36 – 7.28 (4H, m, C**11**-H, C**12**-H), 6.80 (1H, dd, *J* = 3.0, 1.5 Hz, C**4**-H), 6.26 – 6.10 (2H, m, C**5**-H, C**6**-H), 5.37 (1H, d, *J* = 1.5 Hz, C**9**-H_2), 5.33 (1H, d, *J* = 1.5 Hz, C**9**-H_2), 3.48 (2H, br. s, C**2**-H), 1.31 (9H, s, C**15**-H_3), 1.17 – 1.05 (12H, m, C**1**-H_3); ^{13}C NMR (101 MHz, $CDCl_3$): δ 152.0 (C**3**), 150.8 (C**13**), 140.9 (C**8**), 138.3 (C**10**), 134.4 (C**7**), 127.7 (C**11**), 125.2 (C**12**), 120.9 (C**4**), 113.5 (C**9**), 112.1 (C**5**), 109.2 (C**6**), 48.2 (C**2**), 34.7 (C**14**), 31.5 (C**15**), 20.5 (C**1**); HRMS: (ESI^+) calculated for $C_{23}H_{32}N_2ONa$ 375.2407. Found $[M+Na]^+$ 375.2398.

2-(1-(4-Fluorophenyl)vinyl)-*N,N*-diisopropyl-1*H*-pyrrole-1-carboxamide (12c)

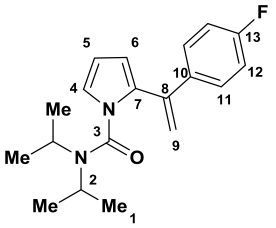

General Procedure G: The reaction was carried out with *N,N*-diisopropyl-1*H*-pyrrole-1-carboxamide **11a** (0.1 mmol), [Ir(cod)$_2$]OTf (10 mol%) and (*S,S*)-f-Binaphane) (10 mol%) and was run for 72 h. The crude material was purified by FCC (hexane/EtOAc 0-4%) to afford the title compound (19.6 mg, 62%) as an orange oil. V_{max}/ Cm^{-1} : 2969 (m), 2931 (m), 1688 (s), 1507 (s); ^{1}H NMR (400 MHz, $CDCl_3$): δ 7.39 – 7.32 (2H, m, C**11**-H), 6.99 (2H, td, *J* = 8.5, 1.5 Hz, C**12**-H), 6.84 – 6.77 (1H, m, C**4**-H), 6.21 – 6.18 (1H, m, C**5**-H), 6.18 – 6.13 (1H, m, C**6**-H), 5.36 (1H, s, C**9**-H_2), 5.29 (1H, s, C**9**-H_2), 3.54 – 3.41 (2H, m, C**2**-H), 1.17 (12H, d, *J* = 6.5 Hz, C**1**-H_3); ^{13}C NMR (101 MHz, $CDCl_3$) δ 162.70 (d, $^1J_{C-F}$ = 246.5 Hz, C**13**), 152.0 (C**3**), 140.0 (C**8**), 137.4 (C**10**), 133.9 (C**7**), 129.70 (d, $^3J_{C-F}$ = 8.0 Hz, C11), 121.2 (C**4**), 115.05 (d, $^2J_{C-F}$ = 21.5 Hz, C**12**), 113.8 (C**9**), 112.3 (C**6**), 109.3 (C**5**), 48.6 (C**2**), 20.5 (C**1**); HRMS: (ESI^+) calculated for $C_{19}H_{23}N_2OFNa$ 337.1687. Found $[M+Na]^+$ 337.1702.

***N,N*-Diisopropyl-3-phenyl-2-(1-phenylvinyl)-1*H*-pyrrole-1-carboxamide (12d)**

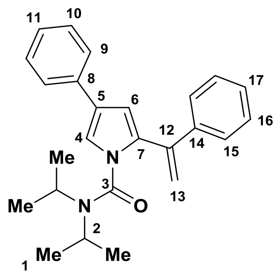

General Procedure G: The reaction was carried out with *N*,*N*-diisopropyl-3-phenyl-1*H*-pyrrole-1-carboxamide **11b** (0.1 mmol), [Ir(cod)$_2$]OTf (10 mol%) and (*S*,*S*)-f-Binaphane) (10 mol%) and was run for 72 h. The crude material was purified by FCC (1st column, $AgNO_3$ treated silica[33]: toluene/CH_2Cl_2/Et_2O 75:25:1; 2nd column: hexane/Et_2O 10%) to afford the title compound (18.5 mg, 50%) as an orange oil. V_{max}/ Cm^{-1} : 2956 (m), 2928 (m), 1687 (s), 1432 (s); ^{1}H NMR (400 MHz, $CDCl_3$) δ 7.53 – 7.48 (2H, m, C**9**-H), 7.45 – 7.40 (2H, m, C**10**-H), 7.37 – 7.30 (5H, m, C**15**-H, C**16**-H, C**17**-H), 7.20 (1H, t, *J* = 7.5 Hz, C**11**-H), 7.10 (1H, d, *J* = 2.0 Hz, C**4**-H), 6.52 (1H, d, *J* = 2.0 Hz, C**6**-H), 5.46 (1H, s, C**13**-H_2), 5.40 (1H, s, C**13**-H_2), 3.60 – 3.51 (2H, m, C**2**-H), 1.19 (12H, d, *J* = 6.5 Hz, C**1**-H_3); ^{13}C NMR (101 MHz, $CDCl_3$): δ 151.6 (C**3**), 140.9 (C**14**), 140.7 (C**8**), 134.8 (C**7**), 134.6 (C**12**), 128.7 (C**15**/**16**), 128.2 (C**15**/**16**), 128.0 (C**10**), 127.9 (C**17**), 126.1 (C**11**), 125.3 (C**5**), 125.3 (C**9**), 117.2 (C**4**), 114.2 (C**13**), 110.2 (C**6**), 30.3 (C**2**), 29.7 (C**2**), 20.4 (C**1**); HRMS: (ESI^+) calculated for $C_{25}H_{29}N_2O$ 373.2274. Found $[M+H]^+$ 373.2293.

N-(3,4-Dihydronaphthalen-2-yl)acetamide (13)

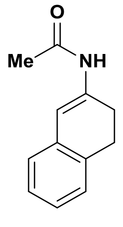

A flame-dried round-bottomed flask equipped with a Dean-Stark apparatus was charged with *β*-tetralone (820 µL, 6.20 mmol), acetamide (916 mg, 15.5 mmol), *p*-toluenesulfonic acid monohydrate (118 mg, 0.62 mmol) and toluene (40 mL). The mixture was heated at reflux for 16 h under a nitrogen atmosphere. The reaction was cooled to ambient temperature, quenched with saturated aq. $NaHCO_3$ (100 mL) and then extracted with EtOAc (3 × 30 mL). The organic extracts were combined, washed with brine (20 mL), dried over Na_2SO_4 and concentrated *in vacuo* to provide the crude material. Purification of the residue by FCC (hexane/EtOAc 50%) afforded the title compound (1.12 g, 97% yield) as an off-white solid. ^{1}H NMR ($CDCl_3$, 400 MHz): δ 7.16 – 6.95 (5H, m), 6.81 (1H, br. s), 2.87 (2H, t, *J* = 8.0 Hz), 2.44 (2H, t, *J* = 8.0 Hz), 2.10 (3H, s); 13C NMR ($CDCl_3$, 100 MHz): δ 168.5, 134.9, 134.6, 132.6, 127.0, 126.7, 126.1, 125.7, 111.3, 27.9, 27.5, 24.7; m.p. = 98 - 100 ˚C (hexane/EtOAc)

(Lit.[26] 99-101 ˚C, *no recrystallization solvent specified*). *The spectroscopic properties were consistent with the data available in the literature.*[34]

Tandem Dehydrogenation/ C-H Arylation Process Depicted in Scheme 3B

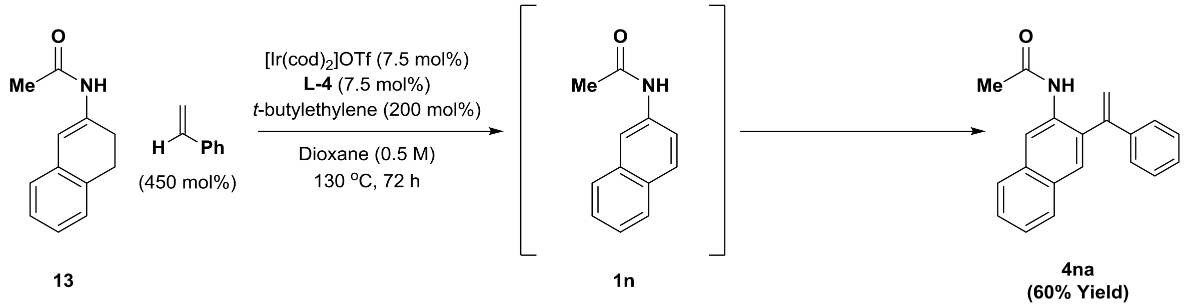

General Procedure E: The reaction was carried out with Ir(cod)$_2$OTf (7.5 mol%), **L-4** (7.5 mol%) and 1,4-dioxane (0.5 M with respect to substrate) and was run for 72 h. The crude material was purified by FCC (1st column: hexane/EtOAc 30-50%; 2nd column: hexane/EtOAc 10-30%) to afford the title compound (24.6 mg, 60%) as a brown solid. Data for **4na** is the same as described above.

Deuterium Labelling Experiments

Preparation of alkene [4-(vinyl-*β,β-d*$_2$)-1,1'-biphenyl] *(deuterio*-2c)

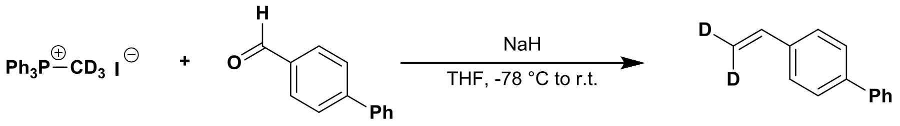

To a suspension of methyl-*d*$_3$-triphenylphosphonium iodide (2.69 g, 6.60 mmol) and biphenyl-4-carboxaldehyde (1.00 g, 5.50 mmol) in anhydrous THF (25 mL) was added portionwise NaH (581 mg, 24.2 mmol, 60% in mineral oil) at 0 ˚C. The reaction was slowly warmed to ambient temperature and stirred for 16 h. The mixture was diluted with CH_2Cl_2 (50 mL), washed with brine (3 × 10 mL), dried over Na_2SO_4, filtered and concentrated *in vacuo*. The crude material was purified by FCC (hexane/EtOAc 0-10%) to provide *deuterio*-**2c** (1.00 g, quantitative yield, 89% deuteration) as a colorless solid. ^{1}H NMR ($CDCl_3$, 400 MHz): δ 7.74 – 7.56 (4H, m), 7.56 – 7.41 (4H, m), 7.42 – 7.31 (1H, m), 6.78 (1H, br. s); ^{2}H NMR (CH_2Cl_2, 77 MHz): δ 5.81 (1D, br. s), 5.27 (1D, s); m.p. = 121 - 123 ˚C [hexane/CH_2Cl_2] (Lit.[27] 122-125 °C, *no recrystallization solvent specified*). *The spectroscopic properties of this compound were consistent with the data available in the literature.*[35]

Deuterium Labelling Experiment of 1q (Scheme 4)

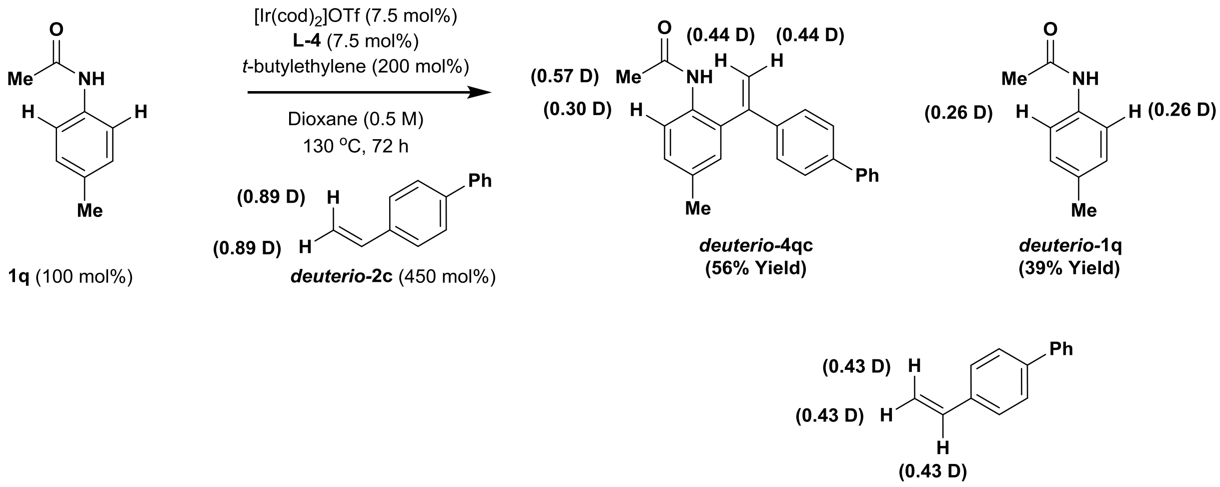

An oven-dried re-sealable tube, fitted with a magnetic stirrer, was charged with substrate **1q** (21.3 mg, 0.143 mmol) [Ir(cod)$_2$]OTf (7.5 mol%) and **L-4** (7.5 mol%). The tube was fitted with a rubber septum and purged with nitrogen. *Deuterio*-**2c** (0.644 mmol) and *t*-butylethylene (200 mol%) in anhydrous 1,4-dioxane (0.5 M concentration with respect to substrate) was added and the tube was fitted with a Young's tap. The reaction mixture was then heated to 130 °C for 72 h, before being cooled to ambient temperature and concentrated in *vacuo*. Purification of the residues by FCC (toluene/EtOAc 0-10%) afforded the *deuterio*-products.

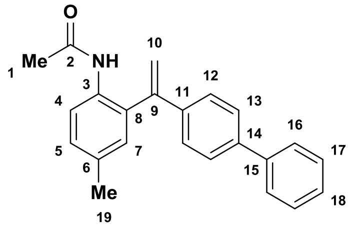

For comparison the non-deuterated product was synthesized by **General Procedure E**. *N-(2-(1-([1,1'-Biphenyl]-4-yl)vinyl)-4-methylphenyl)acetamide (**4qc**):* The reaction was carried out with Ir(cod)$_2$]OTf (7.5 mol%), **L-4** (7.5 mol%) and 1,4-dioxane (0.5 M with respect to substrate) and was run for 96 h. The crude material was purified by FCC (toluene/EtOAc 0-10%) afforded the title compound (23.1 mg, 49%) as colorless cubes. ν_{max} / cm^{-1} : 3283 (m), 3029 (m), 2923 (s), 1656 (s), 1513 (s); ^{1}H NMR (400 MHz, CDCl$_3$): δ 8.02 (1H, d, *J* = 8.5 Hz, C**4**-H), 7.50 – 7.45 (4H, m, C**13**-H, C**16**-H), 7.45 (2H, dd *J* = 7.5, 7.5 Hz, C**17**-H), 7.42 – 7.33 (3H, m, C**12**-H, C**18**-H), 7.20 (1H, dd, *J* = 8.5, 2.0 Hz, C**5**-H), 7.10 (1H, d, *J* = 2.0 Hz, C**7**-H), 6.95 (1H, s, N-H), 5.91 (1H, s, C**10**-H), 5.37 (1H, s, C**10**-H), 2.35 (3H, s, C**19**-H$_3$), 1.81 (3H, s, C**1**-H$_3$); ^{13}C NMR (101 MHz, CDCl$_3$): δ 168.2 (C**2**), 146.1 (C**9**), 141.3 (C**14**), 140.4 (C**15**), 138.5 (C**11**), 134.2 (C**6**), 132.8 (C**3**), 132.1 (C**8**), 130.8 (C**7**), 129.5 (C**5**), 129.0 (C**17**), 127.7 (C**18**), 127.5 (C**13**), 127.1 (C**12**, C**16)**, 122.2 (C**4**), 117.0 (C**10**), 24.4 (C**1**), 21.0 (C**19**); HRMS: (ESI$^+$) calculated for $C_{23}H_{22}NO$ 328.1696. Found [M+H]$^+$ 328.1689; m.p. 152 - 154 °C (CDCl$_3$).
The data for the deuterated products is presented below:

Deuterio-4qc

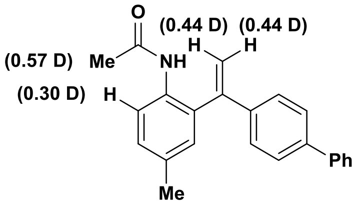

^{1}H NMR (400 MHz, CDCl$_3$): δ 8.02 (0.7H, d, *J* = 8.5 Hz), 7.62 – 7.53 (4H, m), 7.49 – 7.33 (5H, m), 7.24 – 7.16 (1H, m), 7.10 (1H, d, *J* = 2.0 Hz), 6.95 (1H, s), 5.91 (0.56H, d, *J* = 6.0 Hz), 5.37 (0.56H, d, *J* = 6.0 Hz), 2.36 (3H, s), 1.82 (2.43H, s); ^{2}H NMR (77 MHz, CHCl$_3$): δ 8.07 (0.3D, s), 5.94 (0.44D, s), 5.40 (0.44D, s), 1.81 (0.57D, d, *J* = 2.5 Hz). *Deuterium incorporation was calculated by integration of both ^{1}H NMR and ^{2}H NMR signals.*

Deuterio-1q

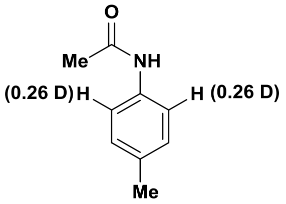

^{1}H NMR (400 MHz, $CDCl_3$): δ 7.36 (1.48H, dd, *J* = 8.5, 6.0 Hz), 7.22 – 7.16 (1H, m), 7.16 – 7.06 (2H, m), 2.31 (3H, s), 2.15 (3H, s); ^{2}H NMR (77 MHz, $CHCl_3$): δ 7.43 (0.52D, s). *Deuterium incorporation was calculated by integration of both ^{1}H NMR and ^{2}H NMR signals.*

Deuterio-2c

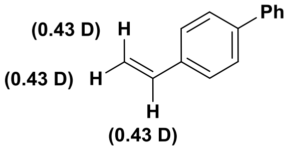

^{1}H NMR (400 MHz, $CDCl_3$): δ 7.79 – 7.31 (9H, m), 6.86 – 6.74 (0.57H, m), 5.86 – 5.76 (0.57H, m), 5.35 – 5.24 (0.57H, m); ^{2}H NMR (77 MHz, $CHCl_3$): δ 6.83 (0.43D, s), 5.86 (0.43D, s), 5.34 (0.43D, s). *Deuterium incorporation was calculated by integration of both ^{1}H NMR and ^{2}H NMR signals.*

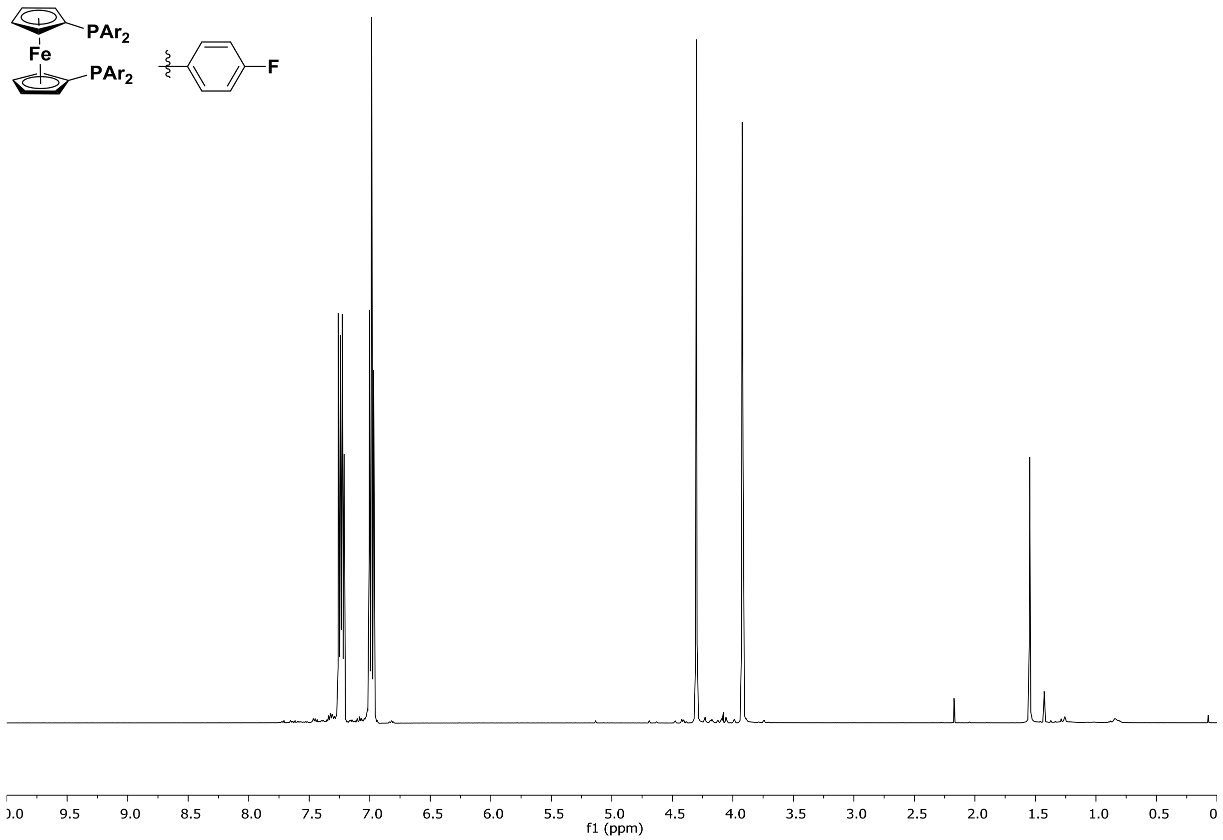
PAr2
Fe
PAr2
F
f1 (ppm)

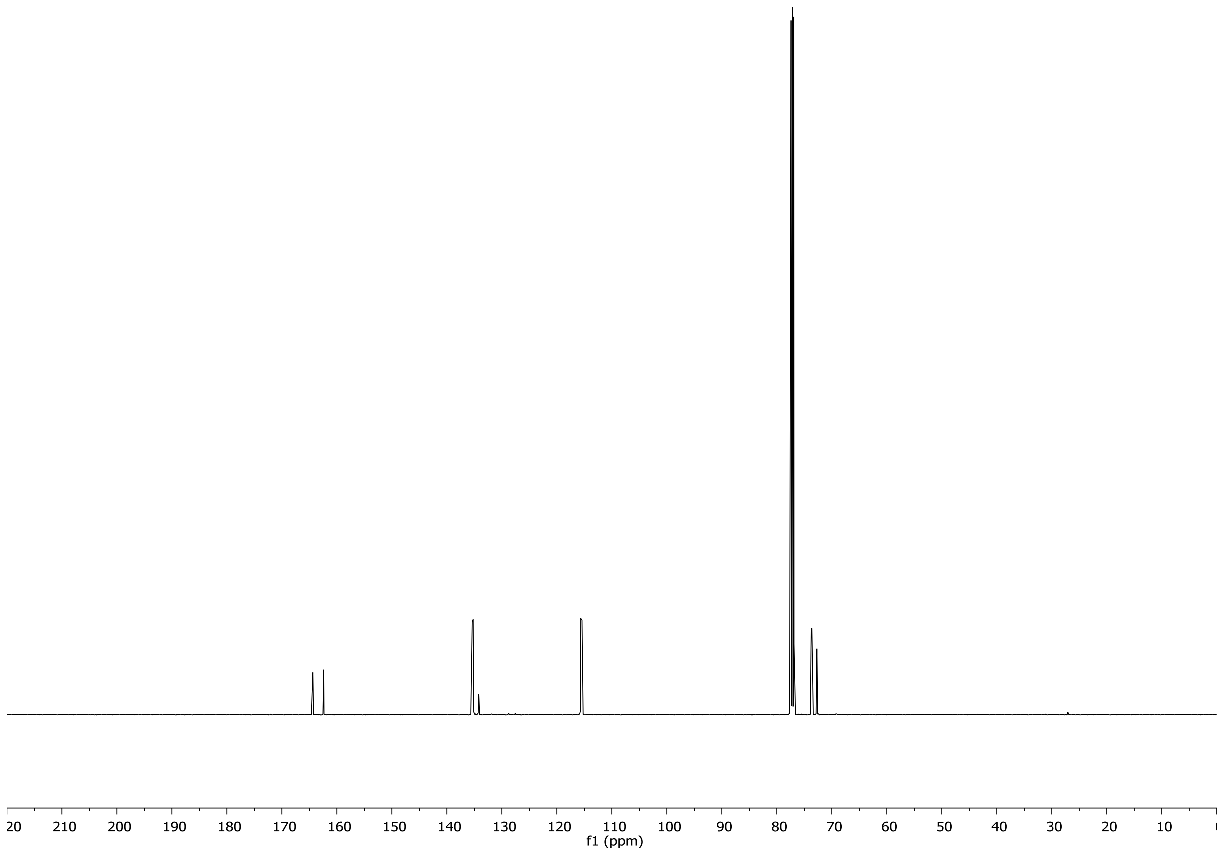
f1 (ppm)

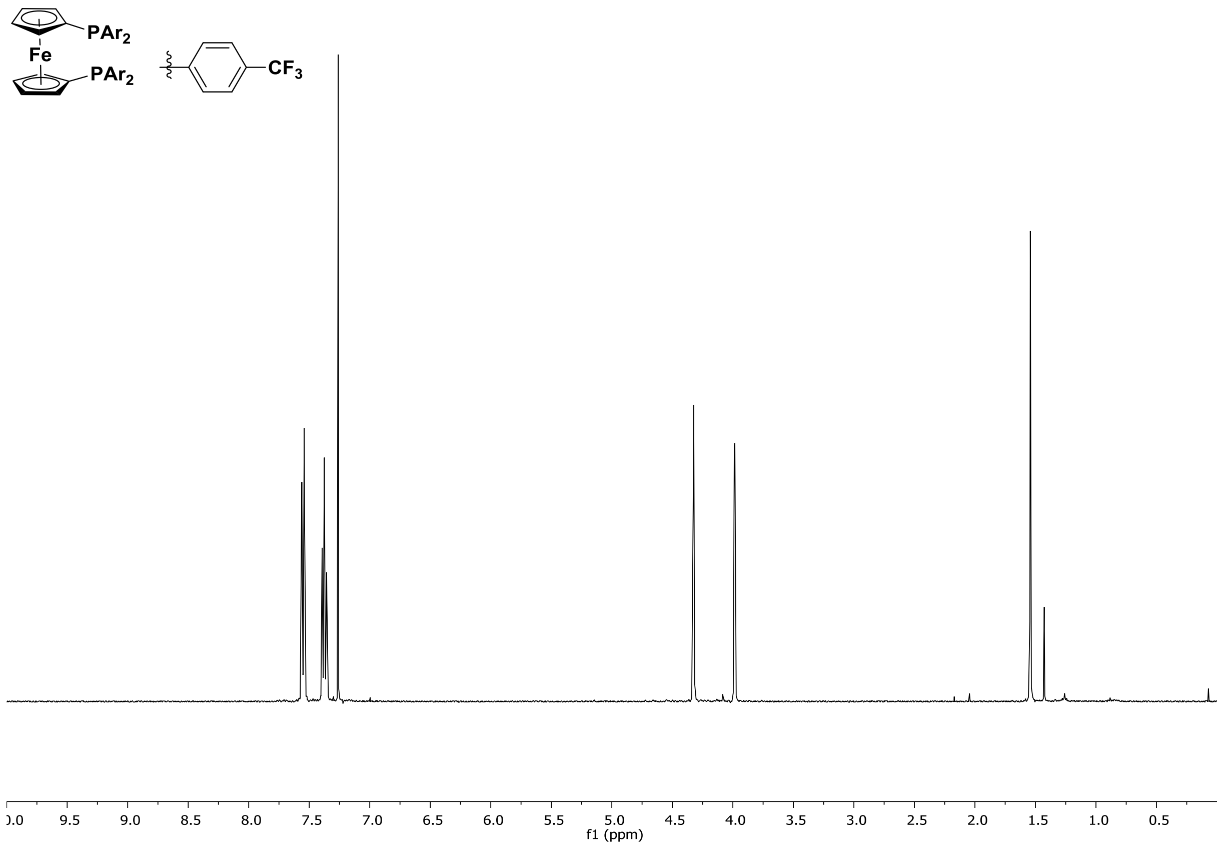
PAr2
Fe
PAr2
CF3
f1 (ppm)

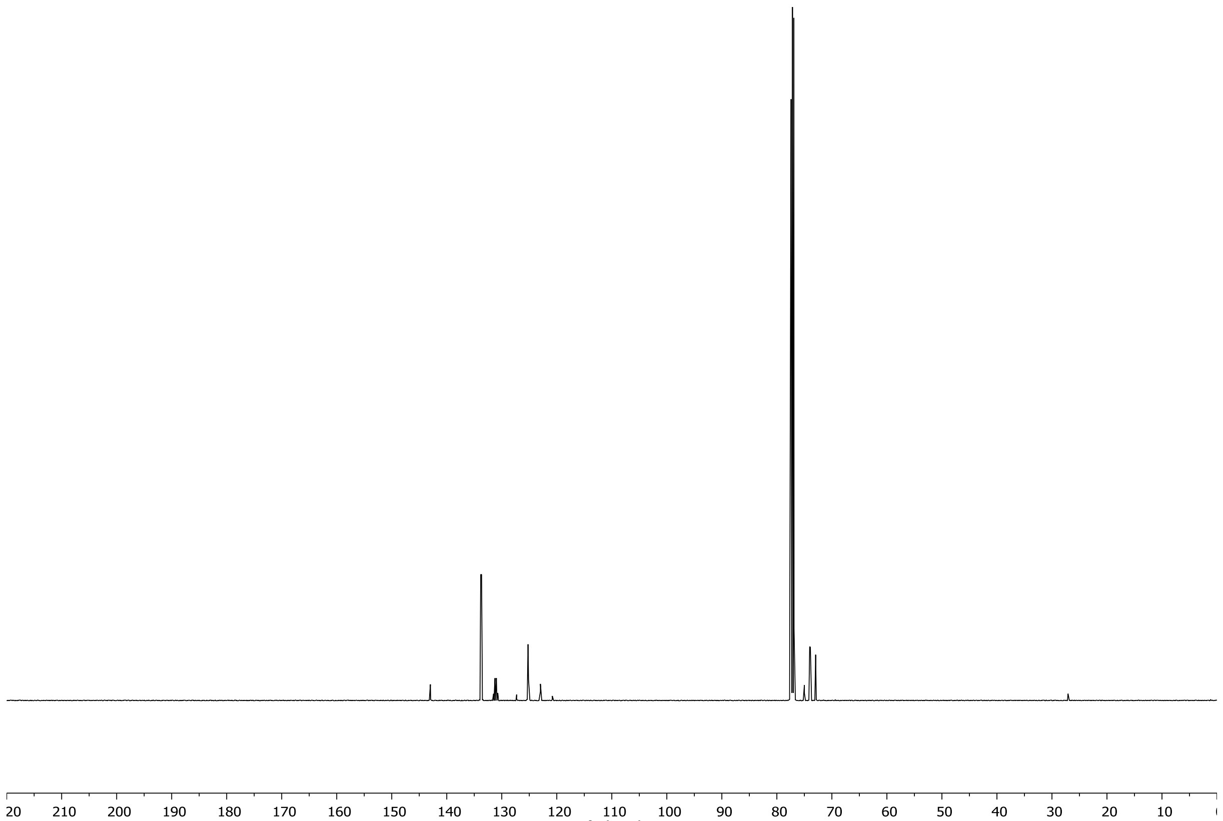
f1 (ppm)

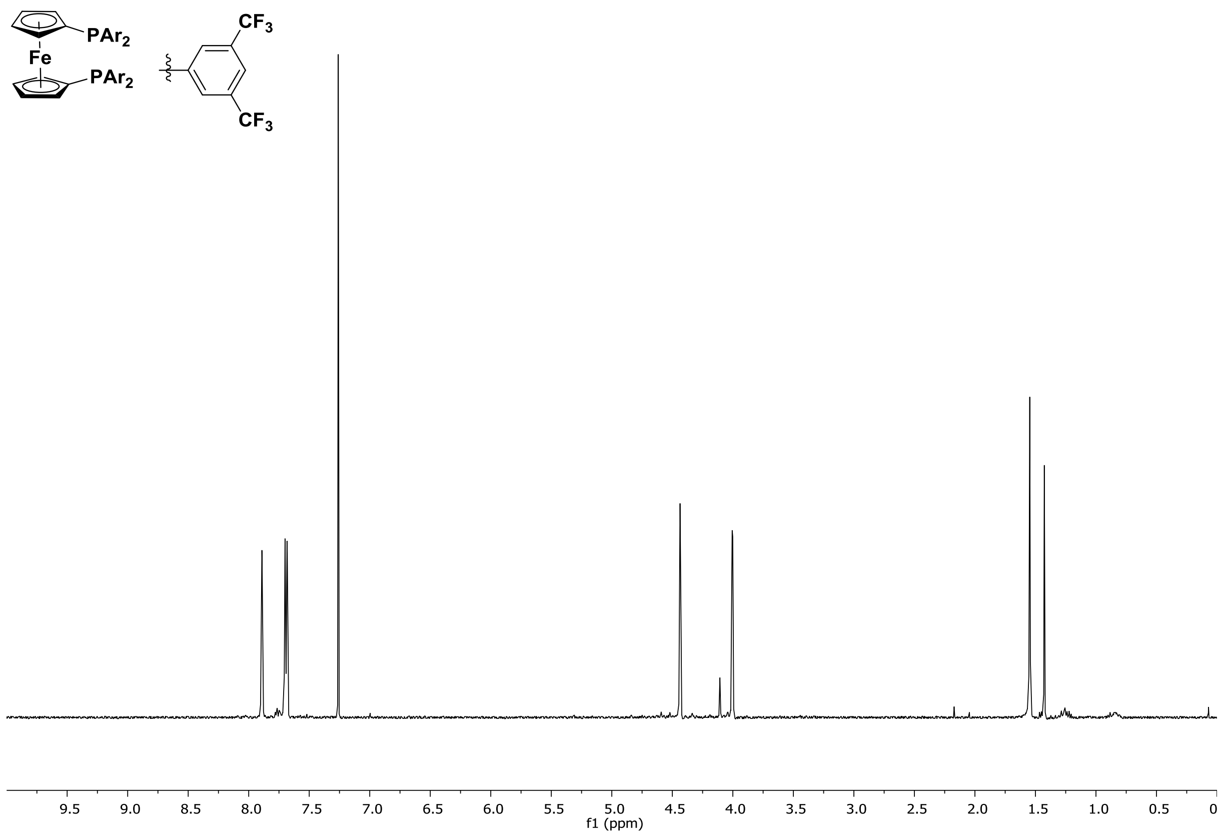
PAr2
Fe
PAr2
CF3
CF3
9.5 9.0 8.5 8.0 7.5 7.0 6.5 6.0 5.5 5.0 4.5 4.0 3.5 3.0 2.5 2.0 1.5 1.0 0.5 0
f1 (ppm)

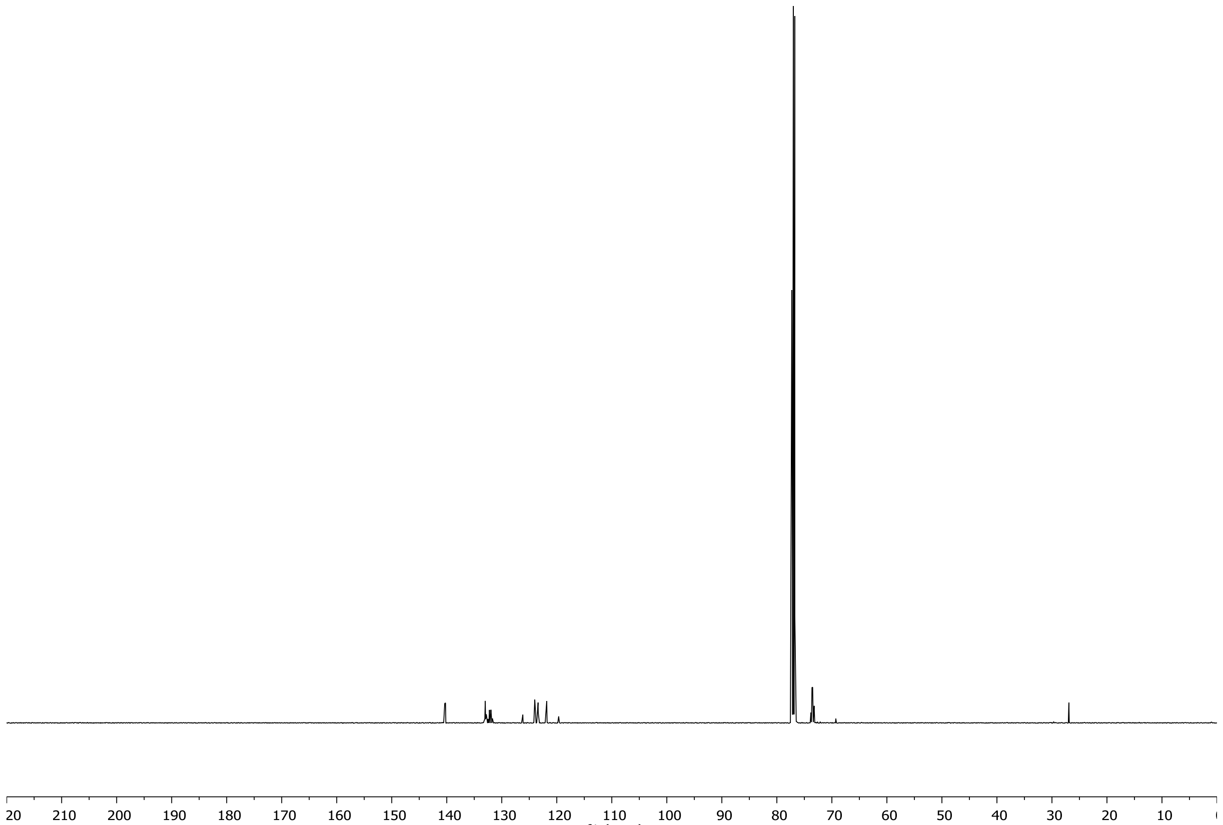
20 210 200 190 180 170 160 150 140 130 120 110 100 90 80 70 60 50 40 30 20 10
f1 (ppm)

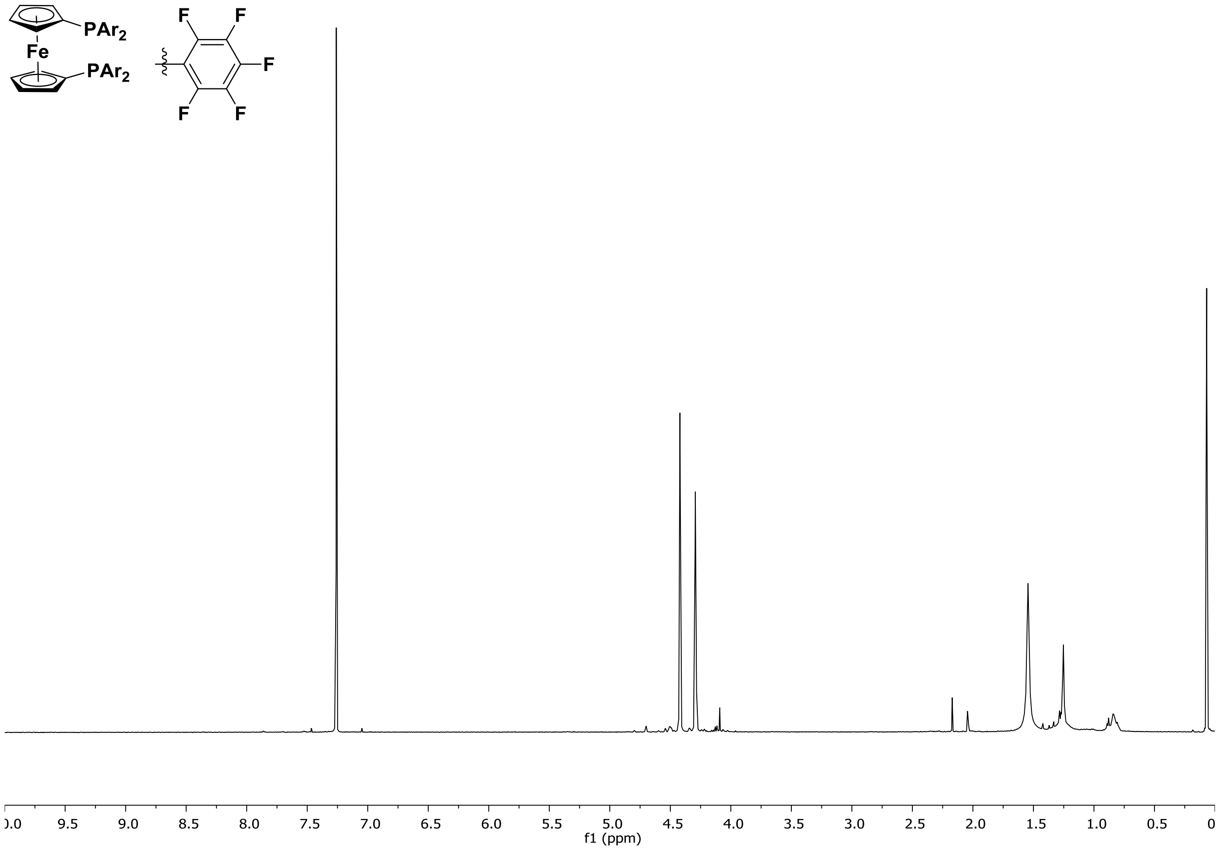
PAr2
Fe
PAr2
F
F
F
F
F
9.5
9.0
8.5
8.0
7.5
7.0
6.5
6.0
5.5
5.0
4.5
4.0
3.5
3.0
2.5
2.0
1.5
1.0
0.5
0
f1 (ppm)

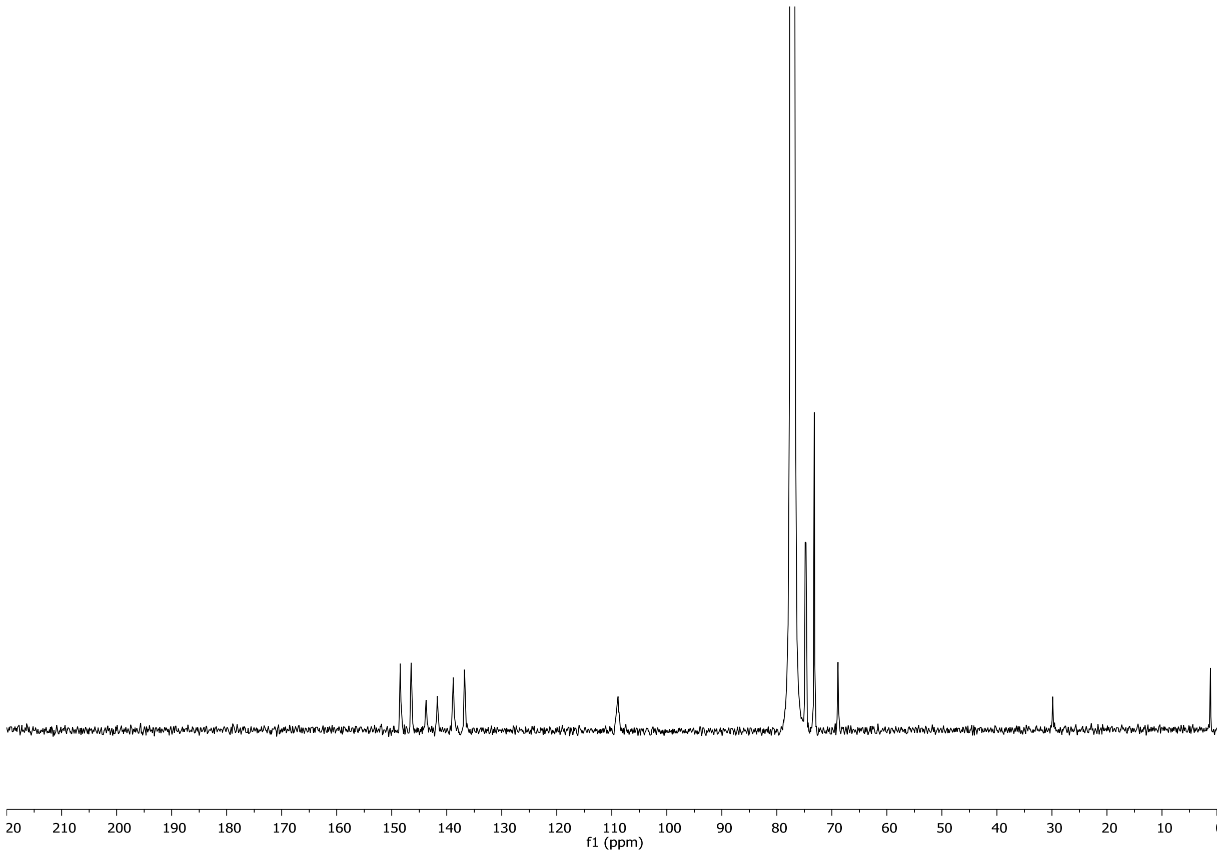
20 210 200 190 180 170 160 150 140 130 120 110 100 90 80 70 60 50 40 30 20 10
f1 (ppm)

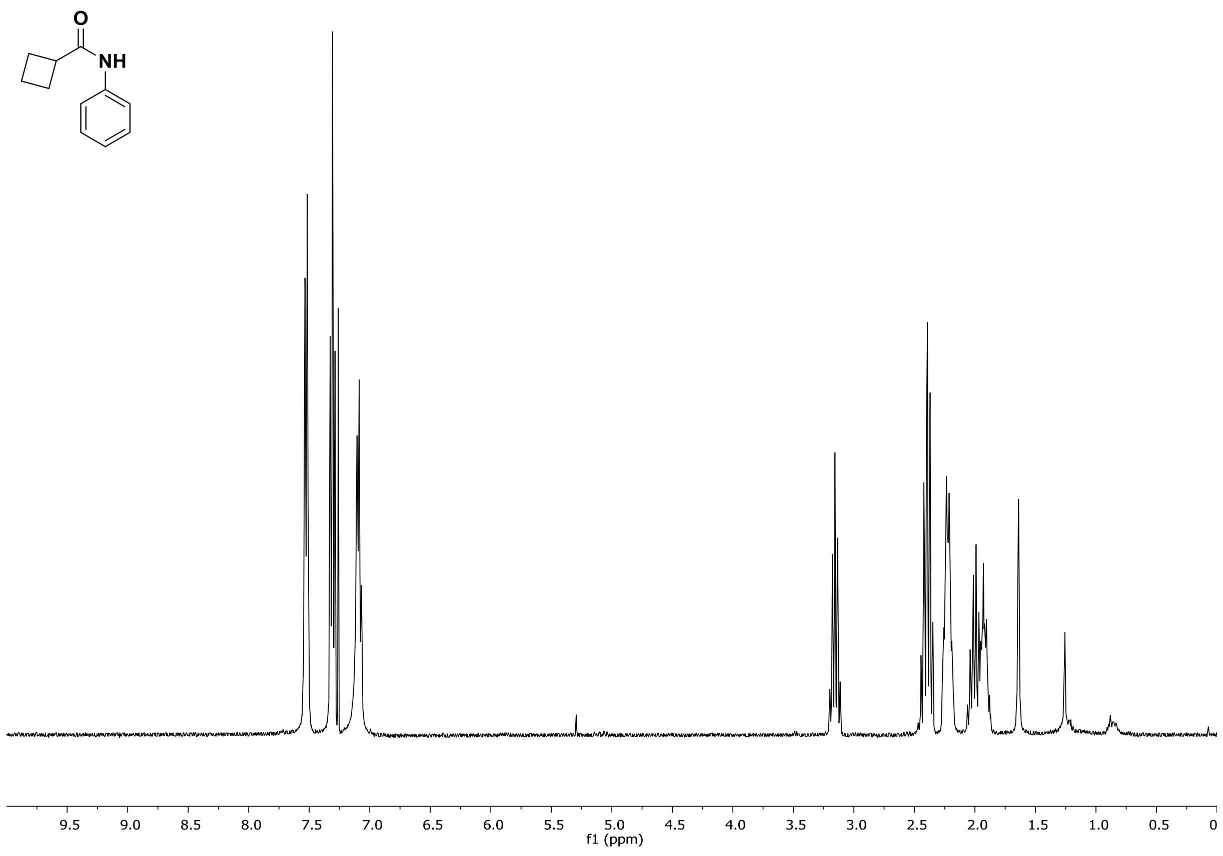
O
NH
9.5 9.0 8.5 8.0 7.5 7.0 6.5 6.0 5.5 5.0 4.5 4.0 3.5 3.0 2.5 2.0 1.5 1.0 0.5 0
f1 (ppm)

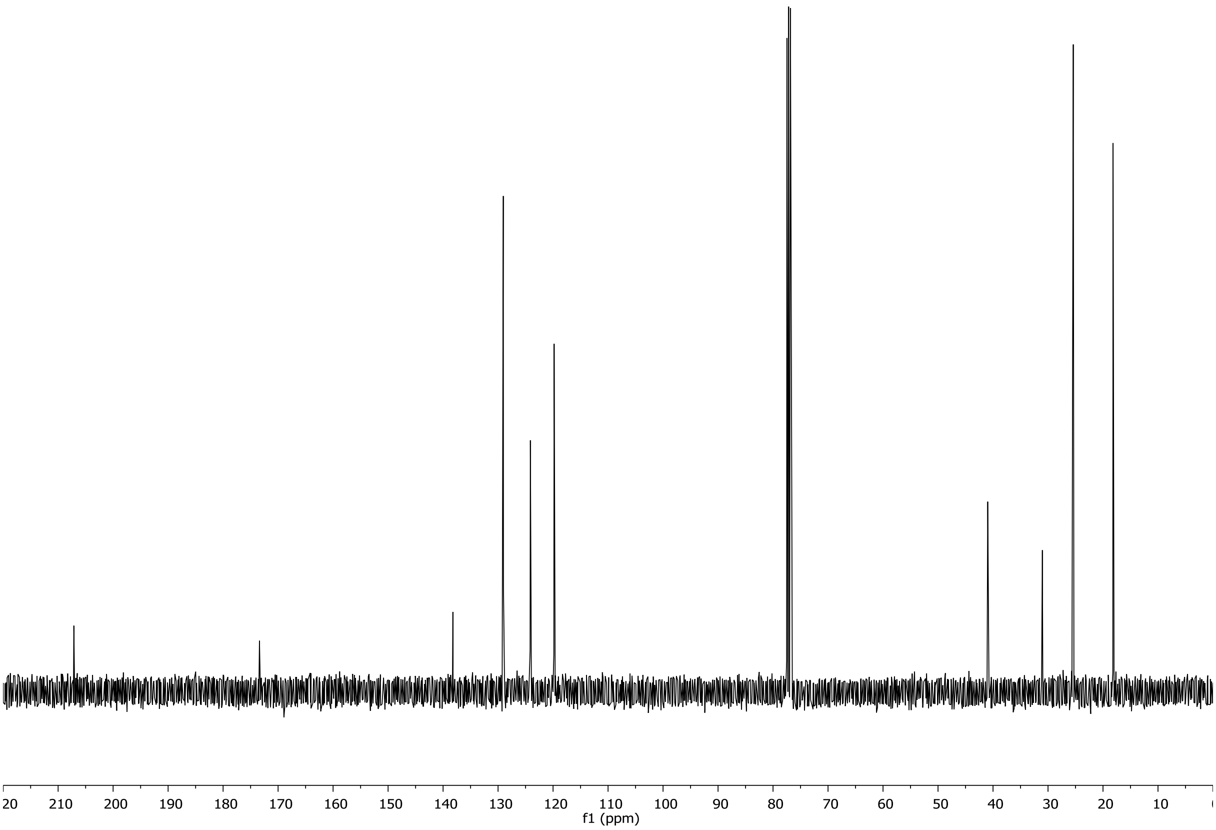
20 210 200 190 180 170 160 150 140 130 120 110 100 90 80 70 60 50 40 30 20 10
f1 (ppm)

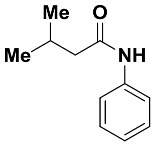
Me
O
Me
NH

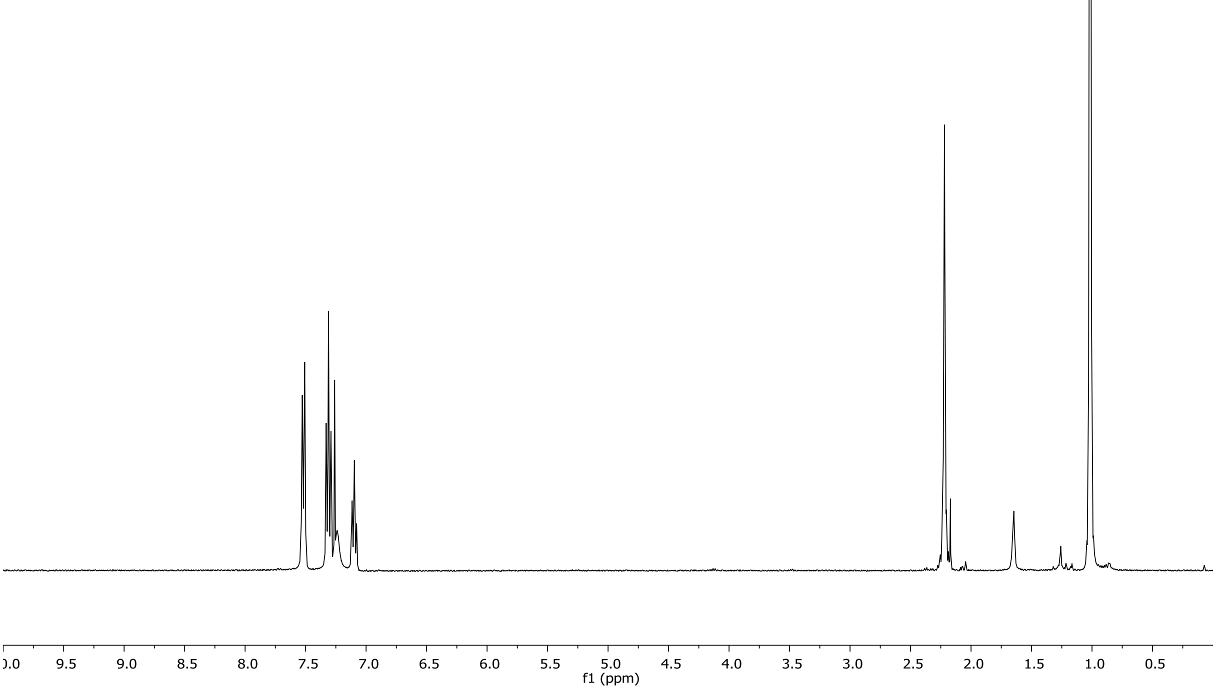
9.5 9.0 8.5 8.0 7.5 7.0 6.5 6.0 5.5 5.0 4.5 4.0 3.5 3.0 2.5 2.0 1.5 1.0 0.5
f1 (ppm)

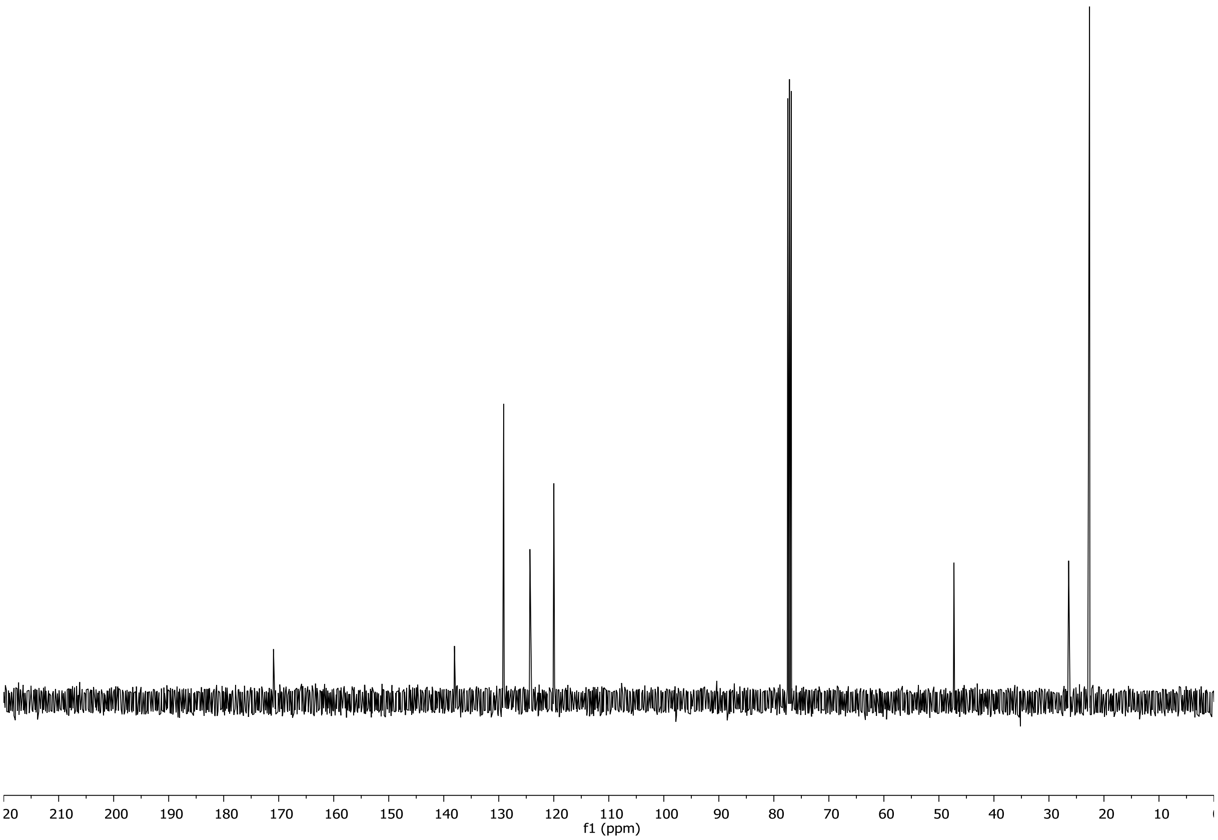
f1 (ppm)

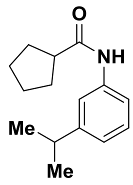
O
NH
Me
Me

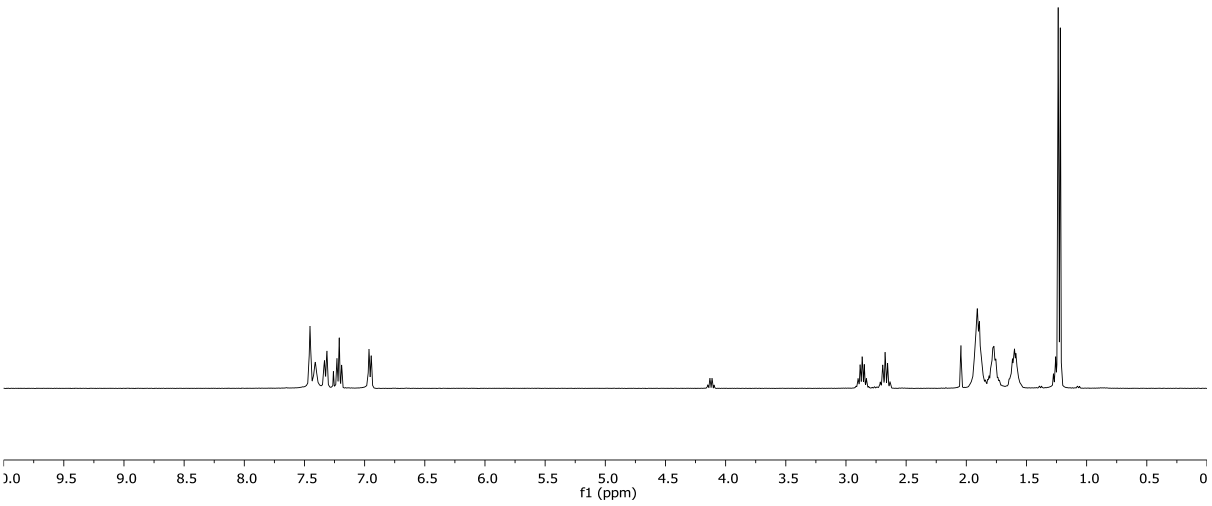
f1 (ppm)

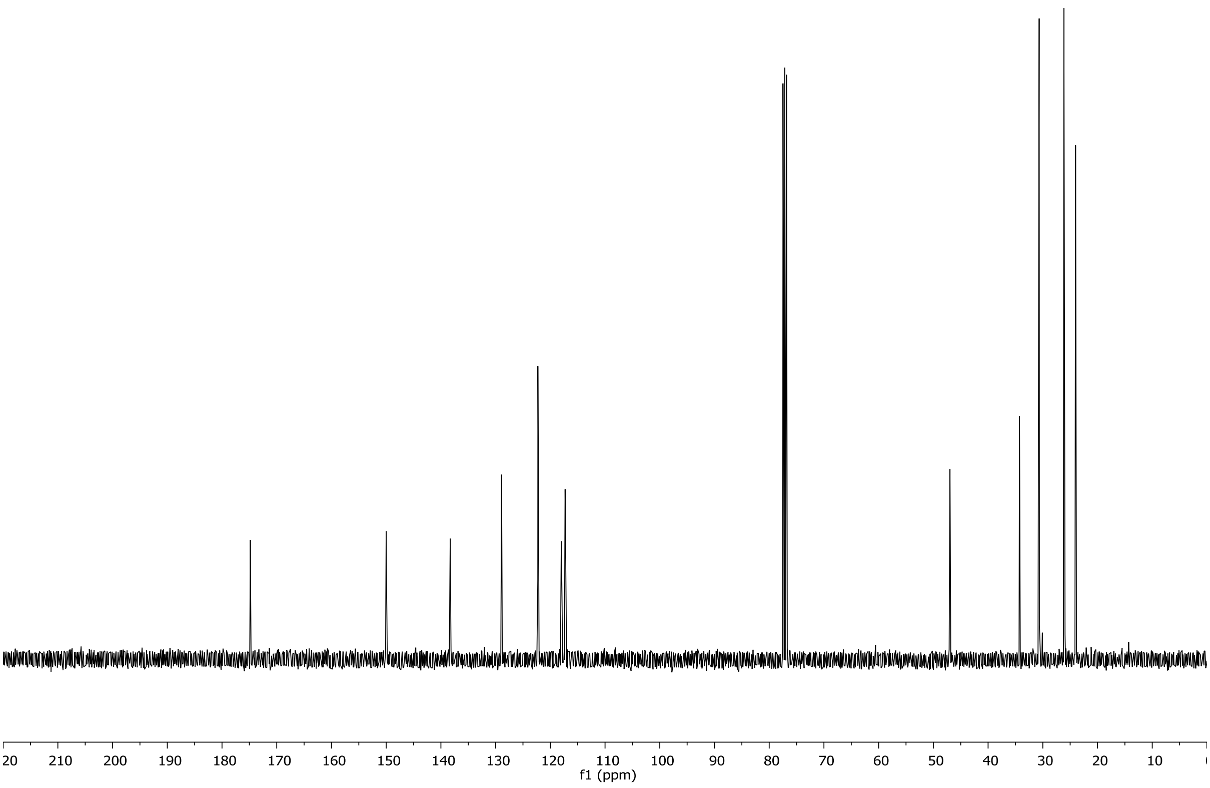
20 210 200 190 180 170 160 150 140 130 120 110 100 90 80 70 60 50 40 30 20 10
f1 (ppm)

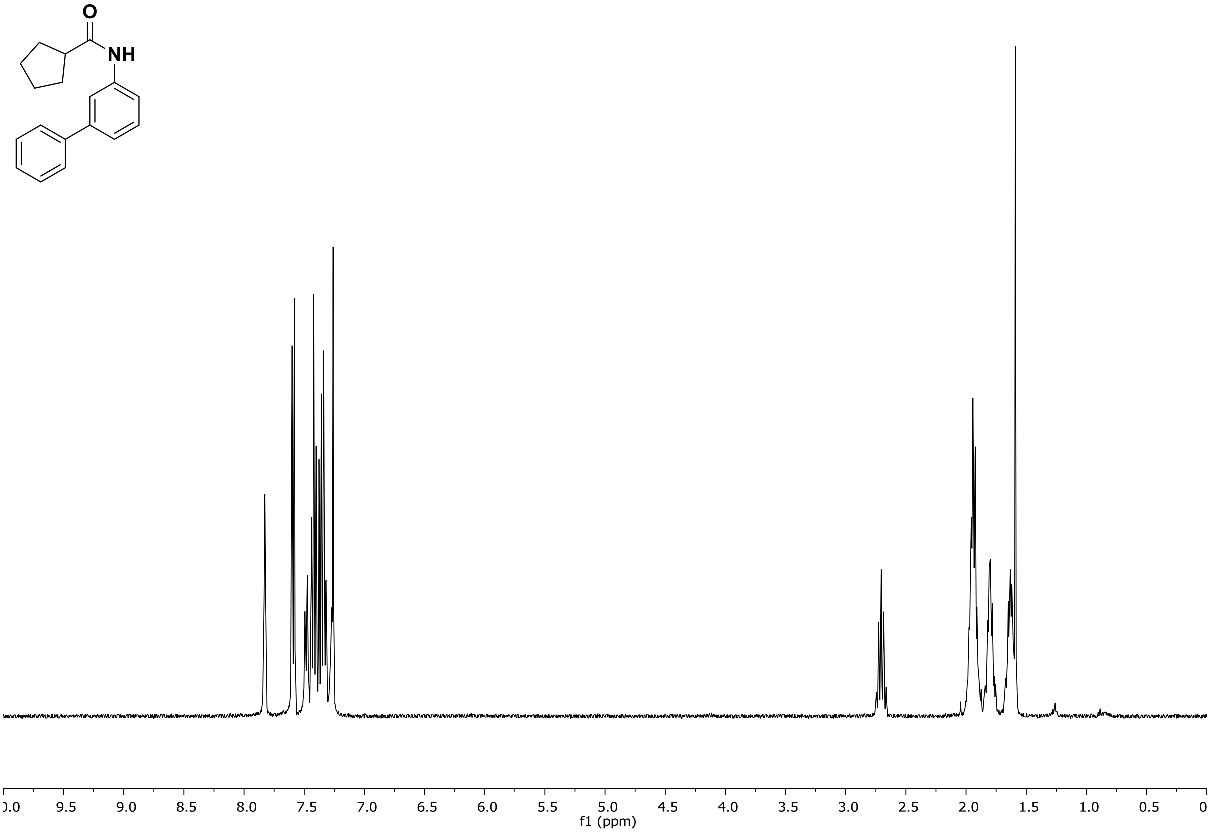
O
NH
).0 9.5 9.0 8.5 8.0 7.5 7.0 6.5 6.0 5.5 5.0 4.5 4.0 3.5 3.0 2.5 2.0 1.5 1.0 0.5 0
f1 (ppm)

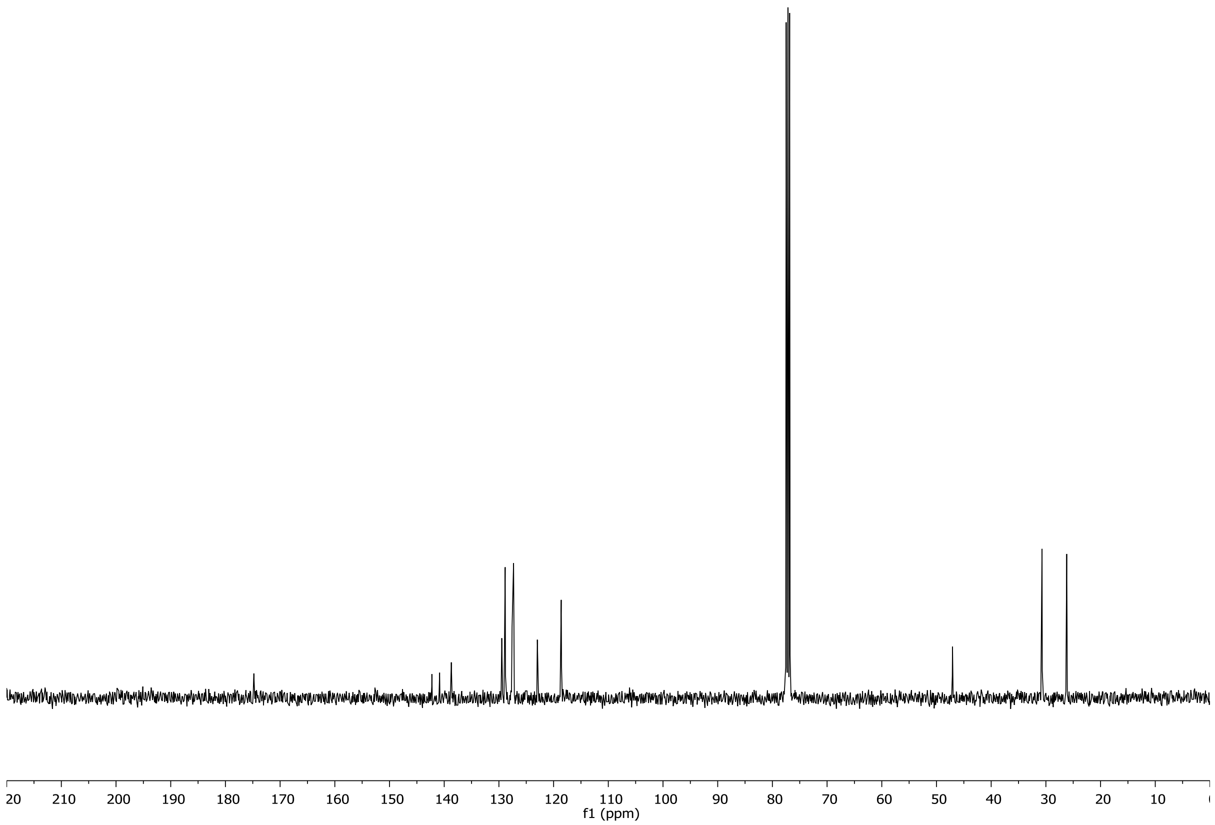
20 210 200 190 180 170 160 150 140 130 120 110 100 90 80 70 60 50 40 30 20 10
f1 (ppm)

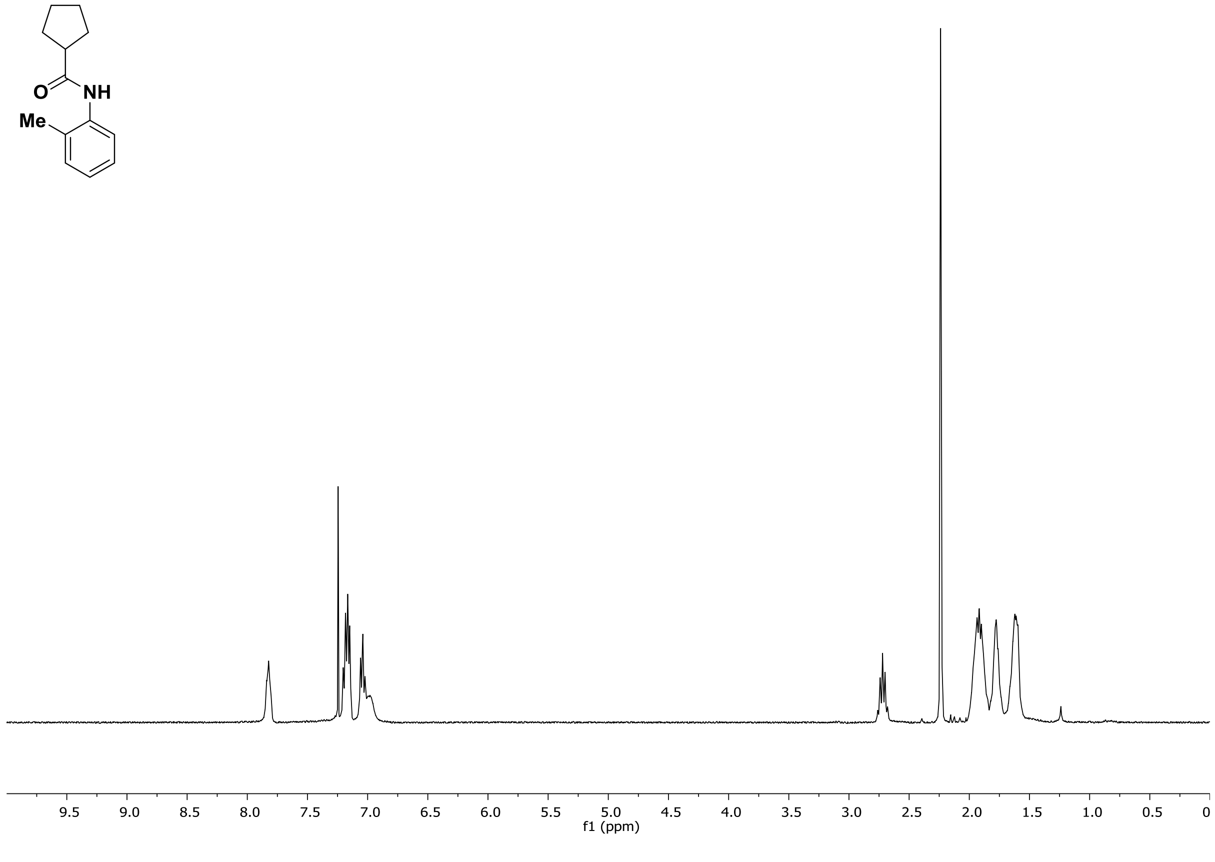
O
NH
Me
9.5 9.0 8.5 8.0 7.5 7.0 6.5 6.0 5.5 5.0 4.5 4.0 3.5 3.0 2.5 2.0 1.5 1.0 0.5 0
f1 (ppm)

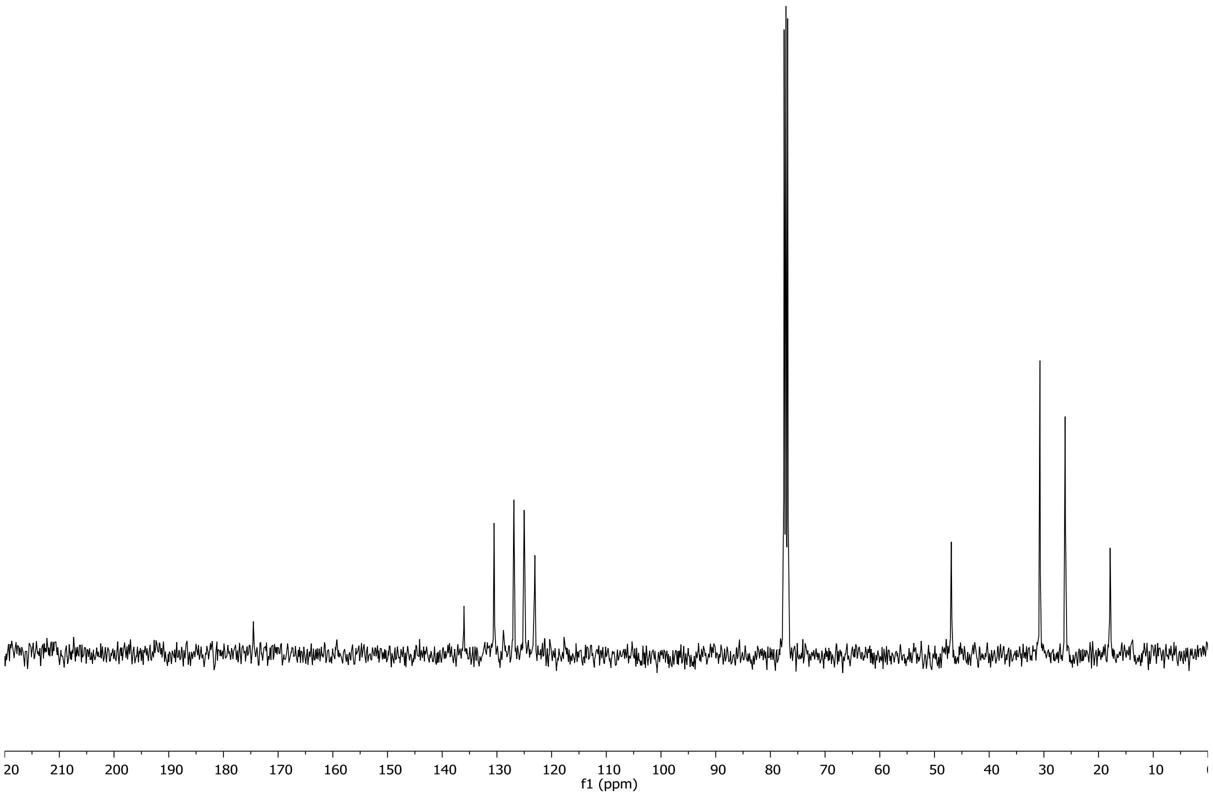
20 210 200 190 180 170 160 150 140 130 120 110 100 90 80 70 60 50 40 30 20 10
f1 (ppm)

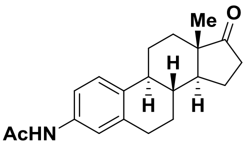
Me
O
H
H
H
AcHN

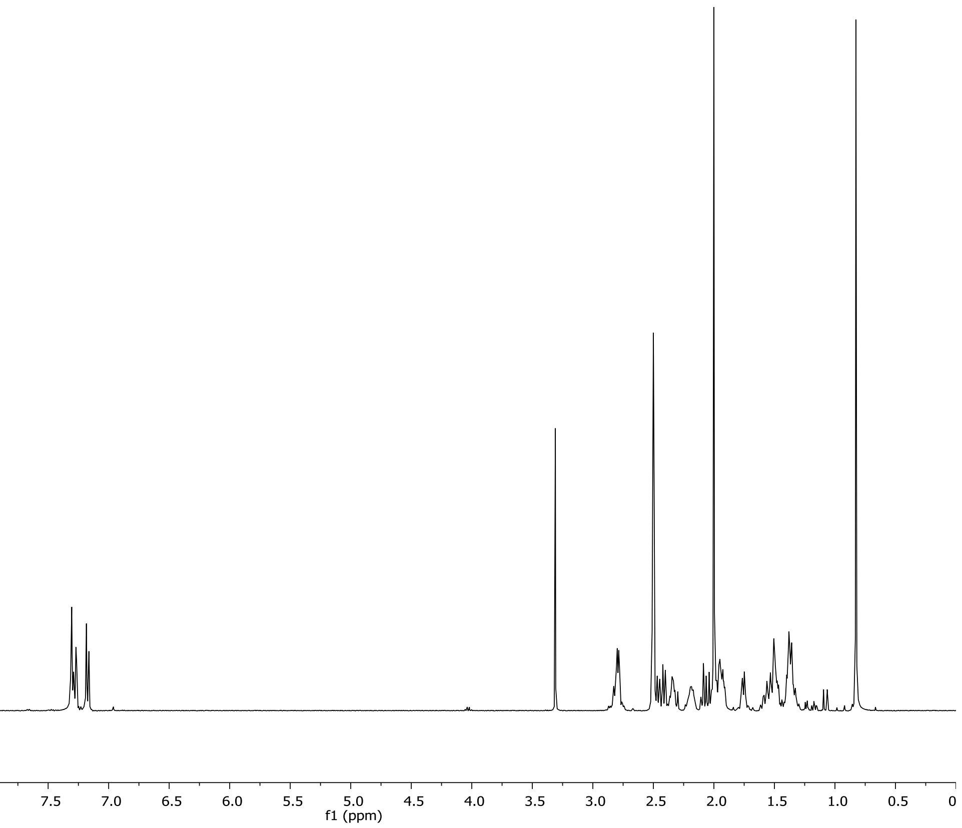
9.5 9.0 8.5 8.0 7.5 7.0 6.5 6.0 5.5 5.0 4.5 4.0 3.5 3.0 2.5 2.0 1.5 1.0 0.5 0
f1 (ppm)

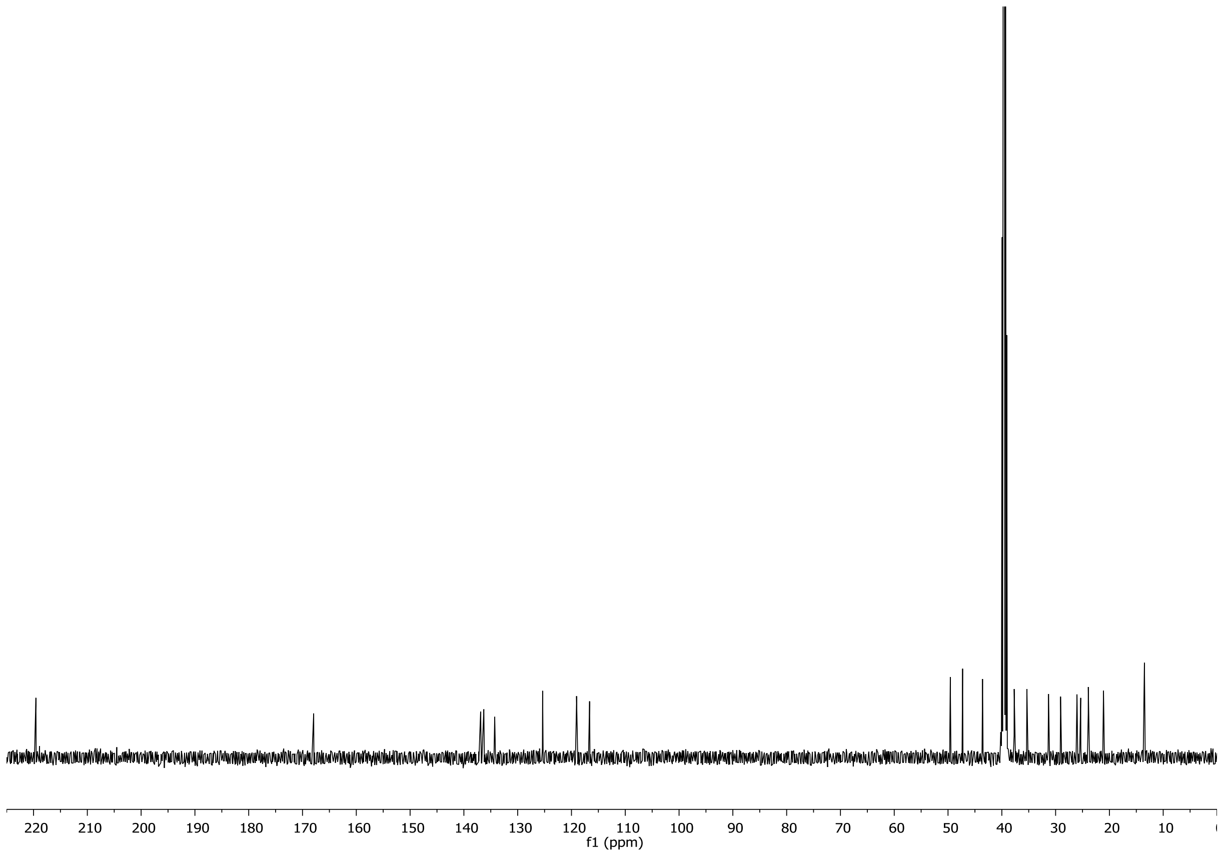
220 210 200 190 180 170 160 150 140 130 120 110 100 90 80 70 60 50 40 30 20 10
f1 (ppm)

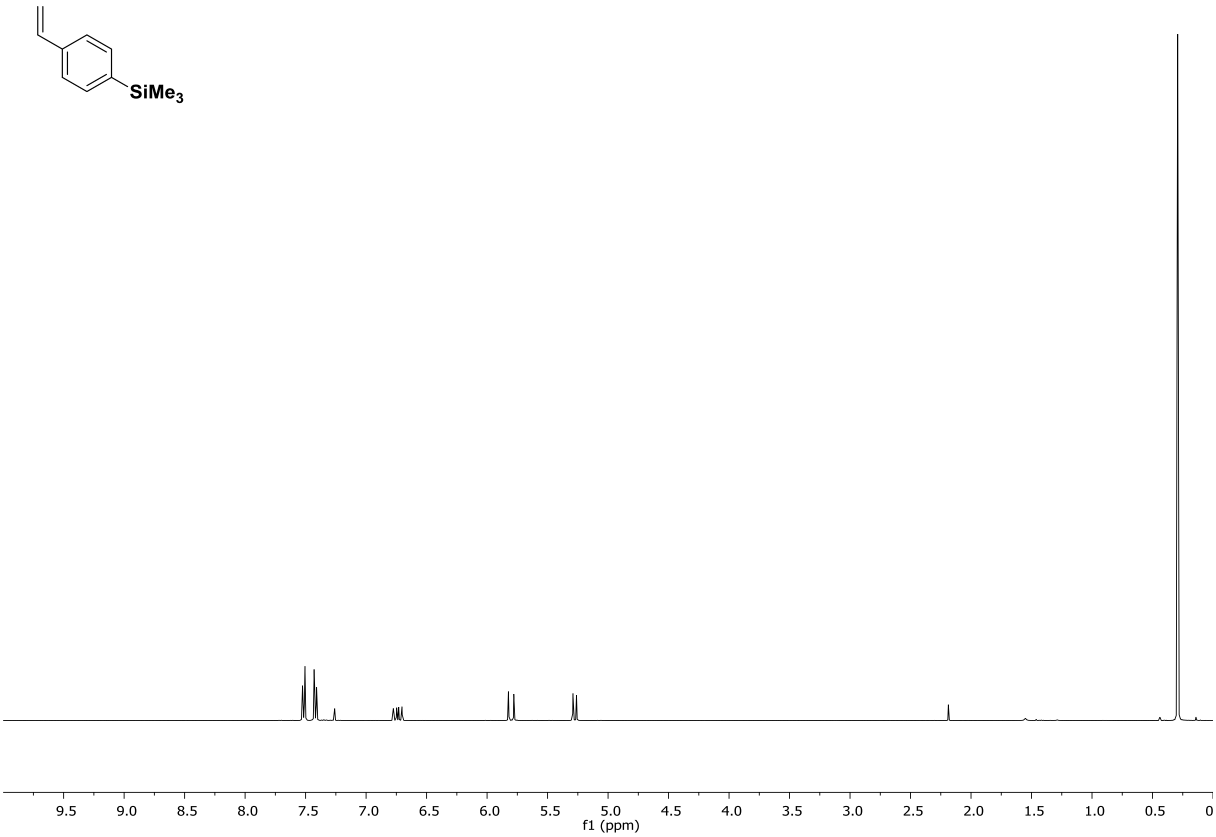
SiMe3
9.5 9.0 8.5 8.0 7.5 7.0 6.5 6.0 5.5 5.0 4.5 4.0 3.5 3.0 2.5 2.0 1.5 1.0 0.5 0
f1 (ppm)

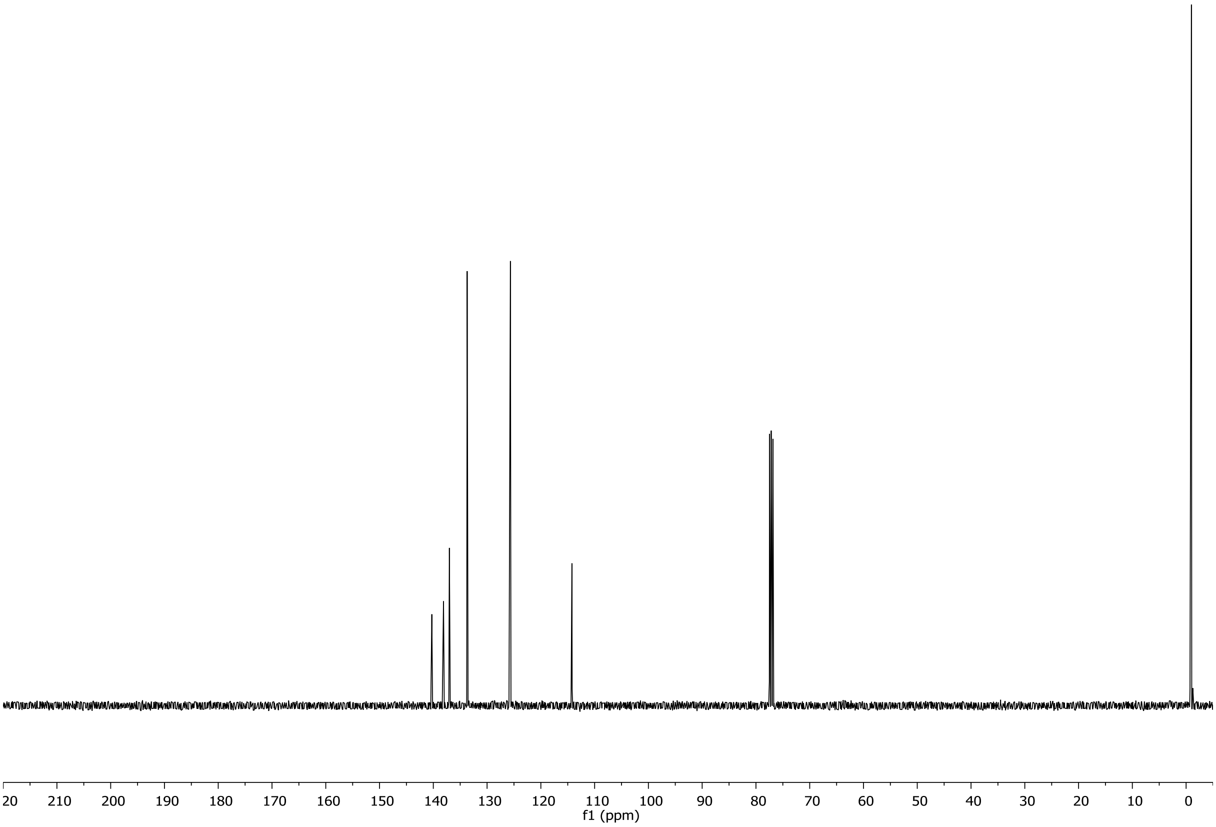
20 210 200 190 180 170 160 150 140 130 120 110 100 90 80 70 60 50 40 30 20 10 0
f1 (ppm)

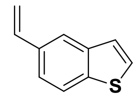
S

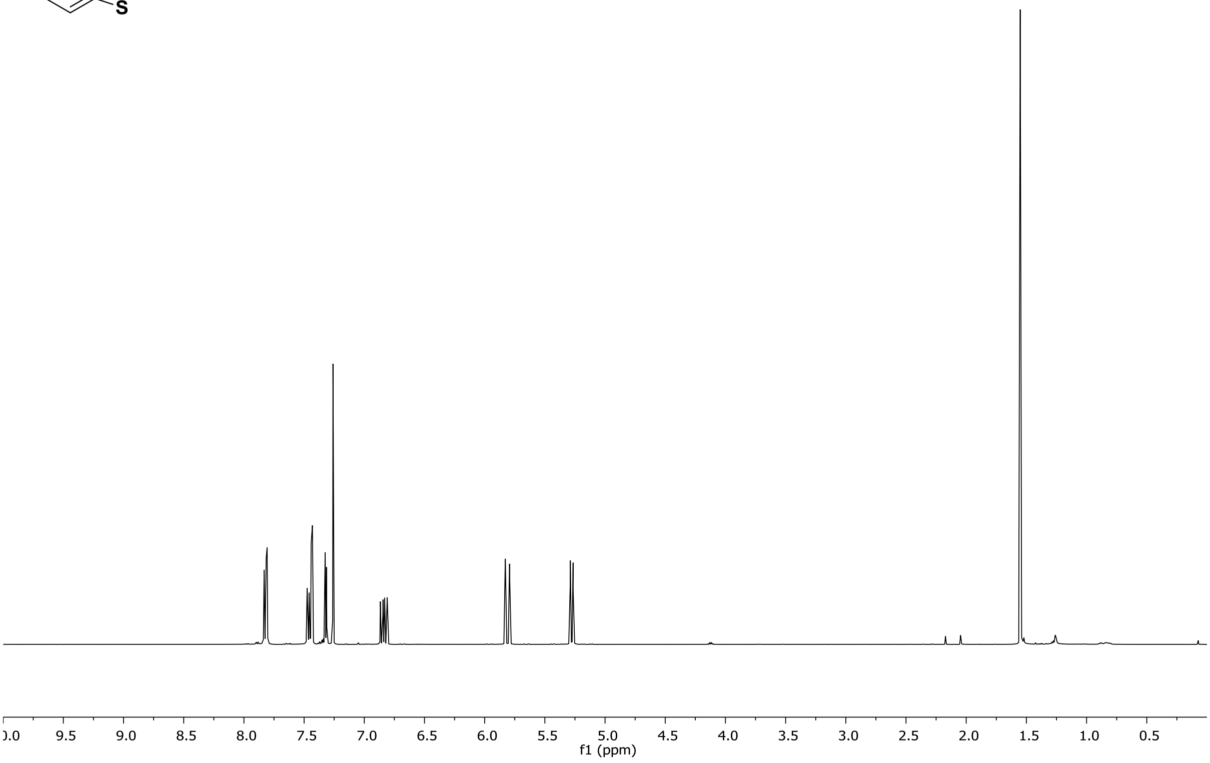
).0 9.5 9.0 8.5 8.0 7.5 7.0 6.5 6.0 5.5 5.0 4.5 4.0 3.5 3.0 2.5 2.0 1.5 1.0 0.5
f1 (ppm)

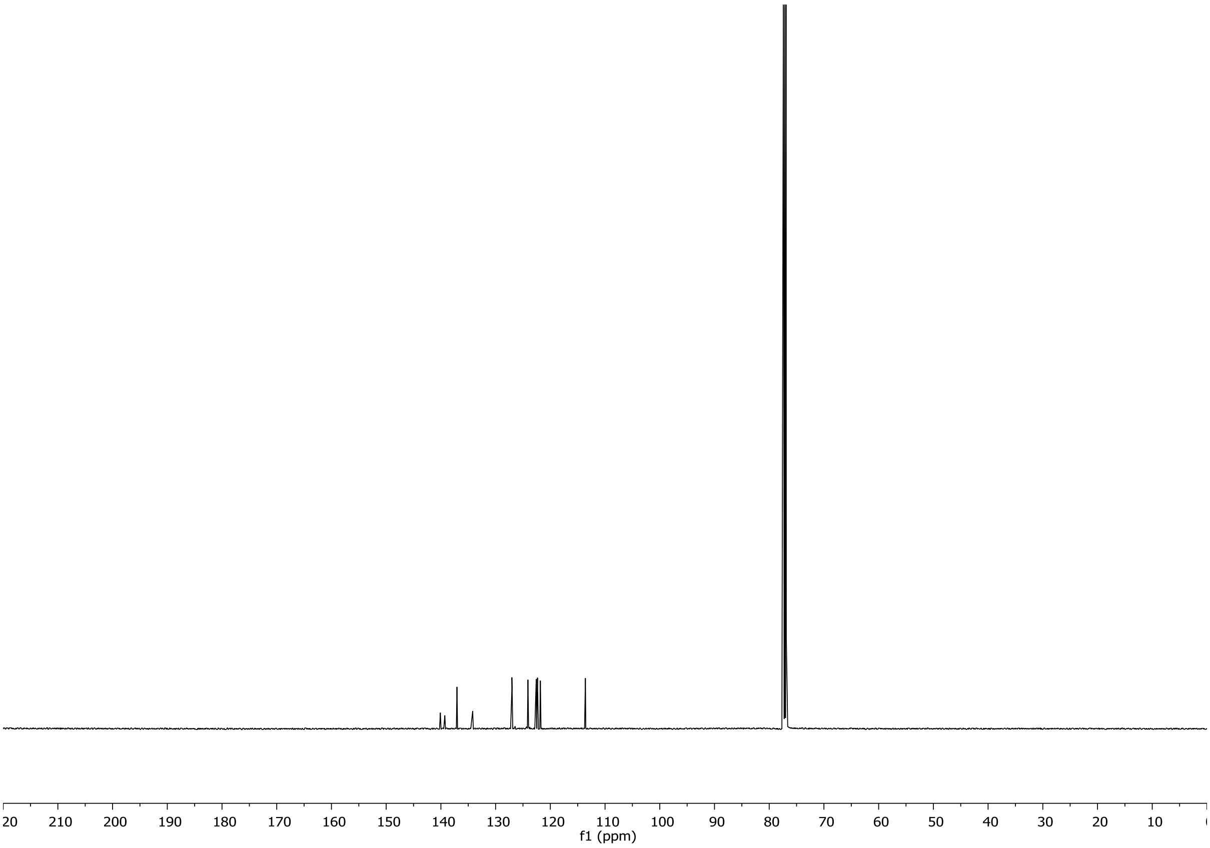
20 210 200 190 180 170 160 150 140 130 120 110 100 90 80 70 60 50 40 30 20 10
f1 (ppm)

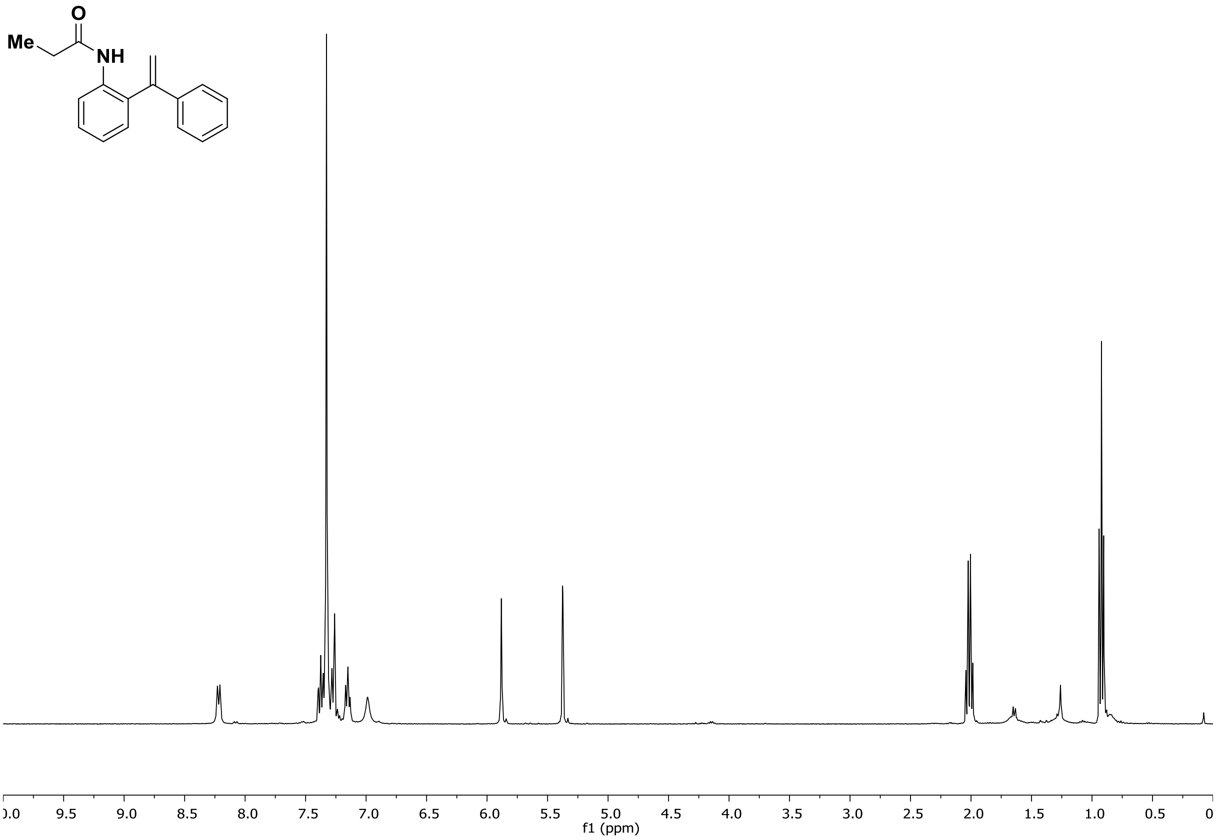
O
Me
NH
f1 (ppm)

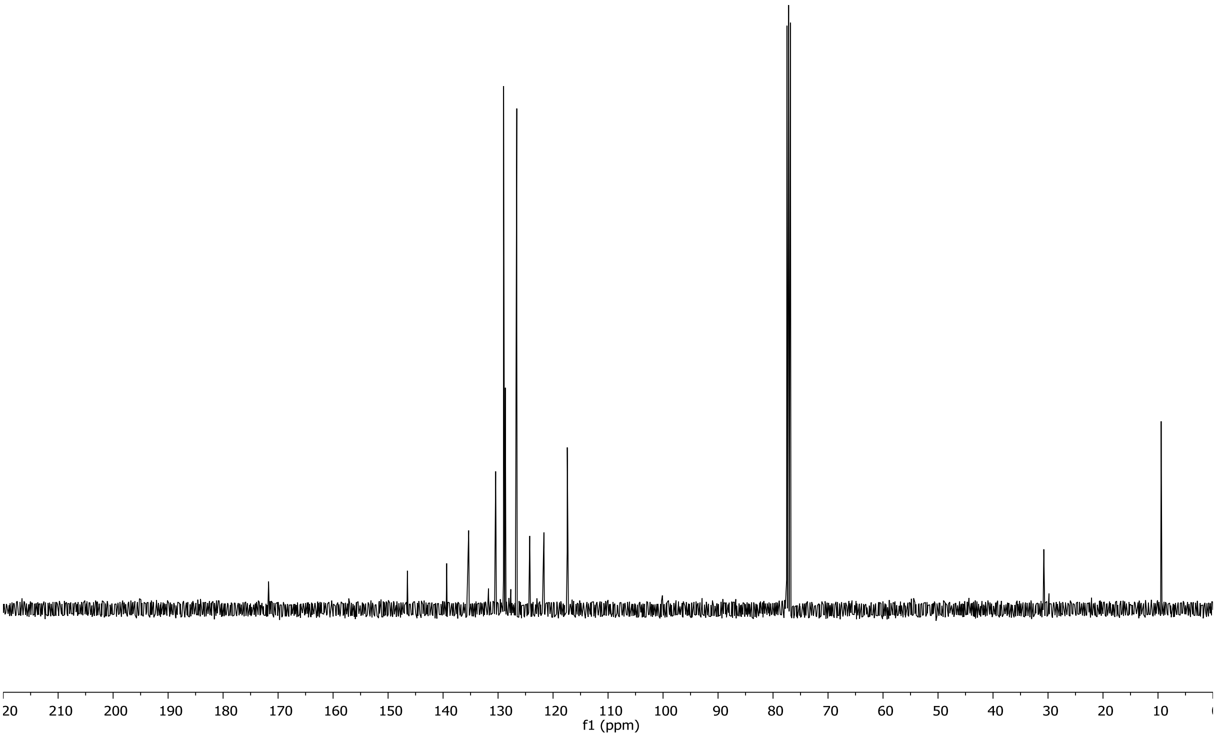
f1 (ppm)

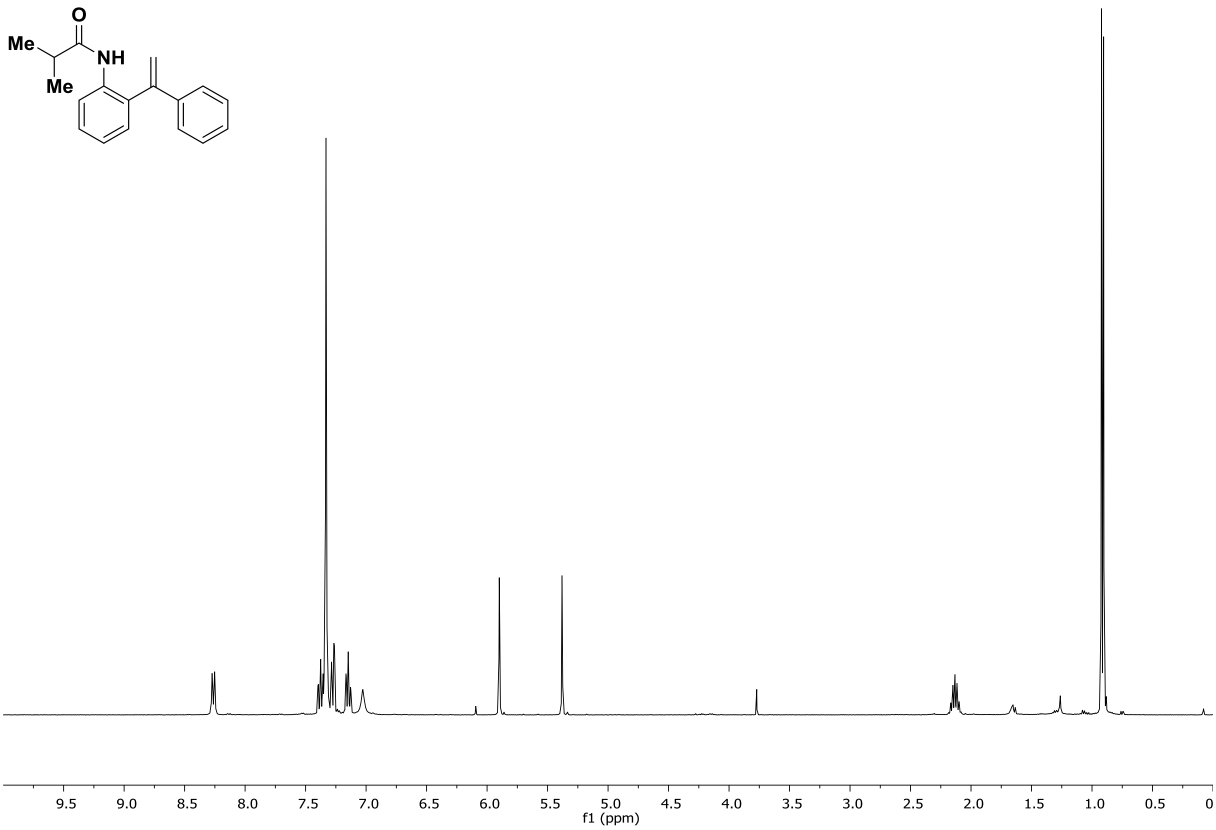
O
Me
NH
Me
9.5 9.0 8.5 8.0 7.5 7.0 6.5 6.0 5.5 5.0 4.5 4.0 3.5 3.0 2.5 2.0 1.5 1.0 0.5 0
f1 (ppm)

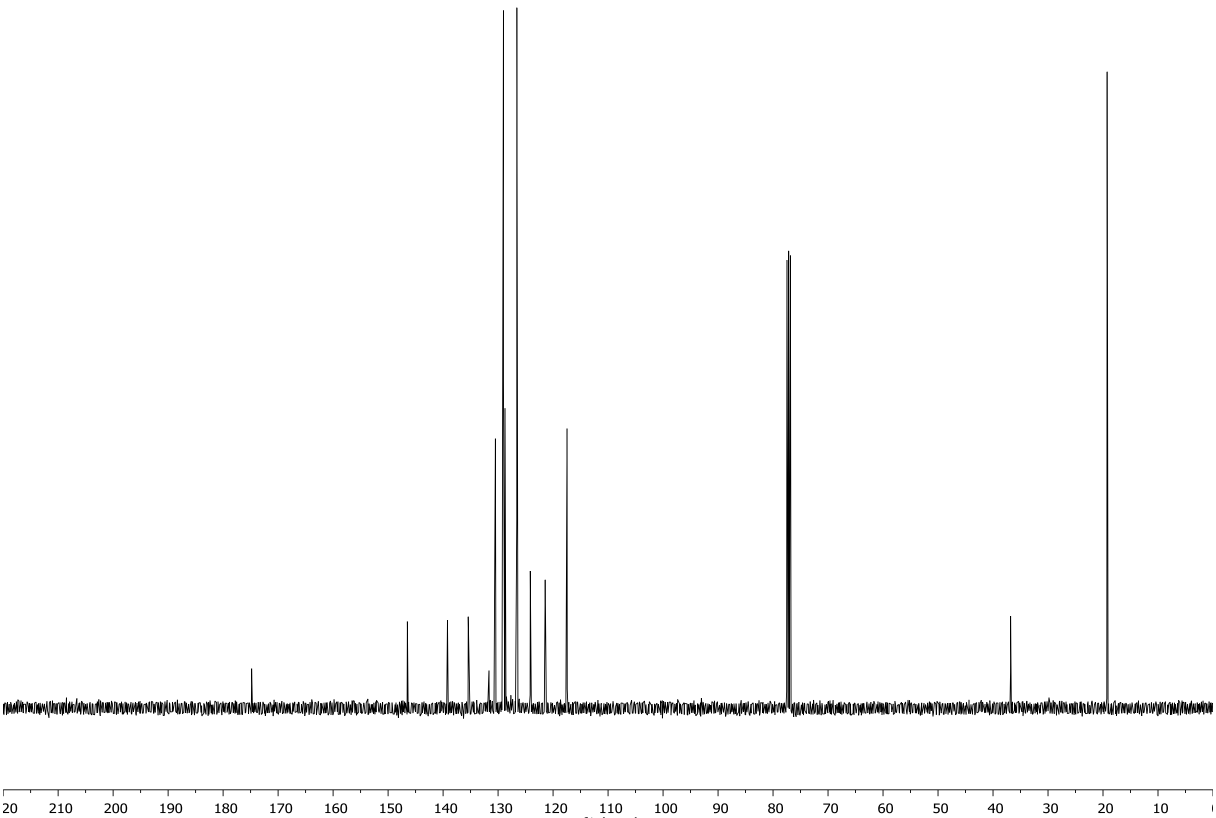
20 210 200 190 180 170 160 150 140 130 120 110 100 90 80 70 60 50 40 30 20 10
f1 (ppm)

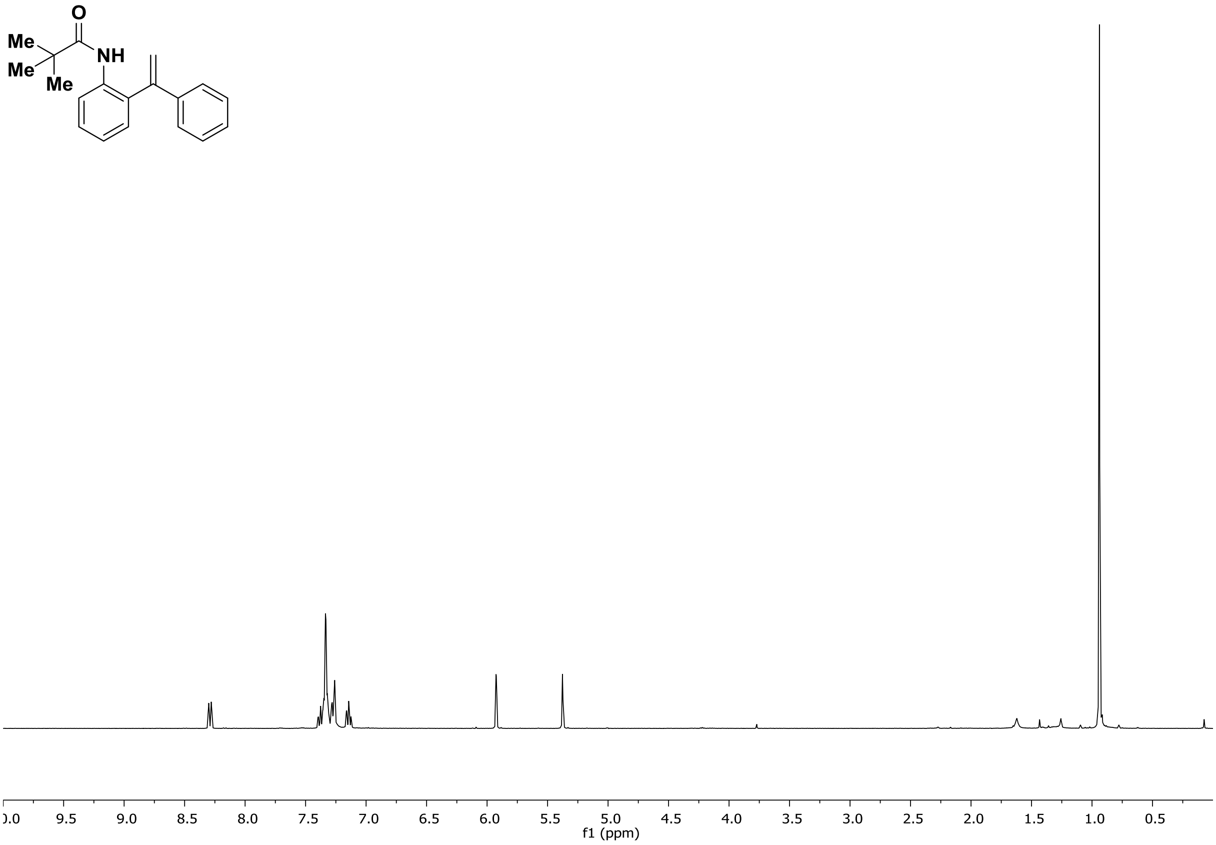
O
Me
Me
Me
NH
f1 (ppm)

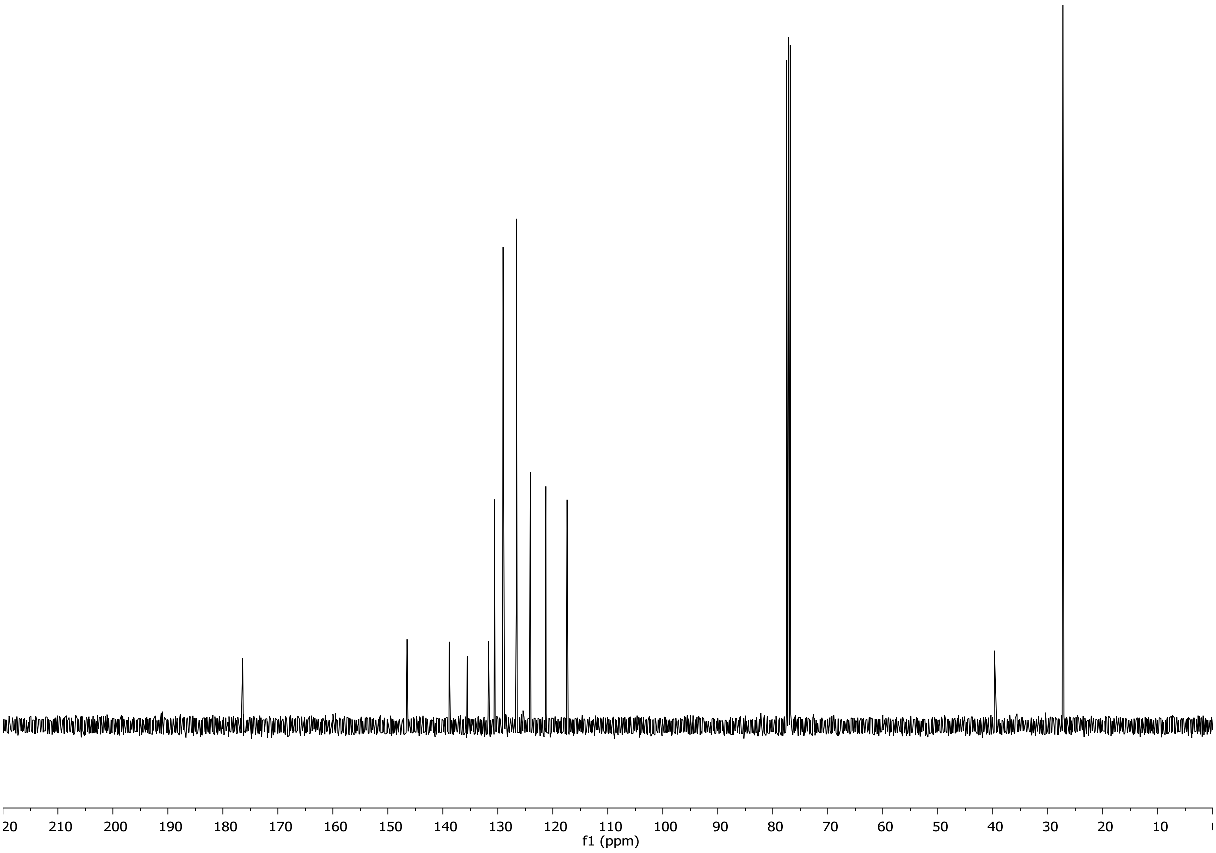
f1 (ppm)

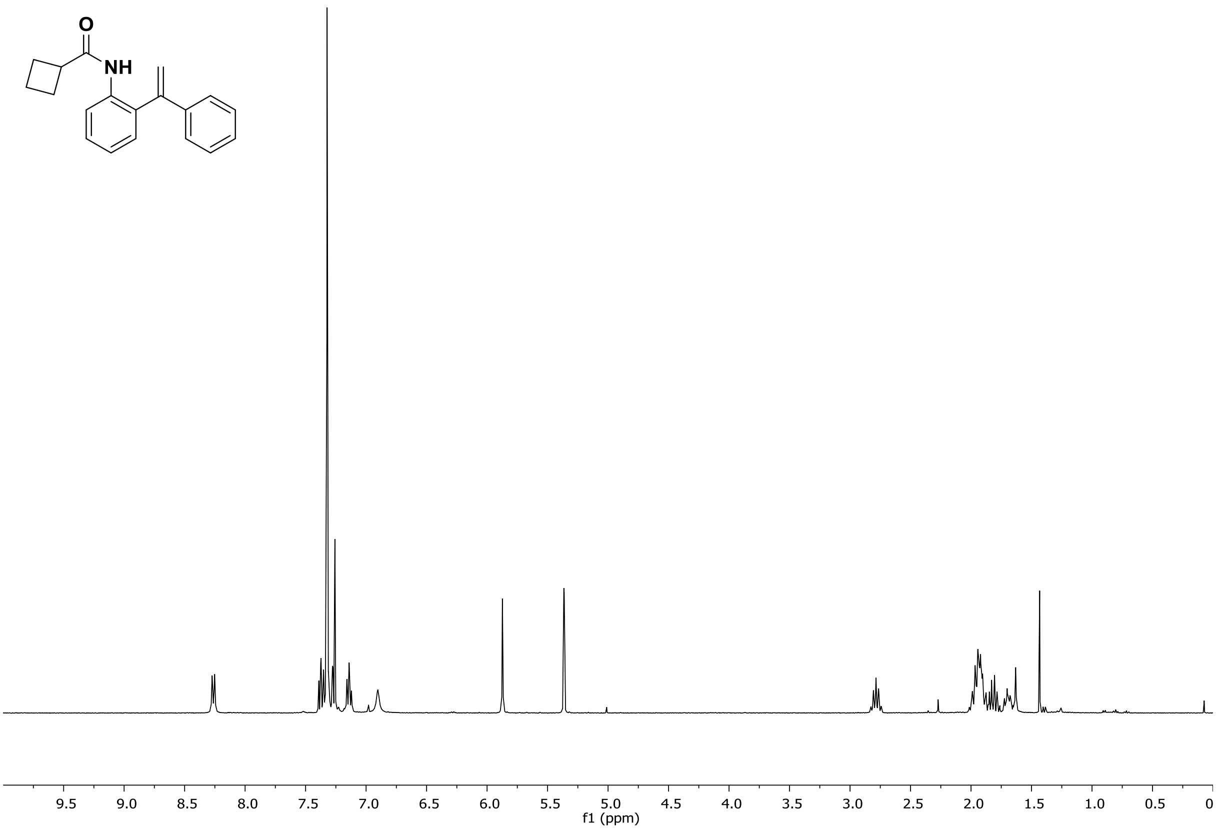
O
NH
9.5 9.0 8.5 8.0 7.5 7.0 6.5 6.0 5.5 5.0 4.5 4.0 3.5 3.0 2.5 2.0 1.5 1.0 0.5 0
f1 (ppm)

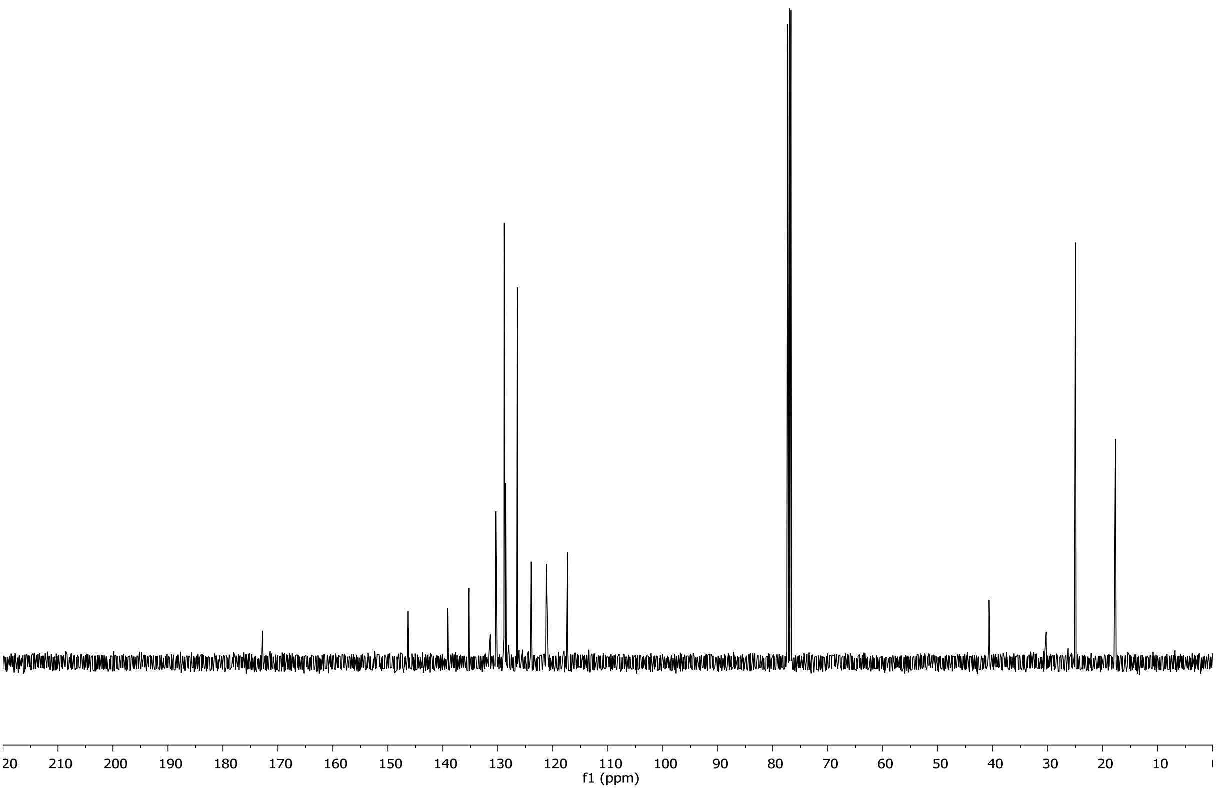
210 200 190 180 170 160 150 140 130 120 110 100 90 80 70 60 50 40 30 20 10
f1 (ppm)

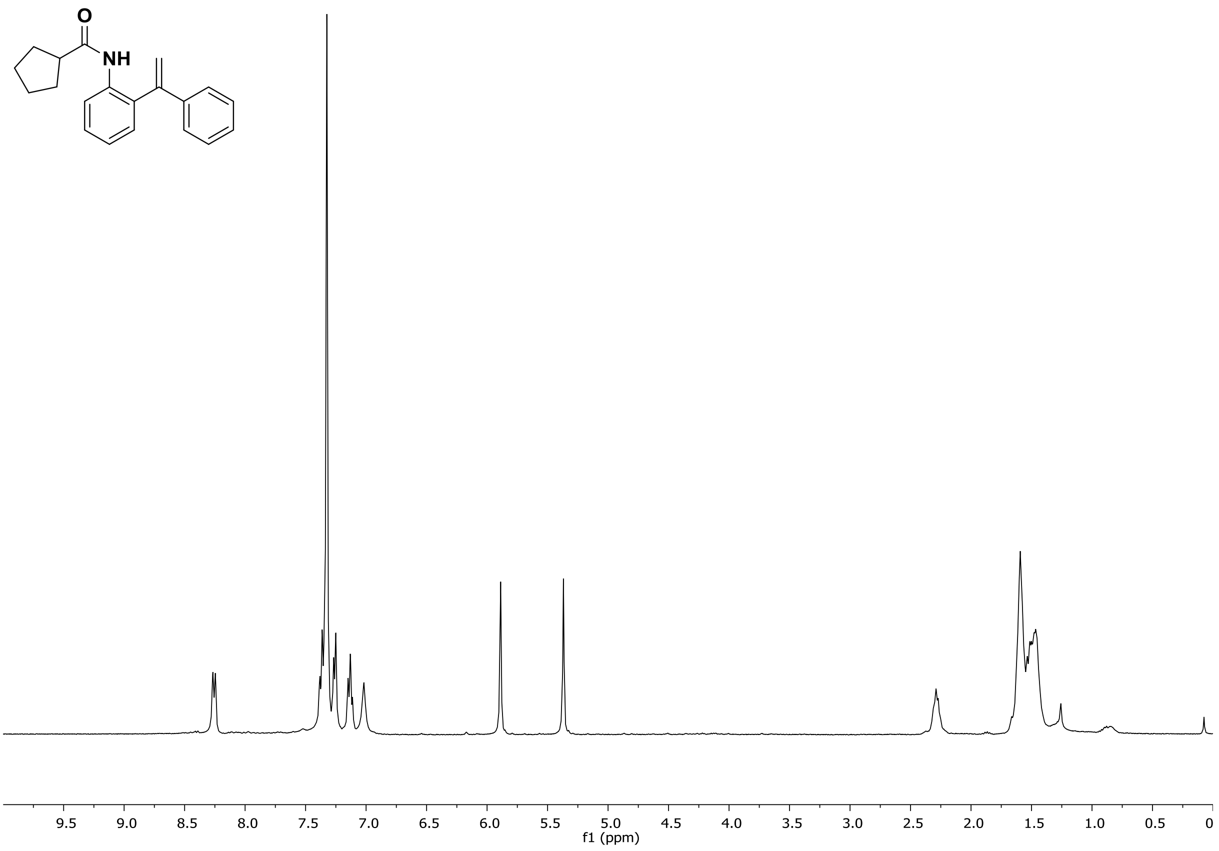
O
NH
9.5 9.0 8.5 8.0 7.5 7.0 6.5 6.0 5.5 5.0 4.5 4.0 3.5 3.0 2.5 2.0 1.5 1.0 0.5 0
f1 (ppm)

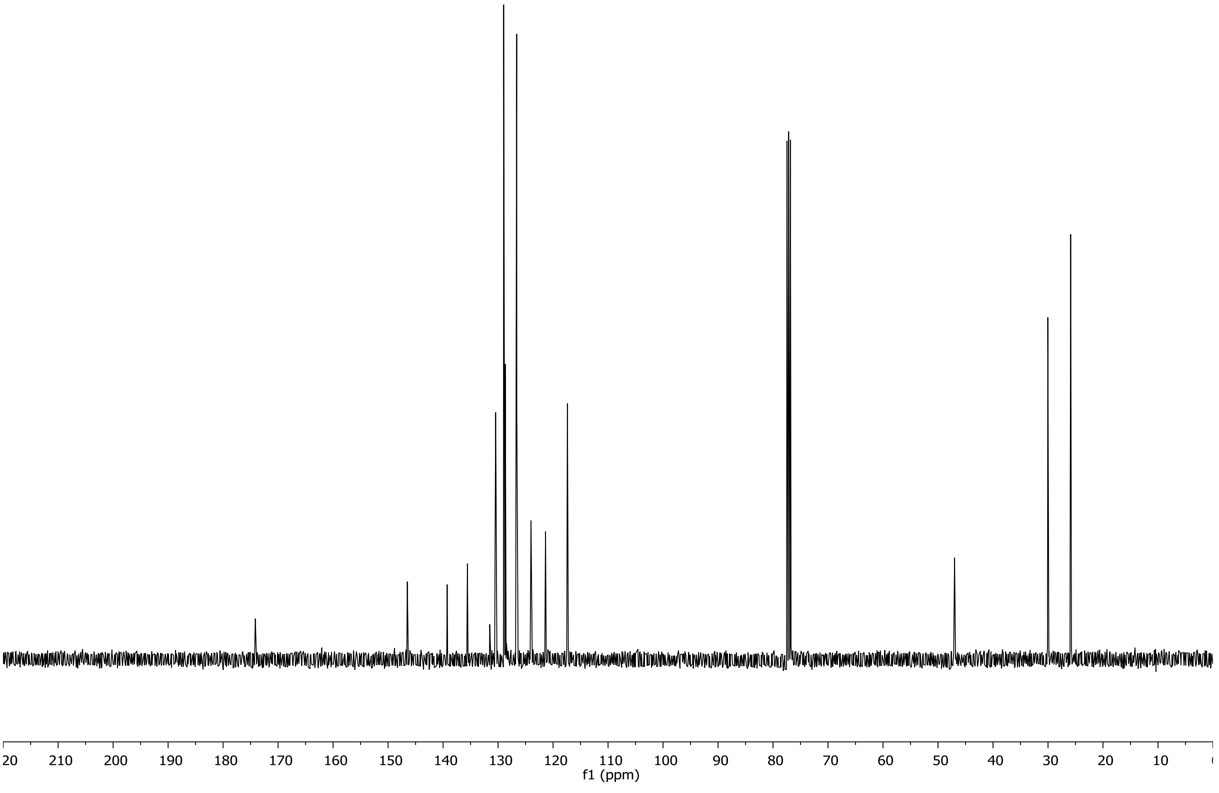
20 210 200 190 180 170 160 150 140 130 120 110 100 90 80 70 60 50 40 30 20 10
f1 (ppm)

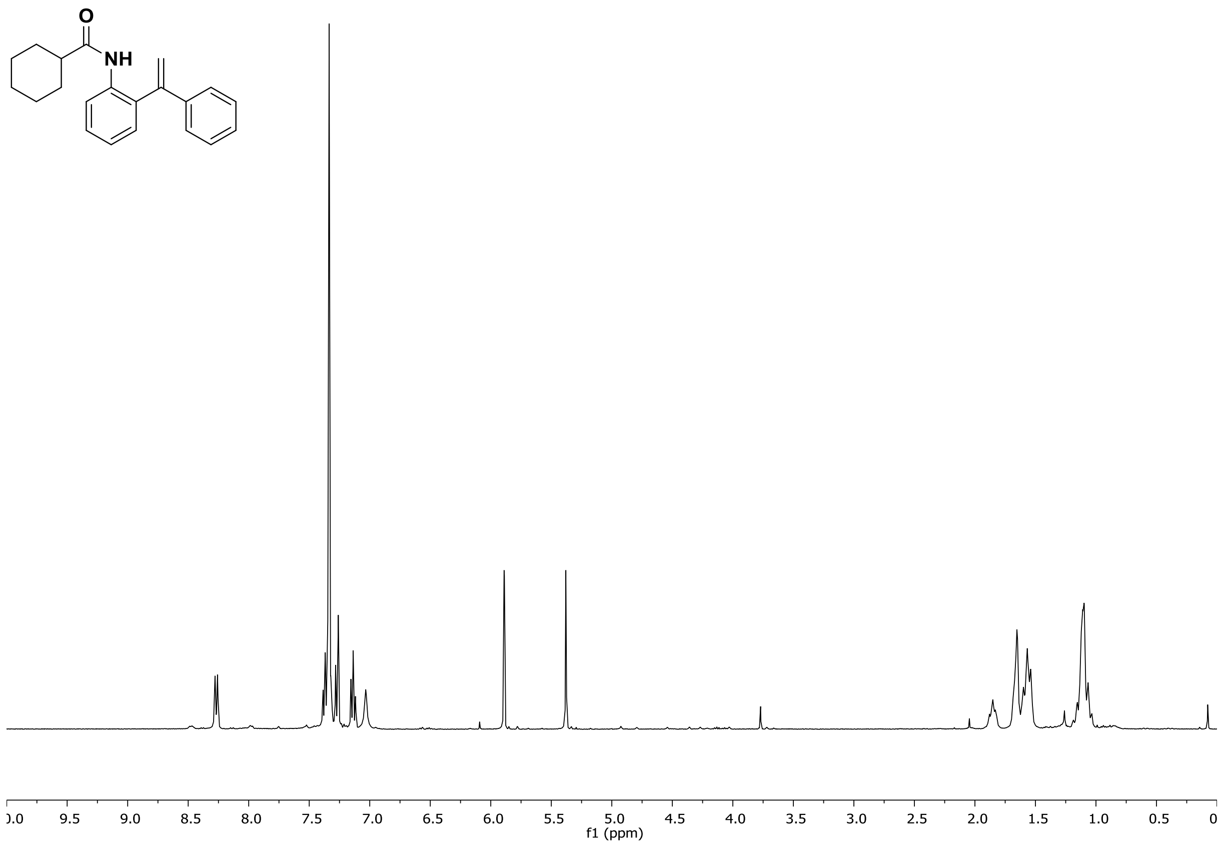
O
NH
f1 (ppm)

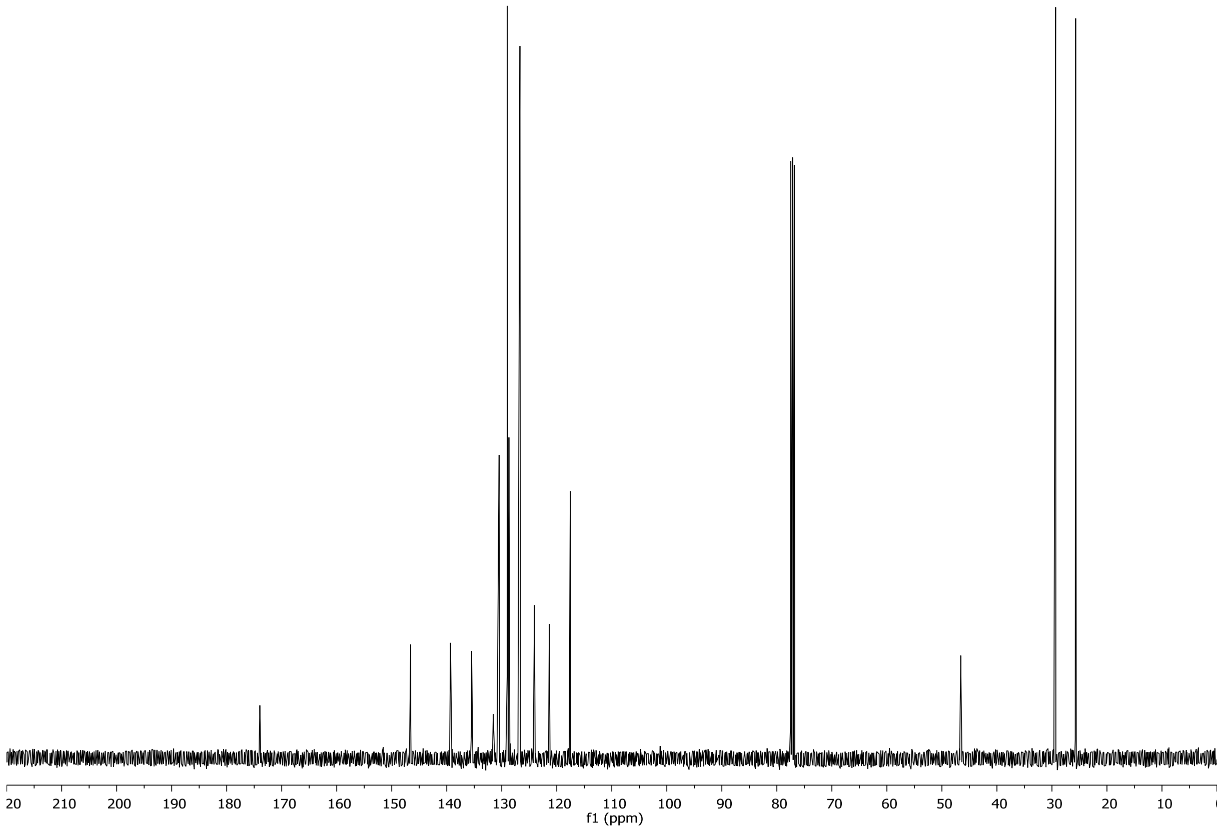
f1 (ppm)

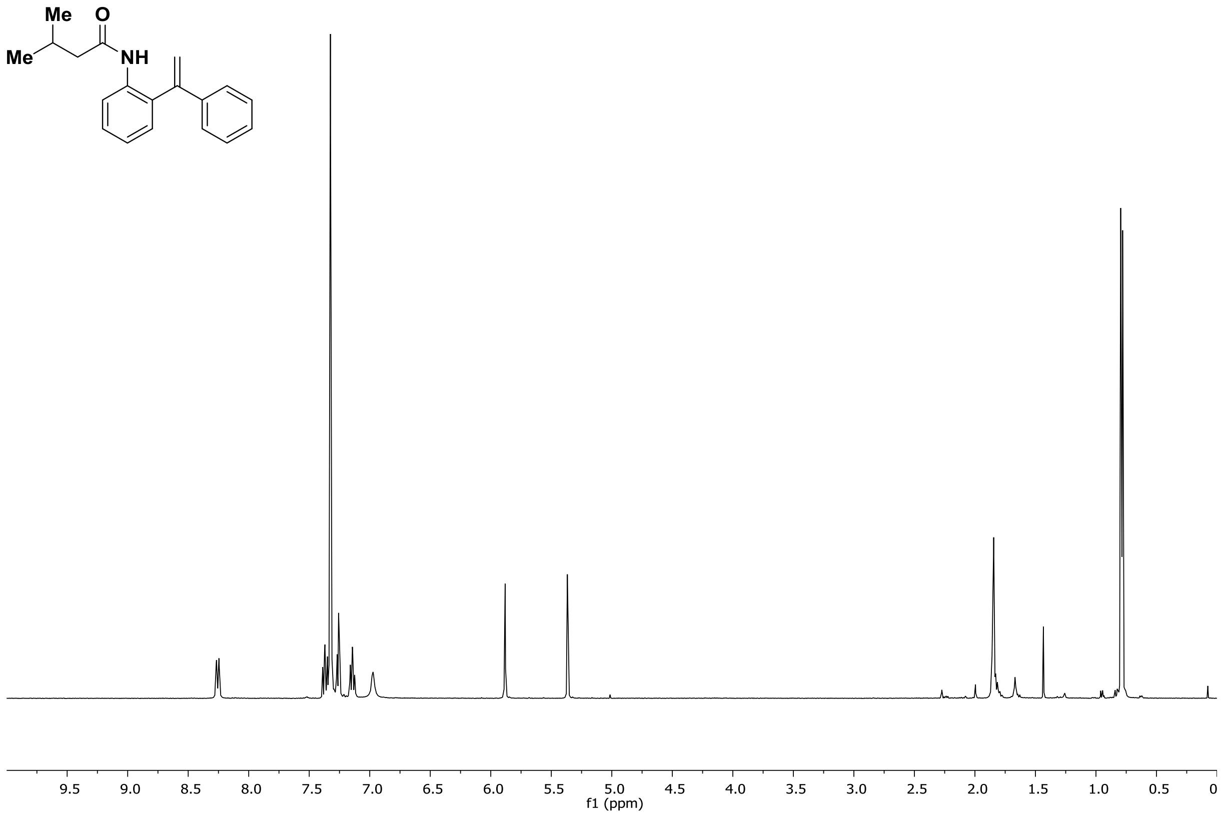
Me
O
Me
NH
9.5 9.0 8.5 8.0 7.5 7.0 6.5 6.0 5.5 5.0 4.5 4.0 3.5 3.0 2.5 2.0 1.5 1.0 0.5 0
f1 (ppm)

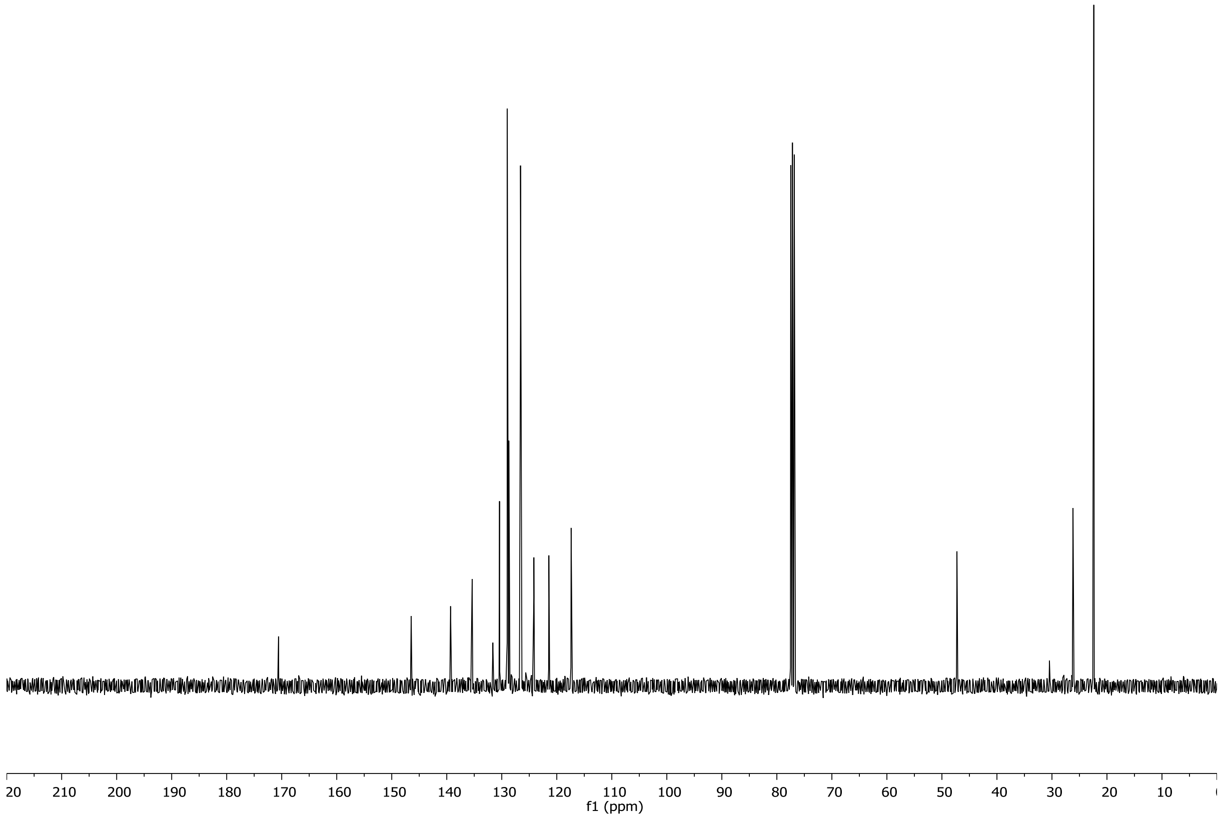
20 210 200 190 180 170 160 150 140 130 120 110 100 90 80 70 60 50 40 30 20 10
f1 (ppm)

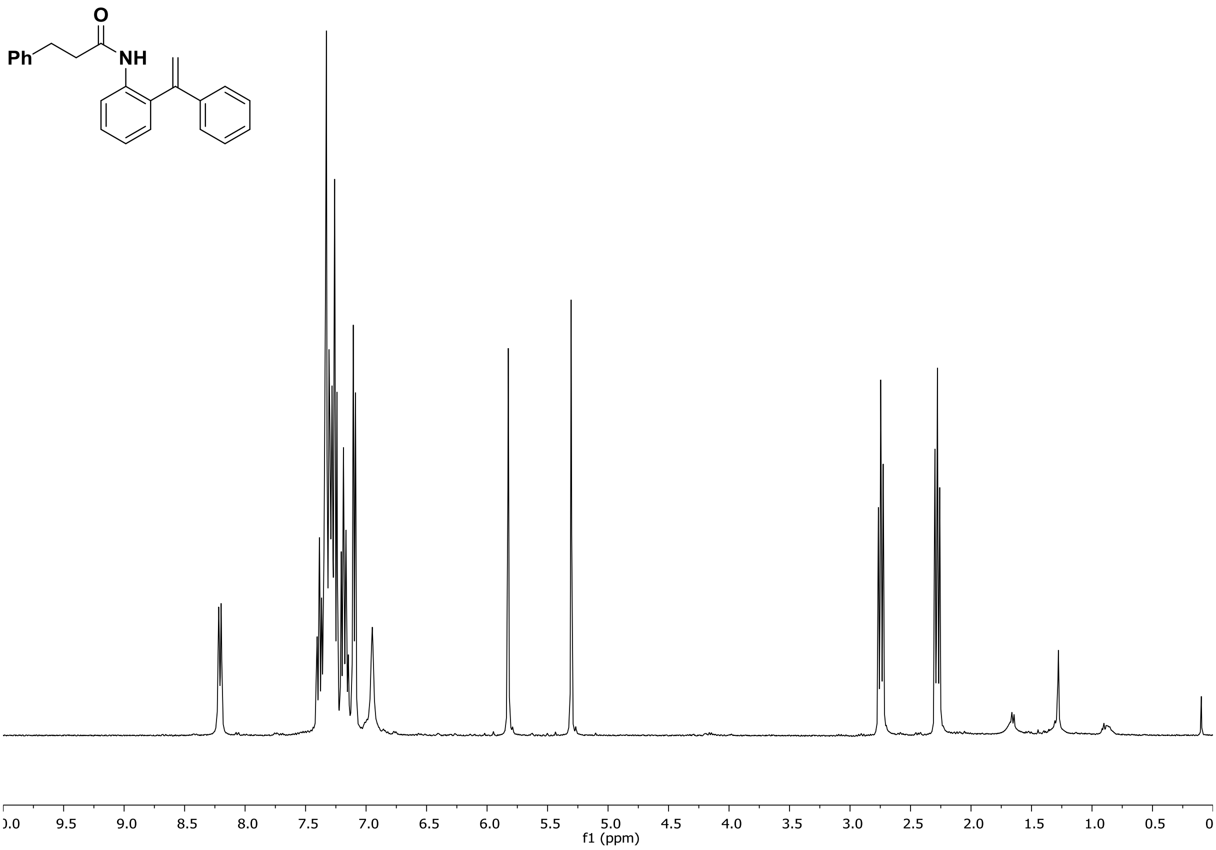
Ph
O
NH
0.0 9.5 9.0 8.5 8.0 7.5 7.0 6.5 6.0 5.5 5.0 4.5 4.0 3.5 3.0 2.5 2.0 1.5 1.0 0.5 0
f1 (ppm)

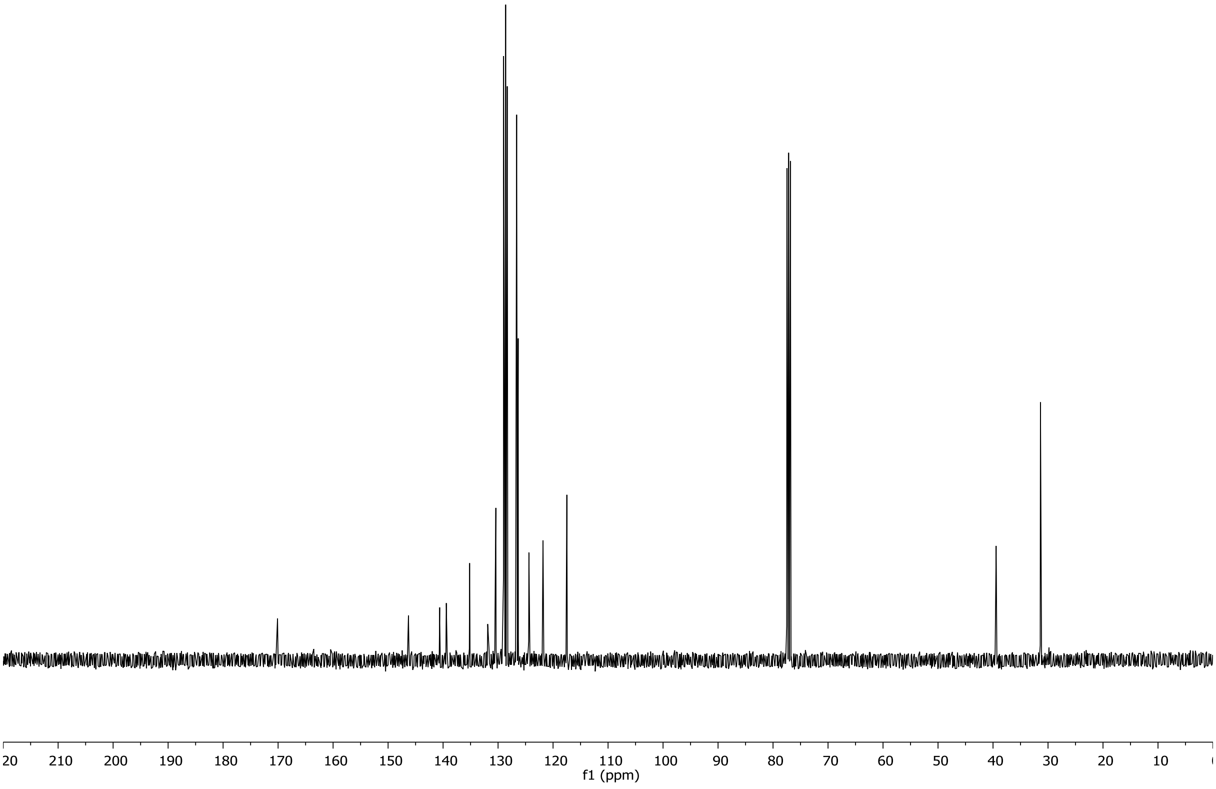
20 210 200 190 180 170 160 150 140 130 120 110 100 90 80 70 60 50 40 30 20 10
f1 (ppm)

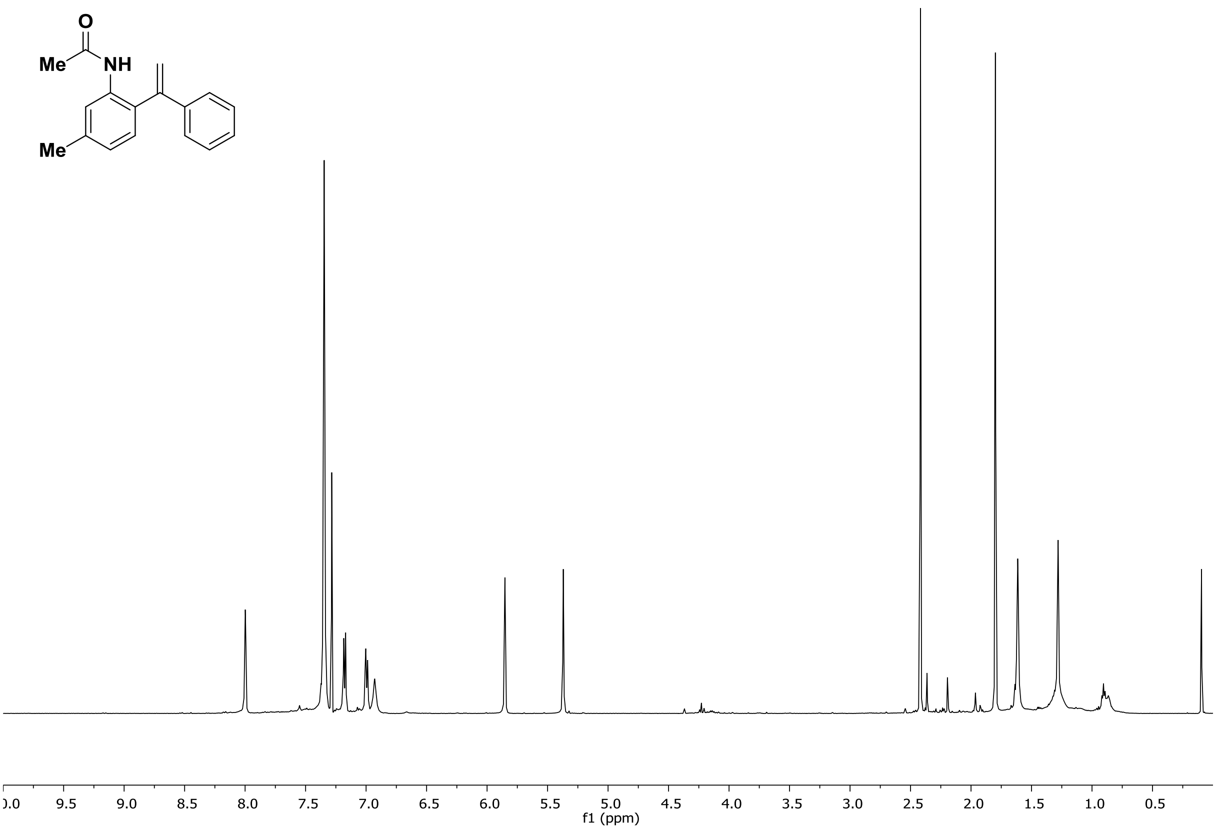
O
NH
Me
Me
0.0 9.5 9.0 8.5 8.0 7.5 7.0 6.5 6.0 5.5 5.0 4.5 4.0 3.5 3.0 2.5 2.0 1.5 1.0 0.5
f1 (ppm)

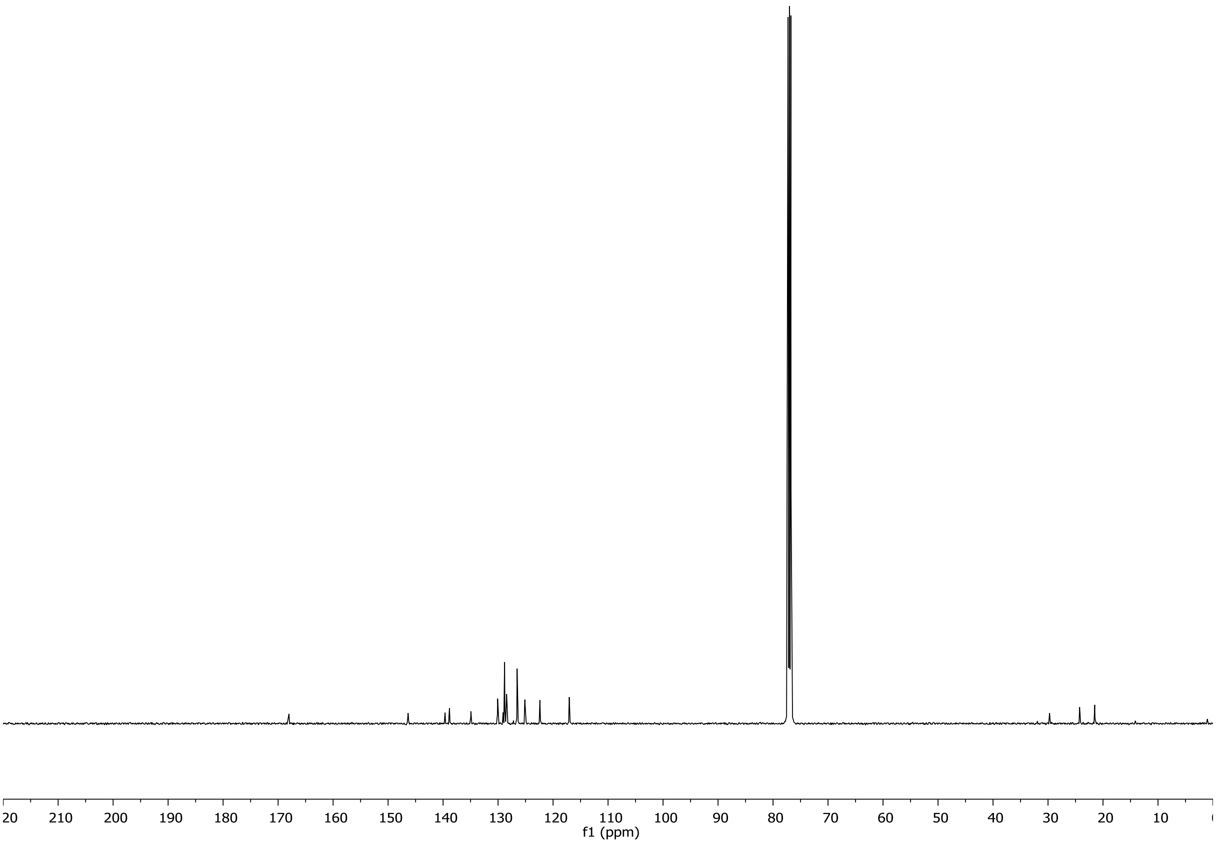
20 210 200 190 180 170 160 150 140 130 120 110 100 90 80 70 60 50 40 30 20 10
f1 (ppm)

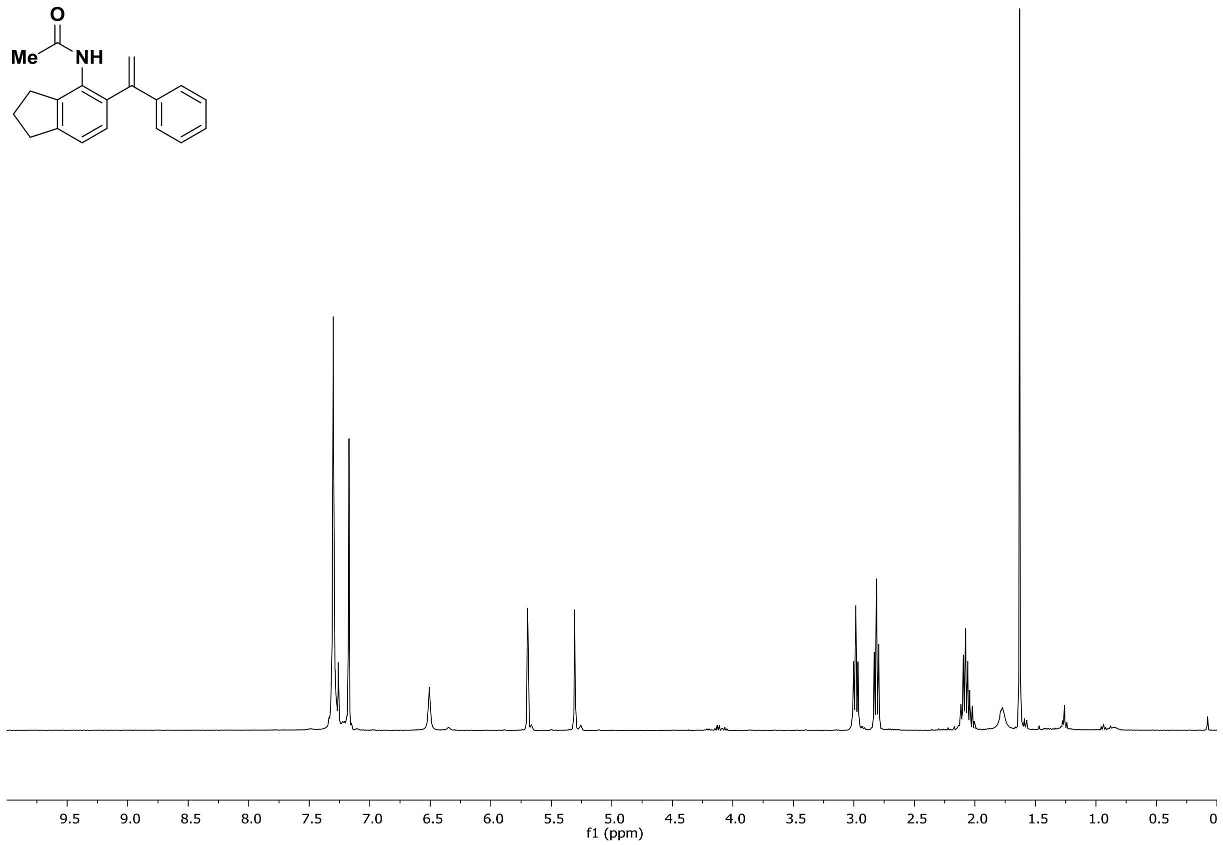
Me
O
NH
9.5 9.0 8.5 8.0 7.5 7.0 6.5 6.0 5.5 5.0 4.5 4.0 3.5 3.0 2.5 2.0 1.5 1.0 0.5 0
f1 (ppm)

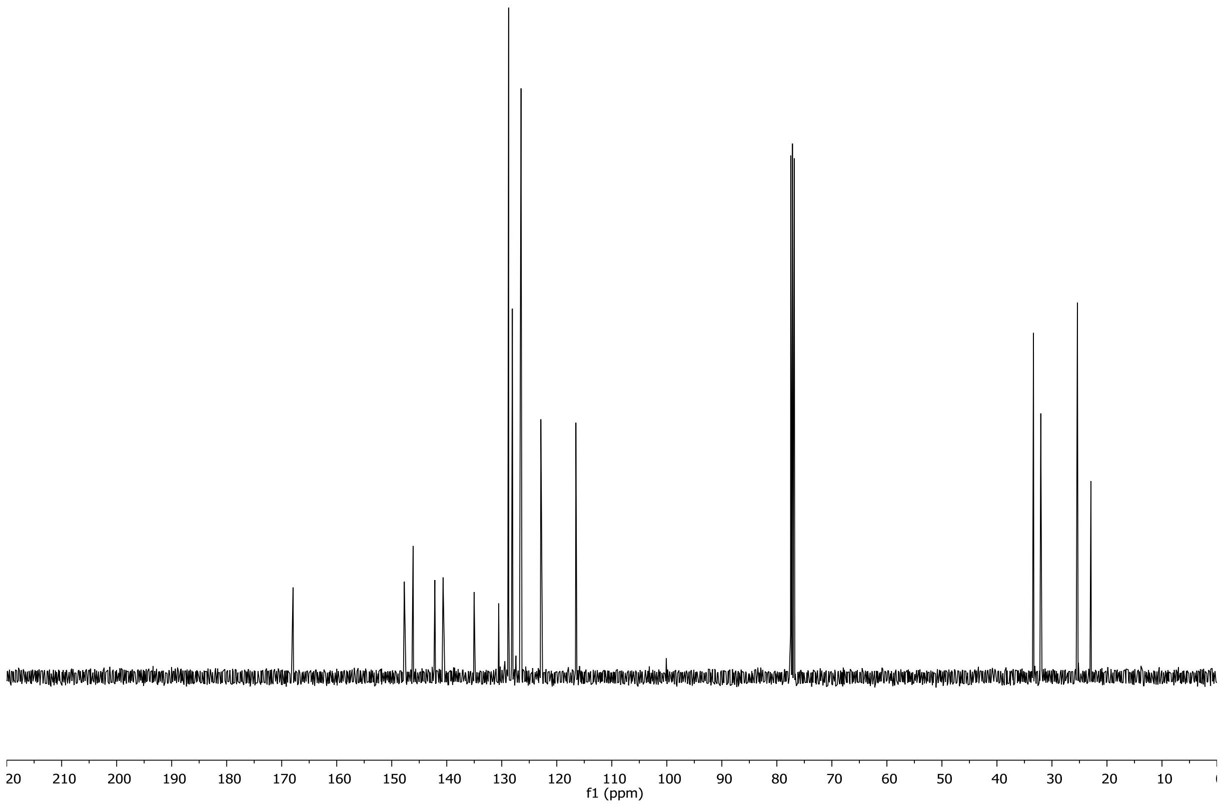
20 210 200 190 180 170 160 150 140 130 120 110 100 90 80 70 60 50 40 30 20 10
f1 (ppm)

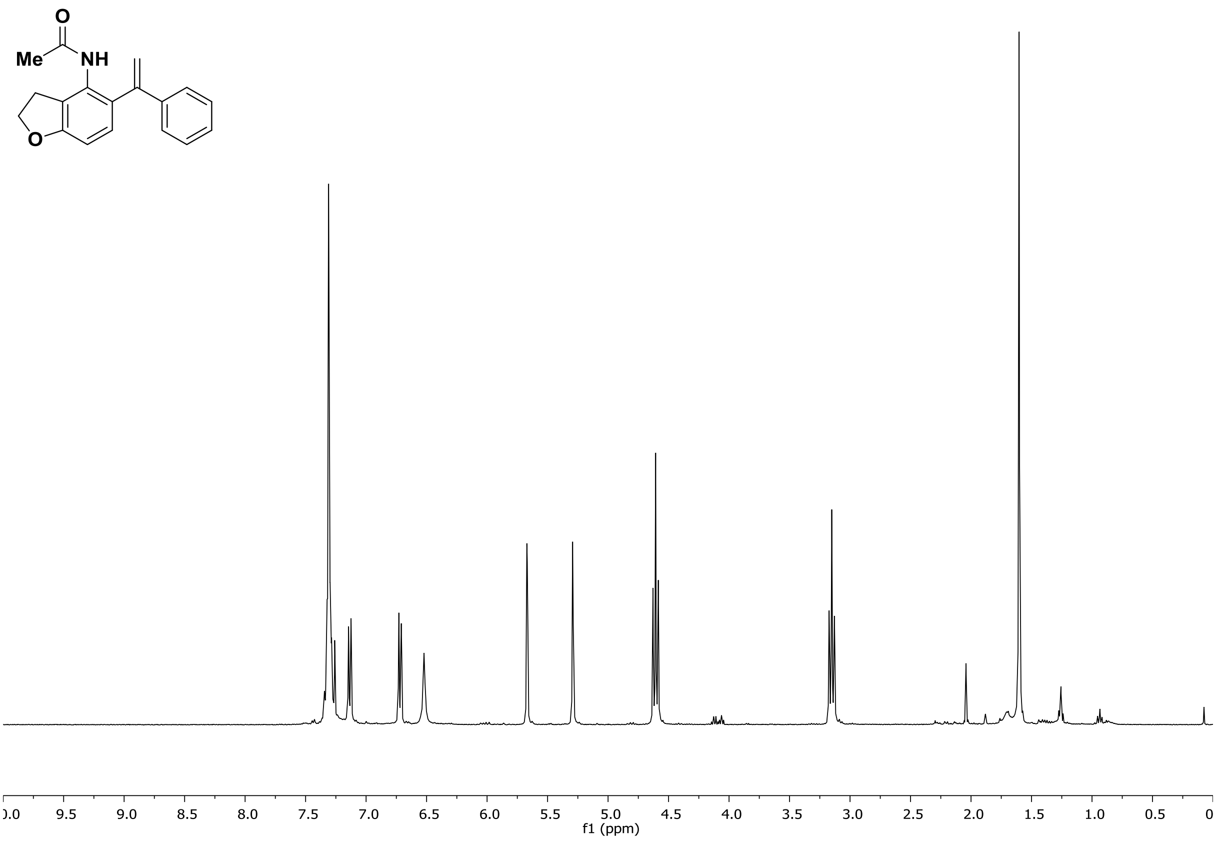
O
Me
NH
O
9.5 9.0 8.5 8.0 7.5 7.0 6.5 6.0 5.5 5.0 4.5 4.0 3.5 3.0 2.5 2.0 1.5 1.0 0.5
f1 (ppm)

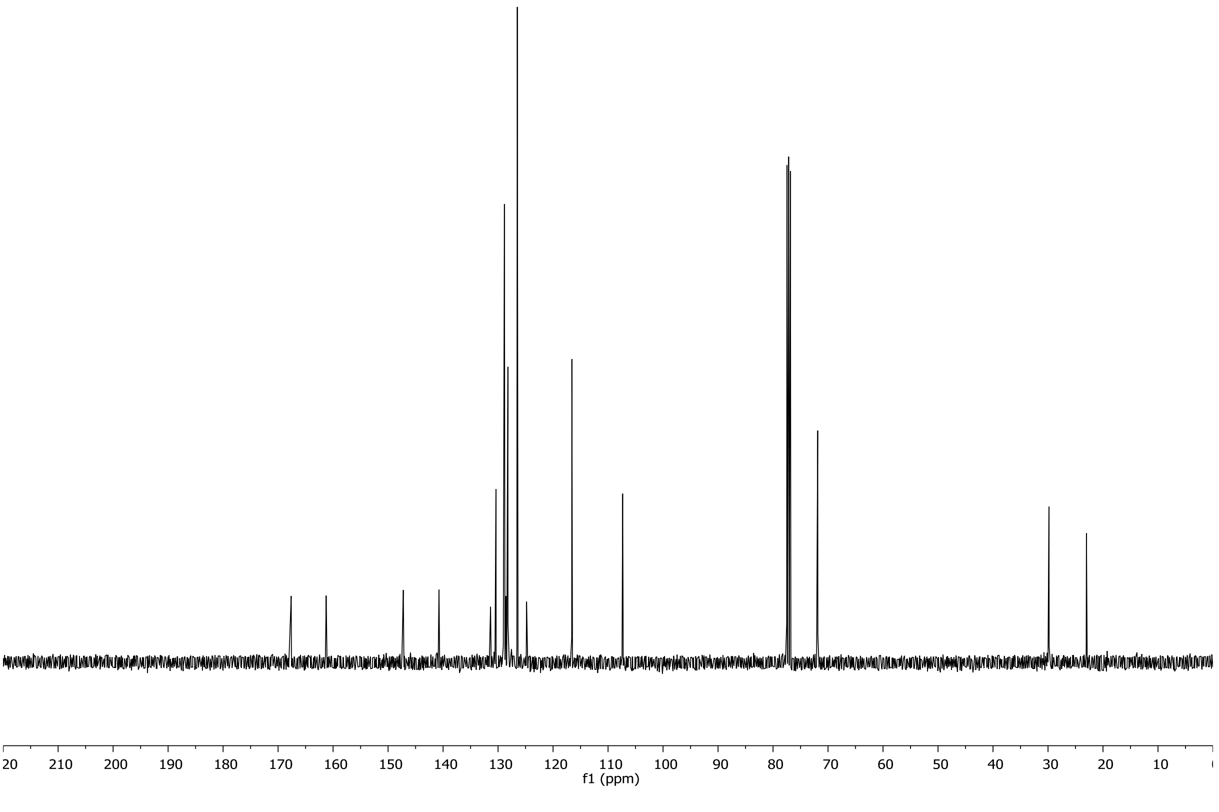
210 200 190 180 170 160 150 140 130 120 110 100 90 80 70 60 50 40 30 20 10
f1 (ppm)

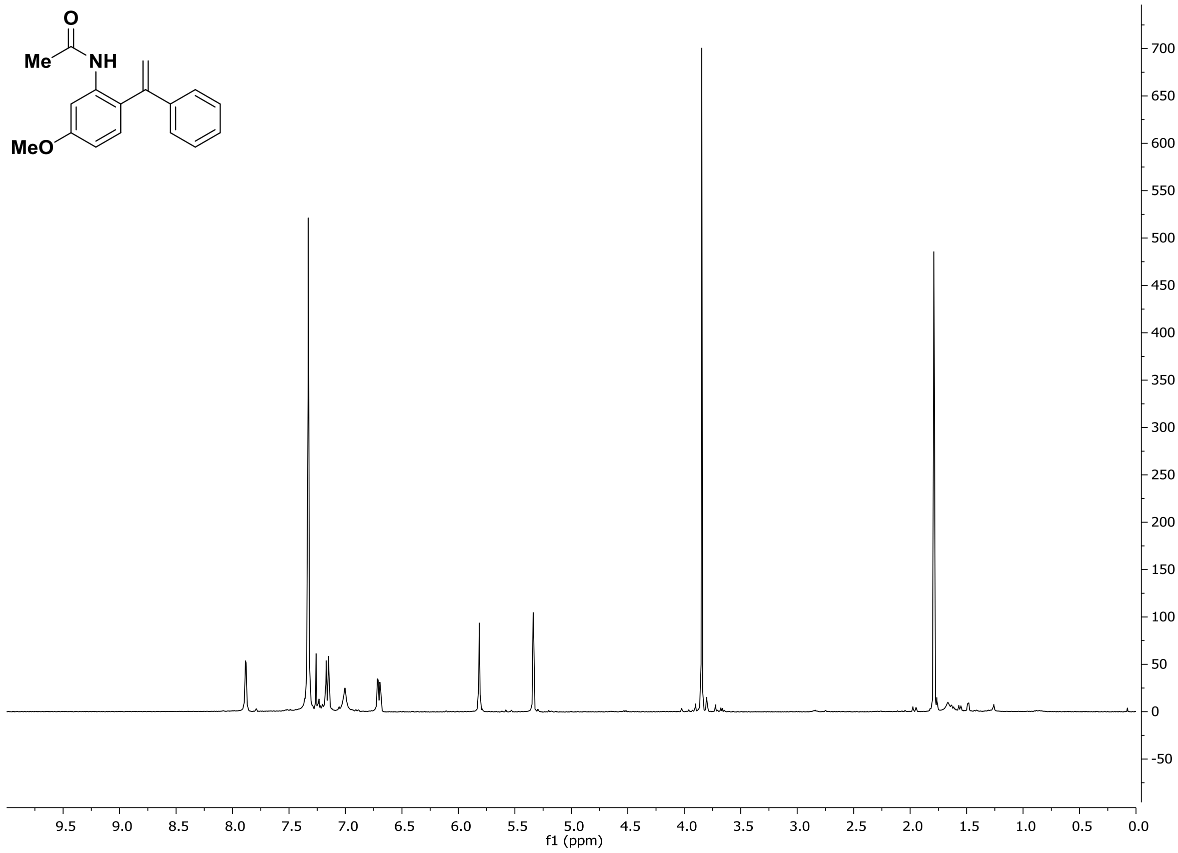
O
Me
NH
MeO
f1 (ppm)

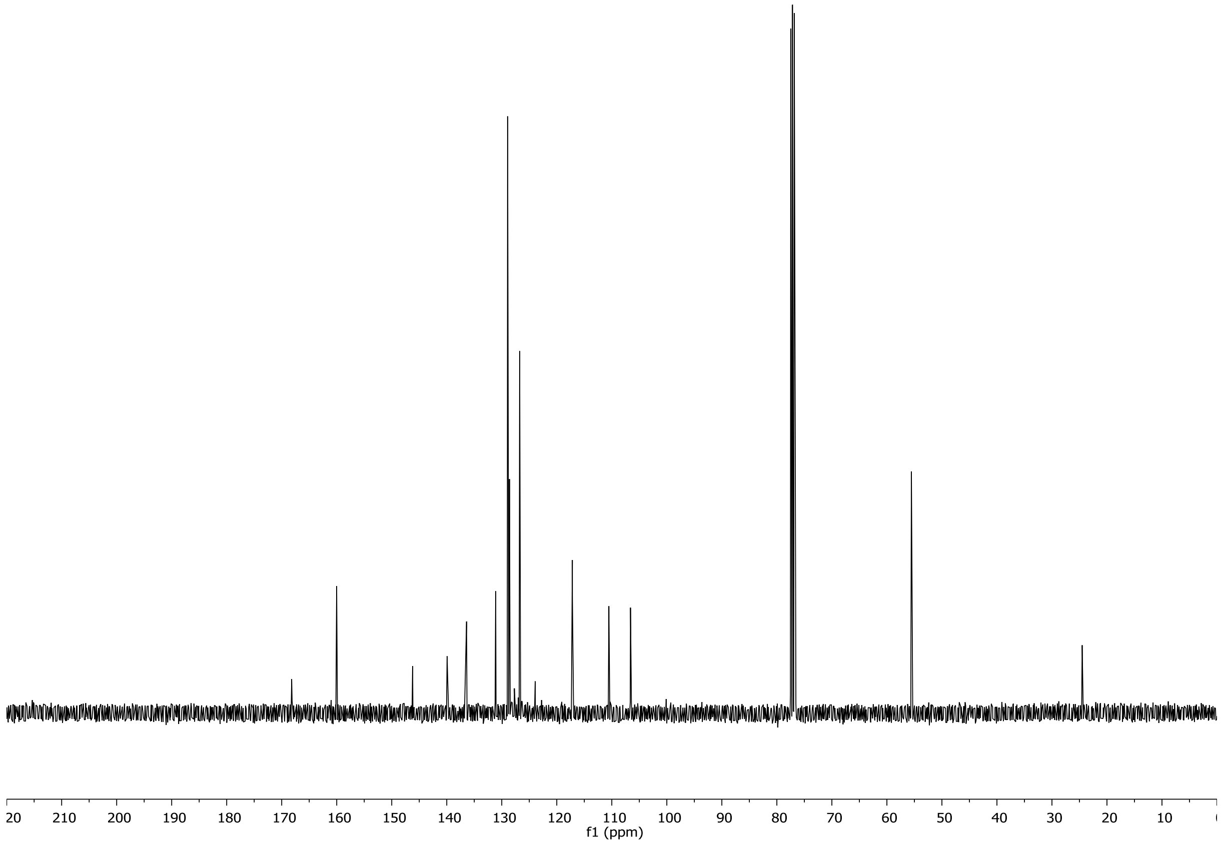
f1 (ppm)

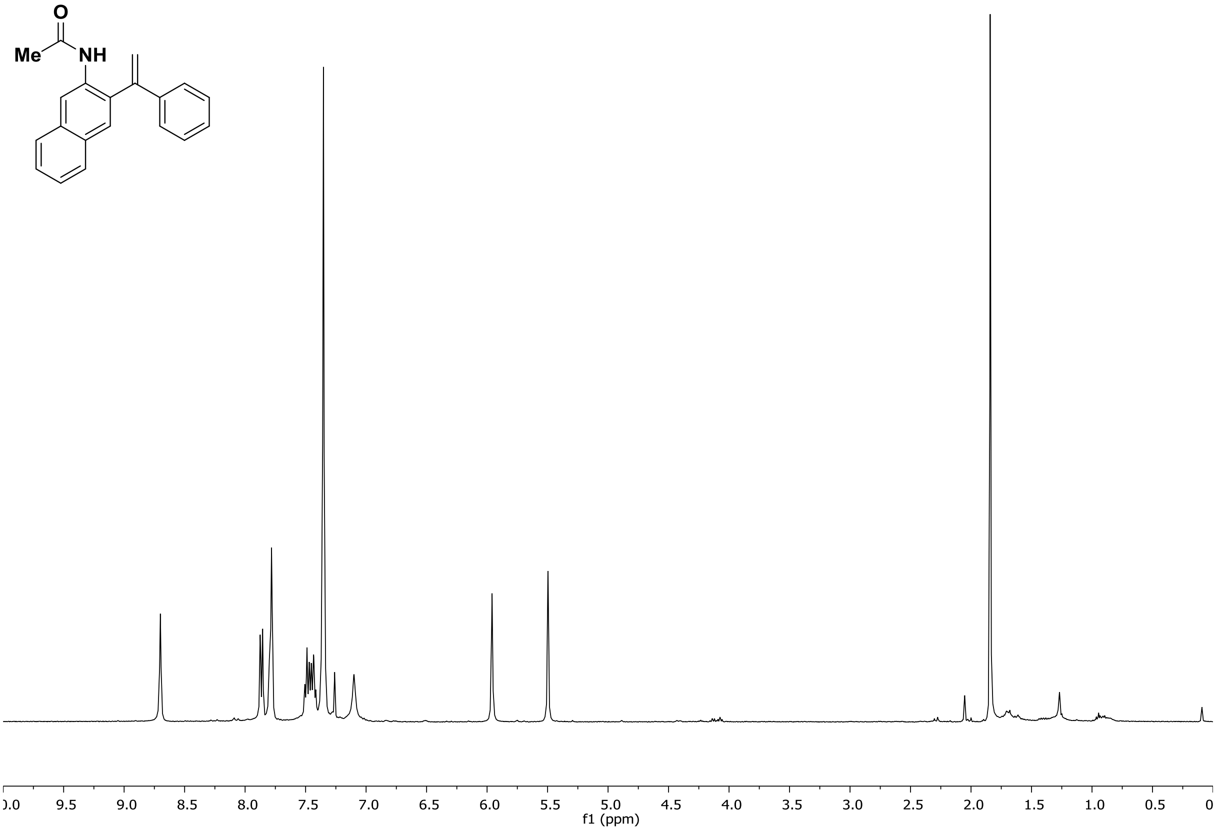
O
Me
NH
f1 (ppm)

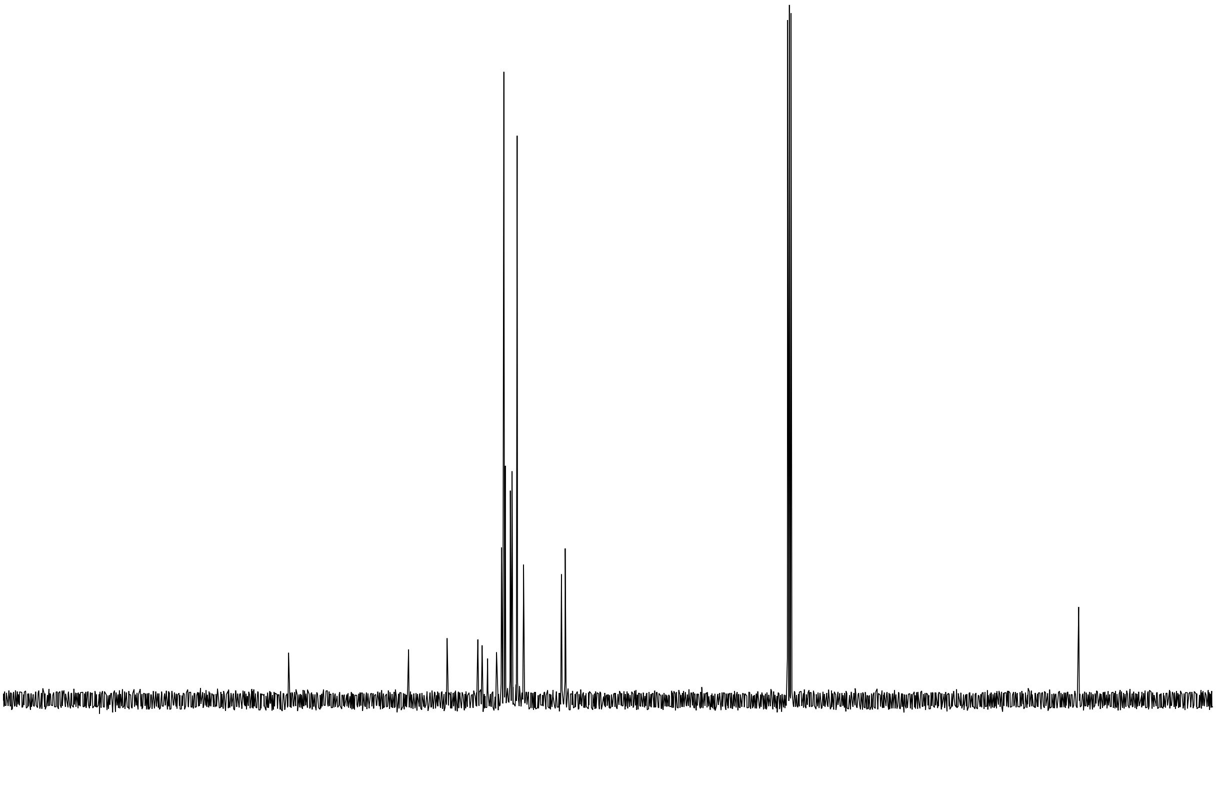
f1 (ppm)

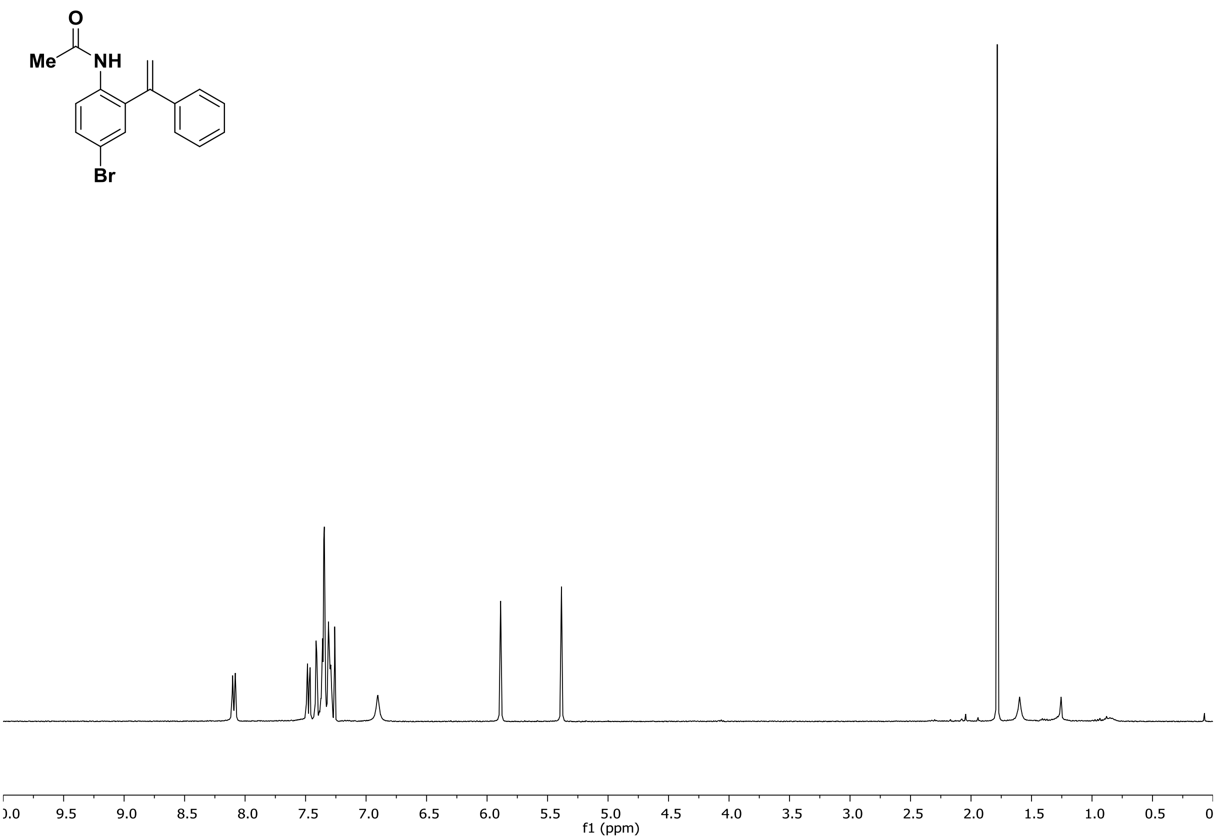
O
Me
NH
Br
f1 (ppm)

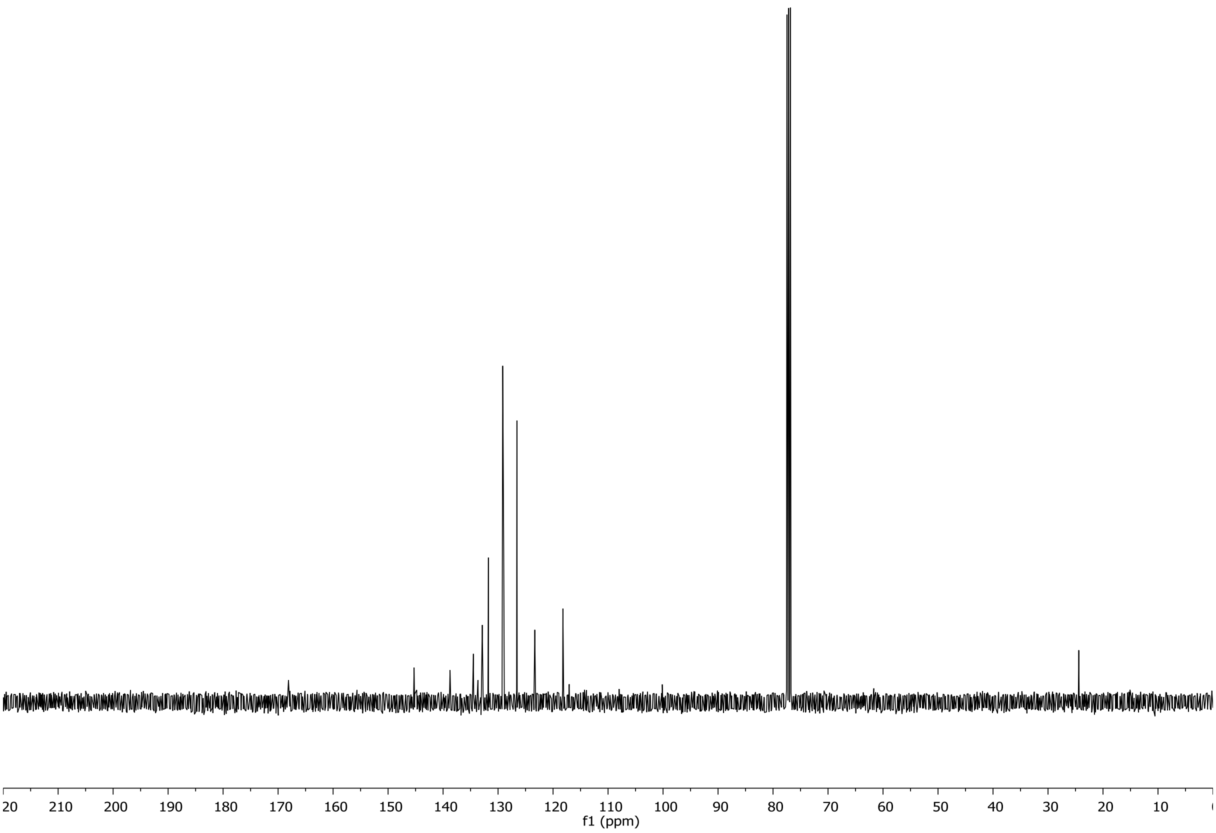
f1 (ppm)

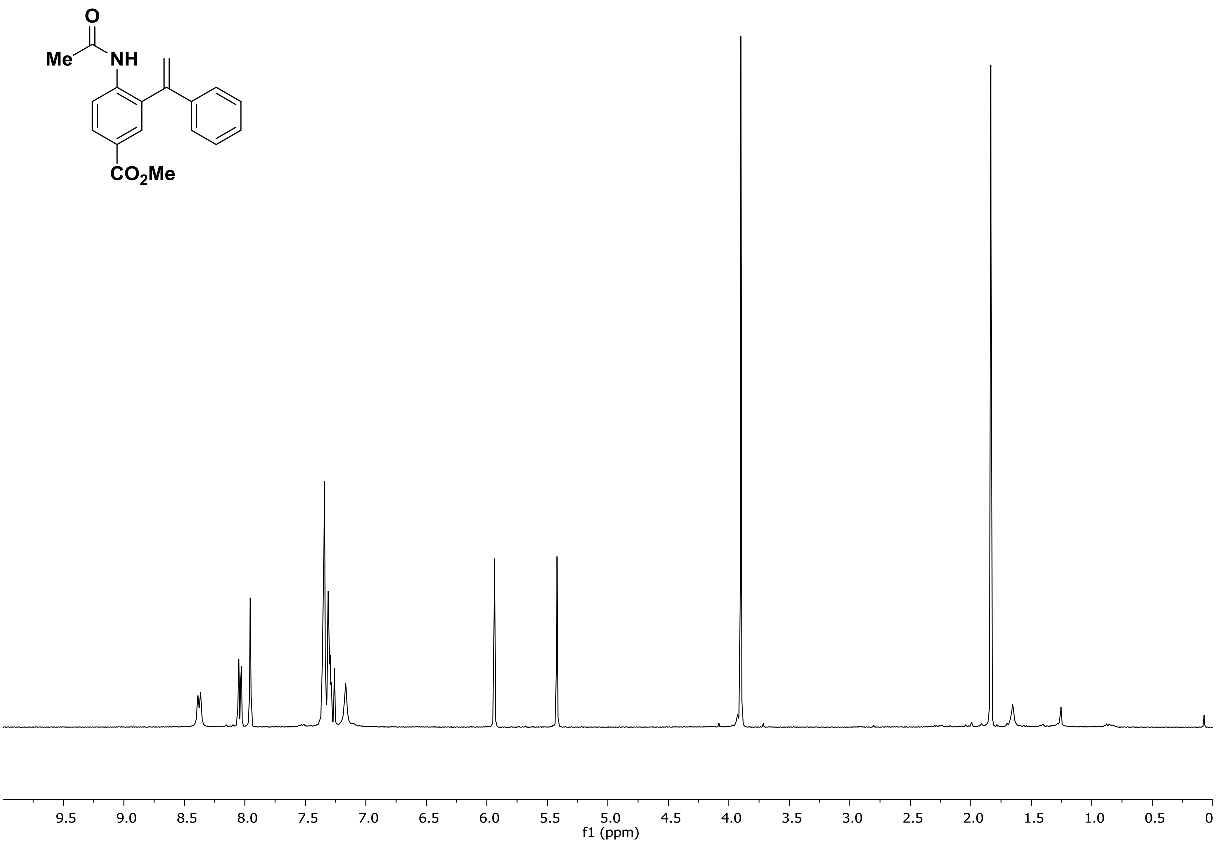
O
Me
NH
CO2Me
9.5 9.0 8.5 8.0 7.5 7.0 6.5 6.0 5.5 5.0 4.5 4.0 3.5 3.0 2.5 2.0 1.5 1.0 0.5 0
f1 (ppm)

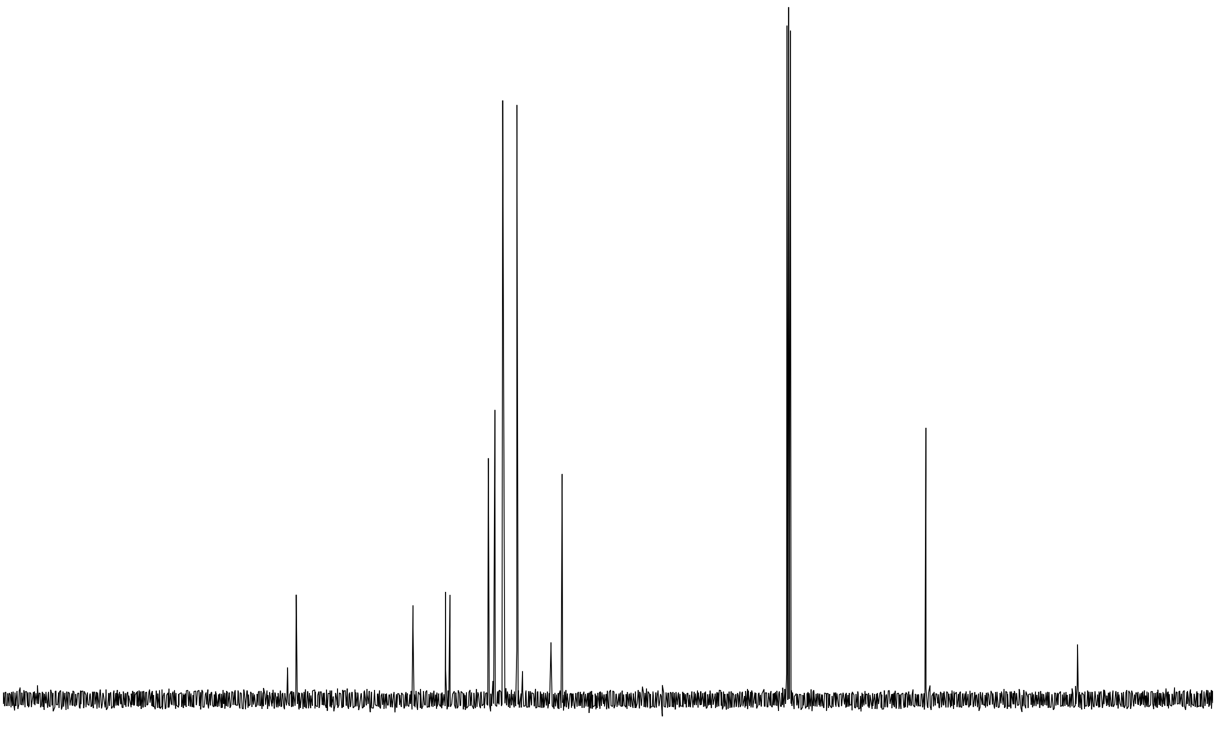
20 210 200 190 180 170 160 150 140 130 120 110 100 90 80 70 60 50 40 30 20 10
f1 (ppm)

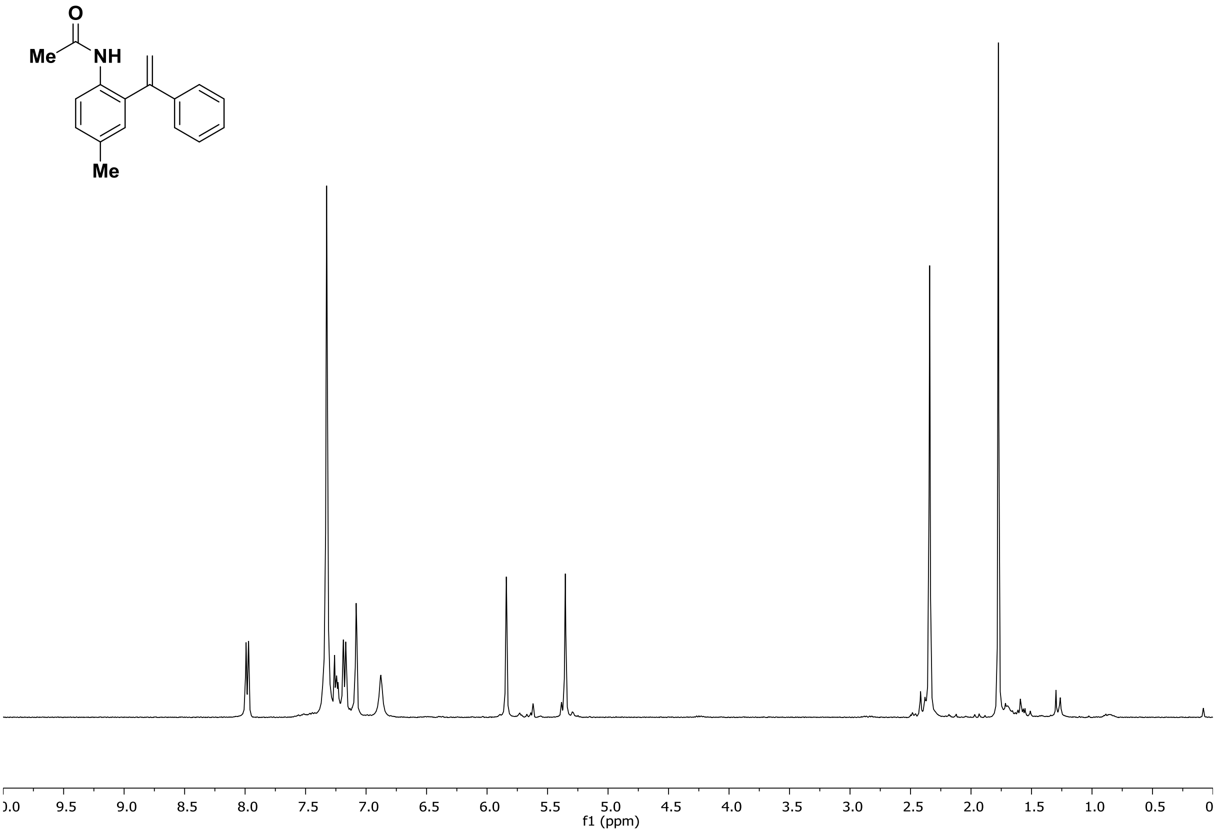
O
Me
NH
Me
f1 (ppm)

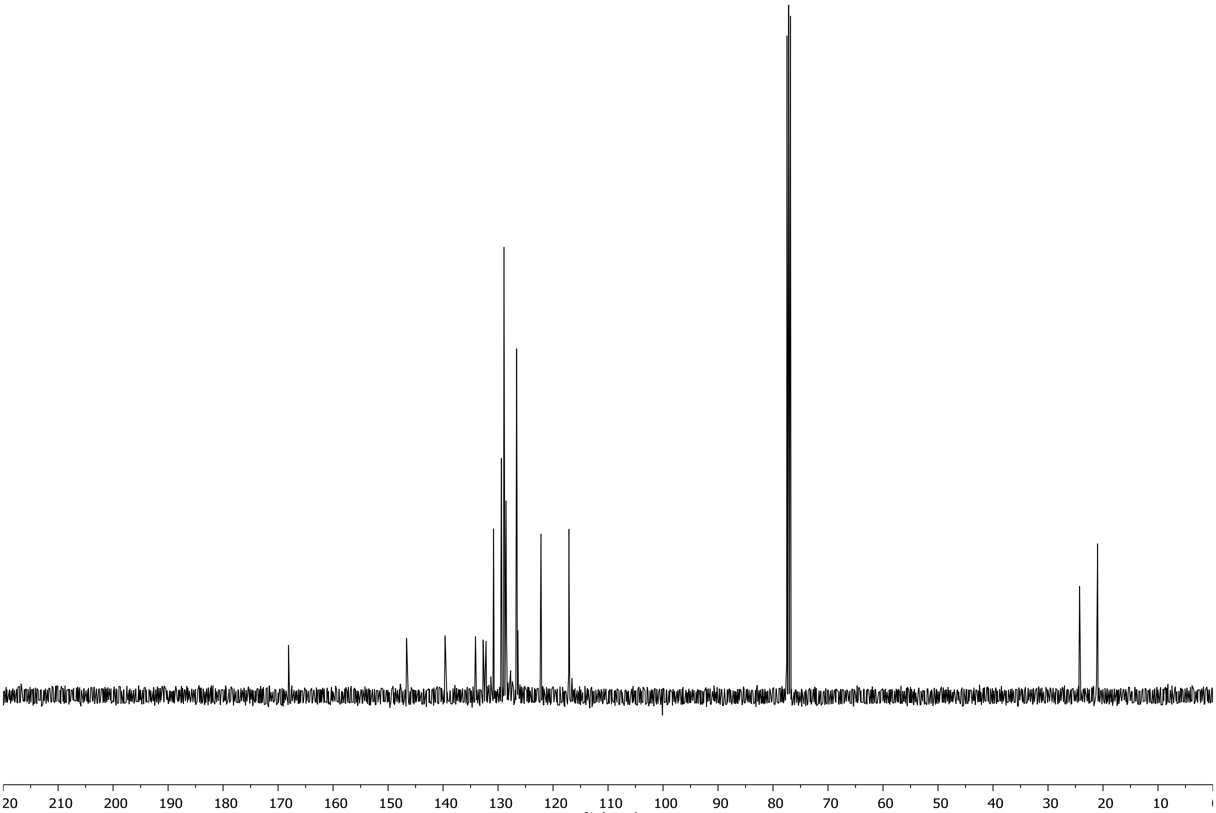
f1 (ppm)

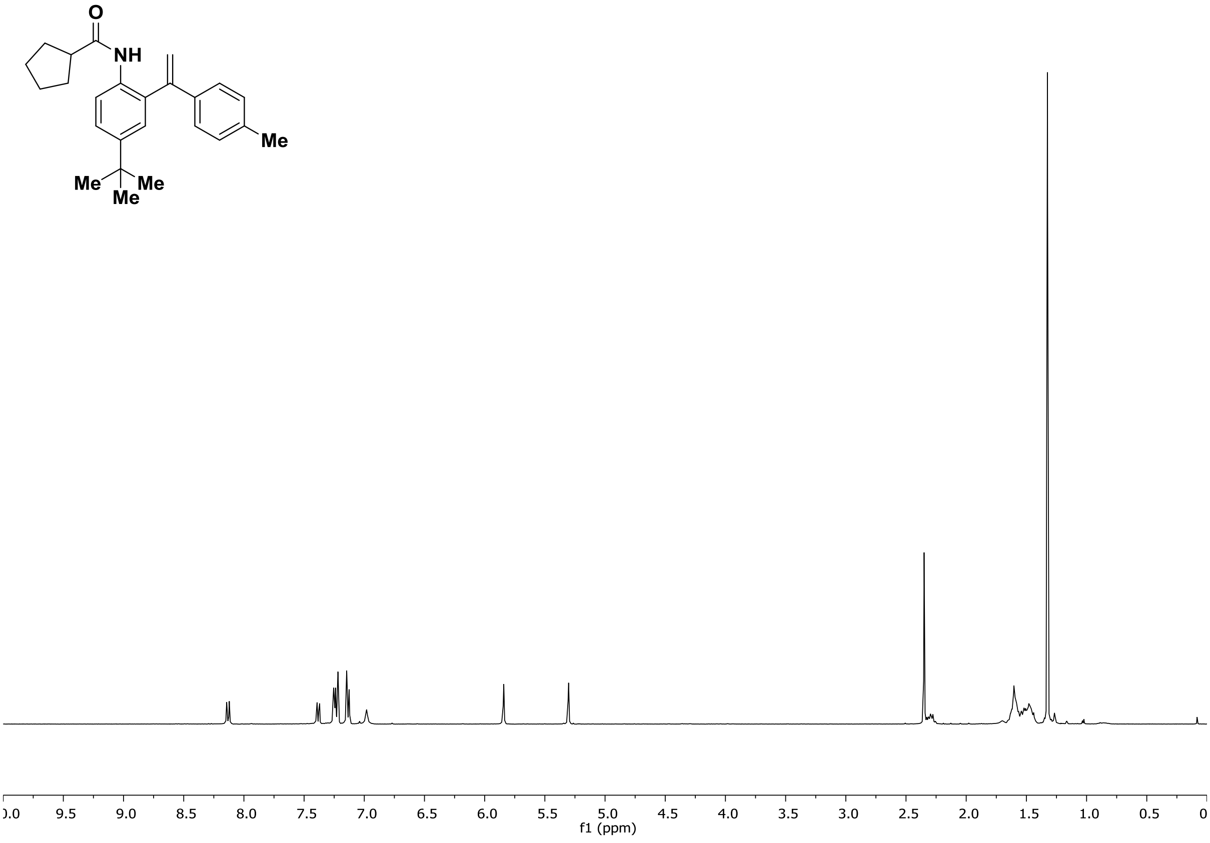
O
NH
Me
Me
Me
Me
).0 9.5 9.0 8.5 8.0 7.5 7.0 6.5 6.0 5.5 5.0 4.5 4.0 3.5 3.0 2.5 2.0 1.5 1.0 0.5 0
f1 (ppm)

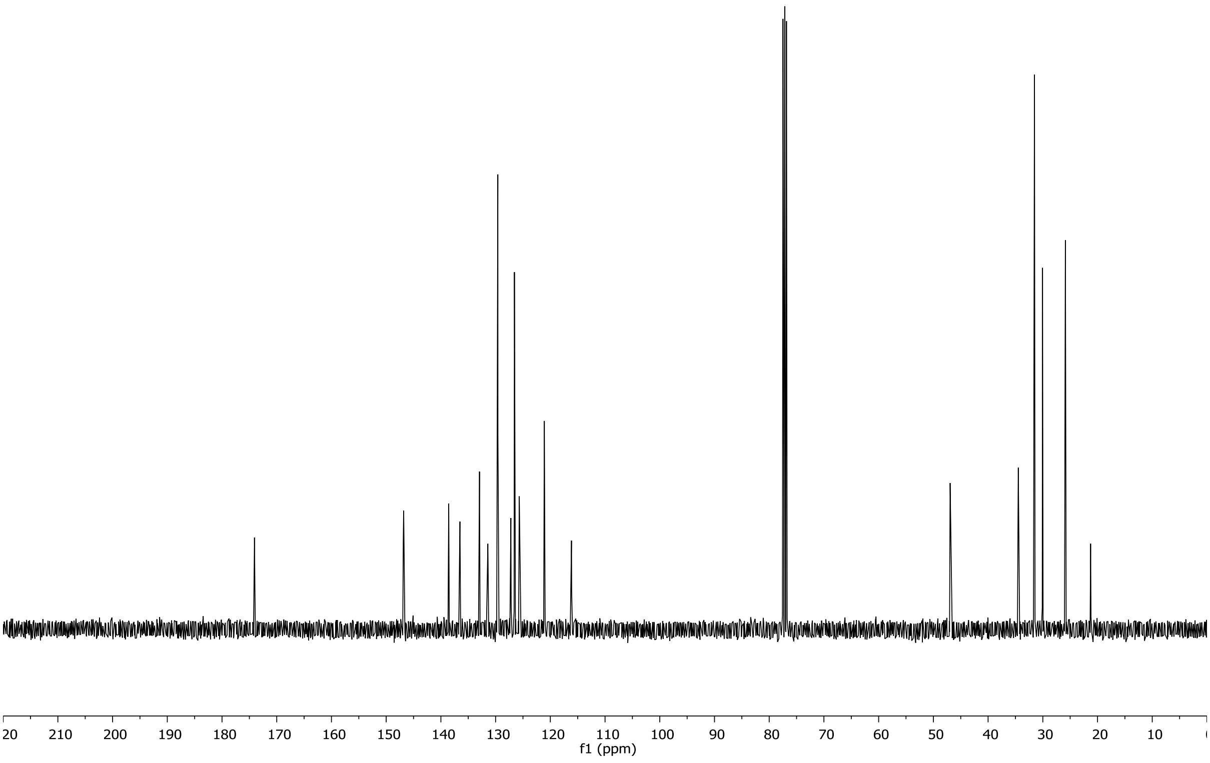
20 210 200 190 180 170 160 150 140 130 120 110 100 90 80 70 60 50 40 30 20 10
f1 (ppm)

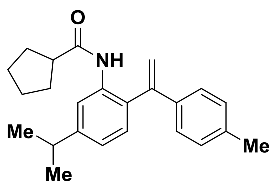
O
NH
Me
Me
Me

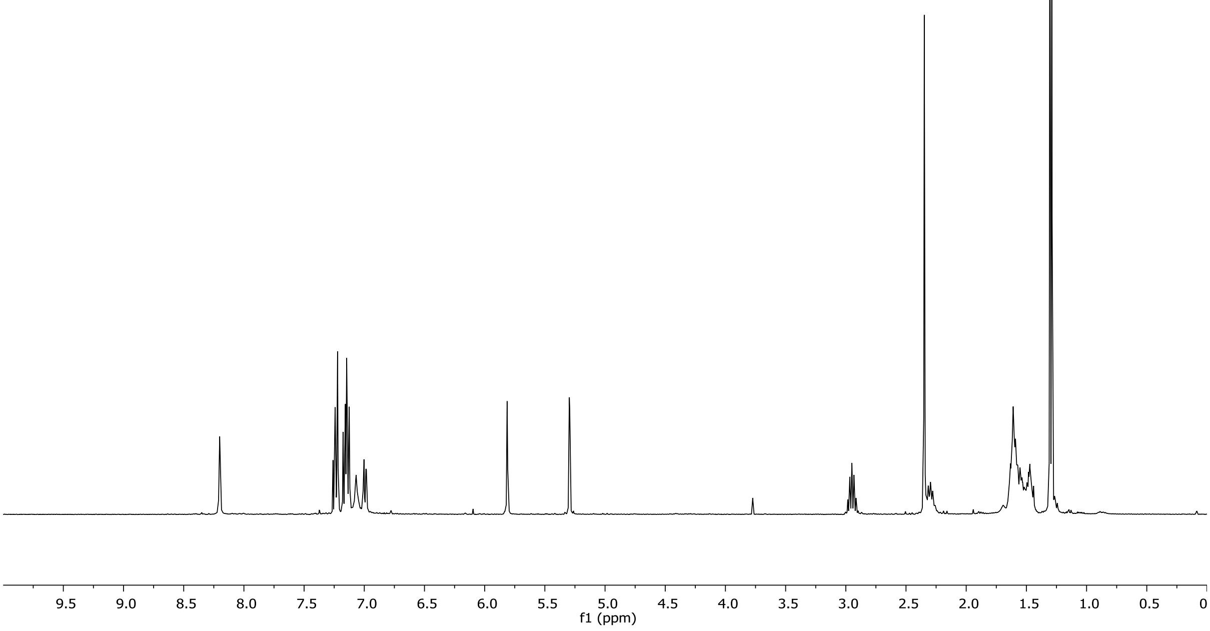
f1 (ppm)

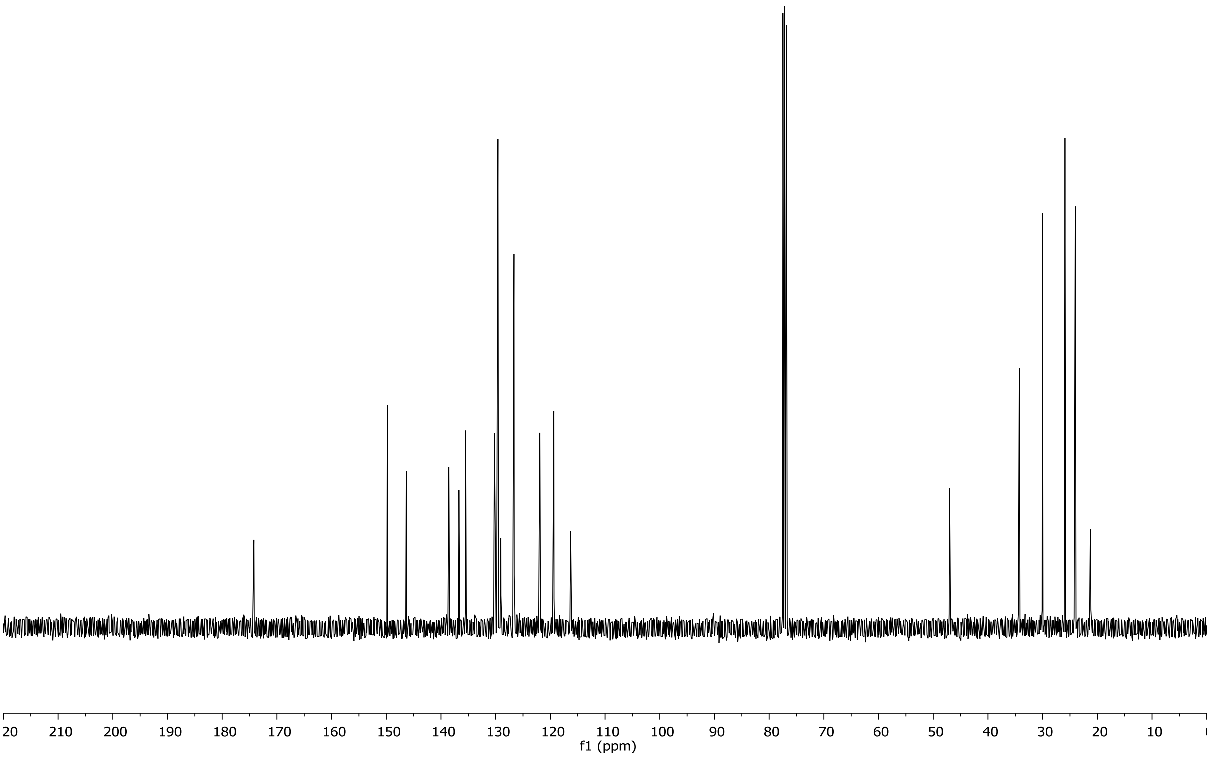
f1 (ppm)

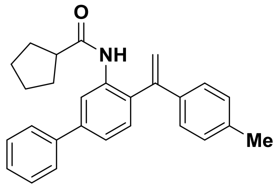
O
NH
Me

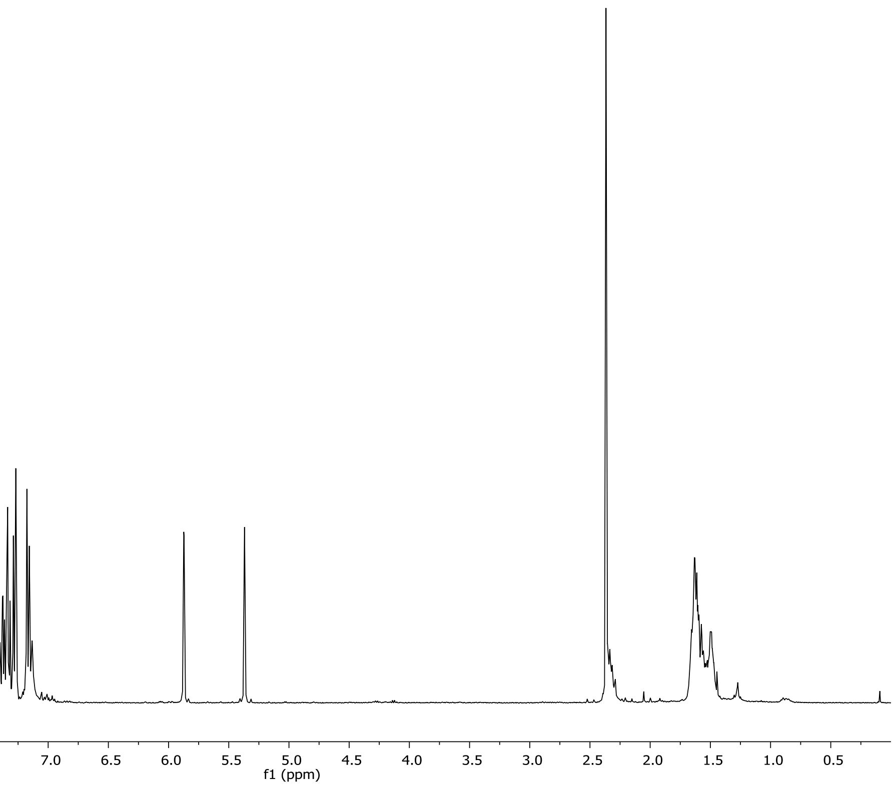
f1 (ppm)

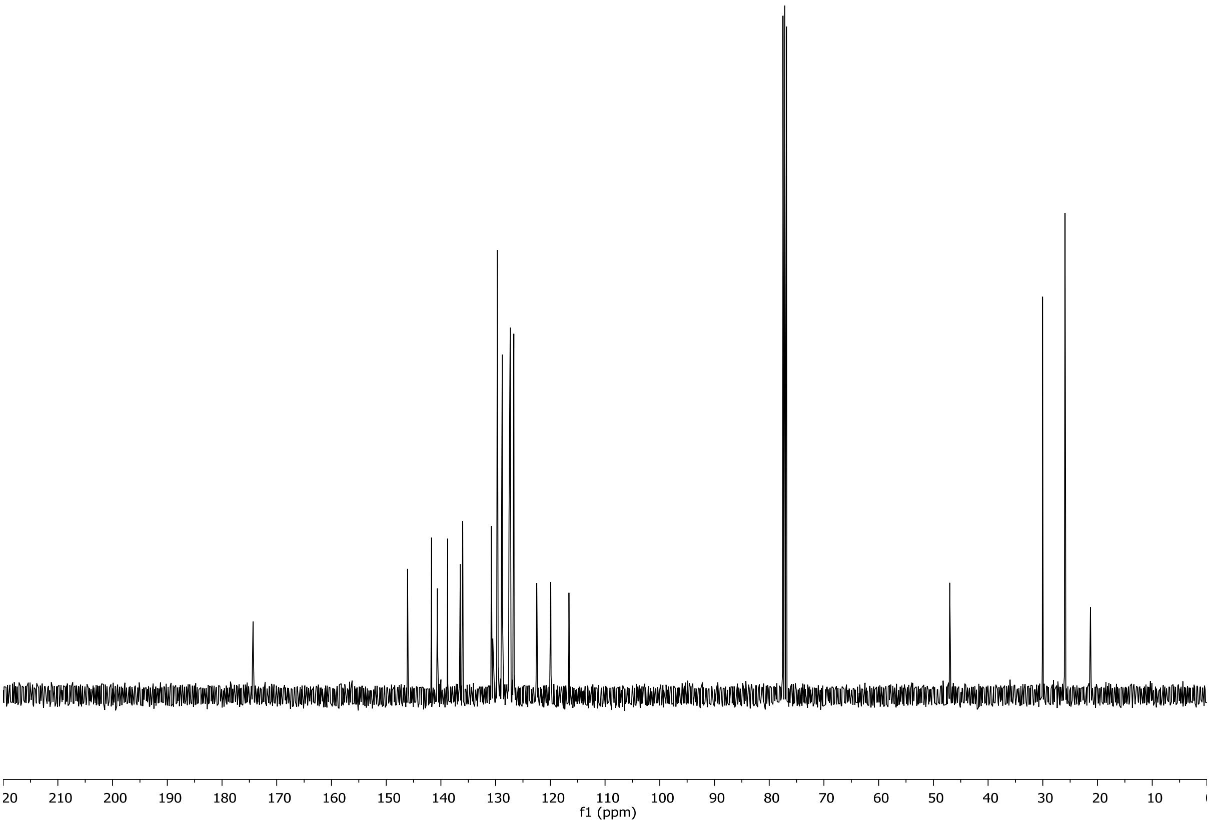
f1 (ppm)

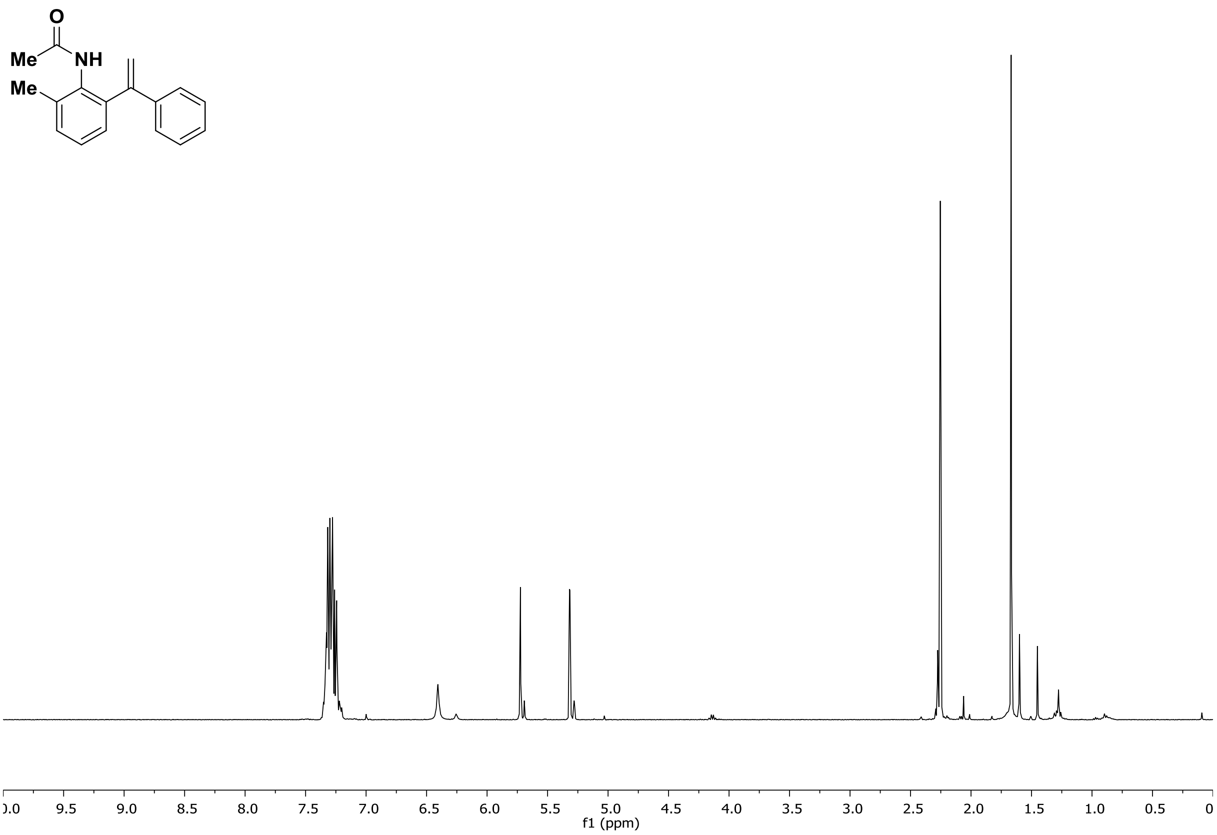
O
Me
NH
Me
).0 9.5 9.0 8.5 8.0 7.5 7.0 6.5 6.0 5.5 5.0 4.5 4.0 3.5 3.0 2.5 2.0 1.5 1.0 0.5 0
f1 (ppm)

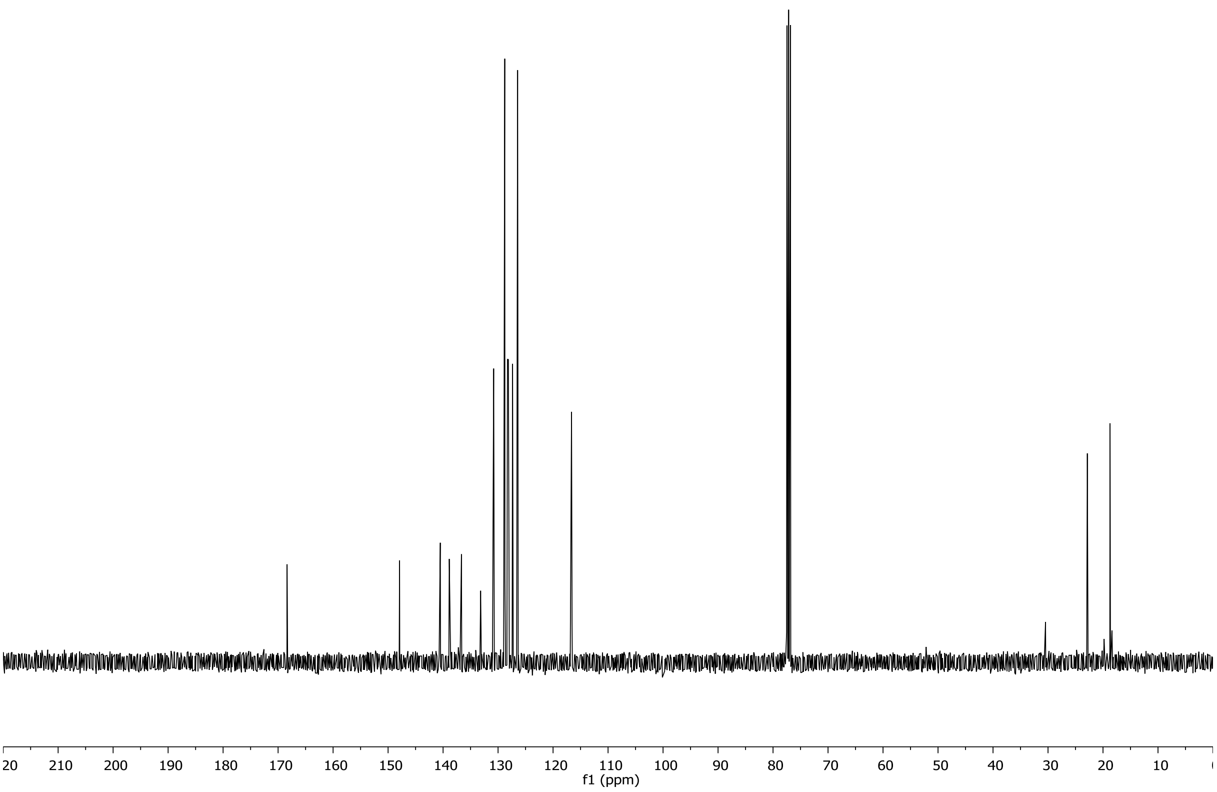
20 210 200 190 180 170 160 150 140 130 120 110 100 90 80 70 60 50 40 30 20 10
f1 (ppm)

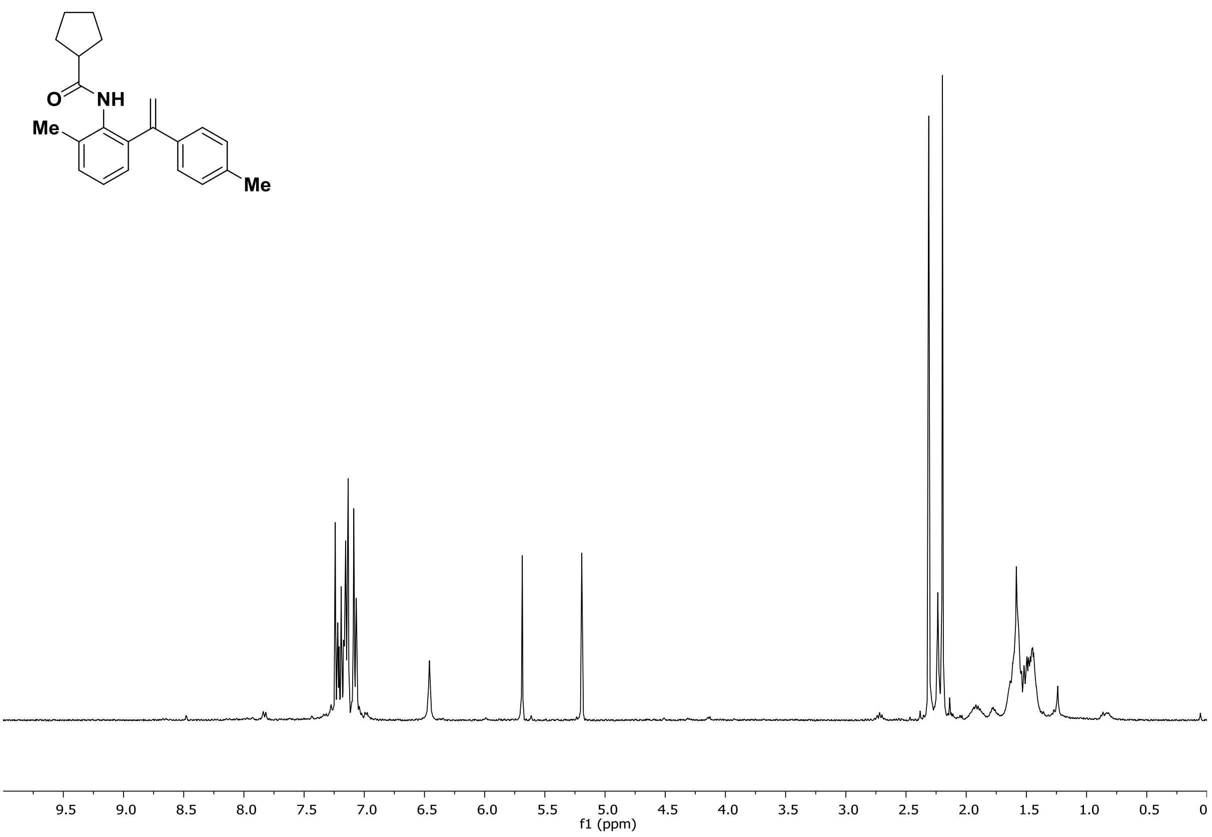
O
NH
Me
Me
9.5 9.0 8.5 8.0 7.5 7.0 6.5 6.0 5.5 5.0 4.5 4.0 3.5 3.0 2.5 2.0 1.5 1.0 0.5 0
f1 (ppm)

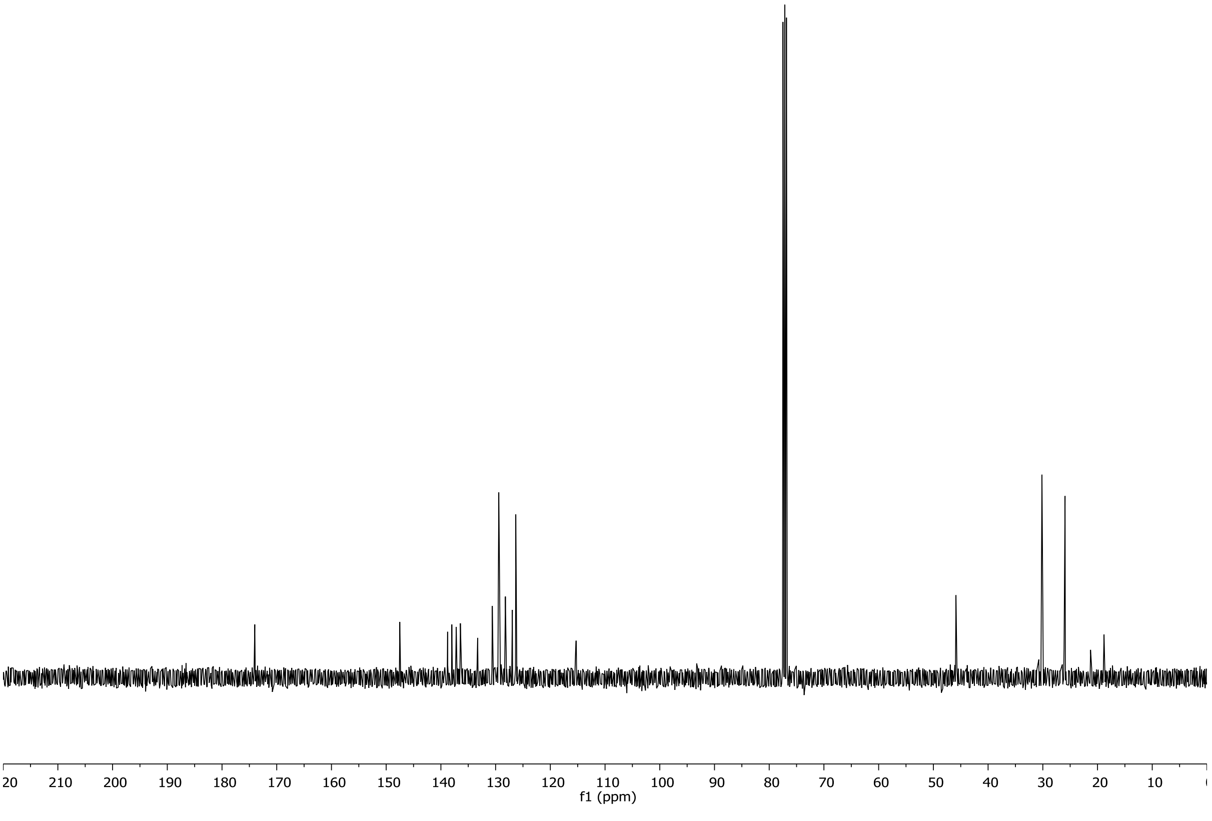
20 210 200 190 180 170 160 150 140 130 120 110 100 90 80 70 60 50 40 30 20 10
f1 (ppm)

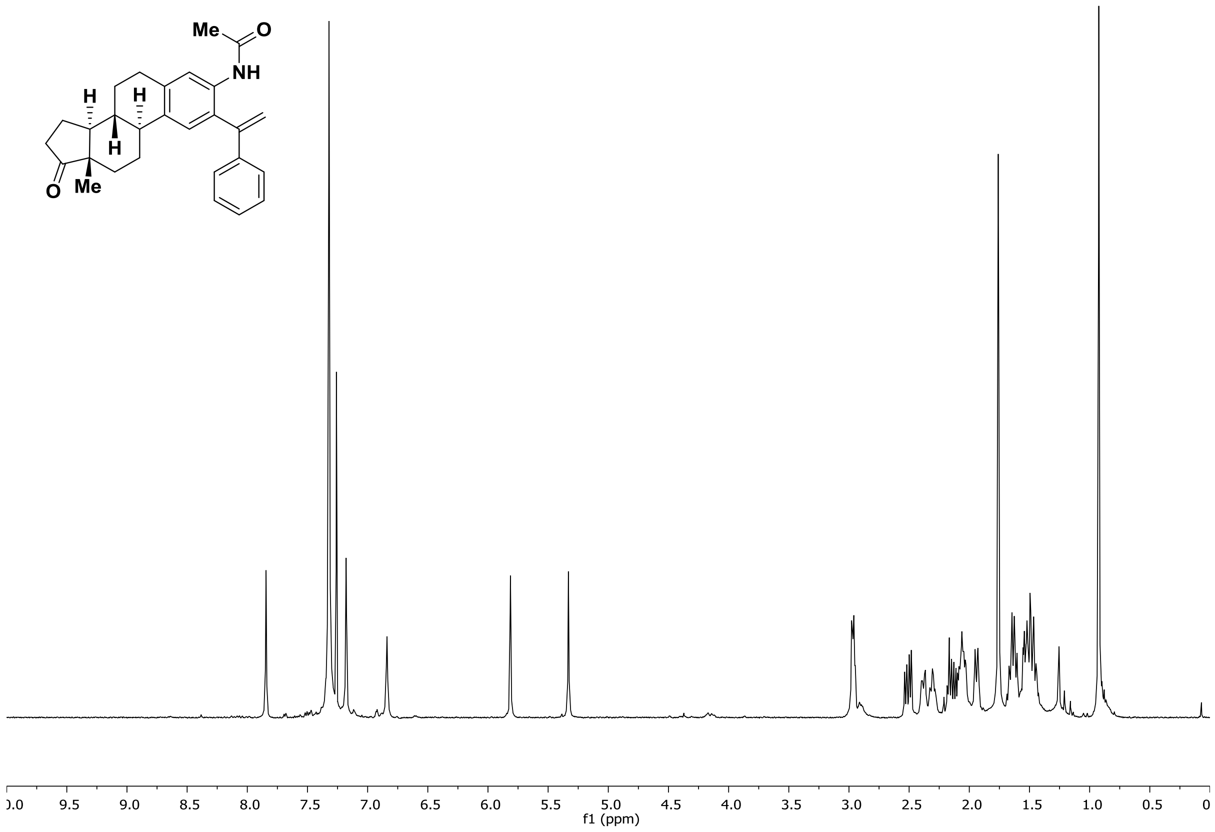
Me
O
NH
H
H
H
O
Me
f1 (ppm)

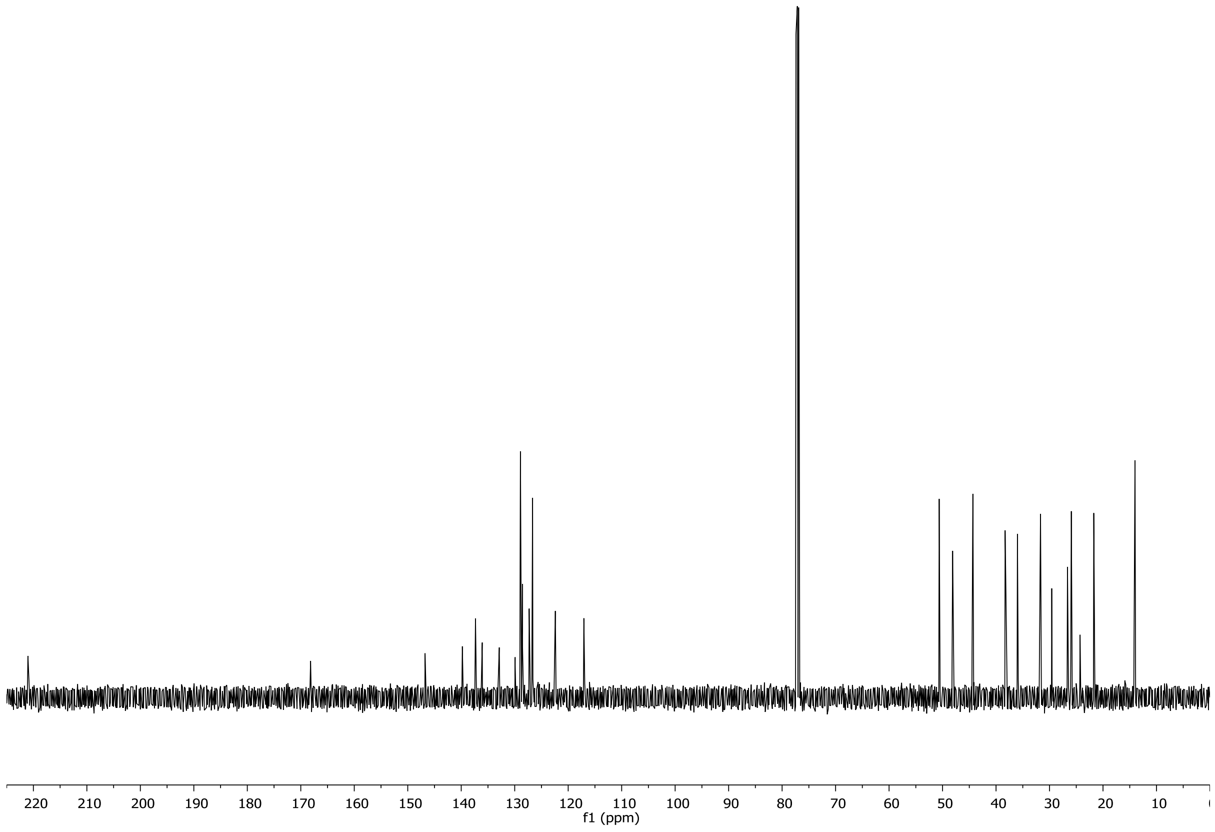
f1 (ppm)

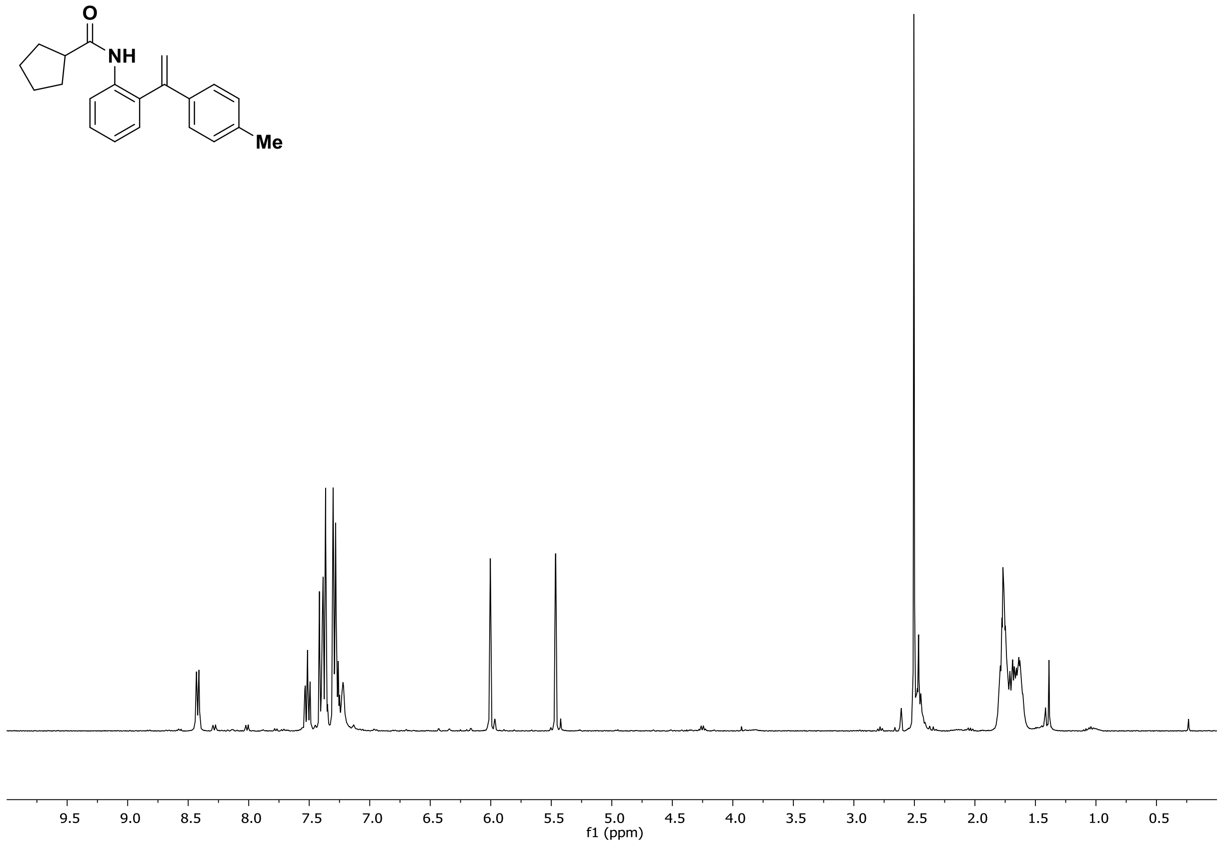
O
NH
Me
9.5 9.0 8.5 8.0 7.5 7.0 6.5 6.0 5.5 5.0 4.5 4.0 3.5 3.0 2.5 2.0 1.5 1.0 0.5
f1 (ppm)

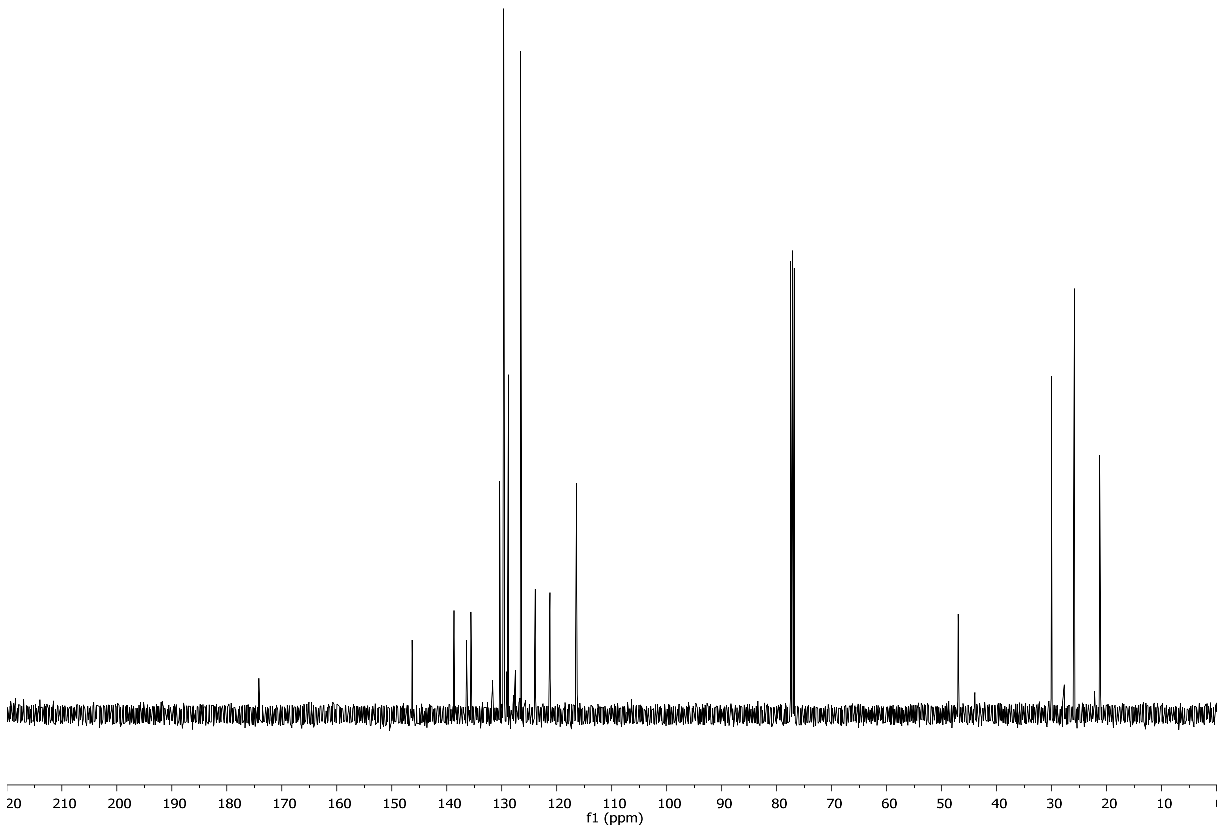
210 200 190 180 170 160 150 140 130 120 110 100 90 80 70 60 50 40 30 20 10
f1 (ppm)

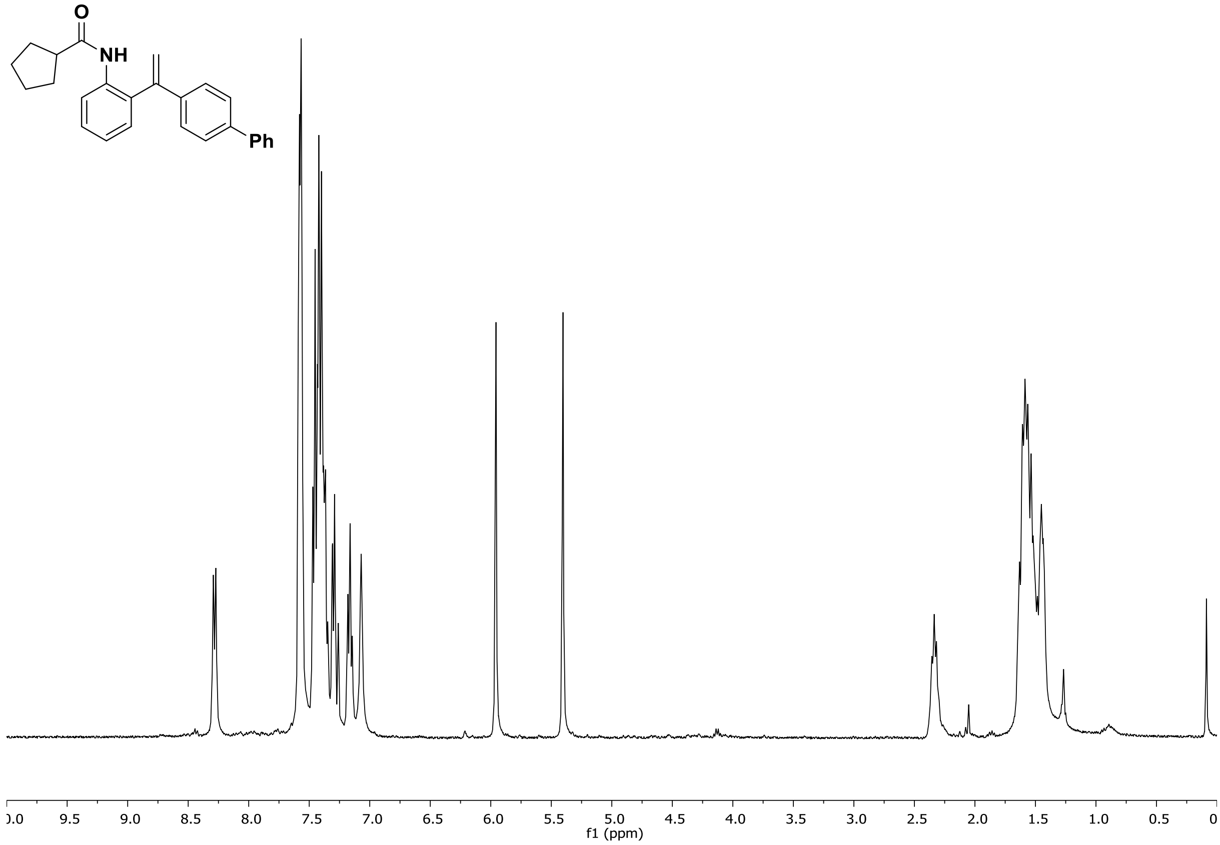
O
NH
Ph
f1 (ppm)

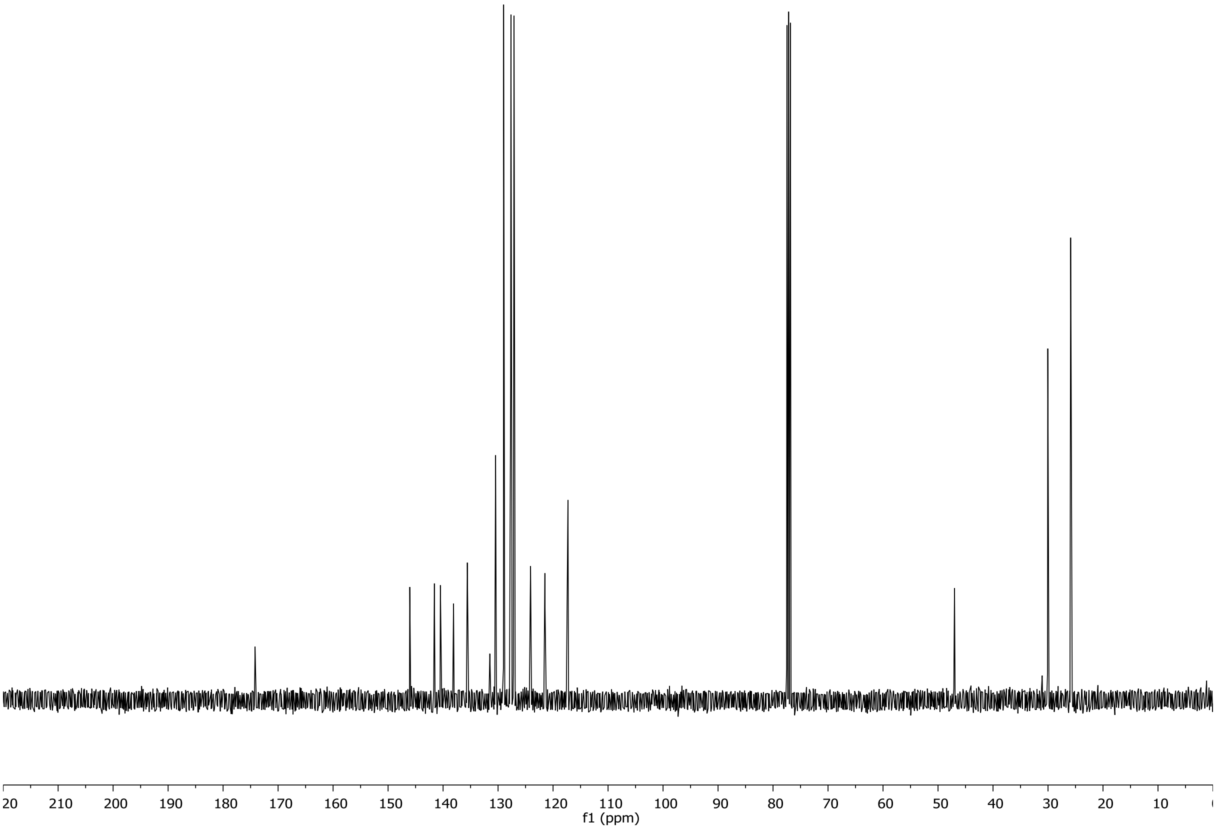
f1 (ppm)

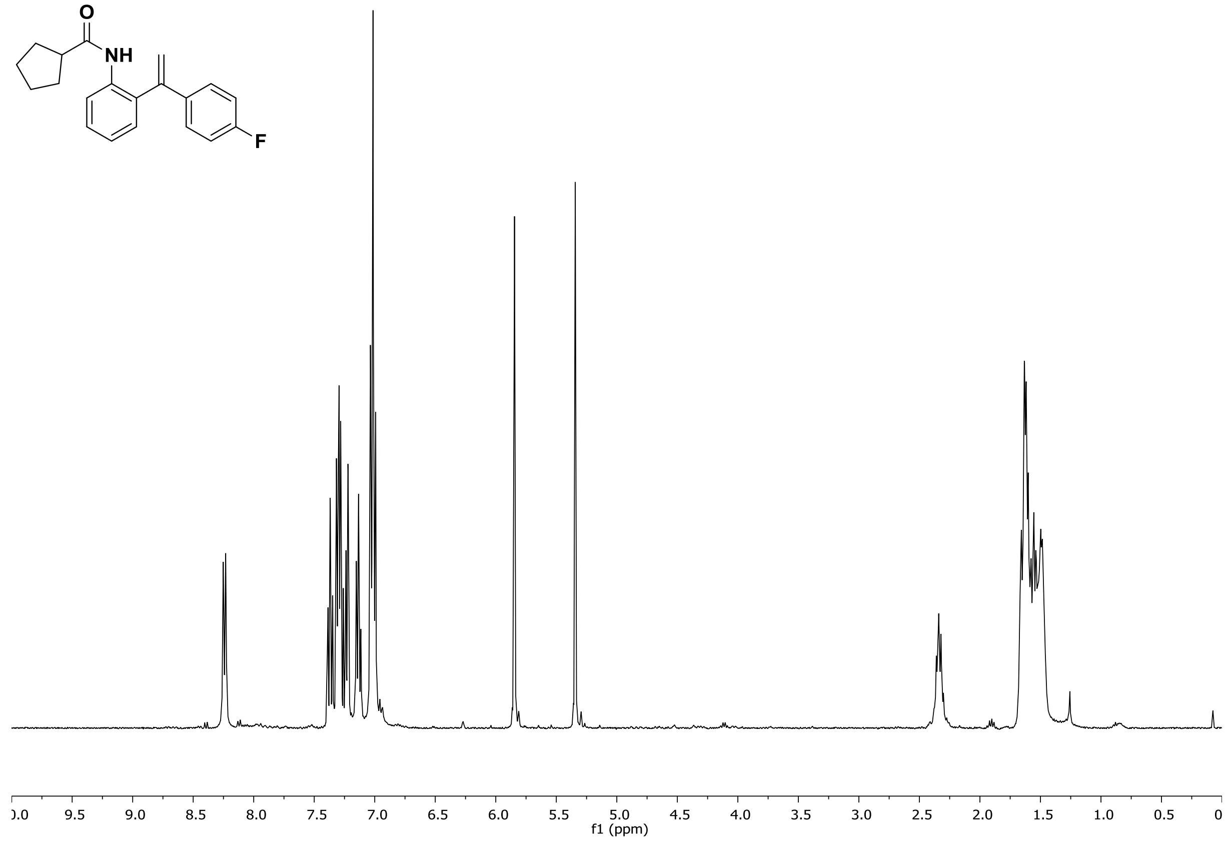
O
NH
F
f1 (ppm)

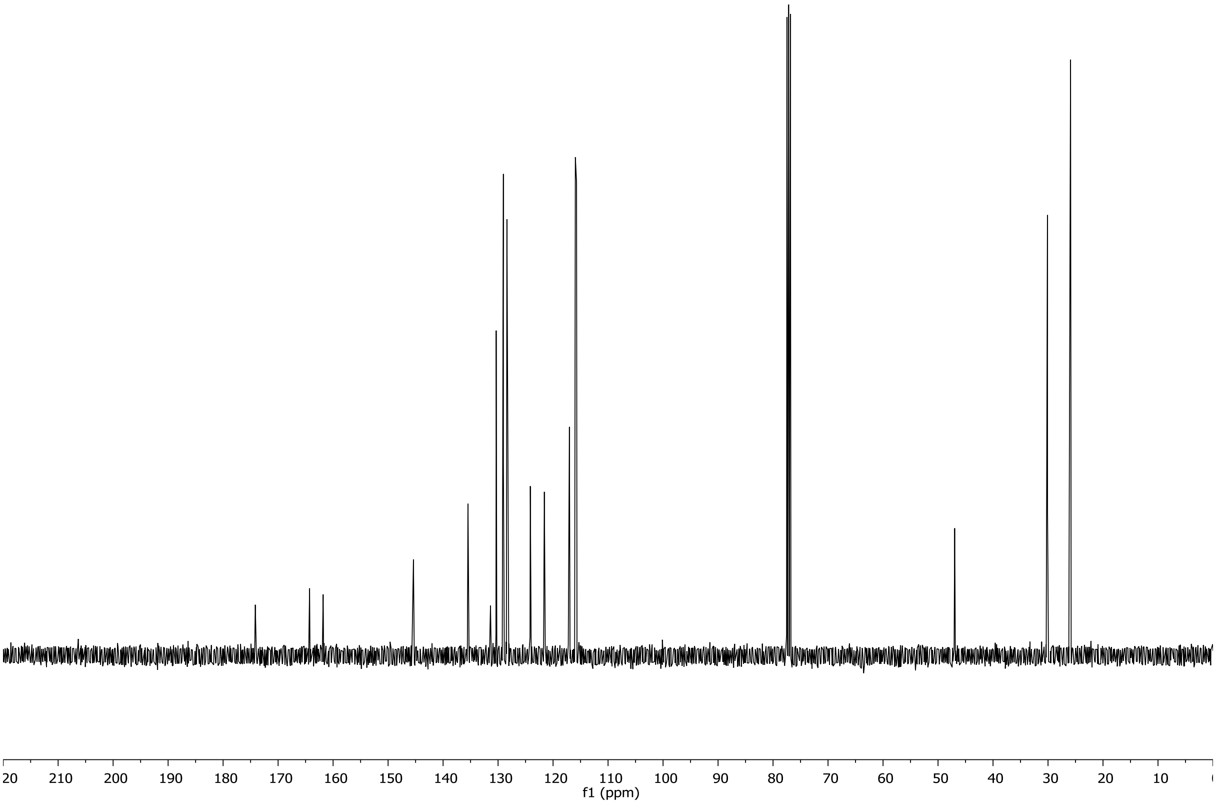
f1 (ppm)

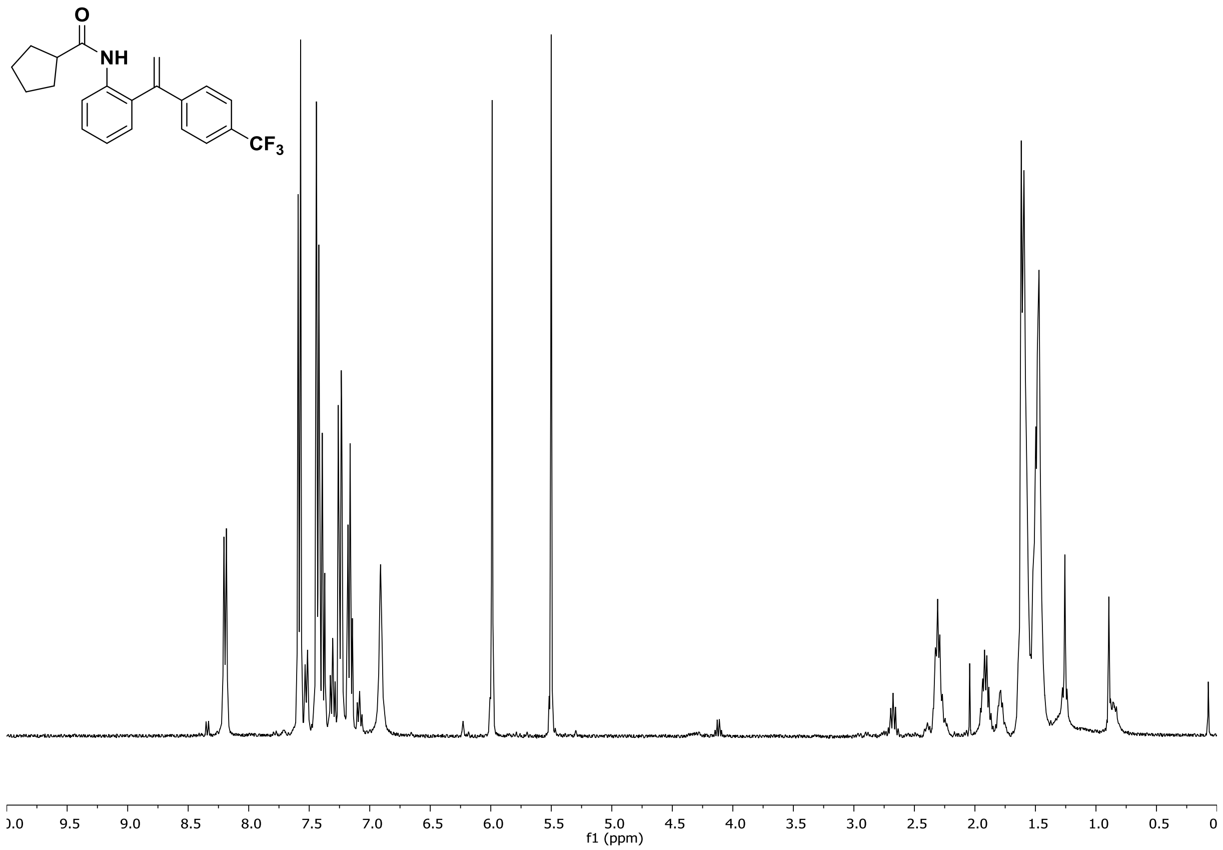
O
NH
CF3
0.0 9.5 9.0 8.5 8.0 7.5 7.0 6.5 6.0 5.5 5.0 4.5 4.0 3.5 3.0 2.5 2.0 1.5 1.0 0.5 0
f1 (ppm)

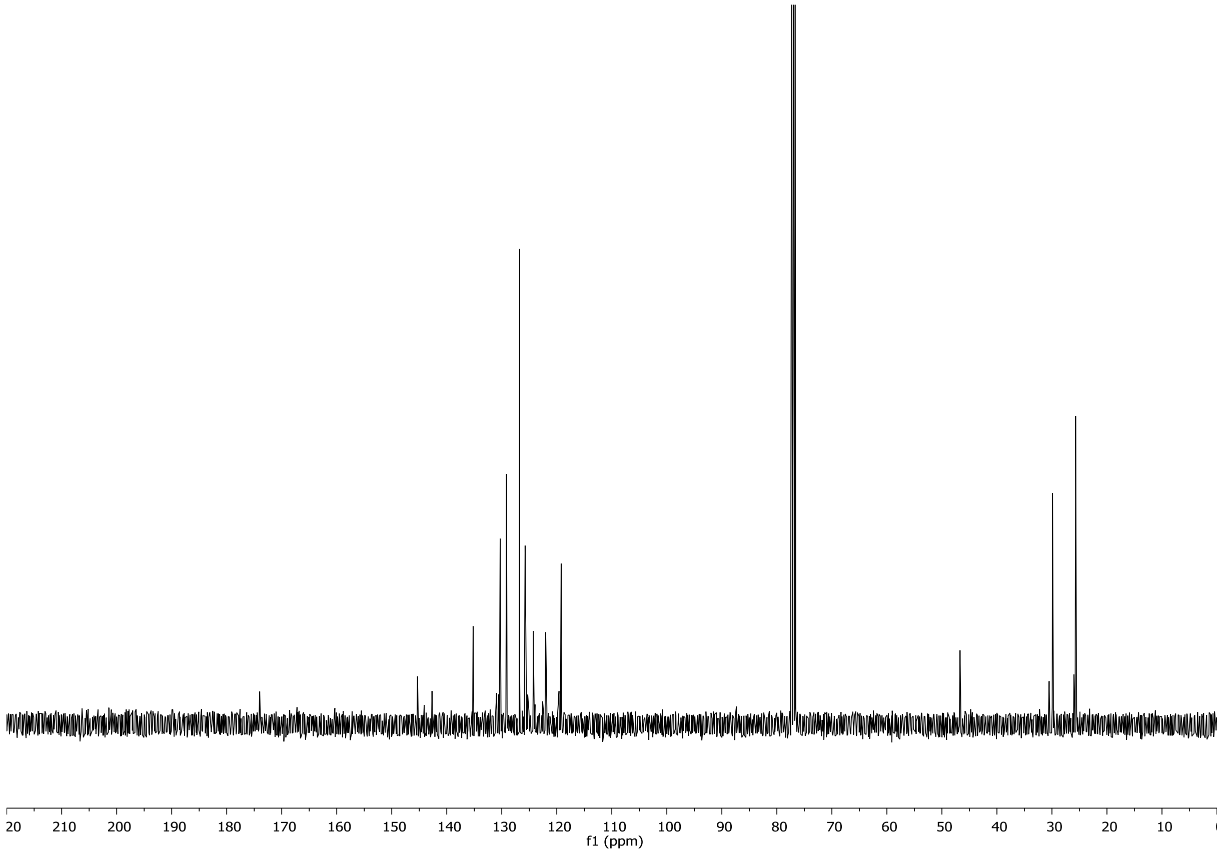
20 210 200 190 180 170 160 150 140 130 120 110 100 90 80 70 60 50 40 30 20 10
f1 (ppm)

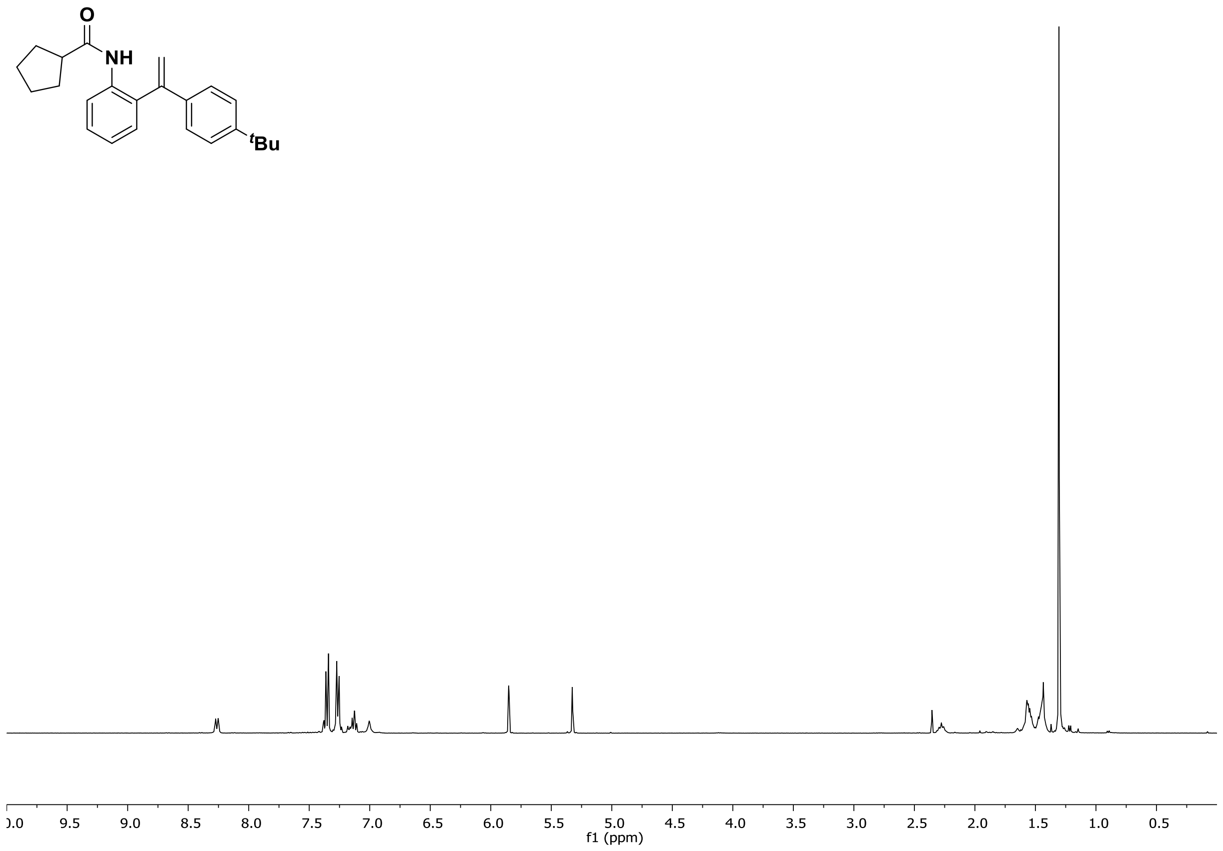
O
NH
tBu
f1 (ppm)

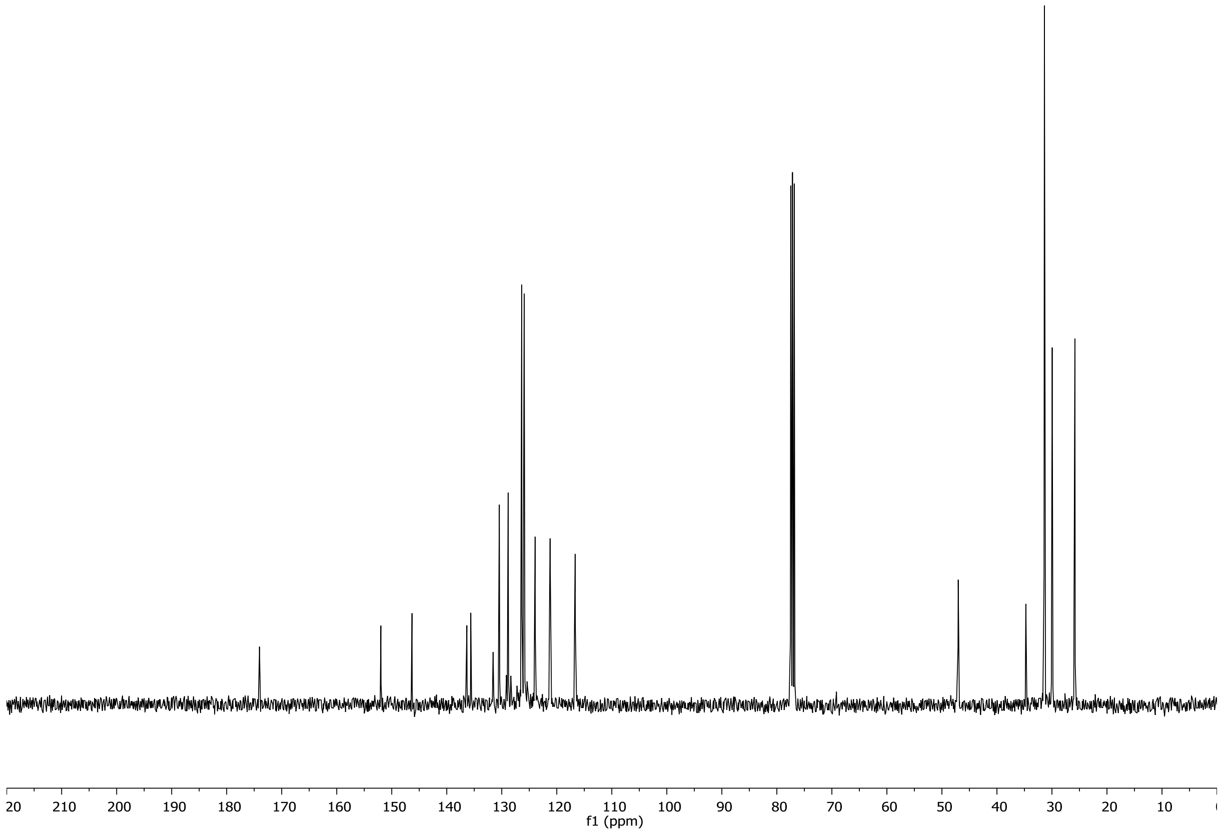
f1 (ppm)

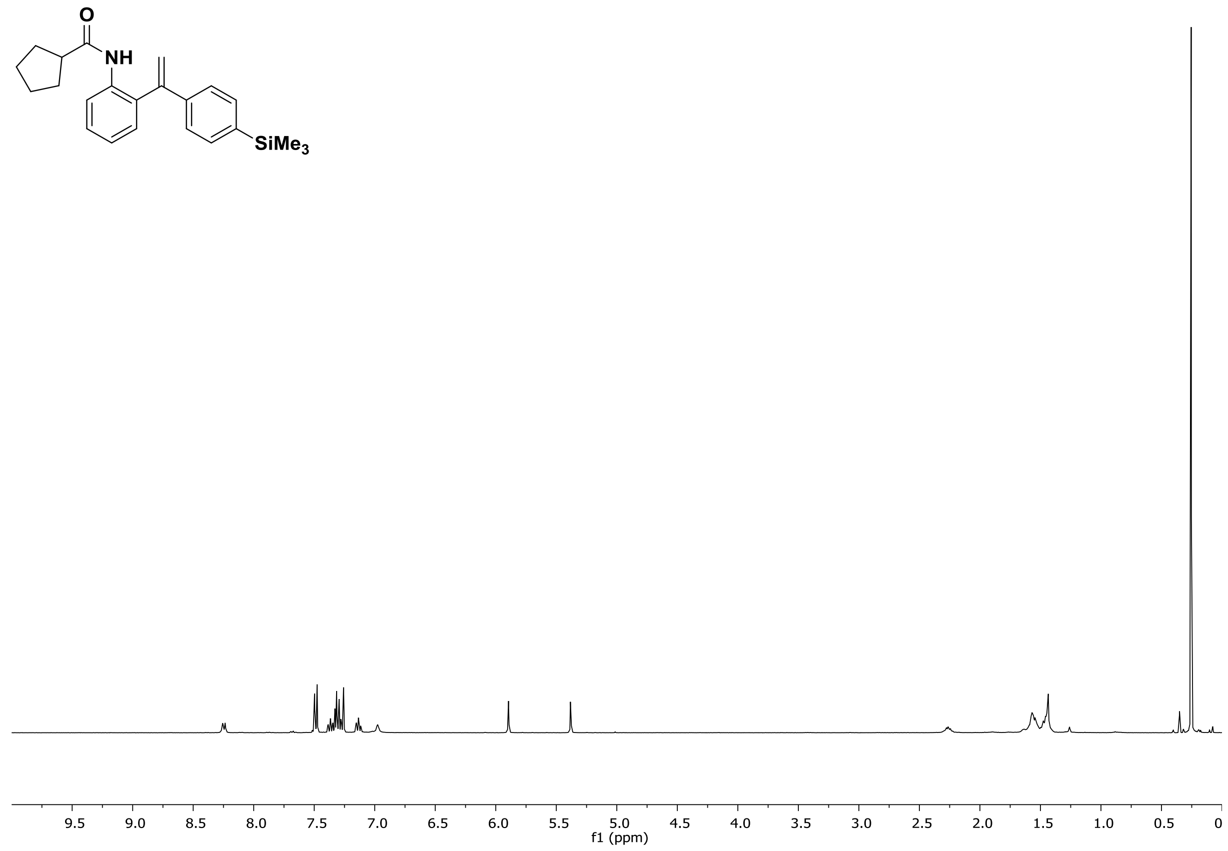
O
NH
SiMe3
9.5 9.0 8.5 8.0 7.5 7.0 6.5 6.0 5.5 5.0 4.5 4.0 3.5 3.0 2.5 2.0 1.5 1.0 0.5 0
f1 (ppm)

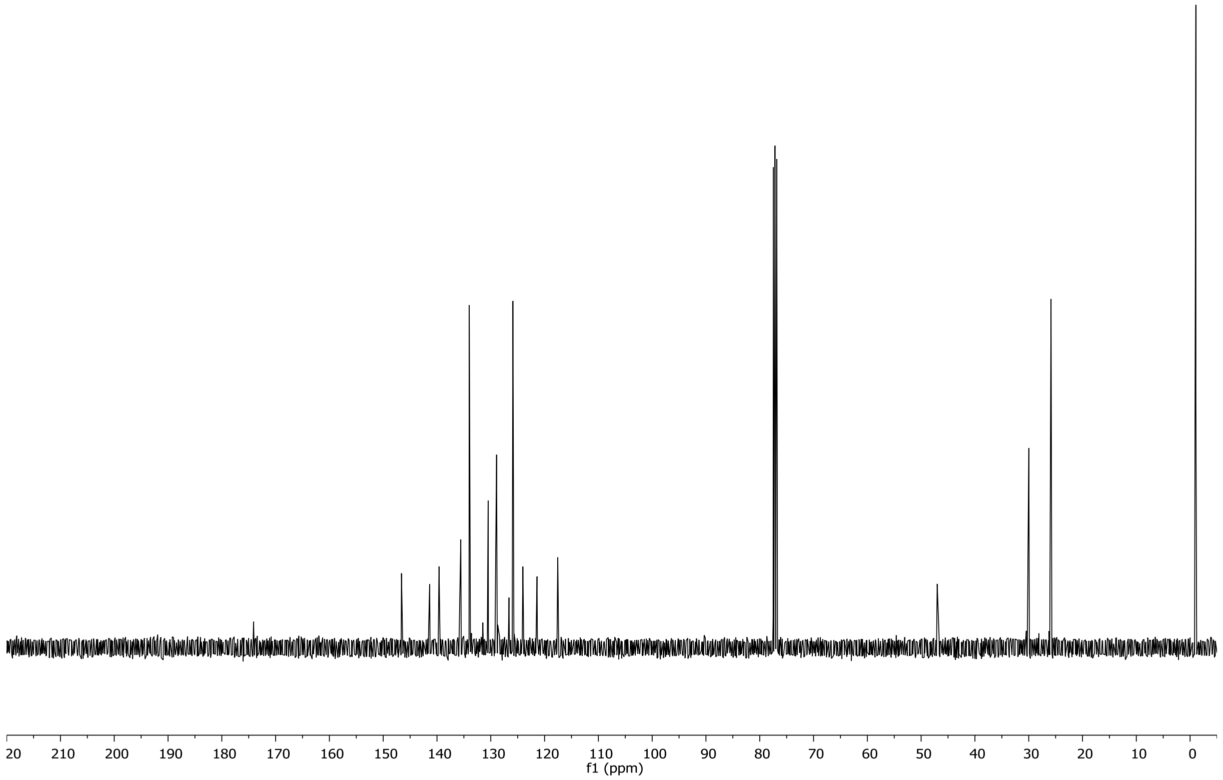
20 210 200 190 180 170 160 150 140 130 120 110 100 90 80 70 60 50 40 30 20 10 0
f1 (ppm)

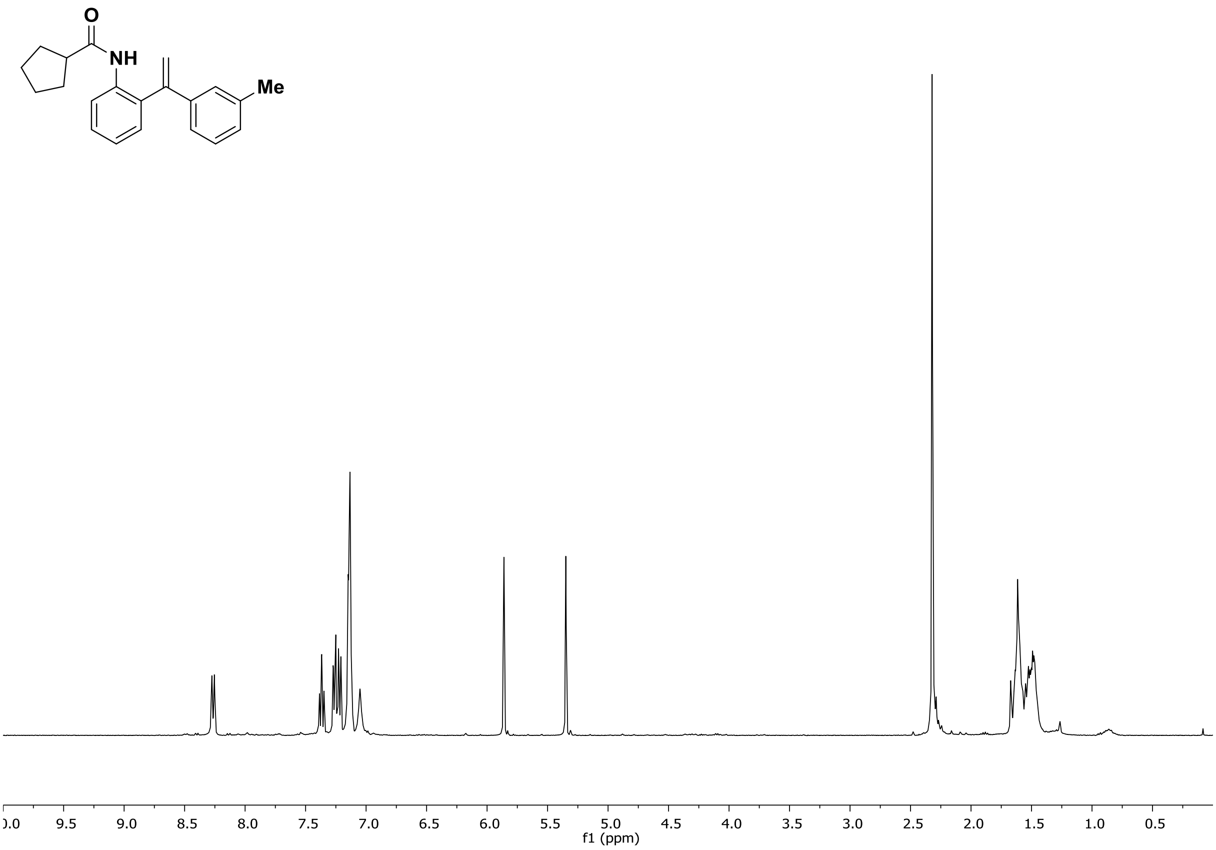
O
NH
Me
0.0 9.5 9.0 8.5 8.0 7.5 7.0 6.5 6.0 5.5 5.0 4.5 4.0 3.5 3.0 2.5 2.0 1.5 1.0 0.5
f1 (ppm)

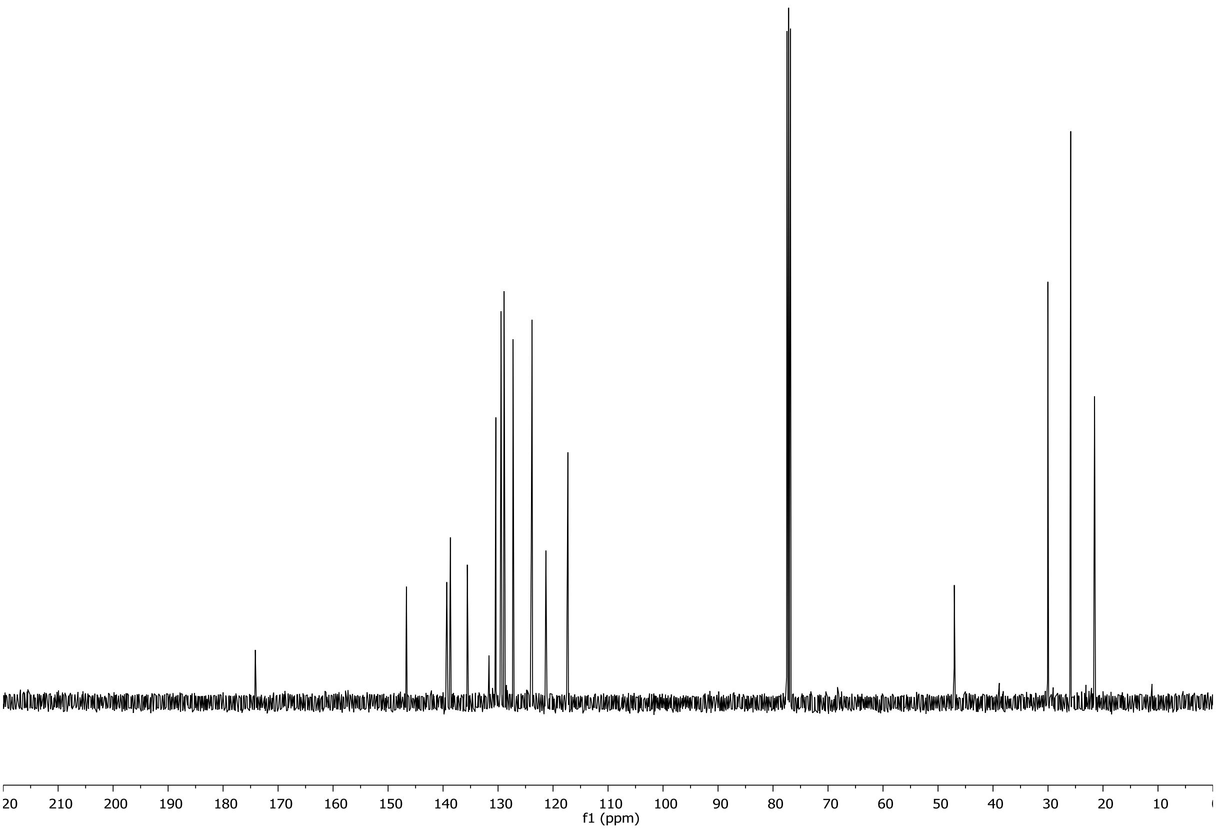
20 210 200 190 180 170 160 150 140 130 120 110 100 90 80 70 60 50 40 30 20 10
f1 (ppm)

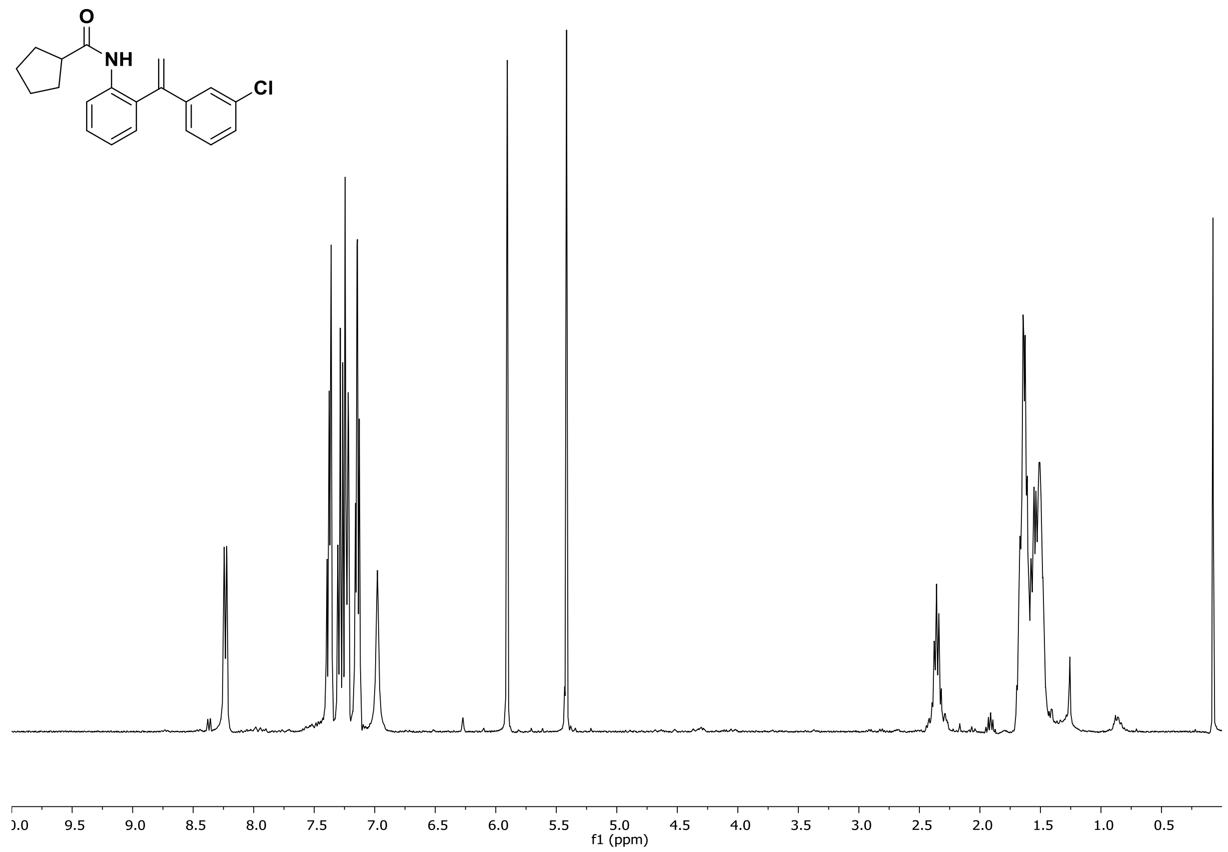
O
NH
Cl
f1 (ppm)

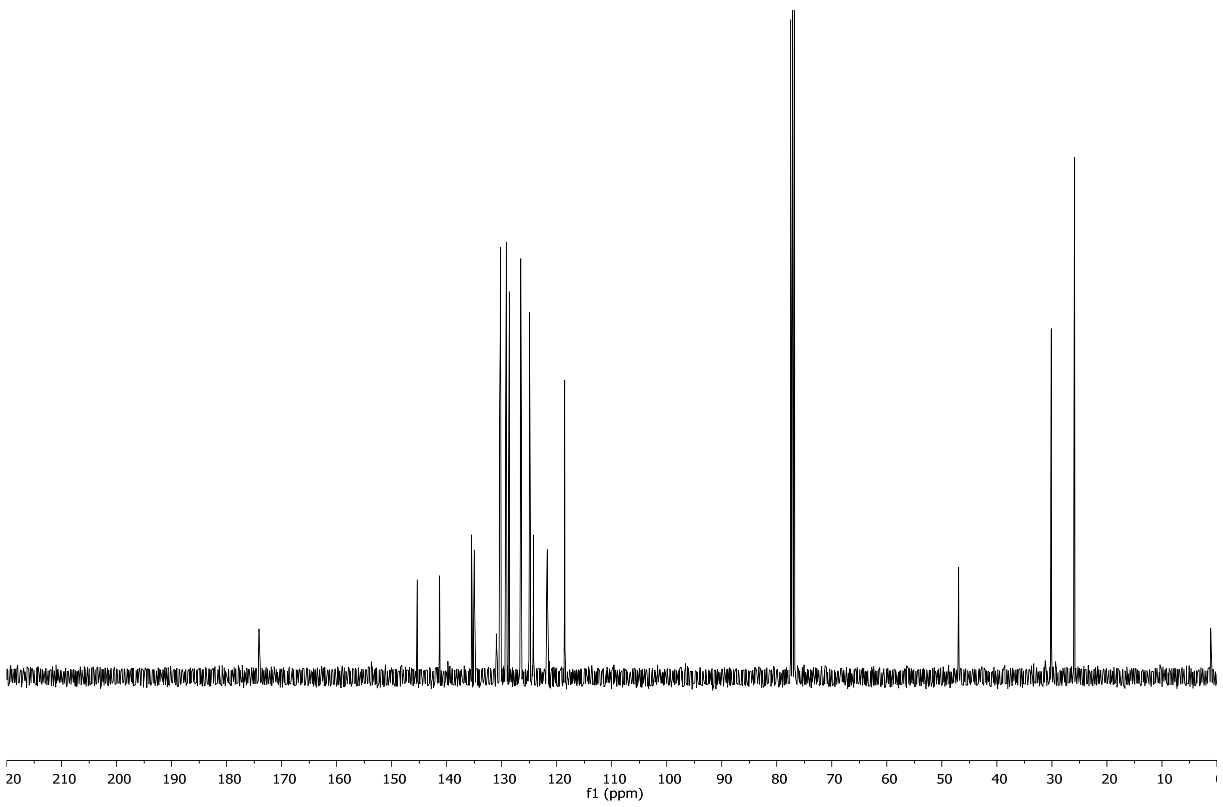

f1 (ppm)

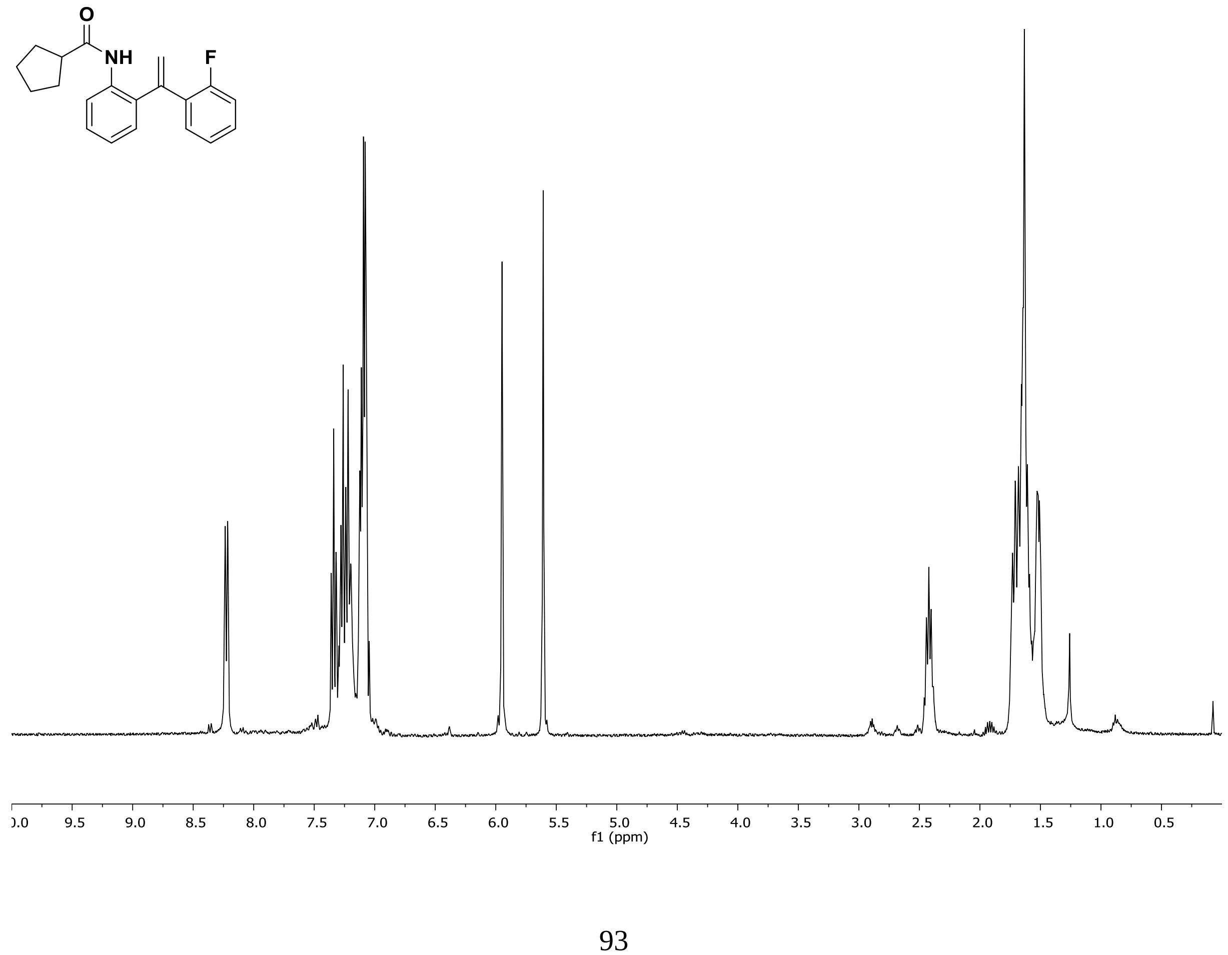

O
NH
F
f1 (ppm)

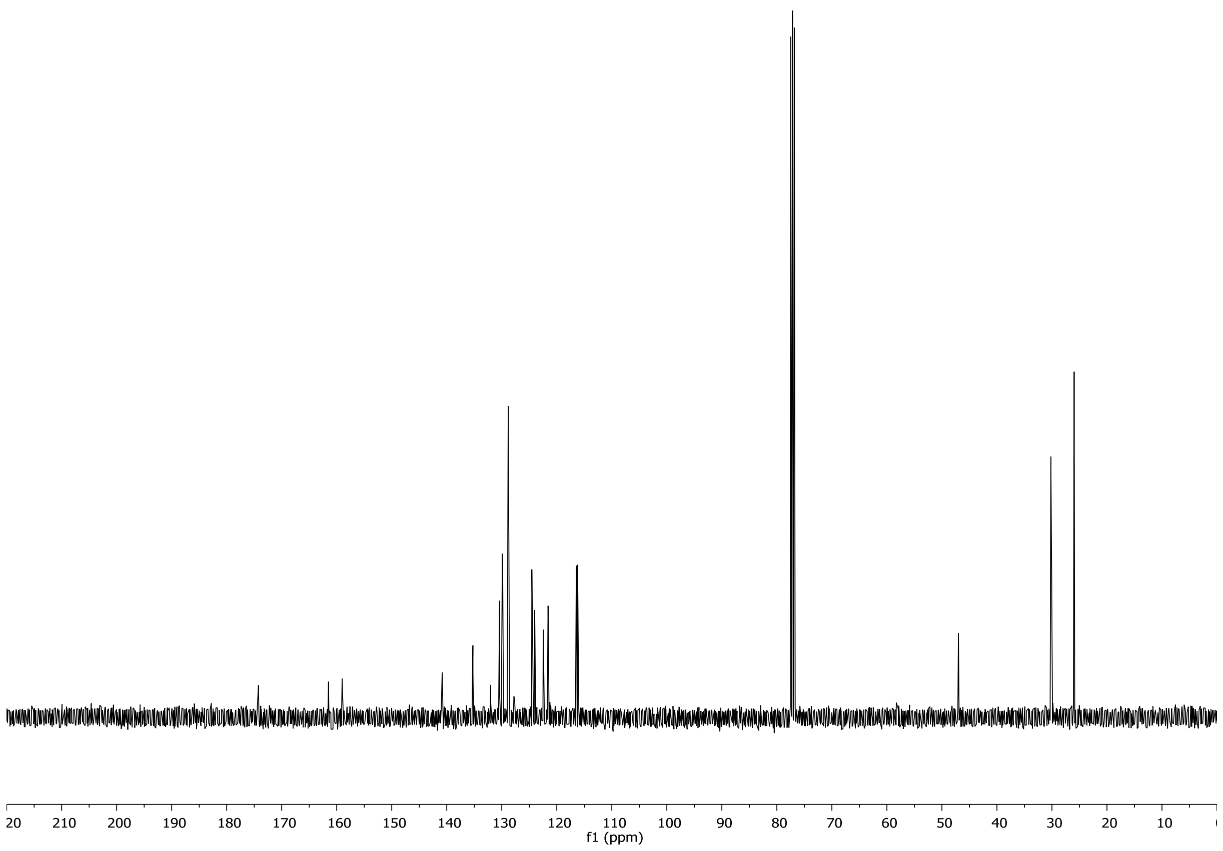
f1 (ppm)

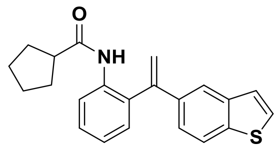
O
NH
S

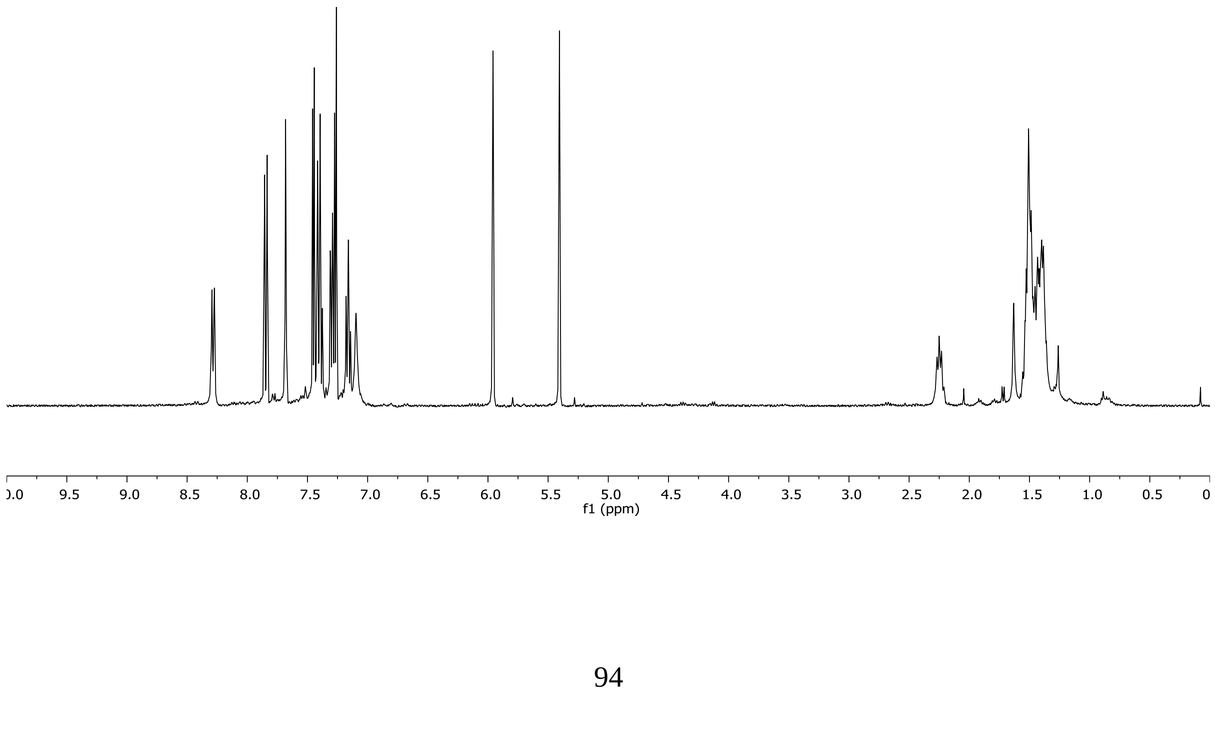
f1 (ppm)

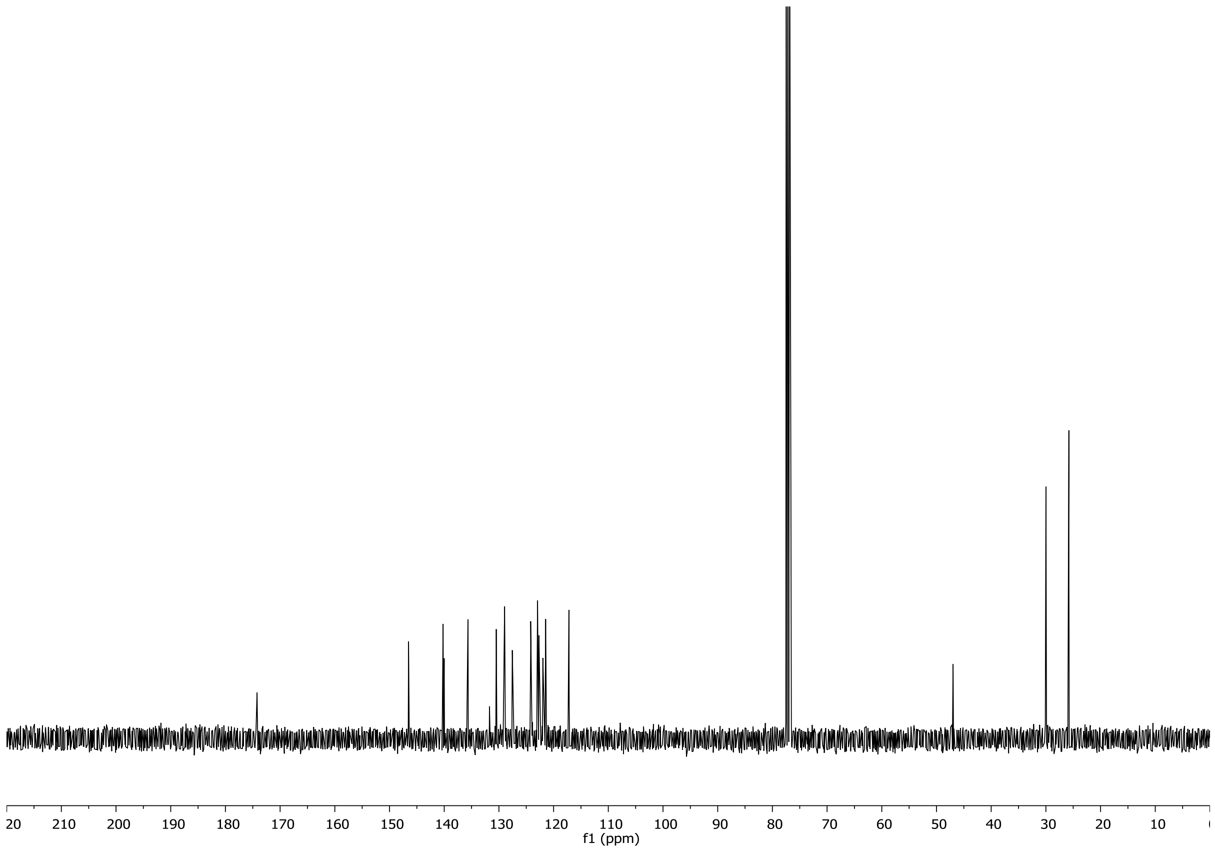

20 210 200 190 180 170 160 150 140 130 120 110 100 90 80 70 60 50 40 30 20 10
f1 (ppm)

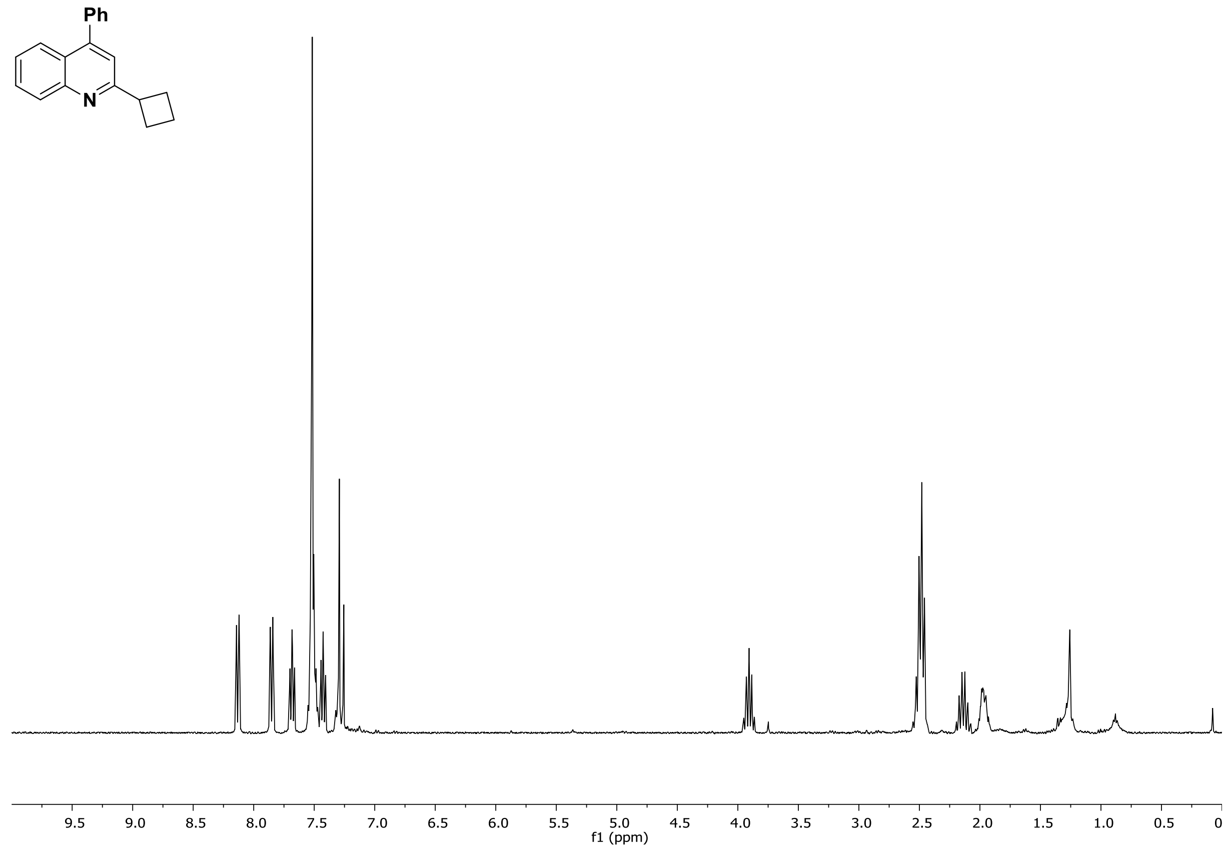
Ph
N
9.5 9.0 8.5 8.0 7.5 7.0 6.5 6.0 5.5 5.0 4.5 4.0 3.5 3.0 2.5 2.0 1.5 1.0 0.5 0
f1 (ppm)

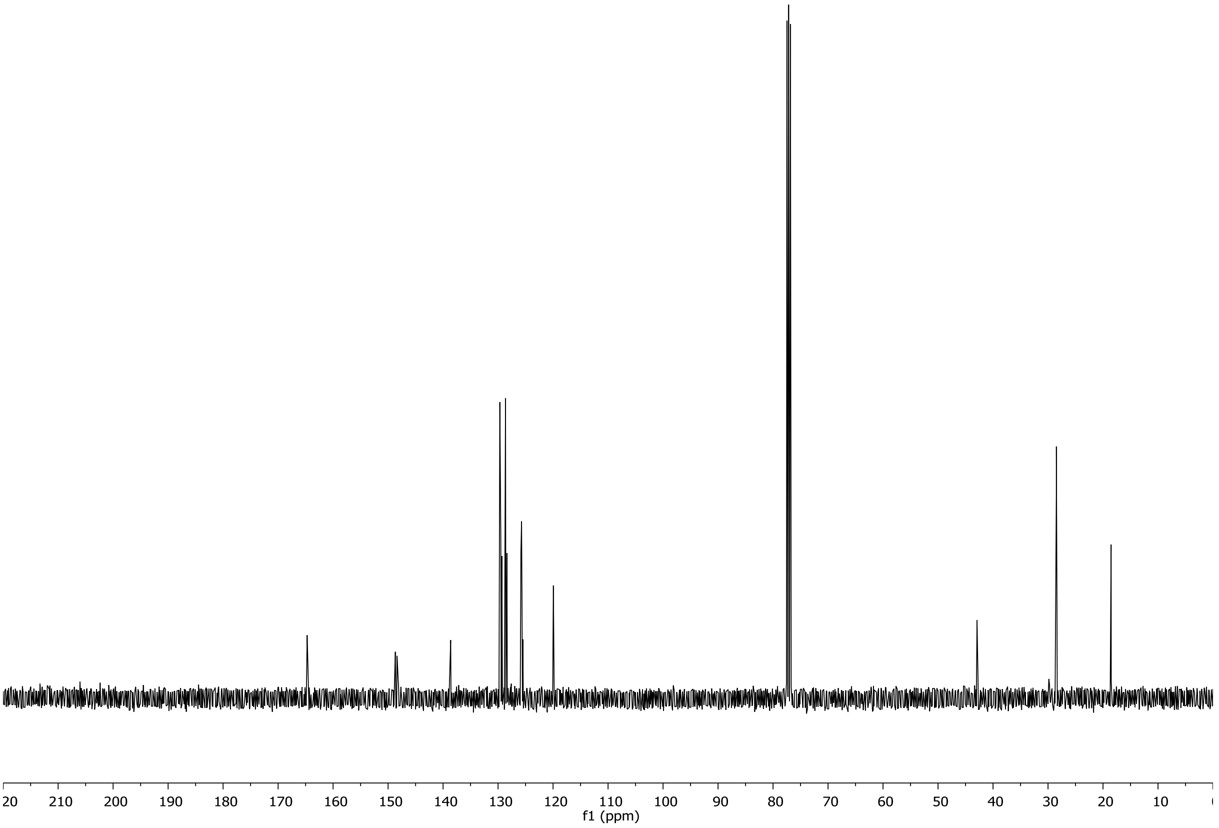
20 210 200 190 180 170 160 150 140 130 120 110 100 90 80 70 60 50 40 30 20 10
f1 (ppm)

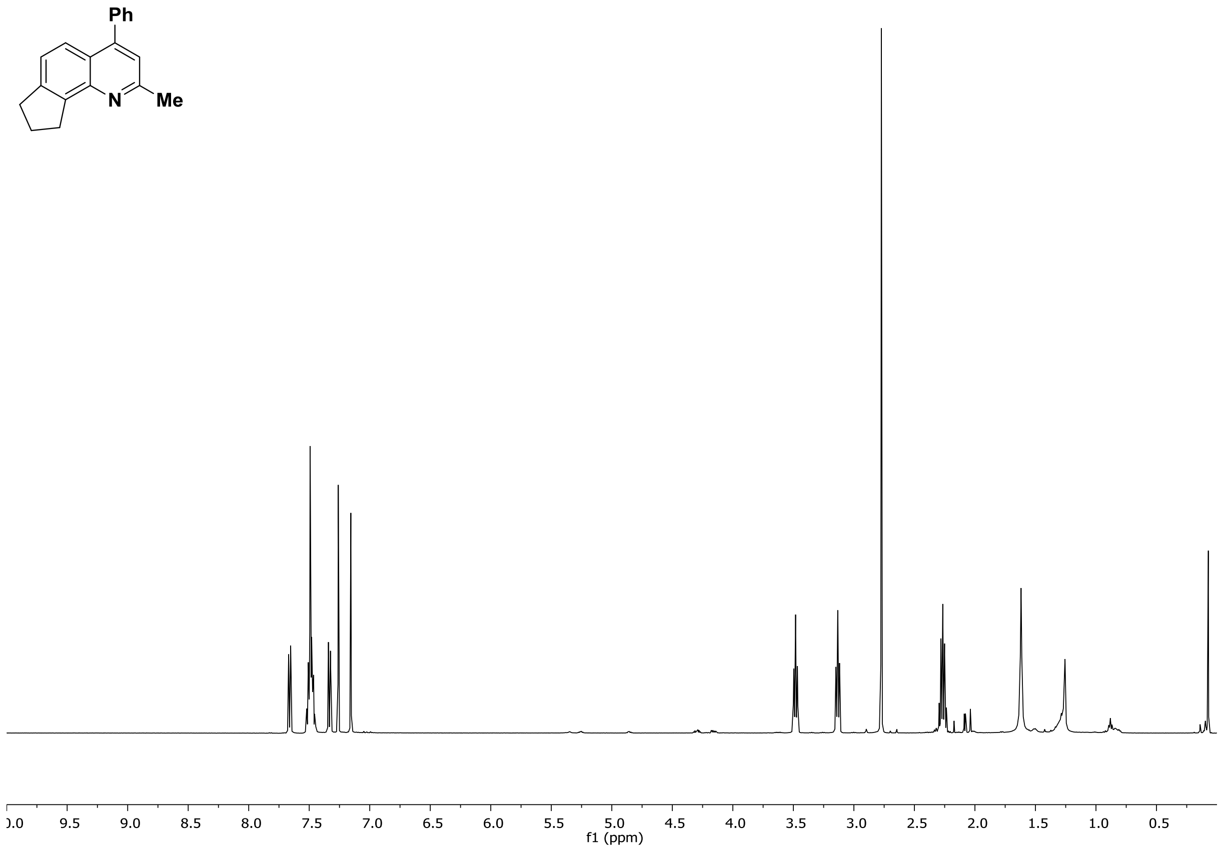
Ph
N
Me
f1 (ppm)

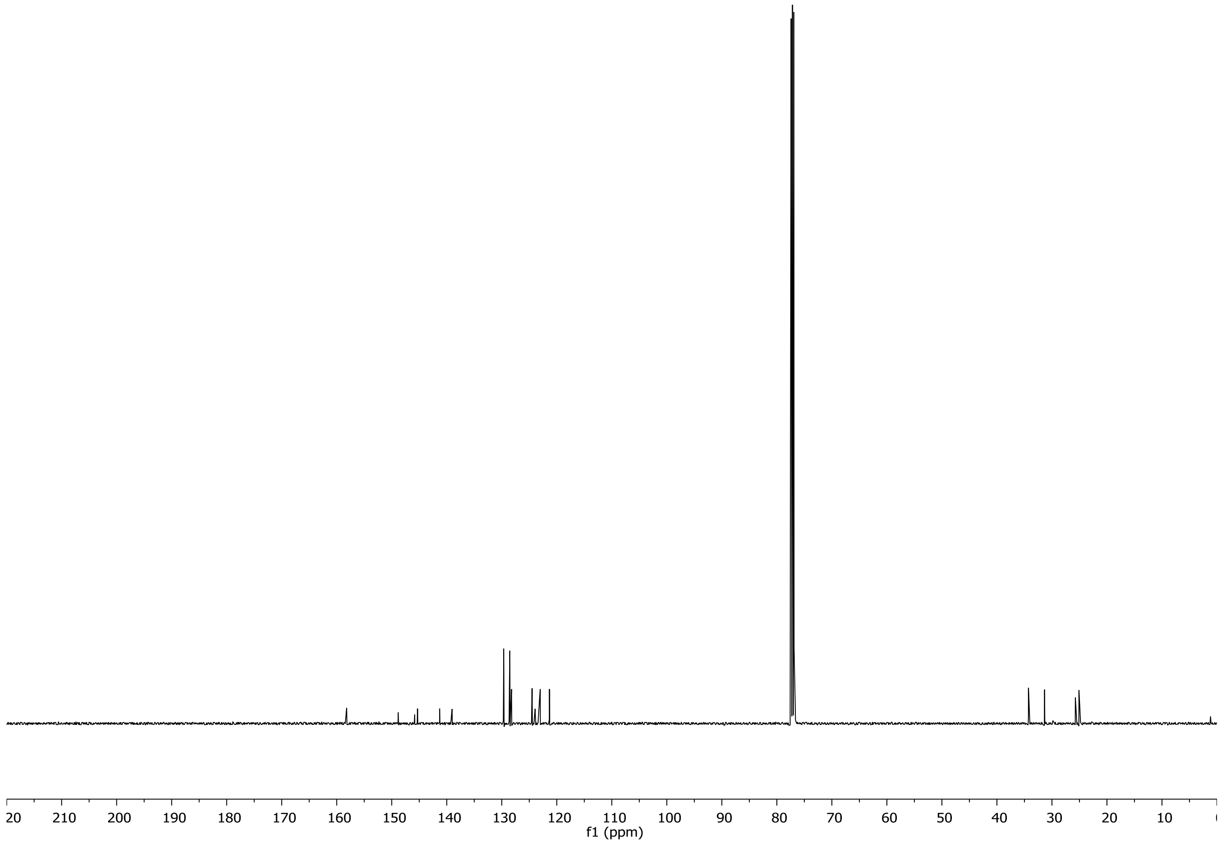
f1 (ppm)

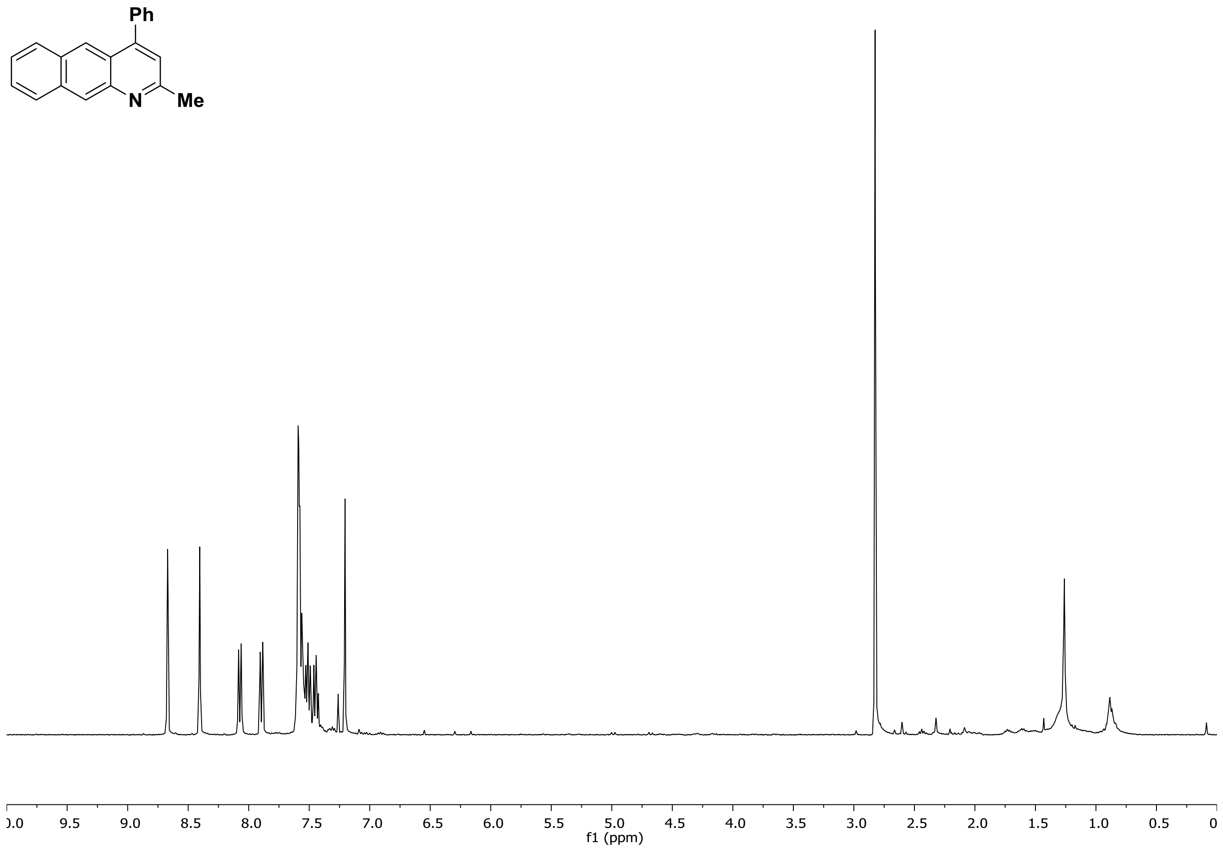
Ph
N
Me
0.0 9.5 9.0 8.5 8.0 7.5 7.0 6.5 6.0 5.5 5.0 4.5 4.0 3.5 3.0 2.5 2.0 1.5 1.0 0.5 0
f1 (ppm)

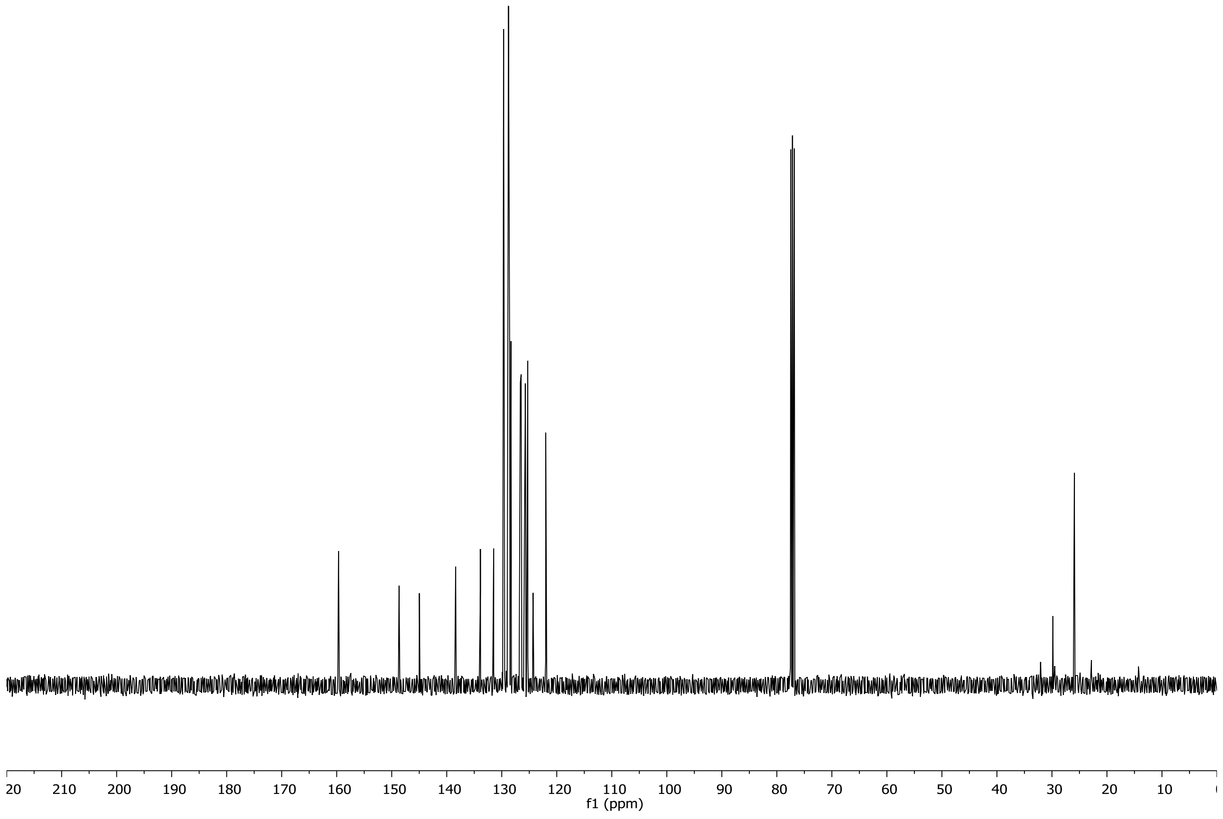
20 210 200 190 180 170 160 150 140 130 120 110 100 90 80 70 60 50 40 30 20 10
f1 (ppm)

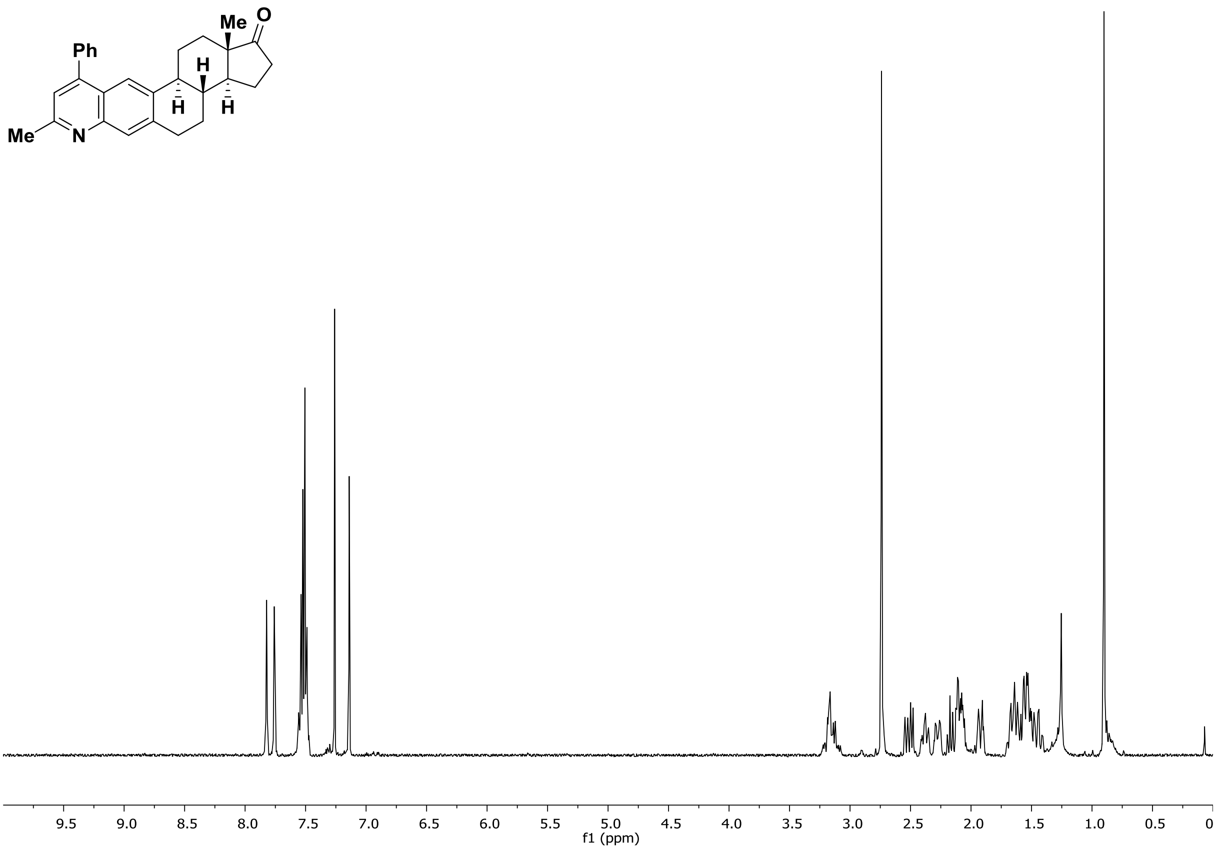
Ph
Me
O
H
Me
N
f1 (ppm)

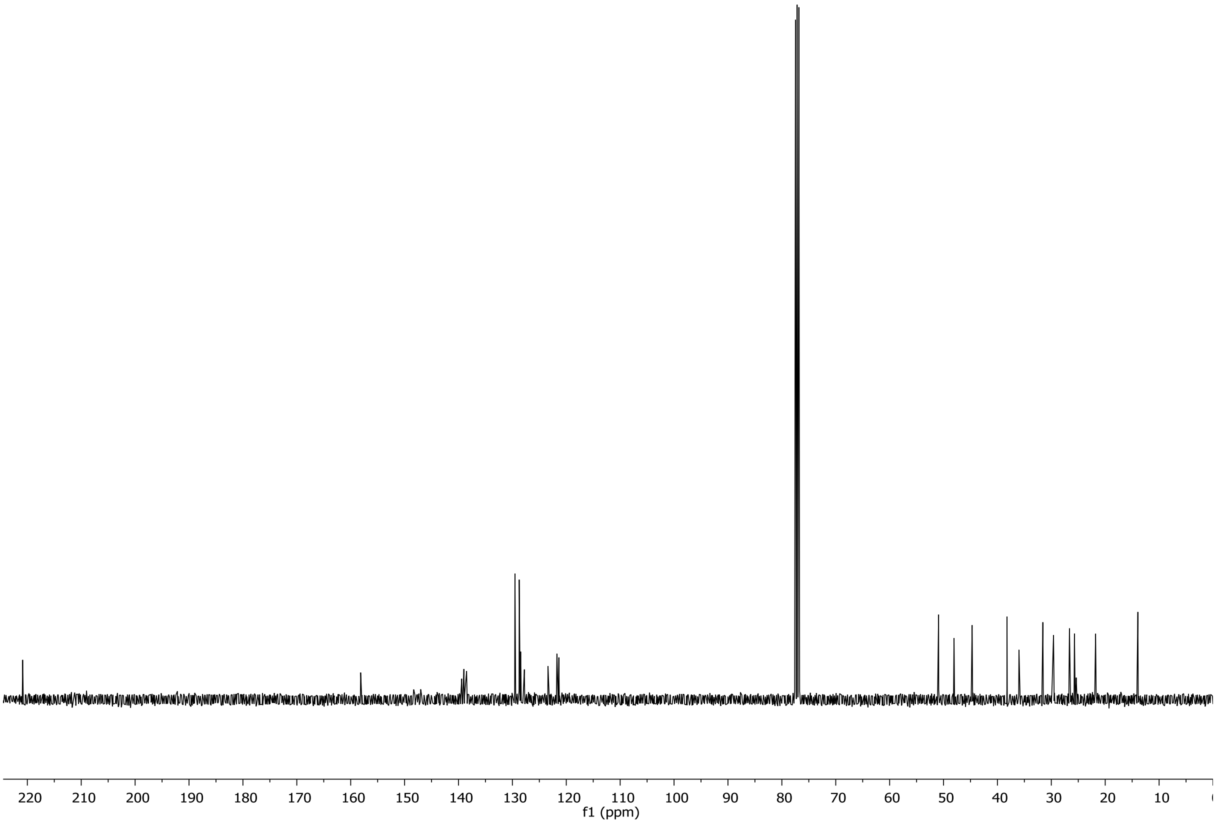
f1 (ppm)

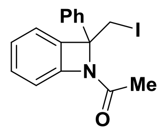
Ph
I
N
Me
O

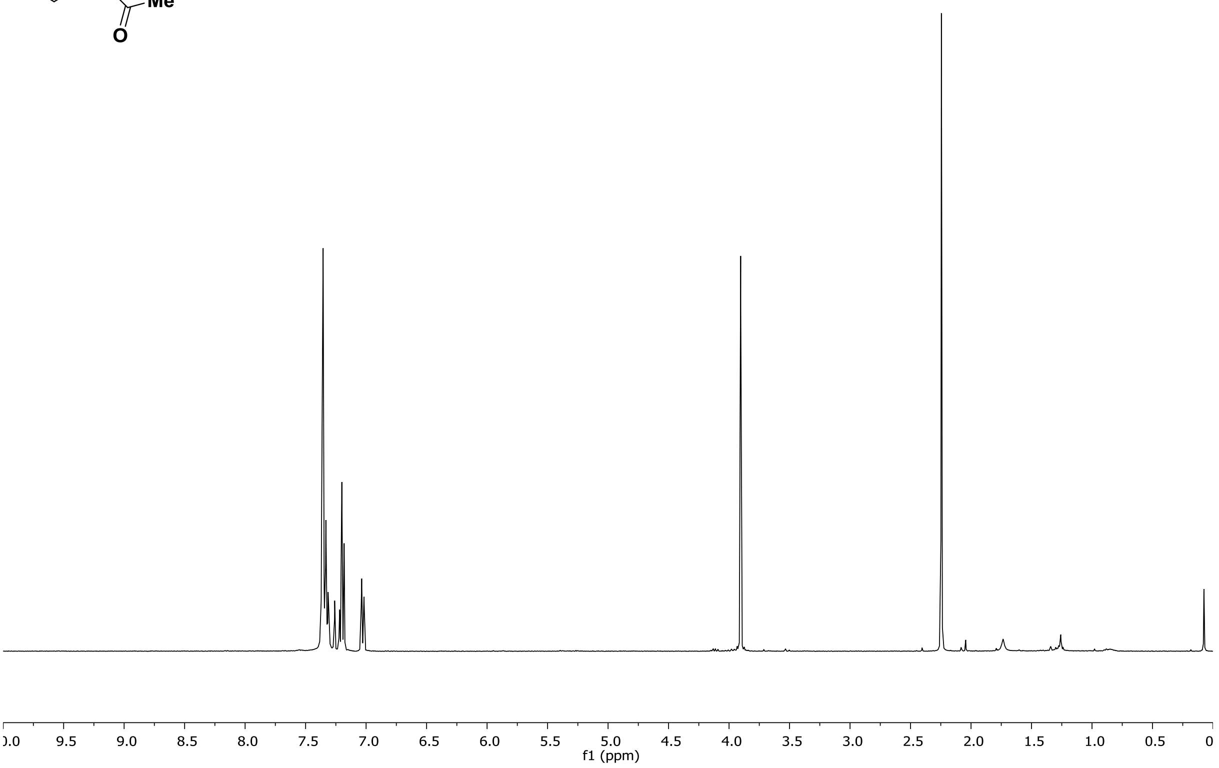
f1 (ppm)

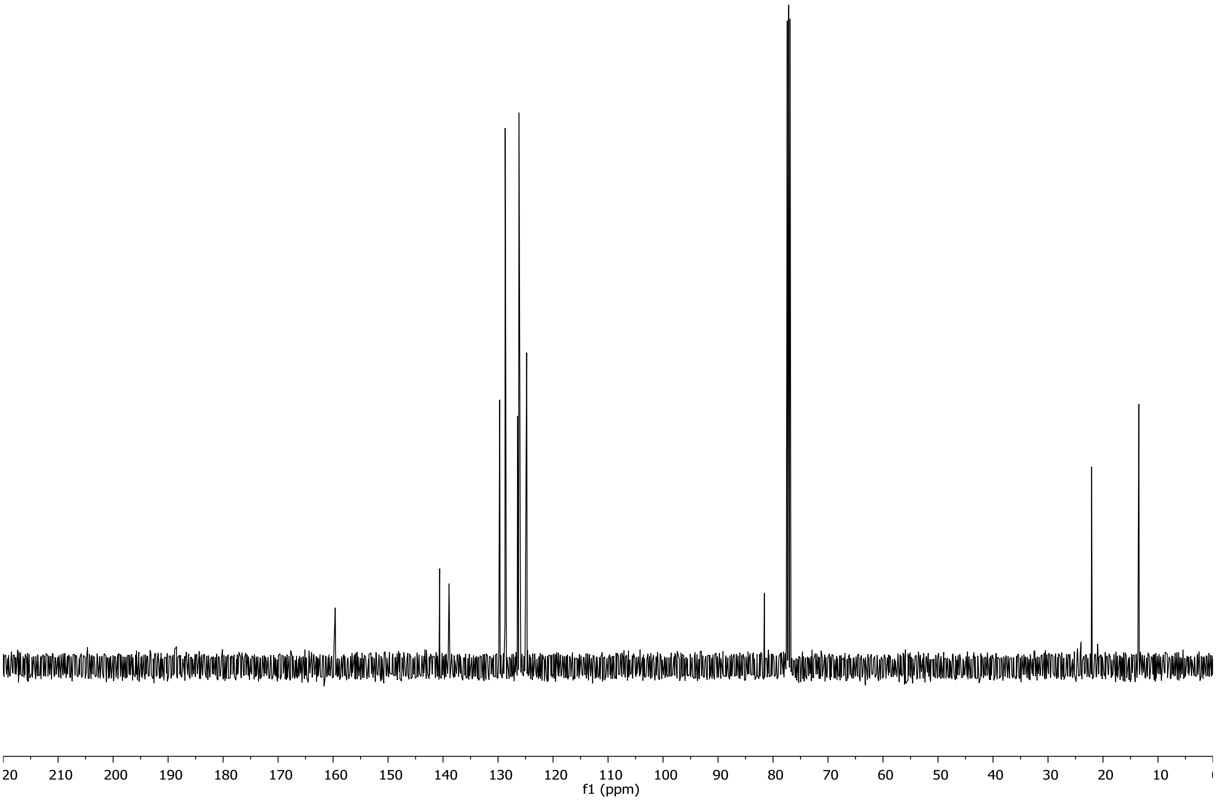
f1 (ppm)

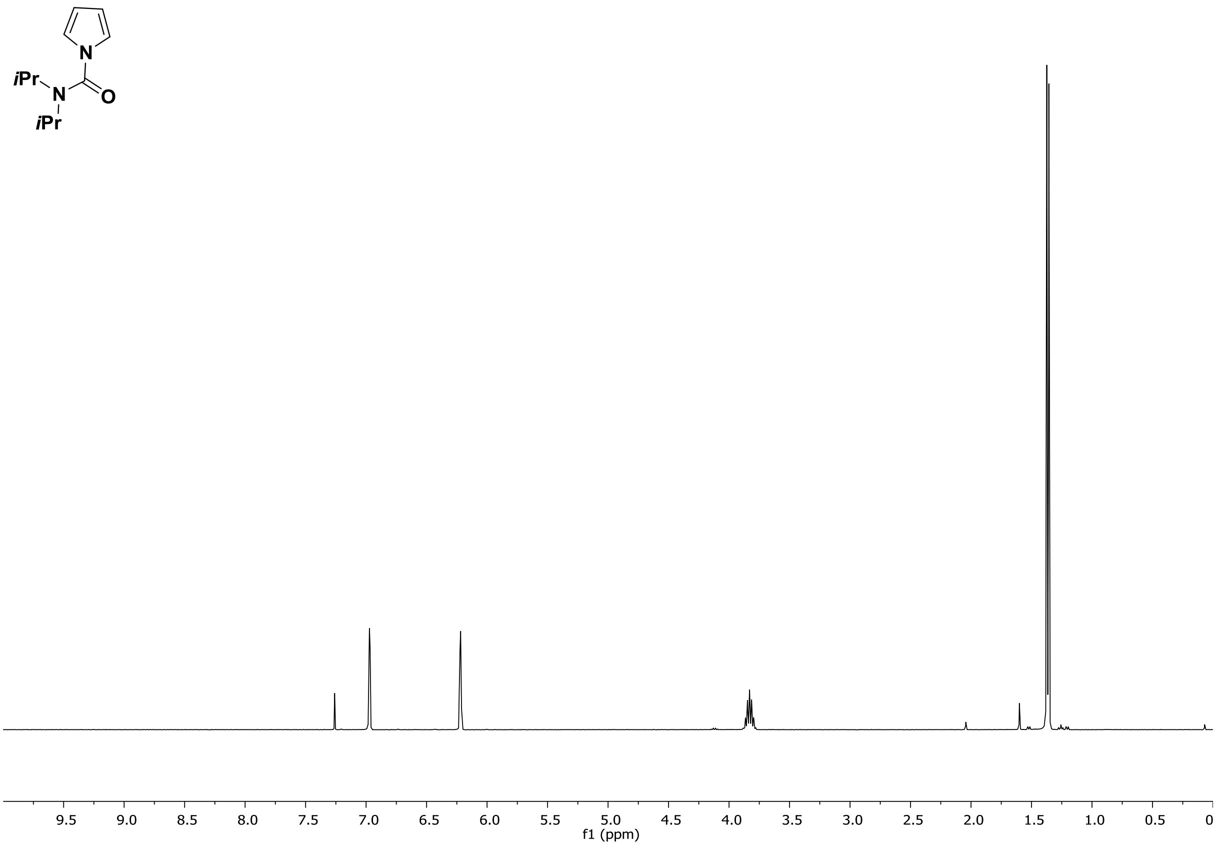
N
iPr
N
O
iPr
9.5 9.0 8.5 8.0 7.5 7.0 6.5 6.0 5.5 5.0 4.5 4.0 3.5 3.0 2.5 2.0 1.5 1.0 0.5 0
f1 (ppm)

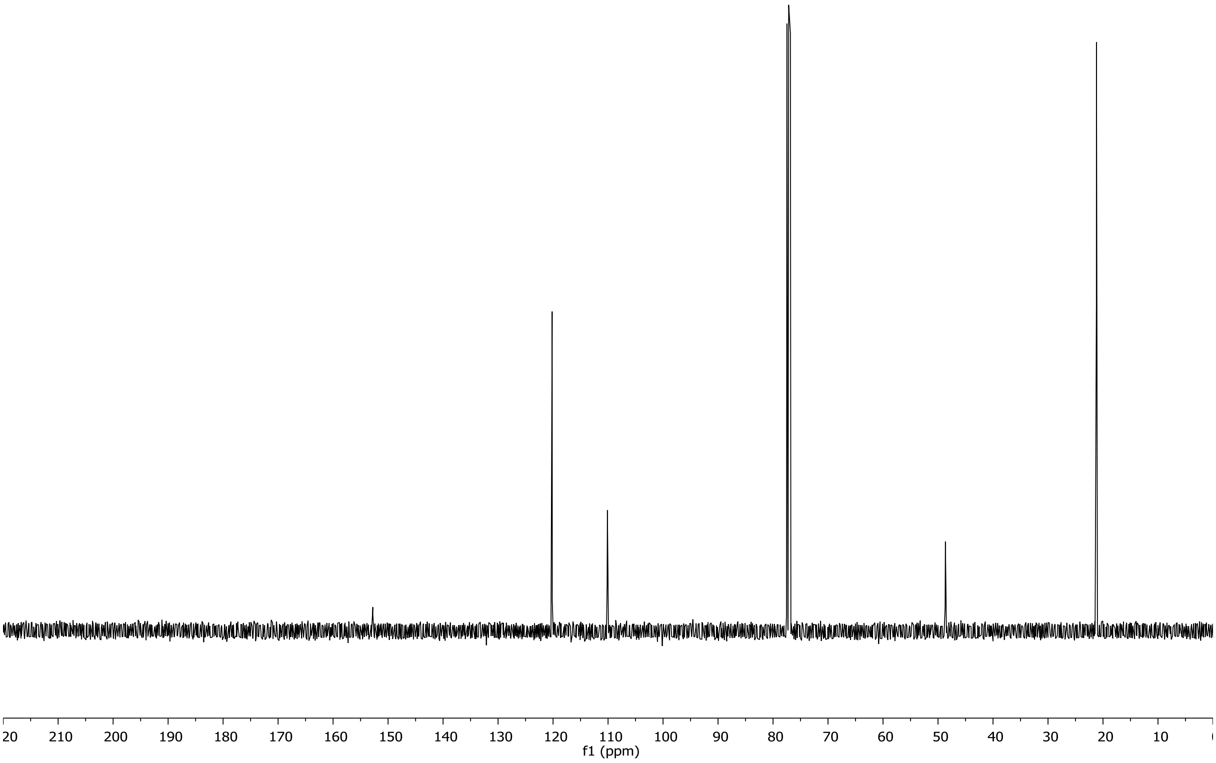
210 200 190 180 170 160 150 140 130 120 110 100 90 80 70 60 50 40 30 20 10
f1 (ppm)

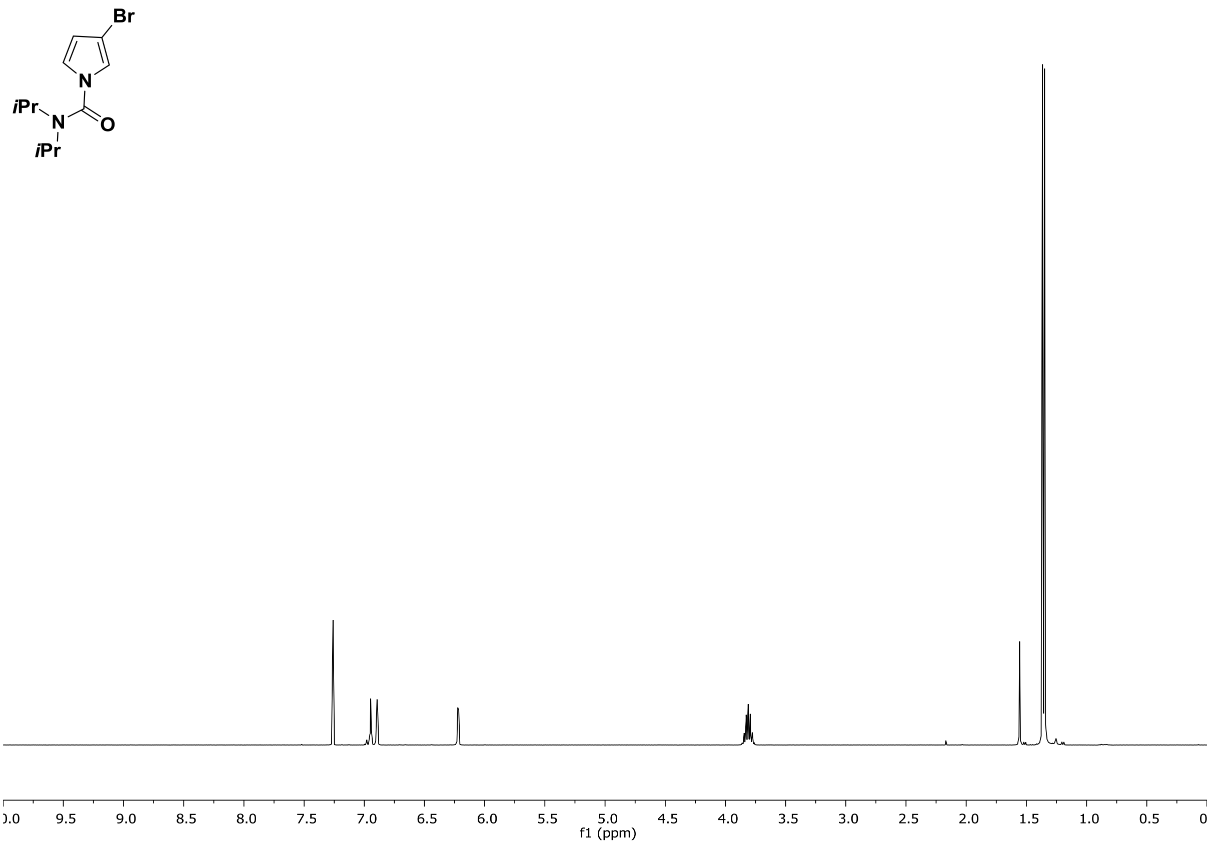
Br
N
iPr
N
O
iPr
f1 (ppm)

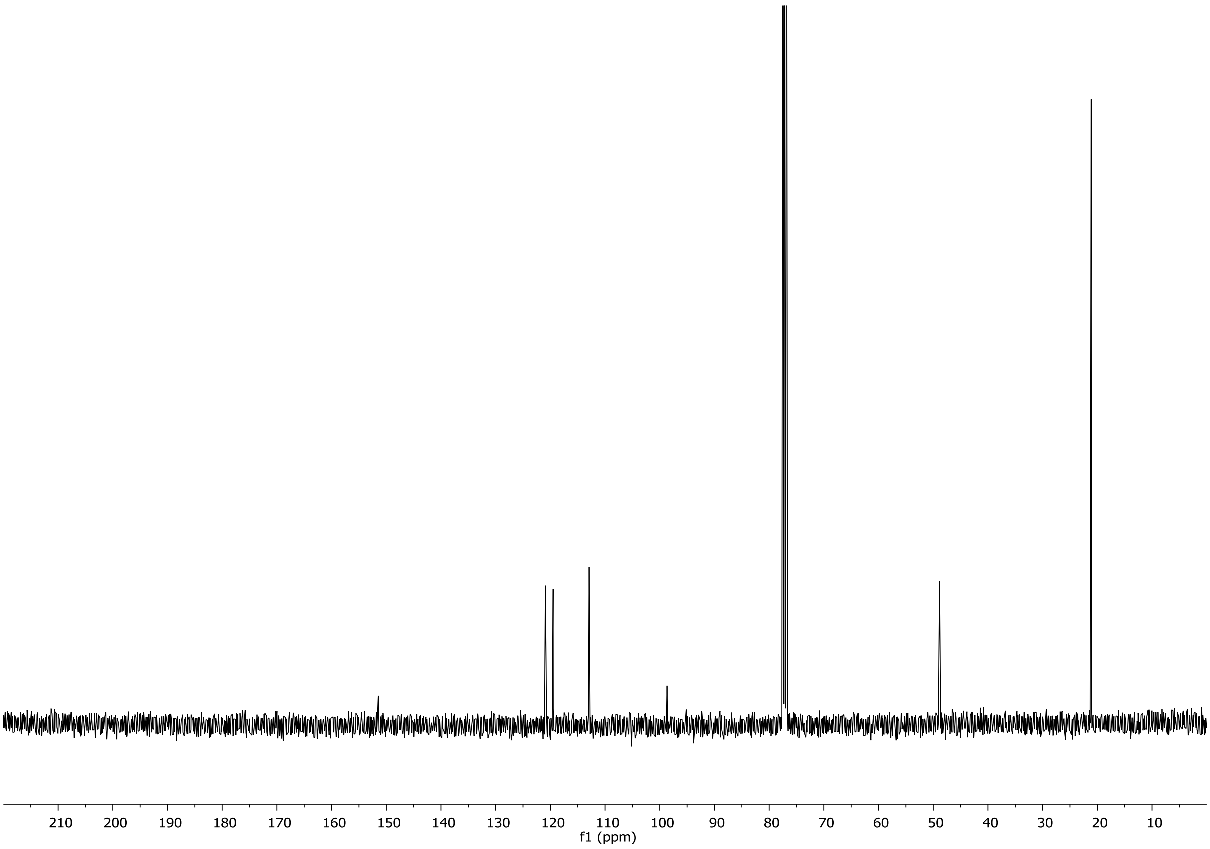
f1 (ppm)

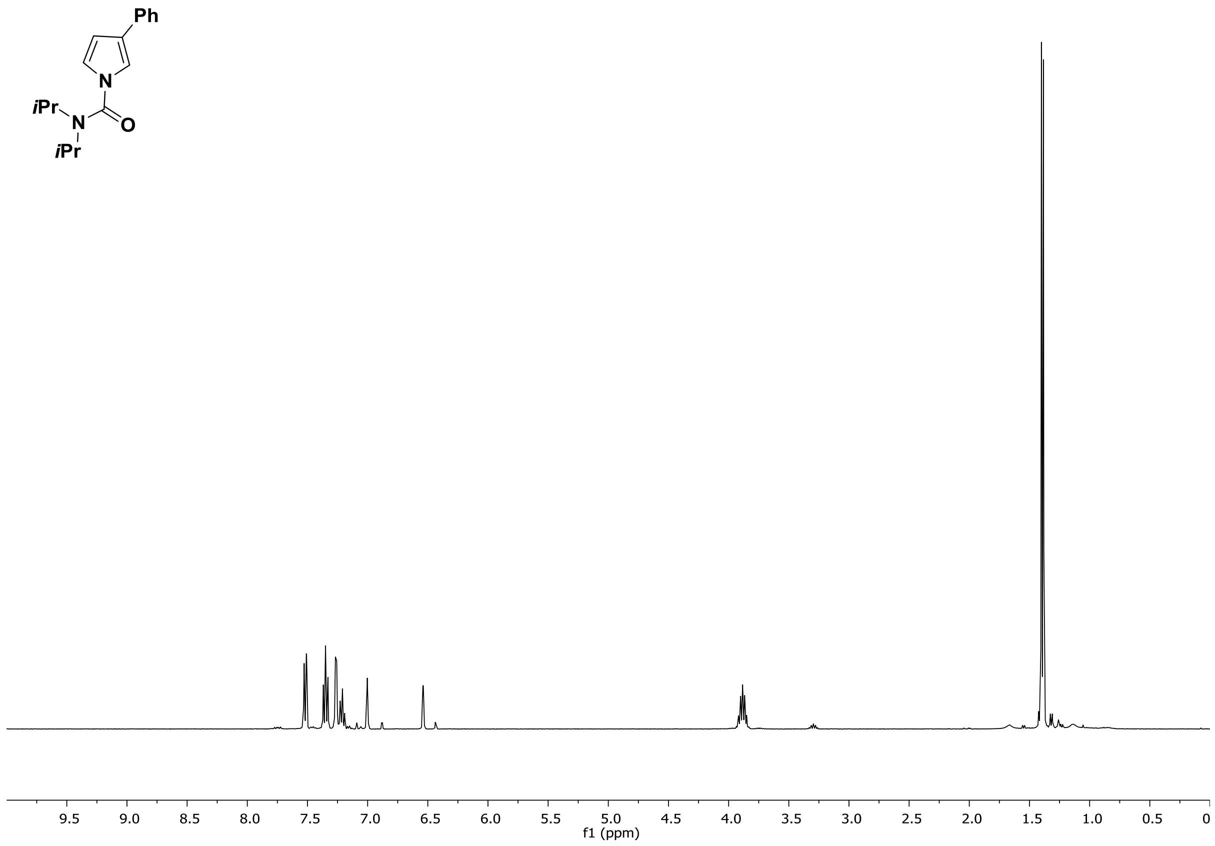
Ph
N
iPr
N
O
iPr
9.5 9.0 8.5 8.0 7.5 7.0 6.5 6.0 5.5 5.0 4.5 4.0 3.5 3.0 2.5 2.0 1.5 1.0 0.5 0
f1 (ppm)

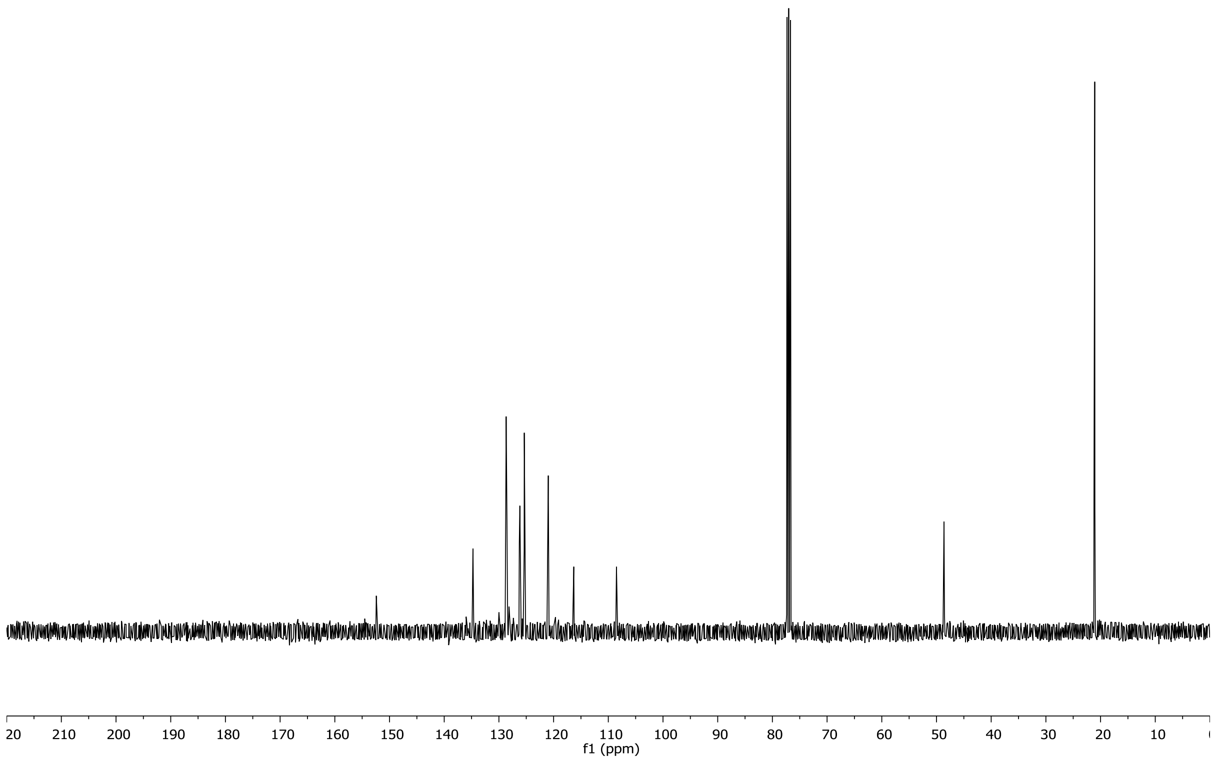
20 210 200 190 180 170 160 150 140 130 120 110 100 90 80 70 60 50 40 30 20 10
f1 (ppm)

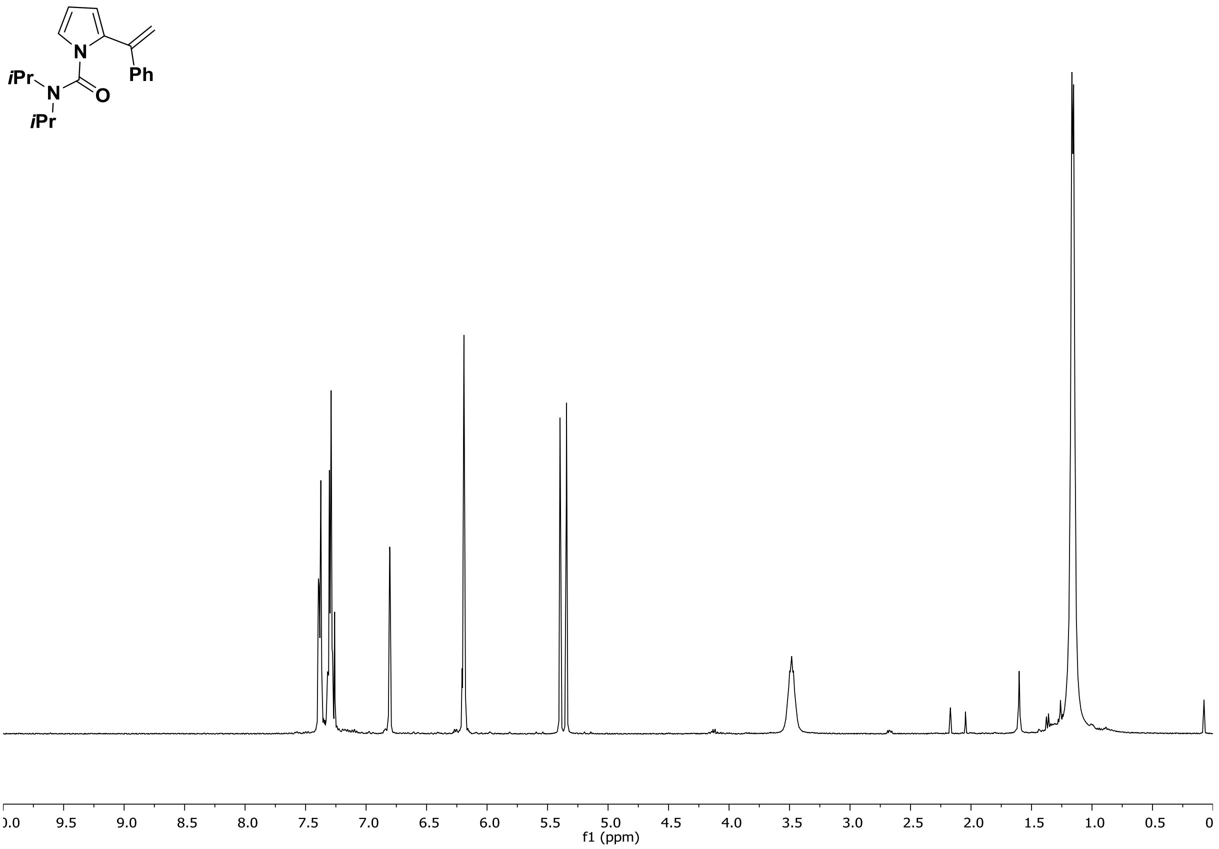

N
iPr
N
O
iPr
Ph
f1 (ppm)

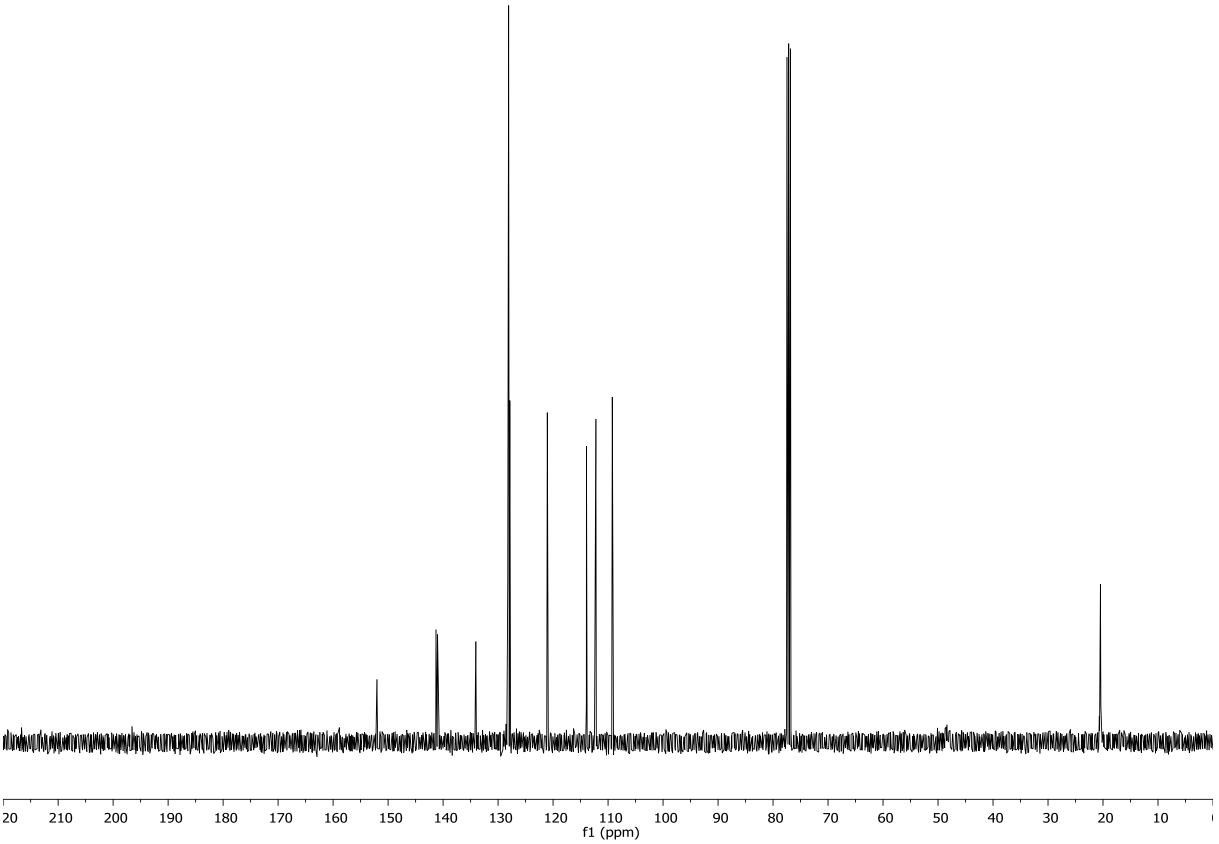

f1 (ppm)

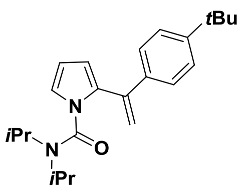
tBu
N
iPr
N
O
iPr

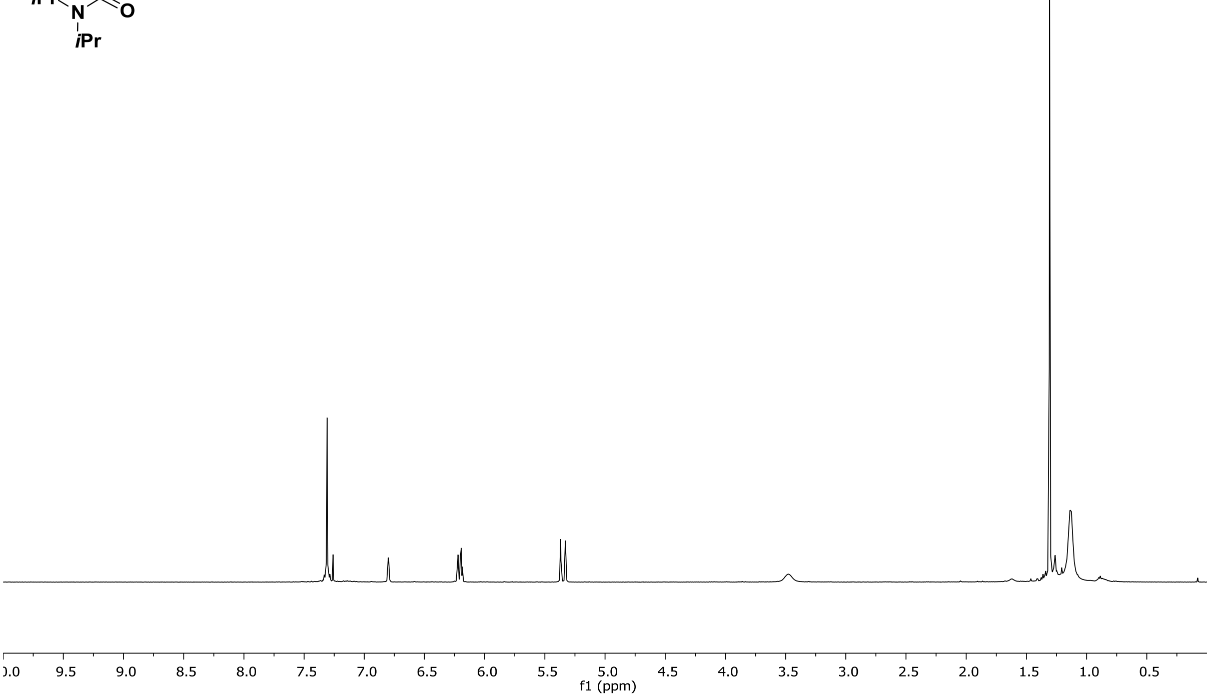
0.0 9.5 9.0 8.5 8.0 7.5 7.0 6.5 6.0 5.5 5.0 4.5 4.0 3.5 3.0 2.5 2.0 1.5 1.0 0.5
f1 (ppm)

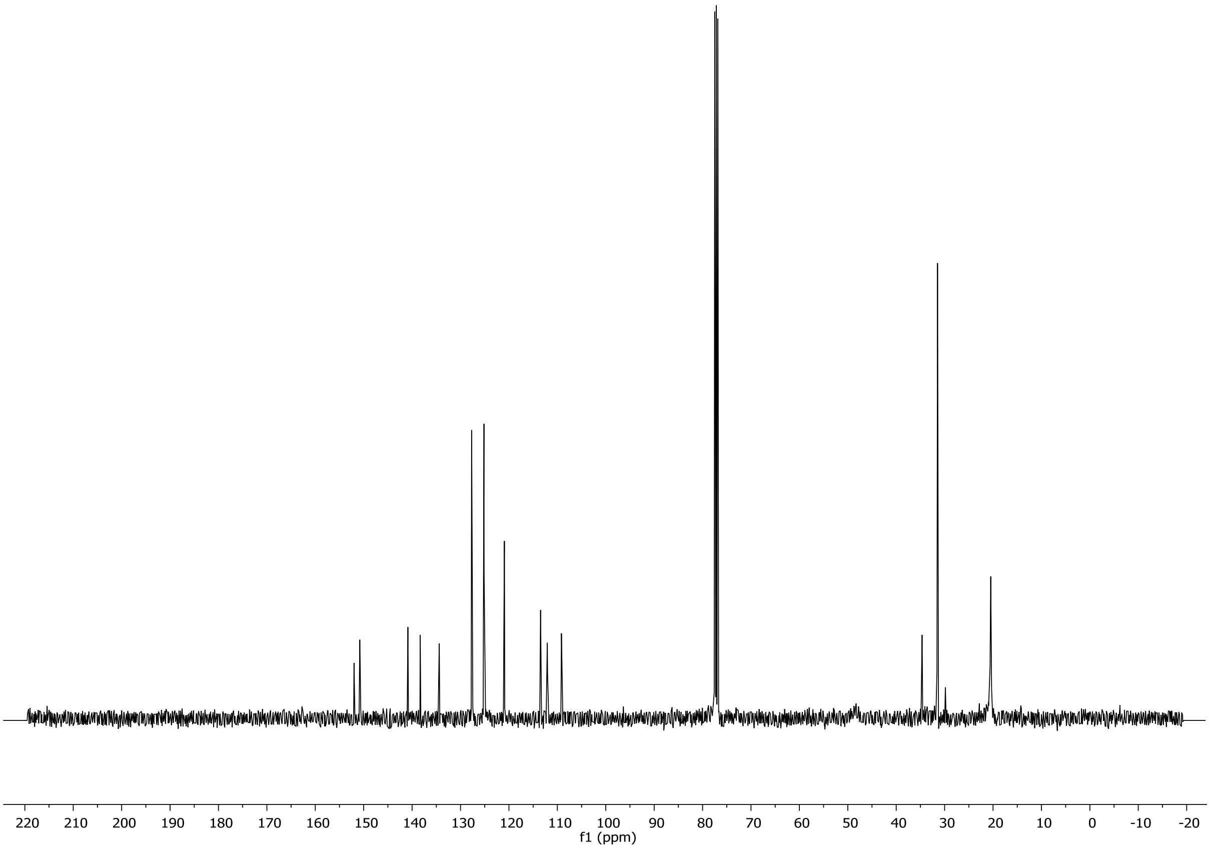
220 210 200 190 180 170 160 150 140 130 120 110 100 90 80 70 60 50 40 30 20 10 0 -10 -20
f1 (ppm)

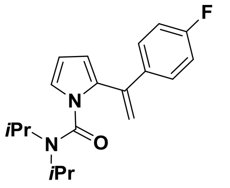
F
N
iPr
N
O
iPr

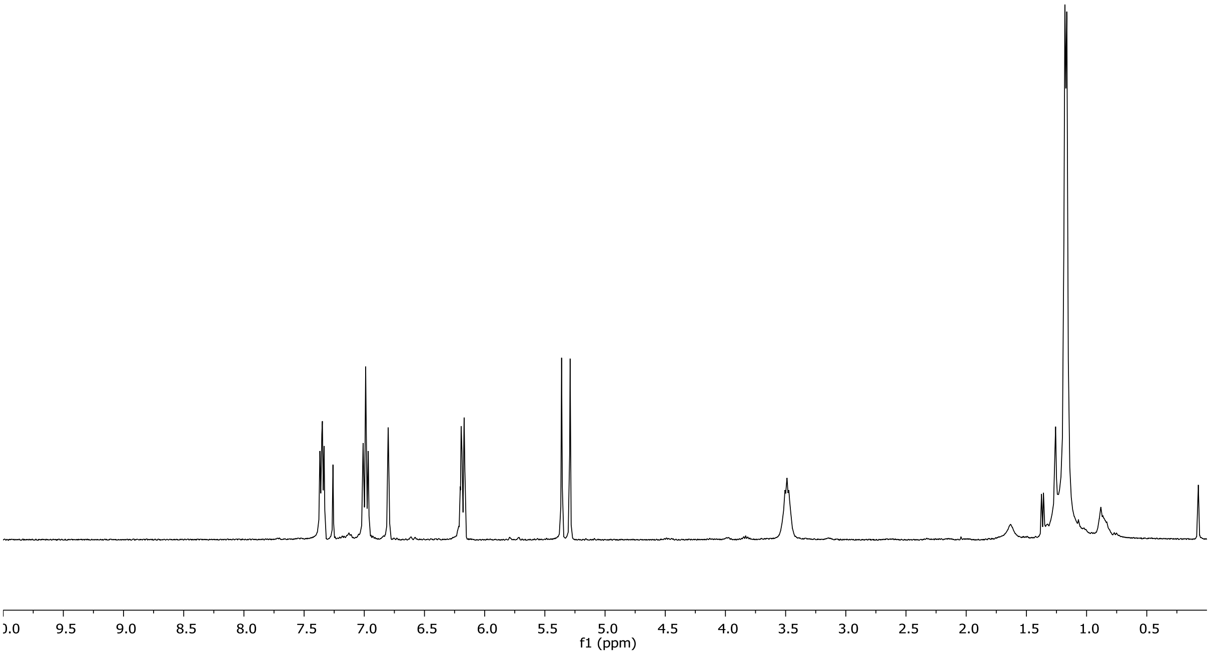
).0 9.5 9.0 8.5 8.0 7.5 7.0 6.5 6.0 5.5 5.0 4.5 4.0 3.5 3.0 2.5 2.0 1.5 1.0 0.5
f1 (ppm)

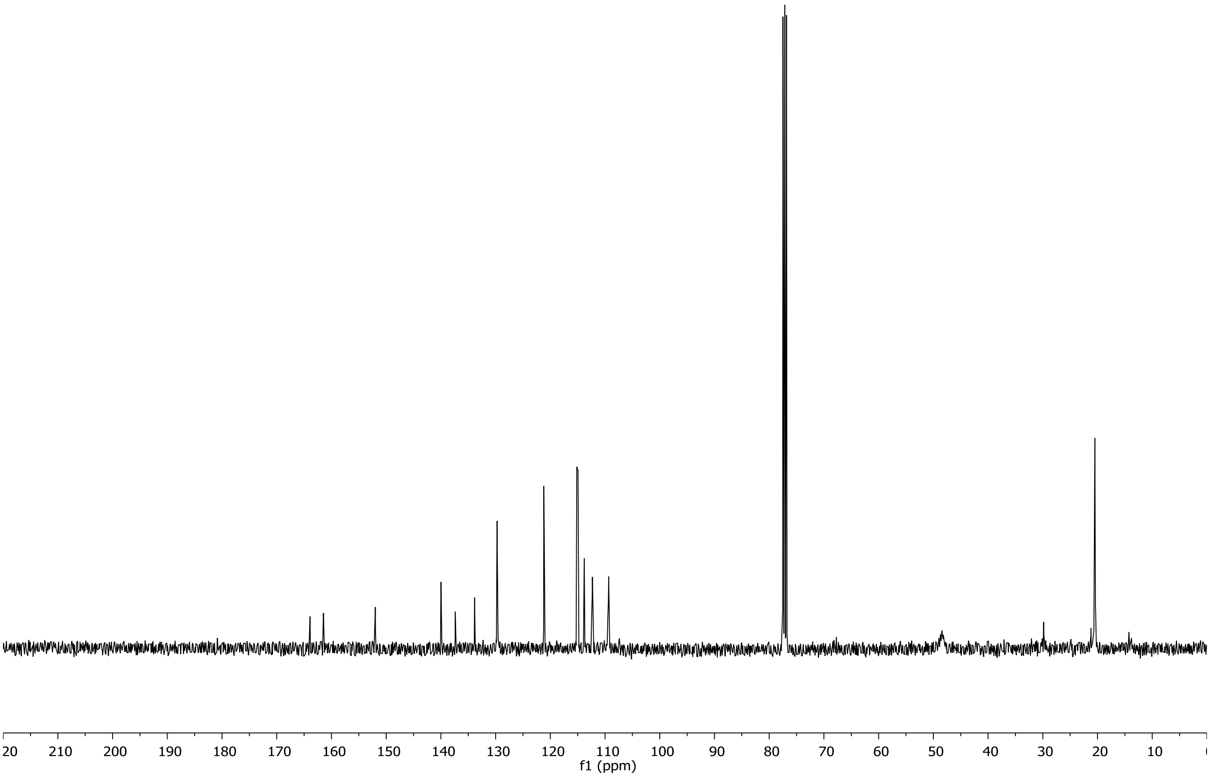
20 210 200 190 180 170 160 150 140 130 120 110 100 90 80 70 60 50 40 30 20 10
f1 (ppm)

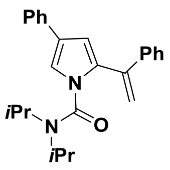
Ph
Ph
N
iPr
N
O
iPr

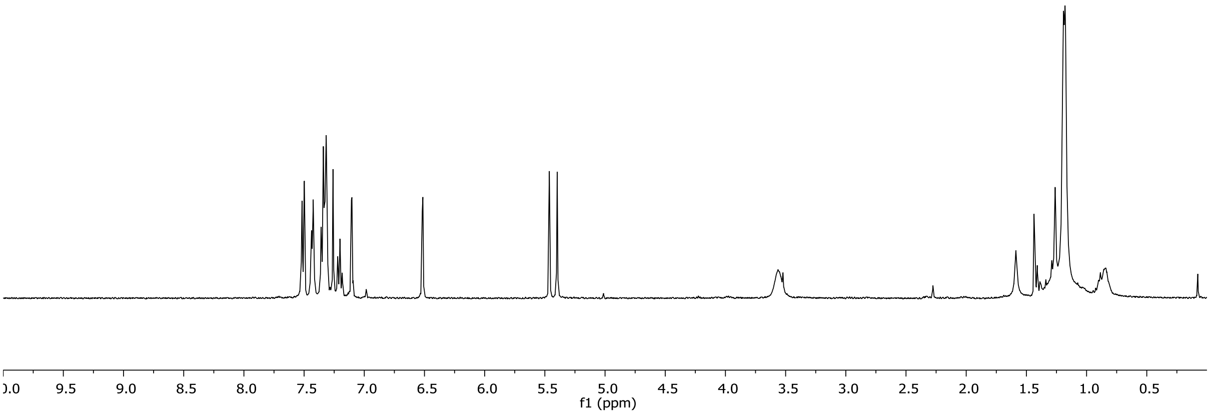
).0 9.5 9.0 8.5 8.0 7.5 7.0 6.5 6.0 5.5 5.0 4.5 4.0 3.5 3.0 2.5 2.0 1.5 1.0 0.5
f1 (ppm)

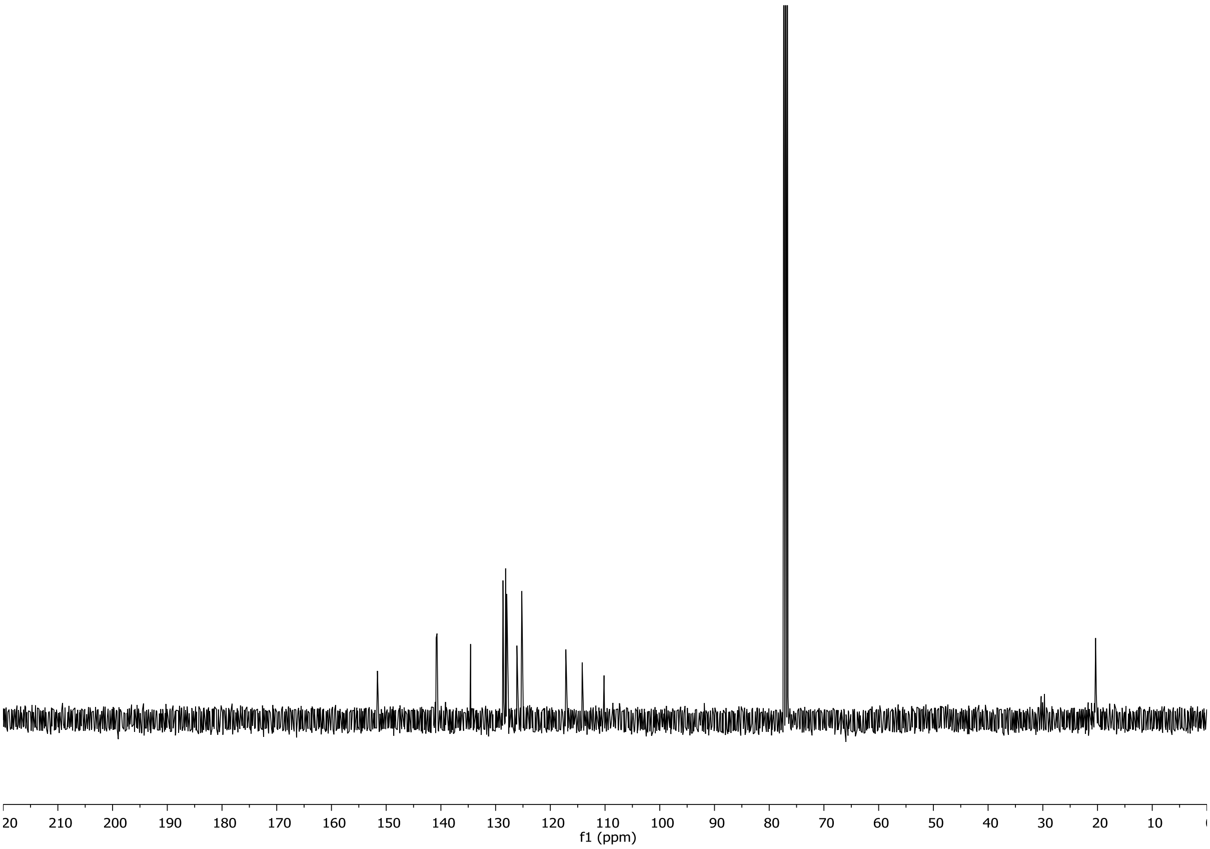
20 210 200 190 180 170 160 150 140 130 120 110 100 90 80 70 60 50 40 30 20 10
f1 (ppm)

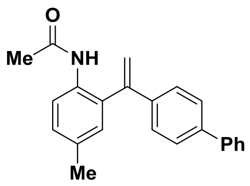
O
Me
NH
Ph
Me

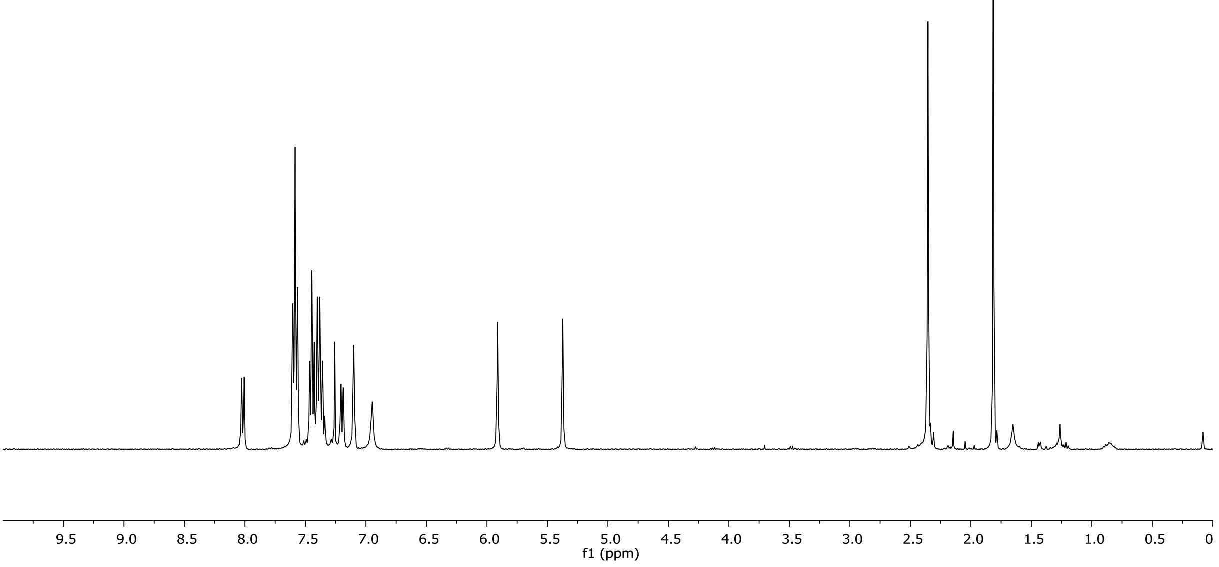
9.5 9.0 8.5 8.0 7.5 7.0 6.5 6.0 5.5 5.0 4.5 4.0 3.5 3.0 2.5 2.0 1.5 1.0 0.5 0
f1 (ppm)

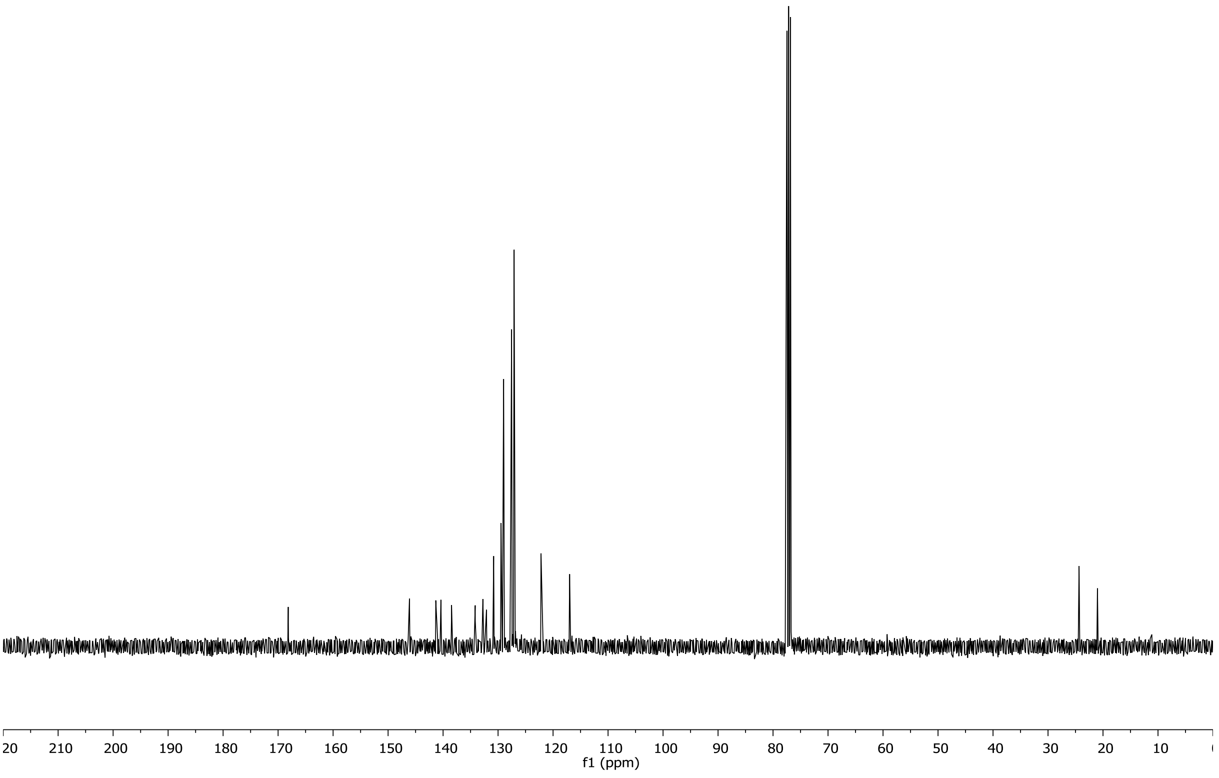
20 210 200 190 180 170 160 150 140 130 120 110 100 90 80 70 60 50 40 30 20 10
f1 (ppm)

References

1. Tsuchikama, K.; Kasagawa, M.; Endo, K.; Shibata, T. *Org. Lett.* **2009**, *11*, 1821.
2. Saunders, W. H.; Melander, L. In *Reaction Rates of Isotopic Molecules*, Wiley, New York, 1981.
3. Singleton, D. A.; Thomas, A. A. *J. Am. Chem. Soc.* **1995**, *117*, 9357.
4. Qin, L.; Ren, X.; Lu, Y.; Li, Y.; Zhou, J. *Angew. Chem. Int. Ed.* **2012**, *51*, 5915.
5. Tinnis, F.; Stridfeldt, E.; Lundberg, H.; Adolfsson, H.; Olofsson, B. *Org. Lett.* **2015**, *17*, 2688.
6. Ueda, S.; Nagasawa, H. *J. Org. Chem.* **2009**, *74*, 4272.
7. Nakatsuji, H.; Morimoto, M.; Misaki, T.; Tanabe, Y. *Tetrahedron* **2007**, *63*, 12071.
8. Crisenza, G. E. M.; Sokolova, O. O.; Bower, J. F. *Angew. Chem. Int. Ed.* **2015**, *54*, 14866.
9. O'Sullivan, S.; Doni, E.; Tuttle, T.; Murphy, J. A. *Angew. Chem. Int. Ed.* **2014**, *53*, 474.
10. Ye, W.; Mo, J.; Zhao, T.; Xu, B. *Chem. Commun.* **2009**, 3246.
11. Lebedyeva, I. O.; Biswas, S.; Goncalves, K.; Sileno, S. M.; Jackson, A. R.; Patel, K.; Steel, P. J.; Katritzky, A. R. *Chem. Eur. J.* **2014**, *20*, 11695.
12. Fang, X.; Jackstell, R.; Beller, M. *Angew. Chem. Int. Ed.* **2013**, *52*, 14089.
13. Nguyen, P.; Corpuz, E.; Heidelbaugh, T. M.; Chow, K.; Garst, M. E. *J. Org. Chem.* **2003**, *68*, 10195.
14. Karimi, B.; Behzadnia, H. *Synlett* **2010**, *2010*, 2019.
15. Chang, D.; Zhu, D.; Zou, P.; Shi, L. *Tetrahedron* **2015**, *71*, 1684.
16. De, S.; Yin, J.; Ma, D., *Org. Lett.* **2017**, *19*, 4864.
17. Zhang, M.; Ruzi, R.; Xi, J.; Li, N.; Wu, Z.; Li, W.; Yu, S.; Zhu, C. *Org. Lett.* **2017**, *19*, 3430.
18. Lipshutz, B. H.; Petersen, T. B.; Abela, A. R. *Org. Lett.* **2008**, *10*, 1333.
19. Ikawa, T.; Barder, T. E.; Biscoe, M. R.; Buchwald, S. L. *J. Am. Chem. Soc.* **2007**, *129*, 13001.
20. Langle, S.; David-Quillot, F.; Balland, A.; Abarbri, M.; Duchêne, A. *J. Organomet. Chem.* **2003**, *671*, 113.
21. Molloy, J. J.; Seath, C. P.; West, J. M.; McLaughlin, C.; Fazakerley, N. J.; Kennedy, A. R. Nelson, D. J.; Watson, A. J. B., *J. Am. Chem. Soc.* **2018**, *140*, 126.
22. Tan, H.; Houpis, I.; Liu, R.; Wang, Y.; Chen, Z.; Fleming, M. J. *Org. Process Res. Dev.* **2015**, *19*, 1044.
23. Ni, L.; Li, Z.; Wu, F.; Xu, J.; Wu, X.; Kong, L.; Yao, H. *Tetrahedron Lett.* **2012**, *53*, 1271.
24. Wolf, C.; Lerebours, R. *J. Org. Chem.* **2003**, *68*, 7551.
25. Zhao, J.-F.; Duan, X.-H.; Yang, H.; Guo, L.-N. *J. Org. Chem.* **2015**, *80*, 11149.
26. Kazuhiro, K.; Kazuna, M.; Osamu, M.; Hisatoshi, K. *Bull. Chem. Soc. Jpn.* **2005**, *78*, 886.
27. Caspers, L. D.; Finkbeiner, P. *Chem. Eur. J.* **2017**, *23*, 2748.
28. Diemer, V.; Chaumeil, H.; Defoin, A.; Fort, A.; Boeglin, A.; Carré, C. *Chem. Eur. J.* **2006**, *12*, 2727.
29. Pang, X.; Zhao, L.; Zhou, D.; He, P. Y.; An, Z.; Ni, J.; Yan, R.; *Org. Biomol. Chem.* **2017**, *15*, 6318.
30. Walter, H. *Helv. Chem. Acta*, **1994**, *77*, 608.
31. Zhao, Y.; Snieckus, V. *Adv. Synth. Catal.* **2014**, *356*, 1527.
32. Kotoku, N.; Fujioka, S.; Nakata, C.; Yamada, M.; Sumii, Y.; Kawachi, T.; Arai, M.; Kobayashi, M. *Tetrahedron* **2011**, *67*, 6673.
33. Li, T.-S.; Li, J.-T.; Li, H.-Z. *J. Chromatogr. A* **1995**, *715*, 372.
34. Frank, D. J.; Franzke, A.; Pfaltz, A. *Chem. Eur. J.* **2013**, *19*, 2405.
35. Ilchenko, N. O.; Tasch, B. O. A.; Szabó, K. J. *Angew. Chem. Int. Ed.* **2014**, *53*, 12897.
